# The third Intensive Care Bundle with Blood Pressure Reduction in Acute Cerebral Haemorrhage Trial (INTERACT3): an international, stepped wedge cluster randomised controlled trial

**DOI:** 10.1016/S0140-6736(23)00806-1

**Published:** 2023-07-01

**Authors:** Lu Ma, Xin Hu, Lili Song, Xiaoying Chen, Menglu Ouyang, Laurent Billot, Qiang Li, Alejandra Malavera, Xi Li, Paula Muñoz-Venturelli, Asita de Silva, Nguyen Huy Thang, Kolawole W Wahab, Jeyaraj D Pandian, Mohammad Wasay, Octavio M Pontes-Neto, Carlos Abanto, Antonio Arauz, Haiping Shi, Guanghai Tang, Sheng Zhu, Xiaochun She, Leibo Liu, Yuki Sakamoto, Shoujiang You, Qiao Han, Bernard Crutzen, Emily Cheung, Yunke Li, Xia Wang, Chen Chen, Feifeng Liu, Yang Zhao, Hao Li, Yi Liu, Yan Jiang, Lei Chen, Bo Wu, Ming Liu, Jianguo Xu, Chao You, Craig S Anderson, INTERACT3 Investigators, Thompson Robinson, J. Jaime Miranda, Craig S. Anderson, Chao You, Lili Song, Adrian Parry-Jones, Nikola Sprigg, Sophie Durrans, Caroline Harris, Ann Bamford, Olivia Smith, Robert Herbert, Christopher Chen, William Whiteley, Rong Hu, Laurent Billot, Qiang Li, Jayanthi Mysore, Xin Hu, Yao Zhang, Feifeng Liu, Yuki Sakamoto, Shoujiang You, Qiao Han, Bernard Crutzen, Yunke Li, Emily Cheung, Stephen Jan, Hueiming Liu, Menglu Ouyang, Lingli Sun, Honglin Chu, Anila Anjum, Francisca Gonzalez Mc Cawley, Alejandra Del Rio, Bruna Rimoli, Rodrigo Cerantola, Thanushanthan Jeevarajah, Madhushani Kannangara, Andrene Joseph, Chamath Nanayakkara, Xiaoying Chen, Alejandra Malavera, Chunmiao Zhang, Zhao Yang, Brook Li, Zhuo Meng, Menglu Ouyang, Leibo Liu, Yi Ning, Le Dong, Manuela Armenis, Joyce Lim, Helen Monaghan, Lu Ma, Xin Hu, Xi Li, Rui Luo, Guojuan Cheng, Yilin Dong, Ziqin Liu, Shuihong Wang, Ying Zhang, Jipeng Cheng, Hui Shi, Wenjing Li, Langming Mou, Ping Yi, Chen Chen, Xue Chen, Shalomi Weerawardena, Poornima Ellawala, Enalee Ranasinghe, Chrishmi Rodrigo, Kolawala Wahab, Sunday Adeniyi, Jeyaraj Pandian, Megha Khanna, Paula Muñoz Venturelli, Francisca González, Francisca Urrutia Goldsack, Mohammad Wasay, Dilshad Begum, Anila Anjum, Octavio Pontes-Neto, Millene Camilo, Francisco Dias, Octavio Vincenzi, Rodrigo Cerantola, Carla Moro, Renata Santos, Nara Texeira, Alexandre Longo, Rafaela Liberato, Sheila Martins, Arthur Pille, Bruna Chwal, Isabel Silva, Natacha Titton, Gustavo Weiss, Daissy Mora, Magda Ouriques, Leonardo Carbonera, Rodrigo Bazan, Gabriel Modolo, Fernanda Winckler, Luana Miranda, Juli Souza, Alexis Rojo, Wilhelm Uslar, Lorena Medel, Javiera Lopez, Diego Herrero, Pablo Lavados, Barbara Vargas Latorre, Nathalie Conejan, Tomas Esparza, Patricio Sotomayor, Denisse Wenger, Juan Pablo Gigoux, Aldo Letelier, Lilian Acevedo, Vivianne Moya, Cristian Figueroa, Nicol Vallejos, Nathalie Conejan, Tomas Esparza, Patricio Sotomayor, Rodrigo Guerrero, Mauricio Velasquez, Jose Vallejos, Kimerly Pallauta, Tamara Santibanez, Angelo Queirolo, Andrea Lobos, Yongming Jiang, Weimin Li, Wei Huang, Ke Luo, Gangying Liu, Guanghai Tang, Guang Yang, Hongtao Jiang, Xu Zhang, Hongyan Jing, Sheng Zhu, Bo Pu, Dong Lv, Hui Kang, Qiuping Hu, Xiaochun She, Xiaoming Jiang, Yanli Chen, Shenghua Yang, Jianjun He, Zongping Li, Gang Cheng, Hailin Huang, Xiaoyi Wang, Jianqiong Lin, Minhui Chen, Chenghao Yang, Hao Ding, Yunliang Deng, Fei Luo, Rongjun Zhang, Xiaofeng Wang, Hongbing Zhang, Xiaoliang Yang, Yang Zhang, Chengyi Yang, Yu He, Feng Liu, Rongjie Wang, Yuhui Zhang, Xiaodong Xin, Bin Feng, Wanru Hao, Chang Song, Yun Guo, Dehua Jiang, Jie Chen, Changtong Tang, Hongliang Zhu, Xin Li, Jin Cui, Haidong Xu, Boyang Li, Fusheng Tang, Yuanbin Li, Min Gao, Bo Yang, Xuejun Xu, Bing Deng, Yi Zheng, Yuanhong Ge, Keyu Chen, Yang Liu, Xinshen Li, Tingting Zhong, Jianfeng Xu, Hai Zhang, Jiyue Wang, Jianxin Zhu, Hanyu Sun, Fuhua Yu, Xueguang Zhang, Chao You, Lu Ma, Xin Hu, Jianguo Xu, Xi Li, Mingsen Zhang, Bin Wang, Yiming Ma, Donglin Jiang, Jun Zhou, Cong Liu, Wenhong Nie, Mingguo Li, Tao Tian, Yong Li, Mingfang He, Xiaolong Tu, Zhengjun Wu, Hong Liu, Dongsheng Zhong, Rongcai Jiang, Jian Sun, Ye Tian, Yingsheng Wei, Shuo An, Pingbo Wei, Le Luo, Bin Lin, Gang Liu, Yan Wen, Qiang Cai, Qianxue Chen, Pan Lei, Zhiyang Li, Meifang Zhang, Jiaquan He, Yan Chen, Jun Liu, Xinghai Liu, Junyan Li, Min Chen, Jing Wang, Bingzhi Zhou, Baichun Ye, Jiancheng Zhang, Manyuan Zhang, Xuming Pan, Xiaoxiang Yu, Jian Xu, Qingbao Xiao, Yuefei Wang, Liang Tao, Lin Shi, Niandong Zheng, Guoliang You, Bo Lei, Shu Chen, Honggang Wu, Jin Hu, Jianlan Zhao, Jian Yu, Qiang Yuan, Zhuoying Du, Xielin Tang, Qianke Li, Shenghua Liu, Feilong Yang, Kui Xiao, Chao Luo, Guang Wang, Xudong Che, Zhipeng Teng, Wenwu Wan, Jun Li, Yu Liu, Mingbo Fan, Tao Zhang, Lun Cai, Yuan Ma, Zhifeng Ma, Bin Li, Linlin He, Jinghui Li, Weibing Zhang, Shuxin Zhang, Hongzhen Zhang, Yingguang Dai, Jun Lei, Lei Mao, Yiyang Huang, Zhi Zhou, Ping Chen, Fang Chen, Pan Wei, Tiangui Li, Honglin Chen, Mengfei Zeng, Kejie Mou, Jun Xue, Yong Jiang, Xiaoping Tang, Tao Chen, Yalan Zhang, Yanbing Xu, Yuchen Gu, Lei Chen, Yujun Zhao, Bin Yang, Peng Kuai, Xi Wang, Yuwang Yang, Xueling Hu, Huitian Zhang, Yintao Yang, Weifeng Wang, Junyi Zhang, Wei Cheng, Xiaoxue Zhang, Xiaowen Ma, Qin He, Li Zhang, Rong Gao, Huixiang Liu, Jingwei Ye, Ping Xu, Xin Wu, Yuan Yuan, Peng Zou, Zhen Zhang, Jiyong Cheng, Zhangming Zhou, Yijun Zeng, Zhang Liang, Deming Du, Shui Yu, Yongjun Cao, Shoujiang You, Jiaping Xu, Zhichao Huang, Dongqin Chen, Wenfeng Xiao, Li Zhu, Miao Yuan, Yuhai Wang, Dongliang Shi, Xu Hu, Dingchao Xiang, Like Shi, Hongqin Wang, Liu Yang, Wang Miao, Yiyi Hu, Yuchun Zhao, Xi Hu, Yang Liu, Weiduo Zhou, Chao Sun, Tao Chen, Dong Tang, Kun Yao, Jin You, Shishi Chen, Jianmin Yao, Huanmei Li, Jinmei Liu, Ailin Bai, Yong Yi, Qingshan Deng, Peng Luo, Han Wang, Jingcheng Jiang, Qingwei Yang, Shunpo He, Jun Wang, Yu Chen, Hua He, Yuyang Deng, Zhikai Cao, Xuxia Yi, Jinbiao Luo, Shuang Luo, Min Gong, Li Liu, Xuejun Gao, Jia Liu, Li'e Wu, Jia Zhang, Hongying Sun, Xinhui Li, Lu Jia, Jianbing Wu, Jie Zhang, Huajun Zhang, Chunfu Du, Shun Li, Xiaobin Yang, Jie He, Lei Liao, Gezhi Zhou, Wentao Dong, Yunxiang Chen, Xiaofeng Lin, Xujian Shui, Peng Zhang, Yuan Zhao, Hongli Yang, Wenbin Zhao, Xiaoyi Zhang, Jincao Chen, Qian Wu, Xuan Dai, Baogui Tang, Yinjuan Wang, Tao Liu, Haixia Zhang, Faliang Duan, Ming Luo, Qingfang Jiao, Guoliang Lei, Dong Wang, Chunwang Song, Haopeng Tan, Feng Ye, Xinghu Qin, Xiaolong Liang, Junling Liu, Lang Yang, Jie Yang, Yapeng Lin, Qian Yang, Xuntai Ma, Yinkuang Qi, Baogen Pan, Caixia Jiang, Zhanying Ye, Ce Dong, Xiongfei Yue, Xiaopeng Yang, Tuoheti Maimaitiyiming, Jun Dong, Yonggang Wu, Feng Gao, Deqiang Zhao, Xinghai Zhang, PengJun Wang, Hongbo Jiang, Jianping Li, Wei Zhang, Jing Chen, Haibo Tong, Yonghong Wang, Kaipeng Qiao, Fuyou Guo, Mingchu Zhang, Yan Hu, Mengzhao Feng, Dengpan Song, Yi Zuo, Shangjun Chen, Chao Qian, Baoming Li, Jingku Ma, Sunfu Zhang, Bin Kong, Xingyu Dong, Qiang Li, Sheng Fang, Bin Lu, Yang Li, Zhen Zhang, Yongling Yang, Hong Yu, Huaiyu Sun, Yue Wang, Weimin Wang, Tong Li, Shengli Li, Zhiming Xu, Yongyi Wang, Qiang Dong, Yuping Tang, Heling Chu, Ying Lu, Zhong Wang, Xiaoou Sun, Jianhua Zhao, Shuaifeng Yang, Xiying Qian, Aralikatte Onkarappa Saroja, Ravishankar Naik k, Sandip Chindhi, Nakul Pampaniya, Kurubara Amaresh, Thomas Iype, Dileep R, Reeja Rajan, Praveen Panicker, Rupjyoti Das, Nupur Choudhury, Pankaja Gohain, Jemin Webster, Biyol Pakma, Lalbiak Sangi, Ivy Sebastian, Gaurav Aggrawal, Komal Raj, Deepankshi Rajoura, Sulena Singh, Varun Aggrawal, Amit Narang, Antonio Arauz, Vanesa Cano-Nigenda, Diego López-Mena, Héctor Valdez-Ruvalcaba, Roberto Toledo-Treviño, Reginald Obiako, Sani Abubakar, Oguike Emeka, Balogun Olayemi, Melika Lois, Ibinaiye Philip, Olurishe Comfort O, Njideka Okubadejo, Osigwe Agabi, Oluwadamilola Ojo, Kolawole Wahab, Abiodun Bello, Oyinloye Ibukun, Olufemi Sanayaolu, Sunday Adeniyi, Abdulraheem Jimoh, Mohammad Wasay, Dilshad Begum, Anila Anjum, Shahid Waheed, Dr.Ayeesha Kamal, Raja Farhat Shoaib, Fizza Orooj, Sadaf Majid, Taskeen Zehra, Abdus Salam Khan, Ravi Shanker, Nadir Ali Syed, Nashwa Ahmad, Carlos Abanto, Ana Valencia, Danny Barrientos, Jorge Ramirez, Pilar Calle, Dilum Palliyeguruge, Sumudu Muthucumarana, Shiroma Ratnayaka, Dilhara Ganihiarachchi, Arundathi Bandaranayake, S.D.B Somaratne, Saumya Narayana, Sithara Gallage, Bimsara Senanayake, Udari Samarasiri, Dunya Luke, Mythily Sivapathasundaram, Vithoosan Sahadevan, Amani Rasmi, Yuran Deshaka, Nilukshi Fernando, Aruna Munasinghe, Kapilanga Rathnapriya, A.S Nissanka, Kanchana Karunathilake, Isuru Gayan, Kaminda Wijenayake, Hasitha Gunasekara, Jagath Vidyarathne, Ajantha Keshavaraj, Kanagasabapathy Janarthanan, Arhivalaky Gerald Jeevathasan, Sivaram Sivamainthan, Mathyamuthan John Priyanth, Abirami John Priyanth, Thambippillai Rajendiran, Sanjeewa Alwis, Nushara Gunasekare, Vasundara Liyanarachchi, Athula Dissanayake, Wimalasiri Mewa Uluwattage, Gimhani Ratnayake, Charika Rajinee, Sakura Jayawardana, Janaka Peiris, Ranjith Wicramasinghe, Chamila Fernando, Jessie Abbas, Nethmini Withanage, Makaranda Bandara, Duy Ton Mai, Van Chi Nguyen, Viet Phuong Dao, Xuan Trung Vuong, Tien Dung Nguyen, Trung Hieu Dinh, Ha Quan Phan, Quoc Viet Bui, Dinh Tho Phung, Quang Tho Pham, Dinh Dai Pham, Duc Thuan Do, Phuc Duc Dang, Minh Duc Dang, Dang Hai Nguyen, Thi Phuong Nga Nguyen, Quoc Huy Nguyen, Quoc Dai Pham, Quoc Vinh Chau, Vinh Thy Van Tai, Tran Vinh Le, Cong Tri Le, Ha Mai Khuong Tran, Huu Khanh Nguyen, Hoang Minh Thao Ngyen, Duc Chien Vo, Thai My Phuong Nguyen, Trung Thanh Tran, Thi Hanh Vi Vo, Hao Nhien Cao, Ba Thang Nguyen, Thi Ngoc Suong Le, Thien Duc La, Chi Duc Pham, Huy Thai

**Affiliations:** a Department of Neurosurgery, West China Hospital, Sichuan University, Chengdu, China; b Department of Neurology, West China Hospital, Sichuan University, Chengdu, China; c The George Institute for Global Health China, Beijing, China; d The George Institute for Global Health, Faculty of Medicine, University of New South Wales, Sydney, NSW, Australia; e Clinical Research Center, Faculty of Medicine Clinica Alemana Universidad del Desarrollo, Santiago, Chile; f Clinical Trials Unit, Faculty of Medicine, University of Kelaniya, Colombo, Sri Lanka; g Stroke Unit, 115 Hospital, Ho Chi Minh City, Viet Nam; h Department of Medicine, University of Ilorin & University of Ilorin Teaching Hospital, Ilorin, Nigeria; i Neurology Department, Christian Medical College and Hospital, Ludhiana, India; j Department of Medicine, The Aga Khan University, Karachi, Pakistan; k Department of Neurology, Ribeirão Preto Medical School, University of São Paulo, São Paulo, Brazil; l The Cerebrovascular Disease Research Center, National Institute of Neurological Sciences, Lima, Peru; m Instituto Nacional de Neurologia y Neurocirugia Manuel Velasco Suarez, Mexico City, Mexico; n Department of Neurosurgery, Suining Central Hospital, Suining, China; o Department of Neurology, Liaoning Thrombus Treatment Centre of Integrated Chinese and Western Medicine, Shenyang, China; p Department of Neurosurgery, Dazhu County People's Hospital, Dazhou, China; q Department of Neurosurgery, Jiangsu Rudong County People's Hospital, Nantong, China; r Department of Neurology, Graduate School of Medicine, Nippon Medical School, Tokyo, Japan; s Department of Neurology and Clinical Research Center of Neurological Disease, The Second Affiliated Hospital of Soochow University, Suzhou, China; t Department of Neurology, Suzhou Hospital of Traditional Chinese Medicine, Suzhou, China; u Department of Radiology, Cliniques Universitaires Saint-Luc, Brussels, Belgium; v Department of Radiology, Grand Hôpital de Charleroi, Charleroi, Belgium; w Neurology Department, Royal Prince Alfred Hospital, Sydney, Australia; x Department of Neurology, Shanghai East Hospital, Tongji University, Shanghai, China; y Department of Nursing and Evidence-based Nursing Center, West China Hospital, Sichuan University, Chengdu, Sichuan, China; z Heart Health Research Center, Beijing, China

## Abstract

**Background:**

Early control of elevated blood pressure is the most promising treatment for acute intracerebral haemorrhage. We aimed to establish whether implementing a goal-directed care bundle incorporating protocols for early intensive blood pressure lowering and management algorithms for hyperglycaemia, pyrexia, and abnormal anticoagulation, implemented in a hospital setting, could improve outcomes for patients with acute spontaneous intracerebral haemorrhage.

**Methods:**

We performed a pragmatic, international, multicentre, blinded endpoint, stepped wedge cluster randomised controlled trial at hospitals in nine low-income and middle-income countries (Brazil, China, India, Mexico, Nigeria, Pakistan, Peru, Sri Lanka, and Viet Nam) and one high-income country (Chile). Hospitals were eligible if they had no or inconsistent relevant, disease-specific protocols, and were willing to implement the care bundle to consecutive patients (aged ≥18 years) with imaging-confirmed spontaneous intracerebral haemorrhage presenting within 6 h of the onset of symptoms, had a local champion, and could provide the required study data. Hospitals were centrally randomly allocated using permuted blocks to three sequences of implementation, stratified by country and the projected number of patients to be recruited over the 12 months of the study period. These sequences had four periods that dictated the order in which the hospitals were to switch from the control usual care procedure to the intervention implementation of the care bundle procedure to different clusters of patients in a stepped manner. To avoid contamination, details of the intervention, sequence, and allocation periods were concealed from sites until they had completed the usual care control periods. The care bundle protocol included the early intensive lowering of systolic blood pressure (target <140 mm Hg), strict glucose control (target 6·1–7·8 mmol/L in those without diabetes and 7·8–10·0 mmol/L in those with diabetes), antipyrexia treatment (target body temperature ≤37·5°C), and rapid reversal of warfarin-related anticoagulation (target international normalised ratio <1·5) within 1 h of treatment, in patients where these variables were abnormal. Analyses were performed according to a modified intention-to-treat population with available outcome data (ie, excluding sites that withdrew during the study). The primary outcome was functional recovery, measured with the modified Rankin scale (mRS; range 0 [no symptoms] to 6 [death]) at 6 months by masked research staff, analysed using proportional ordinal logistic regression to assess the distribution in scores on the mRS, with adjustments for cluster (hospital site), group assignment of cluster per period, and time (6-month periods from Dec 12, 2017). This trial is registered at Clinicaltrials.gov (NCT03209258) and the Chinese Clinical Trial Registry (ChiCTR-IOC-17011787) and is completed.

**Findings:**

Between May 27, 2017, and July 8, 2021, 206 hospitals were assessed for eligibility, of which 144 hospitals in ten countries agreed to join and were randomly assigned in the trial, but 22 hospitals withdrew before starting to enrol patients and another hospital was withdrawn and their data on enrolled patients was deleted because regulatory approval was not obtained. Between Dec 12, 2017, and Dec 31, 2021, 10 857 patients were screened but 3821 were excluded. Overall, the modified intention-to-treat population included 7036 patients enrolled at 121 hospitals, with 3221 assigned to the care bundle group and 3815 to the usual care group, with primary outcome data available in 2892 patients in the care bundle group and 3363 patients in the usual care group. The likelihood of a poor functional outcome was lower in the care bundle group (common odds ratio 0·86; 95% CI 0·76–0·97; p=0·015). The favourable shift in mRS scores in the care bundle group was generally consistent across a range of sensitivity analyses that included additional adjustments for country and patient variables (0·84; 0·73–0·97; p=0·017), and with different approaches to the use of multiple imputations for missing data. Patients in the care bundle group had fewer serious adverse events than those in the usual care group (16·0% *vs* 20·1%; p=0·0098).

**Interpretation:**

Implementation of a care bundle protocol for intensive blood pressure lowering and other management algorithms for physiological control within several hours of the onset of symptoms resulted in improved functional outcome for patients with acute intracerebral haemorrhage. Hospitals should incorporate this approach into clinical practice as part of active management for this serious condition.

**Funding:**

Joint Global Health Trials scheme from the Department of Health and Social Care, the Foreign, Commonwealth & Development Office, and the Medical Research Council and Wellcome Trust; West China Hospital; the National Health and Medical Research Council of Australia; Sichuan Credit Pharmaceutic and Takeda China.

## Introduction

Intracerebral haemorrhage is the most serious and least treatable form of stroke and accounts for approximately 20% of the almost 20 million new strokes that occur globally each year.^1^ Most cases of intracerebral haemorrhage occur in low-income and middle-income countries (LMICs), where there is a high prevalence of hypertension, unhealthy diets (eg, a high salt intake), and other risk factors.^2^ Because elevated blood pressure is common after the onset of intracerebral haemorrhage and is strongly associated with a poor outcome, a central component of the management of patients is to provide treatment to lower blood pressure towards a systolic target of 140 mm Hg or less.^3^, ^4^, ^5^, ^6^, ^7^ However, inconsistent results across randomised controlled trials^8^, ^9^ and little evidence specifically in patients with a large intracerebral haemorrhage or who require neurosurgical intervention^10^ has restricted the uptake of this strategy in clinical practice where guidelines generally provide an intermediate strength to their recommendation.^3^, ^4^, ^5^, ^6^, ^7^ Moreover, the absence of a proven medical or surgical treatment for intracerebral haemorrhage has resulted in an absence of urgency to treat these patients and a low threshold for the withdrawal of active care in these patients,^11^, ^12^ which contrasts sharply with modern systems of care for patients with acute ischaemic stroke.

Efforts to improve the success of randomised controlled trials in identifying an effective treatment for intracerebral haemorrhage have included the use of clinical and imaging variables to define a potential responder group with a high likelihood of early neurological deterioration from ongoing haemorrhage or haematoma growth.^13^, ^14^, ^15^ Another effort has been to extend the assessment of functional outcome beyond the conventional 90 days, because recovery from intracerebral haemorrhage takes longer than from acute ischaemic stroke.^16^ Assessing combinations of interventions as part of a multifaceted care bundle might also offer advantages, as shown in an Australian cluster clinical trial where the implementation of a treatment protocol for hyperglycaemia, fever, and dysphagia improved outcome from acute stroke.^17^ Further support for this approach was provided by a single-site, so-called before-and-after study in the UK, where the implementation of a quality improvement protocol that included the reversal of anticoagulation, intensive blood pressure lowering, and rapid triage to neurosurgery and critical care was associated with a lower 30-day case fatality after intracerebral haemorrhage.^18^ A post-hoc analysis of the second phase of the international Intensive Blood Pressure Reduction in Acute Cerebral Haemorrhage Trial (INTERACT2)^19^ showed that higher scores assigned for any elevation in the baseline of systolic blood pressure, glucose, body temperature, and previous use of anticoagulants in participants independently predicted a poor functional outcome after intracerebral haemorrhage. These issues informed the design of the third Intensive Care Bundle with Blood Pressure Reduction in Acute Cerebral Haemorrhage Trial (INTERACT3), with the aim of establishing whether a goal-directed care bundle protocol, comprising early intensive lowering of blood pressure with other management protocols for abnormal physiological variables, improves functional outcome in a broad range of patients with acute spontaneous intracerebral haemorrhage.

**Research in context**

**Evidence before this study**

We searched PubMed (from Jan 1, 1970, to Oct 30, 2022) and Embase (from Jan 1, 1947, to Oct 30, 2022), with no language or data restrictions, on Nov 24, 2022, for publications with relevant text words in the title or abstract or keywords that included: “intracerebral haemorrhage” OR “haemorrhagic stroke”, “care bundle” OR “treatment combination”, “blood pressure” OR “blood pressure lowering”, “blood glucose” OR “hyperglycaemia”, “body temperature”, and “anticoagulation reversal”. Studies were eligible for inclusion if they assessed the effectiveness of a combination of treatments on clinical outcomes. We identified only one completed cluster randomised controlled trial, done in 2011, the Quality in Acute Stroke Care trial, which showed that a combined protocol to manage fever, hyperglycaemia, and swallowing dysfunction improved the functional outcome in patients with acute stroke (both intracerebral haemorrhage and ischaemic stroke). A 2019 study in Salford, UK, showed a significant association between the implementation of a care bundle of anticoagulation reversal, blood pressure control, and rapid access to neurosurgery and improved survival after cerebral haemorrhage. The 2011 trial did not include blood pressure lowering in the protocol and there were few cases of intracerebral haemorrhage, whereas the 2019 study was limited by its before-and-after design. No ongoing trials were identified through a search of registered trials at ClinicalTrials.gov. There was no strong evidence to recommend the implementation of an intensive care bundle in acute intracerebral haemorrhage.

**Added value of this study**

The third Intensive Care Bundle with Blood Pressure Reduction in Acute Cerebral Haemorrhage Trial is the only randomised controlled trial involving a care bundle that included intensive blood pressure lowering treatment in acute intracerebral haemorrhage. The primary result was that the implementation of the care bundle across participating hospitals resulted in patients having a better functional outcome (measured on the modified Rankin Scale) at 6 months post-treatment compared with usual care. This result included favourable effects on survival and health-related quality of life. The results could not be explained by temporal trends in the characteristics of the patients or their management.

**Implications of all the available evidence**

A simple, time-sensitive, goal-directed, care bundle protocol, with a foundation strategy of early intensive blood pressure management to a systolic blood pressure target of less than 140 mm Hg, was safe and effective in improving the functional outcome of acute intracerebral haemorrhage. These results provide a clear implication for the rapid control of blood pressure and other physiological variables to be incorporated into clinical practice as a part of active management for this serious disease.

## Methods

### Study design

INTERACT3 was an international, multicentre, prospective, stepped wedge, cluster randomised, blinded, outcome assessed, controlled trial undertaken at hospitals located in nine LMICs (Brazil, China, India, Mexico, Nigeria, Pakistan, Peru, Sri Lanka, and Viet Nam), and one high-income country (Chile). The trial had a hybrid discovery–implementation design, in which clusters of patients were prospectively followed up to establish their outcome as their hospitals were randomly allocated to switch from control usual care to the intervention implementation at different timepoints. The experimental approach was done to mirror the natural process of rolling out a new quality improvement policy, that of implementing a goal-directed intensive care bundle protocol involving the rapid correction of any abnormal physiological variables (hypertension, hyperglycaemia, and pyrexia) and altered coagulation profile in patients with acute intracerebral haemorrhage. The trial followed the Consolidated Standards of Reporting Trials Extension reporting guideline for stepped wedge cluster randomised trials.^20^ Details of the protocol and statistical analysis plan have been published elsewhere^21^, ^22^ and are available in the appendix (pp 89–221).

The study was approved by the ethics committees at participating hospitals and appropriate regulatory authorities. A mixed consent process was applied, whereby a cluster guardian (an appropriate senior delegate [eg, chief executive officer]) provided consent for patients with acute intracerebral haemorrhage to receive the intervention as part of routine care. Participants (or an approved surrogate) provided written informed consent for the collection of sociodemographic, medical, and clinical information, and for them to be contacted to assess their outcome at 6 months. Two amendments were made to the original protocol dated July 12, 2017, to document additional sources of funding, extend the time for each intervention period to 4 months for sites in Asia to maximise the recruitment of patients, allow a transition time of 7–10 days before the sites entered the implementation period, for stratification variables to be incorporated into the randomisation programme, and to extend the study timelines (April 8, 2018); and additionally to expand the number of hospitals, further extend the study timelines, embed a process evaluation and economic evaluation within the trial, clarify the inclusion criteria for the diagnosis of intracerebral haemorrhage, include a form to document consent in patients who withdrew from the study, and clarify the patient recruitment targets within the study periods for the required sample size (Aug 12, 2019).

### Clusters and participants

Hospital sites were approached through neurosurgery and neurology professional networks. To be eligible, they either had no or inconsistent protocols for managing abnormal physiological variables in patients with acute intracerebral haemorrhage and had to be willing to implement the required interventional care bundle protocol as part of routine care. Eligible sites needed to enrol consecutive adult (age ≥18 years) patients presenting within 6 h after the onset of acute intracerebral haemorrhage, have a local representative (known as a champion) who was willing to lead the implementation of the intervention, and be able to provide the required study data. Key patient exclusion criteria included the intracerebral haemorrhage being secondary to a structural abnormality in the brain (eg, an arteriovenous malformation, intracranial aneurysm, tumour, trauma, or previous cerebral infarction) or to reperfusion therapy (eg, intravenous thrombolysis or endovascular thrombectomy), or that a patient was unlikely to adhere to the study treatment or follow-up regimen, or both, as judged by the treating clinician. Full details of the inclusion and exclusion criteria are provided in the appendix (p 14).

### Randomisation and masking

Eligible hospital sites were randomly assigned into three sequences (with four periods) by use of a computer-generated list by the trial statistician (QL) using permuted blocks, stratified by country and the projected number of patients to be recruited per site during the study period (<80, 80–160, and >160 patients). In period 1, all sites kept to their usual care and monitoring procedures for patients with intracerebral haemorrhage. Sites assigned to sequence 1 commenced the care bundle in period 2, those in sequence 2 received the intervention at period 3, and those in sequence 3 received the intervention at period 4, as outlined in the appendix (pp 26–30, 78). The criteria that triggered sites to progress to the next period were either achieving the predetermined patient enrolment target or reaching a time of 3 months (4 months in Asia where there was greater capacity for patient recruitment) from the start date for each period. To avoid contamination, details of the intervention, sequence, and allocation periods were concealed from sites until they had completed the usual care control periods. A period of 7–10 days was used to allow sites time for their staff to receive training before transitioning from usual care to the intervention period. Trained research staff masked to group allocation and patient details located in country-based central offices (in China, Chile, Brazil, and Nigeria) or at hospitals (in the other countries), undertook telephone assessments of the outcome of patients at 6 months post-randomisation.

For logistical reasons and according to available funding, sites were activated in batches beginning in China in 2017, and subsequently in the other countries in 2019 (appendix pp 26–32, 79–80). However, because of the COVID-19 pandemic, patient recruitment was suspended in Chile, Pakistan, and Peru between March 1 and Aug 31, 2020, and no new hospitals outside of China commenced the recruitment of patients until Nov 2, 2020. Because the pandemic affected patient recruitment, mainly at sites in the intervention phase, and caused an imbalance in patient numbers between the randomly assigned groups, the duration of this phase was extended to allow as many sites as possible to participate and enrol patients into the intervention group.

### Procedures

Before hospitals began screening and recruiting patients, key staff from participating sites were trained in the study procedures at regional and individual hospital meetings. In the 7–10 days after sites had completed the control usual care period, these staff received online training and remote communication on the intensive care bundle protocol to be applied as a system of care for eligible patients. The components of the care bundle included: early intensive blood pressure management with the goal of achieving a target systolic blood pressure of less than 140 mm Hg within 1 h of the initiation of treatment, with a systolic blood pressure of 130 mm Hg being the threshold for the cessation of treatment; intensive control of elevated blood glucose with the goal of achieving a glucose target of 6·1–7·8 mmol/L for patients without diabetes and 7·8–10·0 mmol/L for patients with diabetes as soon as possible after the initiation of treatment, as recommended in guidelines for minimal risk of harm from hypoglycaemia;^23^ treatment of pyrexia with the goal of achieving a body temperature of less than 37·5°C within 1 h of initiation; and the reversal of abnormal anticoagulation in those taking warfarin using fresh frozen plasma or prothrombin concentrate complex with the goal of reaching an international normalised ratio of less than 1·5 within 1 h of treatment. All target concentrations within the care bundle were to be maintained in patients for 7 days (or until discharge or death, should these events occur earlier). Site-specific data uploaded to the study database on blood pressure, glucose, temperature, and international normalised ratio served as a method to check adherence to the protocol, and also allowed feedback and further training to be offered to sites by trial staff. All sites received monthly performance reports and were required to have staff attend at least two online quality improvement meetings held repeatedly during the study. A process evaluation, whereby formative stakeholder engagement interviews were conducted during the trial, allowed insight to be gained into the barriers and facilitators to change systems of care and to implement the protocol.^24^ The information also served as a further guide to the training of site staff in their integration of the care bundle into practice. Further details of the care bundle protocol are provided in the appendix (pp 88–170).

To ensure continuous recruitment, each participating site was required to register all patients with a diagnosis of intracerebral haemorrhage into screening and enrolment logs. For enrolled patients, demographic and clinical data, including the amount of neurological impairment measured using the National Institutes of Health Stroke Scale (NIHSS; range 0–42, with higher scores indicating greater severity) were collected at the time of admission to hospital. Follow-up data were collected on clinical status, management, and outcomes at days 1 and 7 of treatment (or discharge or death if earlier) by hospital staff, and of outcomes to be ascertained at 6 months by independent staff. Brain imaging undertaken at presentation (the diagnostic scan), and at 24 h and 7 days of treatment in a subsample of 1619 patients (first seven patients in each of the usual care and intervention phases at each site) were uploaded to a secure central imaging server for analysis by trained physicians who were masked to treatment allocation. Results of the centrally adjudicated brain imaging will be presented in future publications.

### Outcomes

The primary outcome was functional recovery measured at 6 months according to the modified Rankin scale (mRS) and analysed as an ordinal outcome (shift across all categories). The mRS is a standard global measure of disability scored from 0 to 6, in which scores of 0–1 indicate a favourable outcome without or with symptoms but no disability; scores of 2–5 indicate increasing amounts of disability (and dependency); and a score of 6 indicates death. Secondary outcomes included the dichotomous analysis of scores on the mRS at 6 months: 3–6 (disability or death) versus 0–2, and 3–5 (major disability) versus 0–2 in survivors; death at 6 months; death or neurological deterioration at 7 days according to a change in scores on the NIHSS, both as a continuous measure and categorised into seven groups (<5, 5–9, 10–14, 15–19, 20–24, ≥25, and death);^25^ health-related quality of life measured using the EuroQoL Group 5-Dimension Self-Report Questionnaire (EQ-5D-3L); and residence (own home *vs* other) at 6 months. Time to discharge from hospital was also planned but was only recorded at day 7 to coincide with the other assessments undertaken at this time, and was therefore censored at 7 days. The safety outcomes of all-cause and cause-specific serious adverse events were recorded according to standard definitions for the duration of follow-up.

### Statistical analysis

This study was designed with 90% power (α=0·05) to detect a common odds ratio (OR) of 0·8 for worse functional outcome at 6 months using ordinal logistic regression. This design required a sample size of 8360 patients from 110 sites, on the assumption that the distribution in the scores on the mRS in the usual care group would be similar to the control group who received standard less-intensive blood pressure lowering treatment in the INTERACT2 study,^26^ and with 5% of patients lost to follow-up. This treatment effect corresponded to a 5·6% absolute decrease in worse functional outcome (mRS scores 3–6) from receiving the care bundle, from 55·6% down to 50·0% compared with usual care. We assumed an intraclass correlation coefficient of 0·04 on the basis of the results of INTERACT2 and another international cluster randomised trial in acute stroke.^25^ The required sample size, which corresponded to an average of 19 patients being recruited per phase at each participating hospital (ie, 110 sites × 4 phases × 19 patients), was calculated using PASS software 2019, by tests for two ordered categorical variables,^27^ and by adjusting for the clusters corresponding to the stepped wedge design.^28^

All analyses were undertaken at the patient level with adjustment for clustering. The main analyses were performed on a modified intention-to-treat basis by including all participants who provided written consent and had primary outcome data available, regardless of protocol adherence. The primary analysis was to be done using ordinal logistic regression with a random effect for cluster (hospital site), a fixed categorical effect of time (four periods), and a fixed effect indicating the group assignment of each cluster at each period. However, upon unmasking the data, we recognised that the trial did not conform to a standard stepped wedge design in which all clusters contemporaneously switch from one period to the next,^29^ because the period lengths were not equal and the times at which the different clusters crossed from control to intervention conditions varied widely (appendix pp 26–30, 78–79). To more appropriately account for the effect of time, we adjusted for calendar time instead of trial period by using 6-month calendar periods that corresponded to the length of follow-up for the assessment of the clinical outcomes in individual patients from the time the first patient was recruited (Dec 12, 2017) to the time the last patient was recruited (Dec 31, 2021; appendix p 32).^30^, ^31^

We undertook a series of preplanned sensitivity analyses with various approaches to the imputation of missing values,^22^ variable time effects across sites,^32^ and additional adjustment for the covariates of country (grouped regionally as China *vs* India, Pakistan, Sri Lanka, and Viet Nam *vs* Brazil, Peru, Chile, Mexico, and Nigeria), pre-stroke function (estimated categorical mRS scores of 0 to 5), age, sex, and baseline NIHSS score to the primary model. The NIHSS score was also analysed as a continuous variable using an unadjusted hierarchal linear regression model assuming a normal distribution and an identity link function. We also conducted post-hoc sensitivity analyses by modelling the effect of time continuously using restricted cubic splines based on 1-month and 3-month calendar time windows.

We found the proportional-odds assumption was violated but the analyses proceeded on the understanding that the treatment effect was not constant across categories of the mRS. The result was complemented by a graphical assessment of shifts across categories using bar plots as well as binary analyses. As prespecified, the six subgroups for analysis were age, sex, country, NIHSS score (<15 *vs* ≥15), and clinician-reported volume (<15 mL *vs* 15 to <30 mL *vs* ≥30 mL) and location (cortical *vs* deep *vs* brainstem, cerebellar, or primary ventricular) of the haematoma reported by investigators at baseline, with tests of interaction applied between the specific subgroup and the treatment effect on the primary outcome. A post-hoc estimation of the number needed to treat for a benefit from the treatment was made to help in the interpretation of the results.

Although two interim analyses were undertaken during the study period, given the use of a conservative Haybittle–Peto stopping rule^33^ and the negligible amount of type 1 error rate spent, the significance threshold for the primary outcome, including sensitivity analyses, was still p<0·05. For the seven secondary clinical outcomes, we controlled the family-wise error rate by applying a sequential Holm–Sidak correction^34^ to facilitate an interpretation of the findings.^35^ Between-group differences in interventional physiological variables were assessed using a repeated-measure linear mixed model with adjustments for treatment, time, and treatment-by-time interactions fixed effects, site as a random effect, and with within-patient correlations modelled using a repeated patient effect assuming a compound-symmetry structure. We used SAS Enterprise Guide (version 8.2) and R (version 4.0.0 or newer) for statistical analysis. This trial is registered with ClinicalTrials.gov (NCT03209258) and the Chinese Clinical Trial Registry (ChiCTR-IOC-17011787).

### Role of the funding source

The funders of the study had no role in study design, data collection, data analysis, data interpretation, or writing of the report.

## Results

**Figure 1:**
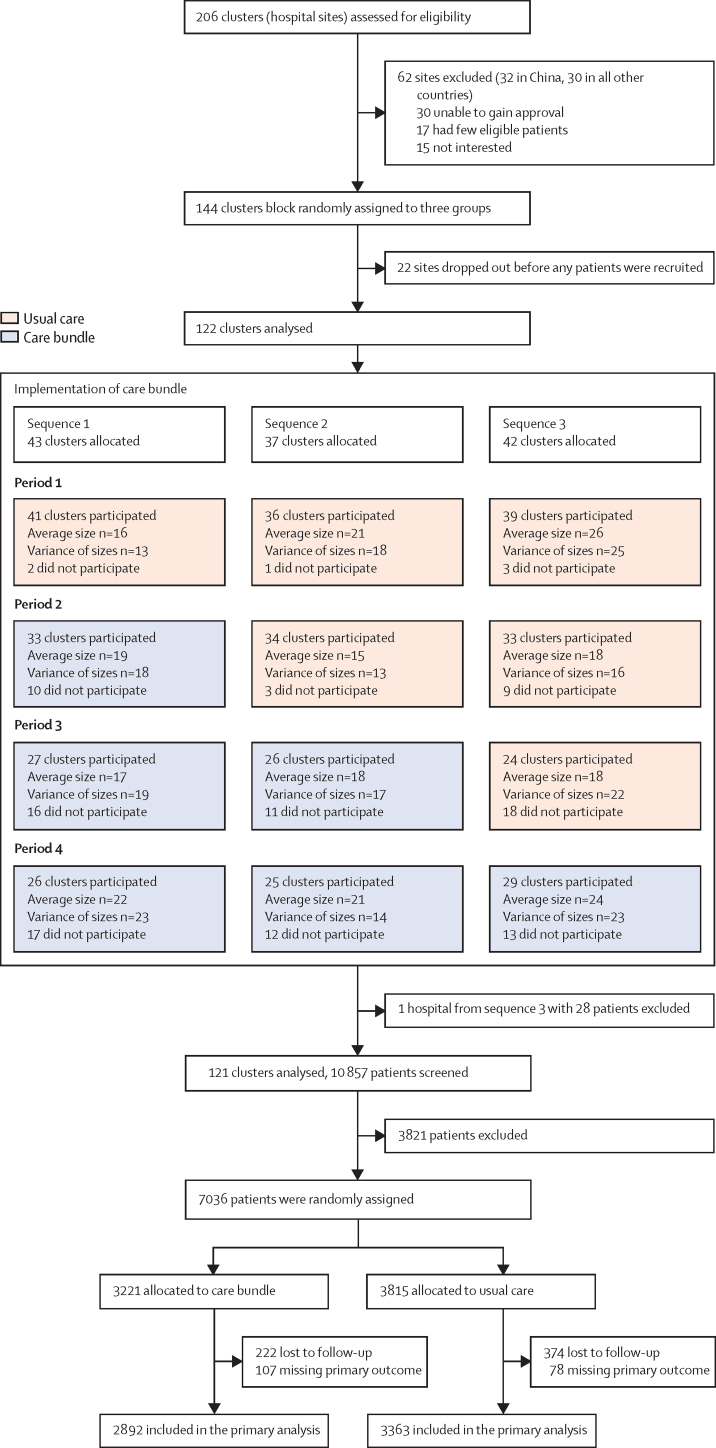
Flow diagram of participating hospitals and patients

Between May 27, 2017, and July 8, 2021, 206 hospitals were assessed for eligibility, of which 144 hospitals in ten countries agreed to join and were randomly assigned in the trial, but 22 hospitals subsequently withdrew before any patients were enrolled. Of the remaining 122 hospitals, 43 hospitals were randomly assigned into sequence 1, 37 hospitals into sequence 2, and 42 hospitals into sequence 3. For logistical reasons, sites were randomly assigned in four batches. The first three batches (30 sites in each) were for sites in China, with each lasting 3–4 months; batch 1 from Nov 17, 2017, to Sept 16, 2019; batch 2 from April 20, 2018, to June 25, 2019; and batch 3 from Aug 29, 2018, to Nov 2, 2019. Random assignment of the fourth batch, which included six sites in China from April 16 to Nov 25, 2020, and all sites outside of China, occurred between March 20, 2019 and March 31, 2021. One hospital allocated to sequence 3 was later withdrawn and their data deleted after the implementation of the intervention because a necessary regulatory approval was not obtained. The continued participation of the hospitals in the trial tended to decline across the phases because of the effect of COVID-19 (32 sites), unavailability of eligible patients (17 sites), loss of interest after the champion left the hospital (11 sites), regulatory changes (two sites), and early achievement of the recruitment target (two sites; appendix pp 26–30). Even so, the number of patients enrolled was balanced across the randomised sequences and periods (figure 1; appendix p 31). Between Dec 12, 2017, and Dec 31, 2021, 10 857 patients with acute intracerebral haemorrhage were screened for eligibility, with 3821 considered ineligible most often due to having a presentation longer than 6 h after the onset of symptoms. 7036 patients were randomly assigned in the trial, of whom 3815 were allocated to the usual care group and 3221 were allocated to the care bundle intervention group. The stepped wedge study timeline, number of patients recruited across sequences and periods, and reasons for excluding patients are outlined in the appendix (p 33). 374 (9·8%) patients in the usual care group and 222 (6·9%) patients in the care bundle group were lost to follow-up, and another 78 (2·0%) patients in the usual care group and 107 (3·3%) patients in the care bundle group had a missing functional status on the mRS obtained at the final visit. Thus, 3363 patients in the usual care group and 2892 patients in the care bundle group had data available on the primary outcome and were included in the primary efficacy analysis (figure 1). The proportions of participants with primary outcome data and the method of assessment were well balanced by group and period (appendix pp 31, 34–35).

Baseline demographic, medical history, and the clinical and brain imaging characteristics of patients were well balanced between the treatment groups (table 1; appendix p 36). The mean age was 62·0 (SD 12·6) years, 2533 (36·0%) of 7036 patients were female, and 6350 (90·3%) were Chinese. Median scores were 12 (IQR 9–14) on the Glasgow coma scale and 13 (IQR 7–22) on the NIHSS. The cause of the intracerebral haemorrhage was presumed to be related to hypertension in 6574 (94·3%) of 6972 patients and located deep in a cerebral hemisphere in 5638 (82·3%) of 6849 patients (appendix p 37). The median volume of haemorrhage was recorded as 15·0 mL (IQR 7·8–30·0) in 6652 patients and intraventricular haemorrhage was present in 2093 (29·8%) of 7032 patients. 5160 (73·3%) of 7035 patients were admitted directly to a neurosurgery department, and 6320 (90·4%) of 6988 patients had a blood pressure of 140 mm Hg or greater, with an overall mean systolic blood pressure of 174·5 mm Hg (SD 28·3). 2413 (36·0%) of 6711 patients had elevated blood glucose and the overall mean blood glucose concentration was 8·0 mmol/L (SD 2·9). Only 120 (1·7%) of 6930 patients had an elevated body temperature and 84 (1·2%) of 6760 patients had a higher international normalised ratio at presentation.

**Table 1:** Patient characteristics at baseline

			**Care bundle (n=3221)**	**Usual care (n=3815)**
Age, years			61·8 (12·6)	62·1 (12·6)
Sex				
	Female		1146 (35·6%)	1387 (36·4%)
	Male		2075 (64·4%)	2428 (63·6%)
Ethnicity				
	Han Chinese		2807 (87·1%)	3490/3814 (91·5%)
	Non-Han Chinese		19 (0·6%)	34/3814 (0·9%)
	Caucasian or European		2 (0·1%)	3/3814 (0·1%)
	Latin American		45 (1·4%)	45/3814 (1·2%)
	Mixed		4 (0·1%)	2/3814 (0·1%)
	Other Asian		92 (2·9%)	76/3814 (2·0%)
	Indian subcontinent		194 (6·0%)	143/3814 (3·7%)
	Other		58 (1·8%)	21/3814 (0·6%)
Main occupation*				
	Unskilled		2855/3218 (88·7%)	3289/3809 (86·3%)
	Skilled		363/3218 (11·3%)	520/3809 (13·7%)
BMI, kg/m^2^†				
	Number for which this information was collected		2713	3172
	Mean		24·1 (3·7)	24·1 (3·6)
Medical history				
	History of hypertension		2207 (68·5%)	2681/3814 (70·3%)
	Previous intracerebral haemorrhage		247 (7·7%)	294/3814 (7·7%)
	Previous ischaemic stroke		246 (7·6%)	319/3814 (8·4%)
	History of coronary artery disease		70 (2·2%)	123/3814 (3·2%)
	History of other heart disease		142 (4·4%)	155/3814 (4·1%)
	History of atrial fibrillation		31 (1·0%)	51/3814 (1·3%)
	History of diabetes		338 (10·5%)	391/3814 (10·3%)
	History of hypercholesterolaemia		90 (2·8%)	117/3813 (3·1%)
	Current smoker		588 (18·3%)	771/3813 (20·2%)
	Current alcohol consumption		622 (19·3%)	772/3813 (20·2%)
	Modified Rankin scale score before onset‡			
		0	2489 (77·3%)	2894/3748 (77·2%)
		1	358 (11·1%)	440/3748 (11·7%)
		2	109 (3·4%)	149/3748 (4·0%)
		3	89 (2·8%)	77/3748 (2·1%)
		4	102 (3·2%)	108/3748 (2·9%)
		5	74 (2·3%)	80/3748 (2·1%)
Medications				
	Antihypertensive medication		1446 (44·9%)	1626/3813 (42·6%)
	Blood glucose lowering agents		250 (7·8%)	263/3814 (6·9%)
	Statin or other lipid lowering agent		89 (2·8%)	133/3814 (3·5%)
	Aspirin or other antiplatelet agent		151 (4·7%)	227/3814 (6·0%)
	Anticoagulation agent		28 (0·9%)	37/3814 (1·0%)
Systolic blood pressure, mm Hg				
	Number for which this information was collected		3221	3813
	Mean		174·6 (28·2)	174·5 (28·4)
Diastolic blood pressure, mm Hg				
	Number for which this information was collected		3221	3813
	Mean		99·7 (17·7)	99·0 (17·9)
Severity of neurological deficit by scores on the NIHSS§				
	Number for which this information was collected		3149	3693
	Median (IQR)		13 (7–23)	13 (6–22)
Consciousness by scores on the GCS¶				
	Number for which this information was collected		3219	3810
	Median (IQR)		12 (9–14)	12 (9–14)
Brain imaging features‖				
	Haematoma present on CT scan		3175 (98·6%)	3680/3814 (96·5%)
	Volume of haematoma, mL			
		Number for which this information was collected	3129	3523
		Median (IQR)	15 (7–30)	15 (8–30)
	Side of haematoma			
		Left	1628/3175 (51·3%)	1775/3676 (48·3%)
		Right	1410/3175 (44·4%)	1751/3676 (47·6%)
		Midline	176/3175 (5·5%)	217/3676 (5·9%)
	Location of the haematoma			
		Cortical	299/3175 (9·4%)	329/3674 (9·0%)
		Deep	2631/3175 (82·9%)	3007/3674 (81·8%)
		Cerebellum	157/3175 (4·9%)	212/3674 (5·8%)
		Brainstem	156/3175 (4·9%)	195/3674 (5·3%)
	Intraventricular haemorrhage		863 (26·8%)	1230/3811 (32·3%)
Presumed cause‖				
	Hypertension vasculopathy		3043/3197 (95·2%)	3531/3775 (93·5%)
	Cerebral amyloid angiopathy		167/3197 (5·2%)	178/3775 (4·7%)
Abnormal physiological variables				
	Systolic blood pressure ≥140 mm Hg		2905 (90·2%)	3415/3767 (90·7%)
	Blood glucose >7·8 mmol/L in those without diabetes and >10·0 mmol/L in those with diabetes		1094/3175 (34·5%)	1319/3536 (37·3%)
	Body temperature >37·5°C		52/3214 (1·6%)	68/3716 (1·8%)
	International normalised ratio ≥1·5		25/3113 (0·8%)	59/3647 (1·6%)

Data are shown as mean (SD), n (%), median (IQR), or n/N (%). GCS=Glasgow coma scale. NIHSS=National Institutes of Health Stroke Scale.

* Skilled includes professional or executive, business, sales, and service jobs; unskilled includes driver, farmer or labourer, home duties, and other jobs.

† Derived from self-reported height and weight.

‡ Scores on the modified Rankin scale of functional recovery range from 0 (no symptoms) to 6 (death); a score of 2 or less indicates functional independence. The modified Rankin scale score before stroke onset was assessed by the treating physician with the use of information obtained from patients (if possible) or their family members.

§ Scores on the NIHSS range from 0 to 42, with higher scores indicating more severe neurological deficits.

¶ Scores on the GCS range from 15 (normal) to 3 (deep coma).

‖ Reported by clinician investigators; multiple options recorded.

**Figure 2:**
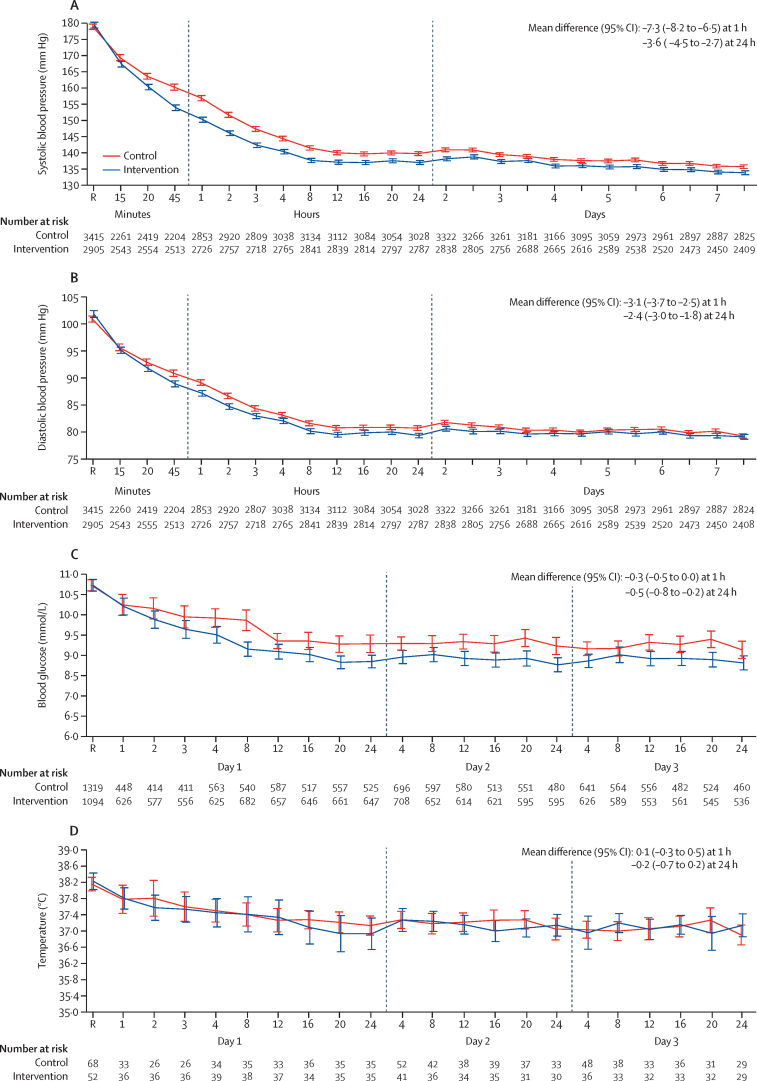
Physiological measures from random assignment after treatment to day 7 Values are shown for patients with abnormal systolic blood pressure (A) and abnormal diastolic blood pressure (B) in 2905 (90·2%) of 3221 patients in the care bundle group and 3415 (90·7%) of 3767 patients in the usual care group; abnormal glucose (C) in 1094 (34·5%) of 3175 patients in the care bundle and 1319 (37·3%) of 3536 patients in the usual care group; and abnormal body temperature (D) in 52 (1·6%) of 3214 patients in the care bundle and 68 (1·8%) of 3716 patients in the usual care group. Recordings were at 15-min intervals in the first hour after commencing treatment (time 0), hourly from hours 1 to 6, once every 6 h until 24 h, and twice per day until day 7. The overall means per treatment group and overall difference in variables between treatment groups at 1 h and at 24 h were calculated using a repeated-measure linear mixed model with a fixed effect of treatment, a fixed categorical effect of time, a fixed interaction between treatment and time, a repeated patient effect (to model within-patient correlations assuming a compound symmetry structure), and a random site effect, with adjustment for baseline measurements. R indicates time of random assignment.

The proportion of patients administered any intravenous blood pressure lowering drug in the first 24 h was higher in the care bundle group (2542 of 3221 [78·9%]) than the usual care group (2703 of 3811 [70·9%]); results were similar during days 2–7 (2131 of 3188 in the care bundle group [66·8%] *vs* 2277 of 3775 in the usual care group [60·3%]; table 2; appendix pp 38–39). The most common intravenous agents used in the first 24 h were urapidil (3351 [61·2%]), sodium nitroprusside (1169 [21·4%]), labetalol (663 [12·1%]), nicardipine (438 [8·0%]), and nimodipine (432 [7·9%]) reported in 5473 patients who received blood pressure lowering treatment (appendix p 38). Mean systolic blood pressures were 148·4 mm Hg (SD 21·5) at 1 h and 136·1 mm Hg (16·5) at 24 h in the care bundle group, and 154·7 mm Hg (22·5) at 1 h and 139·0 mm Hg (17·2) at 24 h in the usual care group (adjusted mean difference at 24 h of –3·6 mm Hg; 95% CI –4·5 to –2·7; p<0·0001; figure 2A). The systolic blood pressure target of 140 mm Hg or less was achieved more quickly in the care bundle group than in the usual care group (median, 2·3 h [IQR 0·8–8·0] *vs* 4·0 h [1·9–16·0]; table 2). The mean diastolic blood pressures were 86·2 mm Hg (SD 13·8) at 1 h and 78·9 mm Hg (11·5) at 24 h in the care bundle group, and 88·3 mm Hg (14·2) at 1 h and 80·4 mm Hg (12·1) at 24 h in the usual care group (adjusted mean difference at 24 h of –2·4 mm Hg; 95% CI –3·0 to –1·8; p<0·0001; figure 2B). In the care bundle group, the proportion of patients who achieved the blood glucose target control was higher than in the usual care group (147 of 1094 [13·4%] *vs* 86 of 1319 [6·5%]), although there were minimal differences in adjusted mean glucose concentrations over 24 h (–0·5 mmol/L; 95% CI –0·8 to –0·2; figure 2C) and in the time to achieving the glycaemic control target (median, 1·0 h [IQR 1·0–1·0] *vs* 1·0 h [1·0–1·0]). Patients in the care bundle group with pyrexia also had more frequent control of their body temperature than those in the usual care group (45 of 52 [86·5%]) at a median of 3·0 h [IQR 1·3–9·5] *vs* 57 of 68 [83·8%] at a median of 4·0 h [2·0–12·3]), but there was no overall adjusted mean difference over 24 h between the two groups (figure 2D). There were no clear differences in the reversal of anticoagulation (12 of 25 [48·0%] at a median of 27 h [IQR 24·0–48·0] in the care bundle group and 24 of 59 [40·7%] at a median of 25·5 h [IQR 24·0–48·0] in the usual care group). Table 3 (and appendix p 40) shows there were no major differences in other management methods over 7 days, especially in the use of decompressive surgery, endotracheal intubation, and early withdrawal of care. The distribution of the reported volumes of the haematoma and NIHSS scores over time are shown in the appendix (pp 80–81).

**Table 2:** Management of patients 7 days after treatment

			**Care bundle (n=3221)**	**Usual care (n=3815)**
**Location of patients after admission to hospital**				
Neurosurgery ward			2222 (69·0%)	2938/3814 (77·0%)
Intensive care unit			463 (14·4%)	351/3814 (9·2%)
Neurology ward			255 (7·9%)	260/3814 (6·8%)
Emergency department			149 (4·6%)	163/3814 (4·3%)
Other area			132 (4·1%)	103/3814 (2·7%)
**Treatment in the first 24 h**				
Intravenous blood pressure lowering treatment			2542 (78·9%)	2703/3811 (70·9%)
	Systolic blood pressure target <140 mm Hg reached*		2809/2905 (96·7%)	3223/3415 (94·4%)
	Median time to reaching target (IQR)		2·3 (0·8 to 8·0)	4·0 (1·9 to 16·0)
Intensive treatment of blood glucose			250 (7·8%)	263/3814 (6·9%)
	Type of agent used for glycaemic control			
		Oral agents	205/250 (82·0%)	211/263 (80·2%)
		Insulin	56/250 (22·4%)	72/263 (27·4%)
	Blood glucose target of 6·1–7·8 mmol/L in patients without diabetes and 7·8–10·0 mmol/L in patients with diabetes reached*		147/1094 (13·4%)	86/1319 (6·5%)
		Median time to reaching target (IQR), h	1·0 (1·0 to 1·0)	1·0 (1·0 to 1·0)
Antipyrexia treatment			314/3219 (9·8%)	280/3809 (7·4%)
	Type of antipyrexia treatment			
		Paracetamol	39/314 (12·4%)	43/280 (15·4%)/
		Metamizole	7/314 (2·2%)	12/280 (4·3%)
		Hypothermia cooling with calf packing	228/314 (72·6%)	166/280 (59·3%)
		Intravenous infusion with cooled (4°C) saline	17/314 (5·4%)	35/280 (12·5%)
		Other	44/314 (14·0%)	60/280 (21·4%)
	Temperature target (≤37·5°C) reached*		45/52 (86·5%)	57/68 (83·8%)
		Time to reach target, h	3·0 (1·3 to 9·5)	4·0 (2·0 to 12·3)
Correction of abnormal coagulation				
	International normalised ratio target <1·5 reached*		12/25 (48·0%)	24/59 (40·7%)
		Time to achieving target, h	27·0 (24·0 to 48·0)	25·5 (24·0 to 48·0)
**Other management from day 2 to day 7**				
Intravenous blood pressure lowering treatment			2131/3188 (66·8%)	2277/3775 (60·3%)
Oral blood pressure lowering treatment			2267/3188 (71·1%)	2547/3775 (67·5%)
Insulin			499/3188 (15·7%)	440/3775 (11·7%)
Hypothermia treatment			669/3188 (21·0%)	751/3775 (19·9%)
Prothrombin complex concentrate administered			332/3188 (10·4%)	289/3775 (7·7%)
Fresh frozen plasma administered			61/3188 (1·9%)	104/3775 (2·8%)
Vitamin K administered			174/3188 (5·5%)	163/3775 (4·3%)
Decompressive surgery			844/3196 (26·4%)	1016/3775 (26·9%)
Mechanical ventilation			646/3196 (20·2%)	806/3775 (21·4%)
Intensive care admission			1105/3196 (34·6%)	1435/3775 (38·0%)
Assisted feeding			1749/3196 (54·7%)	1920/3775 (50·9%)
Decision to withdraw active care			21/3196 (0·7%)	26/3775 (0·7%)

Data are shown as mean (SD), n (%), median (IQR), or n/N (%).

* Reached care bundle targets during the 7 days of treatment.

**Table 3:** Clinical outcomes at 6 months

		**n**	Odds ratio or mean difference (95% CI)*	**p value**†	**Intraclass correlation coefficient**
**Primary outcome**					
Ordinal analysis of category scores measured by the mRS*					
	Unadjusted	6255	0·86 (0·76 to 0·97)†	0·015	0·05
	Adjusted	6069	0·84 (0·73 to 0·97)‡	0·017	0·07
**Secondary outcome**					
Ordinal analysis of category scores for neurological impairment or death measured by the NIHSS by day 7 of treatment		6809	0·89 (0·77 to 1·03)†§	0·12	0·23
Death or disability at 6 months (mRS scores 3–6)		6255	0·89 (0·78 to 1·02)†	0·10	0·04
Death at 6 months		6440	0·77 (0·63 to 0·95)†	0·015	0·07
Major disability in survivors at 6 months (mRS scores 3–5)		5277	0·96 (0·83 to 1·11)†	0·56	0·03
Health-related quality of life measured by the EQ-5D-3L¶					
	Mobility	5277	0·87 (0·75 to 1·01)†	0·064	0·02
	Self-care	5277	0·93 (0·82 to 1·07)†	0·32	0·02
	Usual activities	5277	0·97 (0·85 to 1·10)†	0·62	0·02
	Pain or discomfort	5277	0·78 (0·67 to 0·91)†	0·0016	0·02
	Anxiety or depression	5277	0·83 (0·69 to 1·00)†	0·046	0·04
	Visual analogue scale	5276	1·24 (–0·20 to 2·67)†	0·091	0·02
	Mean overall health utility score	6255	0·04 (0·02 to 0·07)†	0·0008	0·06
Hospital discharge by day 7 of treatment		7016	0·72 (0·53 to 0·98)†	0·034	0·33
Residence at home at 6 months		5277	0·94 (0·65 to 1·35)†	0·73	0·03

EQ-5D-3L=EuroQoL Group 5-Dimension Self-Report Questionnaire. mRS=modified Rankin scale. NIHSS=National Institutes of Health Stroke Scale.

* The mRS evaluates global disability; scores range from 0 (no symptoms) to 6 (death). A score of 2–5 indicates some degree of disability. The common odds was estimated from an ordinal logistic regression model and indicates the odds of worse functional outcome for the care bundle group compared with the usual care group.

† Estimates from a logistic or linear regression model with a random effect of cluster (hospital site) and group assignment (care bundle or usual care) as a fixed effect, and calendar time (6-month window) as a fixed categorical effect.

‡ Adjusted for country (grouped as China *vs* India, Pakistan, Sri Lanka, and Viet Nam *vs* Brazil, Peru, Chile, Mexico, and Nigeria), pre-stroke mRS score, age, sex, baseline NIHSS score, random effect for cluster (hospital site), and group assignment (care bundle or usual care) as a fixed effect, and calendar time (6-month window) as a fixed categorical effect.

§ The common odds were estimated from an ordinal logistic regression model and indicate the odds of worse neurological deterioration measured on the NIHSS (separated into seven categories: <5, 5–9, 10–14, 15–19, 20–24, and ≥25) or death for the care bundle group compared with the usual care group. Scores on the NIHSS range from 0 to 42, with higher scores indicating more severe neurological deficits.

¶ The EQ-5D-3L covers five domains of health-related quality of life: mobility, self-care, usual activities, pain or discomfort, and anxiety or depression. Each domain has three graded levels of response: no problems, moderate problems, or extreme problems. Scores are combined to provide an overall health utility score that was calculated with population normal values using China normative data for China and UK normative data for countries other than China.

**Figure 3:**
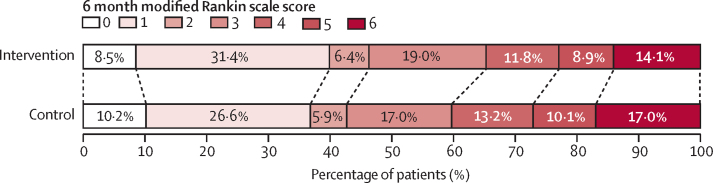
Functional outcome at 90 days in the care bundle and usual care groups, according to scores on the mRS Raw distribution of scores on the mRS at 90 days. Scores on the mRS range from 0 to 6, with 0 indicating no symptoms, 1 indicating symptoms without clinically significant disability, 2 indicating slight disability, 3 indicating moderate disability, 4 indicating moderately severe disability, 5 indicating severe disability, and 6 indicating death. There was a significant difference between the care bundle group and usual care group in the overall distribution of scores (common odds ratio, indicating a lower odds of worse global function outcome on the mRS, 0·86 [95% CI 0·76–0·97]; p=0·015). mRS=modified Rankin Scale.

Patients in the care bundle group had better scores on the mRS levels compared with those allocated to the usual care group, with a common OR of 0·86 (95% CI 0·76–0·97; p=0·015) for an average effect across all mRSs violated in the proportion odds assumption test (p=0·0003; figure 3; appendix pp 41–43). The favourable shifts in mRS scores in the care bundle group were still present when the prespecified methods of imputation were used to replace missing data, except for an extreme score of 6 (death; appendix pp 44–46). These shifts were also significant after further adjustment for country and patient characteristics (table 3) and when using varying time trends (appendix p 46). Furthermore, the results were consistent with post-hoc analyses with time modelled using restricted cubic splines variables (appendix p 52). When modelling time as originally planned using study period instead of calendar time, there was no clear difference between the randomised groups in either unadjusted or adjusted analyses, and with or without varying time trends (appendix pp 52–54).

Secondary outcome results were consistent with all point estimates favouring the care bundle group including significant differences in mortality, health-related quality of life, and time to discharge (table 3; appendix pp 46–48). Compared with the usual care group, the care bundle group had lower odds of death over 6 months in the primary model (common OR 0·77; 95% CI 0·63–0·95; p=0·015; appendix p 82). The difference became non-significant after adjustment for country and patient characteristics (0·84; 0·65–1·07; p=0·16). There was no significant difference in the odds of death or disability at 90 days (mRS scores 3–6, 54% *vs* 57%; 0·89; 0·78–1·02; p=0·10) or death or neurological deterioration at 7 days (0·89; 0·77–1·03; p=0·12). Some differences were observed in health-related quality of life between the randomised groups, including an increase in overall utility score in the care bundle group compared with the usual care group (mean difference 0·04, 95% CI 0·02–0·07; p=0·0008; table 3), but these differences diminished after further adjustment. The proportion of patients discharged from hospital by day 7 was significantly less in the care bundle group (OR 0·72, 95% CI 0·53–0·98; p=0·034) but there was no difference in the place of residence at 6 months. After controlling the family-wise error for multiple testing, only the EQ-5D-3L utility score was significantly higher in the care bundle group of the seven secondary outcomes (appendix p 54).

There was significant heterogeneity in the treatment effect on the primary outcome in the subgroups based on region and COVID-19 period (appendix pp 55–61, 83), which were related variables because all non-China sites were activated after the COVID-19 pandemic. There was no heterogeneity by COVID-19 for sites in China (appendix p 84). Between-group separations in the target systolic blood pressure being reached were greater in patients recruited in the first grouped country region of India, Pakistan, Sri Lanka, and Viet Nam compared with those in China and the other grouped country region (Brazil, Peru, Chile, Mexico, and Nigeria; appendix pp 85–87). However, no major regional differences between the randomised groups were apparent between China and each of the grouped regions regarding other aspects of management including decompressive surgery (care bundle *vs* usual care, 29·5% *vs* 28·8% in China, 2·1% *vs* 2·3% in India, Pakistan, Sri Lanka, and Viet Nam, and 7·2% *vs* 7·6% in Brazil, Peru, Chile, Mexico, and Nigeria) and intensive care (10·9% *vs* 8·4% in China, 9·2% *vs* 6·6% in India, Pakistan, Sri Lanka, and Viet Nam, and 9·3% *vs* 15·2% in Brazil, Peru, Chile, Mexico, and Nigeria; appendix pp 62–64).

Overall, patients in the care bundle group had significantly fewer serious adverse events than the usual care group (516/3221 [16·0%] *vs* 767/3815 [20·1%]; p=0·0098). A complete list of serious adverse events and causes of deaths is provided in the appendix (pp 65–72), as well as details regarding the causes of death (appendix pp 73–76). From the primary model, adjusted frequencies indicated 54·1% (crude number, 1553/2892 [53·7%]) of patients in the care bundle group and 56·9% (1927/3363 [57·3%]) in the usual care group were estimated with death or major disability, and 11·4% (407/2999 [13·6%]) in the care bundle group and 14·3% (571/3441 [16·6%]) in the usual care group were estimated with death alone, therefore the estimated number needed to treat for the care bundle to prevent one patient was 35 (95% CI 15 to infinity) to prevent one patient from death or major disability and 35 (17 to infinity) to prevent one patient from death.

## Discussion

This pragmatic stepped wedge, cluster randomised, controlled trial conducted in various health-care settings has shown that, in patients presenting within 6 h of the onset of acute intracerebral haemorrhage, the use of a care bundle protocol incorporating the early control of elevated blood pressure together with management algorithms for hyperglycaemia, pyrexia, and abnormal coagulation resulted in an improved functional outcome at 6 months. The positive result was consistent across a range of sensitivity analyses and secondary outcomes. Compared with usual care, implementation of the time-sensitive care bundle was also associated with improved survival, health-related quality of life, and serious adverse events during the follow-up of patients. Given that 118 of the participating hospitals admitted more than 100 patients with acute intracerebral haemorrhage per year, the size of the beneficial effect equates to an improved outcome for at least several similar such patients per site each year.

To our knowledge, this is the first phase 3 multicentre randomised controlled trial to show a positive outcome for an acute treatment of intracerebral haemorrhage, and one of few trials that have used a stepped wedge, cluster randomised design across multiple countries.^36^ Trials in intracerebral haemorrhage have been complicated by uncertainty over the optimal timing of the initiation of interventions and in the assessment of outcomes, and in controlling for confounding from comorbid conditions that are either evident at the time of enrolment or arise as a complication of the intervention or condition of the patient during follow-up.^13^, ^14^, ^16^ Previous trials of early intensive blood pressure lowering in acute intracerebral haemorrhage have produced mixed results, because of the variability in the approaches taken towards this treatment and the use of small sample sizes. Individual patient data meta-analyses, where the reliability of the treatment effect is strengthened by an increase in sample size, the inclusion of a broader range of patients, and adjustment for baseline imbalances in prognostic variables, have shown that a careful, targeted, and sustained reduction in systolic blood pressure is safe and is associated with a better functional outcome after intracerebral haemorrhage.^8^, ^9^ However, because these data were derived from conventional trials with restricted inclusion and exclusion criteria, the results are limited to patients with a generally good prognosis with intracerebral haemorrhage of a mild-to-moderate severity according to standard clinical and imaging criteria. We hypothesised that combining early blood pressure lowering treatment with other simple medical interventions would have additive benefits in intracerebral haemorrhage. Such a multifaceted approach is supported by the Quality in Acute Stroke Care trial,^17^ where improved outcomes were seen in patients with acute stroke after nurse-initiated protocols to manage fever, hyperglycaemia, and swallowing dysfunction were implemented at 19 Australian acute stroke units from 2005 to 2010. Moreover, the implementation of a quality improvement protocol involving the reversal of anticoagulation, intensive blood pressure lowering, and rapid triage to neurosurgery and critical care improved survival for patients with acute intracerebral haemorrhage at a large tertiary hospital in the UK.^18^

However, complex relationships are likely to exist between the fidelity of implementation and the effect of the intervention. Intensive blood pressure lowering was the central component of the care bundle, because it has strong supporting evidence, is applicable to a wide variety of settings, and has a plausible mechanism of action in that attenuating the growth of the intracerebral haemorrhage will reduce the primary injury of dissection and compression of brain tissue. Although the rapid control of blood pressure within an early 6 h inclusion time window was emphasised to investigators in this study, most of the growth in intracerebral haemorrhage occurs within a few hours of the onset of symptoms.^37^ Moreover, the effect of blood pressure lowering treatment on this intermediate endpoint is modest and does not have a clear time-dependent association defined to date.^9^ The early correction of an abnormal anticoagulation is likely to benefit patients from a similar mechanism of effect,^38^ but in contrast to ageing populations with ready access to antithrombotic therapies for atrial fibrillation and other cardiovascular disease in high-income countries, only a few patients in our study population presented with anticoagulation-associated intracerebral haemorrhage. Neuroinflammation from the breakdown of red blood cells and blood products is a crucial factor in the cause of secondary brain injury after intracerebral haemorrhage, arising in the perihaematoma region and persisting for several days to weeks.^39^ Because hyperglycaemia^40^ and pyrexia^41^ might potentiate this process, their treatment could provide a crucial adjuvant therapeutic strategy.^42^ Once again, these exposures occurred in only a few participants, with small differences in the amounts of control being achieved between randomly assigned groups. Thus, although the overall treatment effect seems to have been driven by intensive blood pressure lowering, the active multifaceted management of physiological variables and the associated, more intensive, monitoring and nursing care could have affected behaviours and attitudes, including self-management in patients, which translated into benefits that extended after discharge from hospital. A mediation analysis is planned to identify the effects of various components of the care bundle and other key aspects of stroke care on outcomes.

The strengths of our trial include that it comprised an evaluation of a complex intervention, for both implementation in real-world settings and clinical effects, on a robust patient-centred outcome assessed at 6 months. The large sample size allowed for the detection of a modest, but still clinically worthwhile, benefit that is generalisable, because an ethnically and sociodemographically diverse population was included from different resource settings. The broad inclusion criteria allowed testing related to the usual triage and care of patients with acute intracerebral haemorrhage, and the analyses followed a prespecified analysis plan. The provision of the protocol, management algorithms, and a short training package, with individual patient management left to the discretion of the treating team and supported by performance monitoring, reflects how this intervention might be adopted more widely. Whether the implementation difficulties already noted to have happened in China,^24^ in relation to concerns about whether the protocol-defined target concentrations for blood pressure and glycaemic control might harm patients, and whether contextual factors related to staffing processes and medication supply happened more widely, is under investigation through our embedded process evaluation. Given the broad requirement for the standardisation of best practice and quality requirements of well organised systems to have little unwarranted clinical variation in health care, and the absence of any other clearly proven treatment for intracerebral haemorrhage, we anticipate our findings will also support the implementation and enhancement of protocols for intracerebral haemorrhage in high-income countries.

Our trial has limitations. We chose a stepped wedge cluster randomised design to allow large numbers of patients to be rapidly recruited within routine practice, including those with severe illness and requiring urgent neurosurgery, in whom it can be challenging to obtain early consent. The introduction of the care bundle necessitated a change in practice across different clinical services involved in the flow of patients in hospital. These issues made individual randomisation unfeasible, inequitable, and prone to contamination. However, an important potential limitation of this study design is confounding effects from secular trends by calendar time, related to the sequential implementation of the intervention across participating sites, and from disruption to the presentation and management of patients created by the COVID-19 pandemic. There was considerable heterogeneity in the recruitment of patients across sites and in the timing of the phases of the trial. Recruitment was also extended for patients in the intervention group towards the end of the trial, which might have introduced a degree of selection bias. To accommodate this heterogeneity, the extent of which was not fully appreciated until the unmasking of the data, we made a post-hoc decision to adjust the analyses according to calendar time instead of by trial period, as recommended in another stepped wedge trial with delays in cluster recruitment.^31^ We could not find any substantial differences in the characteristics of patients or their management by randomised group, overall, and by region, which is especially relevant for the use of decompressive surgery, mechanical ventilation, and early withdrawal of care, because these interventions have the most potential to influence survival from intracerebral haemorrhage. Even so, the possibility of unmeasured confounding from other changes in the management of patients over time cannot be completely excluded. Our trial was not powered to detect differences across secondary outcomes, with statistical significance varying across the secondary outcomes and generally not present after adjustment for patient characteristics. However, the direction of the effect was consistently in favour of the care bundle across all secondary outcomes. There were logistical and practical issues in delivering the intervention, following up patients, and in monitoring the data quality in LMICs. Bias and measurement error are likely to have influenced the assessment of baseline and outcome measures, such as in the primary use of proxies with wide-ranging sociodemographic and cultural backgrounds to assess the health-related quality of life in patients.^43^

In summary, the findings of our trial provide evidence to support the adoption of an active protocol for intensive blood pressure lowering and the associated management of key abnormal physiological variables within several hours after the onset of signs to improve the recovery of patients presenting with acute intracerebral haemorrhage.

## Data sharing

Individual, de-identified participant data used in these analyses will be shared on request from any qualified investigator after the approval of a protocol and signed data access agreement via both the trial steering committee and the research office of The George Institute for Global Health (Sydney, NSW, Australia).

## Declaration of interests

LS reports funding from the Medical Research Council of the UK, Sichuan Credit Pharmaceutic, and Takeda China; and speaker fees from Takeda China. CSA has received grants from the National Health and Medical Research Council and Medical Research Futures Fund of Australia, the Medical Research Council of the UK, Penumbra, and Takeda China; is also the chair of the data and safety monitoring boards for several trials; is a board member of WHO; and is the Editor-in-Chief of *Cerebrovascular Disease*. CY has received funding from West China Hospital. All other authors declare no competing interests.
