
JOURNAL INFORMATION
==============================
NLM Title Abbreviation: Lancet
Journal ID: Lancet
NLM Journal ID: 2985213R

Lancet (London, England)

ISSN: 0140-6736
EISSN: 1474-547X

ARTICLE INFORMATION
==============================
PMCID: PMC10373951
PMID: 37453751
DOI: 10.1016/S0140-6736(23)01420-4
Article ID: S0140-6736(23)01420-4
Article version: 1
Subjects: Department of Error

Department of Error

Print publication date: 2023 Jul 15
Volume: 402
Issue: 10397
First page: 184
Last page: 184
Copyright: © 2023 The Author(s). Published by Elsevier Ltd.
Copyright year: 2023
Copyright holder: Elsevier Ltd
License: This is an open access article under the CC BY license (http://creativecommons.org/licenses/by/4.0/).
License URL: https://creativecommons.org/licenses/by/4.0/
Erratum for: The third Intensive Care Bundle with Blood Pressure Reduction in Acute Cerebral Haemorrhage Trial (INTERACT3): an international, stepped wedge cluster randomised controlled trial 402 10395 1 7 2023 27 40 Lancet (London, England) 10.1016/S0140-6736(23)00806-1 PMC10401723 37245517

==============================
Ma L, Hu X, Song L, et al. The third Intensive Care Bundle with Blood Pressure Reduction in Acute Cerebral Haemorrhage Trial (INTERACT3): an international, stepped wedge cluster randomised controlled trial. Lancet 2023; 402: 27–40—For this Article, for the INTERACT3 study group and trial investigators listed in the Appendix, the spelling of the names of Kolawole Wahab (appendix p 5) and Olufemi Sanyaolu (appendix p 9) have been corrected. These corrections have been made as of July 13, 2023.

