# Supplementary material for: The third Intensive Care Bundle with Blood Pressure Reduction in Acute Cerebral Haemorrhage Trial (INTERACT3): an international, stepped wedge cluster randomised controlled trial
Source: Lancet. 2023 Jul 1;402(10395):27–40. doi: 10.1016/S0140-6736(23)00806-1 (PMC10401723; doi:10.1016/S0140-6736(23)00806-1)
Supplement: Supplementary appendix [file mmc1.pdf]

THE LANCET

Supplementary appendix

This appendix formed part of the original submission and has been peer reviewed. We post it as supplied by the authors.

Supplement to: Ma L, Hu X, Song L, et al. The third Intensive Care Bundle with Blood Pressure Reduction in Acute Cerebral Haemorrhage Trial (INTERACT3): an international, stepped wedge cluster randomised controlled trial. *Lancet* 2023; published online May 25. https://doi.org/10.1016/S0140-6736(23)00806-1.

Supplementary Appendix

This appendix has been provided by the authors to give readers additional information about their work

Supplement to: Ma L, Hu X, Song L et al. The third Intensive Care Bundle with Blood Pressure Reduction in Acute Cerebral Haemorrhage Trial (INTERACT3): an international, stepped wedge cluster, randomised controlled trial. Lancet 2023

Table of Contents

Section	Title	Page
1	List of INTERACT3 study group and trial investigators	4
2	Study timelines	10
3	The CONSORT 2010 checklist of information to include when reporting a stepped wedge cluster clinical trial	12
4	Agencies providing funding for the study	14
5	Screening procedures	14
6	Inclusion/exclusion criteria	14
7	Training of investigators	14
8	Schedule of monitoring of sites	15
9	Definitions of protocol violations and deviations	16
10	Substudies	17
11	Complete list of major and minor protocol violations	18
12	Sample size calculations	21
13	Terms of reference of the Data and Safety Monitoring Board (DSMB)	21
14	Assessments of functional outcome and health-related quality of life	24
17	Tables	25
	S1. Patient enrolment by site over time from December 2017 to December 2021	26
	S2. Patient enrolment by strata	31
	S3. Calendar windows for time trends	32
	S4. Reasons that patients with acute intracerebral haemorrhage were excluded from participating in the trial	33
	S5. Number of patients enrolled and with primary outcome data per group and per period	34
	S6. Method of assessment of 6 month outcomes	35
	S7. Additional demography, medical history, and use of medications in patients with acute intracerebral haemorrhage	36
	S8. Details of secondary causes of intracerebral haemorrhage	37
	S9. Intravenous blood pressure lowering treatments administered in the first 24 hours and achieved levels of blood pressure	38
	S10. Details of all blood pressure lowering treatments between days 2 and 7	39
	S11. Management and care administered until day 7	40
	S12. Descriptive analysis of clinical outcomes	41
	S13. Multiple imputation for primary outcome	44
	S14. Pre-specified sensitivity analyses of the primary and secondary outcomes	46

	S15. Relationship between intervention effect and calendar time using restricted cubic splines	49
	S16. Post-hoc sensitivity analysis of clinical outcomes with time modelled using restricted cubic spline (RCS)	50
	S17. Prespecified primary analysis with time modelled using the original study period (4 periods)	52
	S18. Family-wise error adjustment across secondary clinical outcomes using a sequential Holm-Sidak correction*	54
	S19. Subgroup analysis for primary outcome on the range of scores on the modified Rankin scale	55
	S20. Baseline characteristics by region	62
	S21. Management of patients over 7 days by region	64
	S22. Serious adverse events summary (investigator reported)	65
	S23. Causes of death	73
18	Figures	77
	S1. Patient enrolment from December 2017 to December 2021, by randomised batches of hospital sites	78
	S2. Patient enrolment from December 2017 to December 2021, by date of enrolment for randomised patient	79
	S3. Boxplot of clinician-reported haematoma volume in patients at baseline and follow-up at 24 hours and 7 days	80
	S4. Boxplot of NIHSS by follow-up assessment	81
	S5. Kaplan Meier curve of mortality over 6 months	82
	S6. Forest plot for subgroup analysis of primary outcome (shift in scores on the modified Rankin scale) at 6 months	83
	S7. Forest plot for subgroup analysis of primary outcome (shift in scores on the modified Rankin scale) at 6 months, only for China sites	84
	S8. Blood pressure plots by region	85
19	Study protocol	88
20	Statistical analysis plan	170

1. List of INTERACT3 study group and trial investigators

Trial Steering Committee

Professor Thompson Robinson (Chair, independent), University of Leicester, Leicester, UK
Professor J. Jaime Miranda (Deputy Chair, independent), Universidad Peruana Cayetano Heredia, Lima, Peru
Professor Craig S. Anderson (Co-Principal Investigator), The George Institute for Global Health Australia and China
Professor Chao You (Co-Principal Investigator), West China Hospital, Chengdu, China
Dr Lili Song (Associate Investigator, Global Project Lead), The George Institute for Global Health China
Professor Adrian Parry-Jones (member, independent), University of Manchester, Manchester, UK
Professor Nikola Sprigg (member, independent), University of Nottingham, Nottingham, UK
Ms Sophie Durrans (observer, 2019-2021), Department of Health and Social Care, London, UK
Ms Caroline Harris (observer, 2021-2023), Department of Health and Social Care, London, UK
Ms Ann Bamford (patient representative), UK
Ms Olivia Smith (patient representative), UK

Data Safety Monitoring Board

Professor Robert Herbert (Chair), University of New South Wales, Sydney, Australia
Professor Christopher Chen, National University Hospital, Singapore
Professor William Whiteley, University of Edinburgh, Edinburgh, UK
Professor Rong Hu, Southwest Hospital, Chongqing, China

Statisticians

Professor Laurent Billot, The George Institute for Global Health, Australia
Mr Qiang Li, The George Institute for Global Health, Australia
Ms Jayanthi Mysore, The George Institute for Global Health, Australia

Medical Review Committee

Associate Professor Xin Hu, West China Hospital, Chengdu, China
Dr Yao Zhang, Shenyang First People’s Hospital, Shenyang Brain Institute, Shenyang, China
Dr Feifeng Liu, Shanghai East Hospital, Tongji University, Shanghai, China

Imaging Adjudication Committee

Dr Yuki Sakamoto, Graduate School of Medicine, Nippon Medical School, Tokyo, Japan
Dr Shoujiang You, The Second Affiliated Hospital of SooChow University, Jiangsu, China
Dr Qiao Han, Suzhou Hospital of Traditional Chinese Medicine, Suzhou, China
Dr Bernard Crutzen, Cliniques Universitaires Saint-Luc, Brussels, Belgium
Dr Yunke Li, The George Institute for Global Health China, Beijing, China
Dr Emily Cheung, Neurology Department, Royal Prince Alfred Hospital, Sydney, Australia

Process Evaluation & Economic Evaluation Committee

Professor Stephen Jan, The George Institute for Global Health, Australia
Dr Hueiming Liu, The George Institute for Global Health, Australia

Dr Menglu Ouyang, The George Institute for Global Health, Australia
Dr Lingli Sun, The George Institute for Global Health China, Beijing, China
Dr Honglin Chu, Research Center of Clinical Epidemiology, Peking University Third Hospital, Beijing, China
Anila Anjum, Clinical Trials Unit, The Aga Khan University, Karachi, Pakistan
Francisca Gonzalez Mc Cawley, Centro de estudios Clínicos, Instituto de Ciencias e Innovación en Medicina. Facultad de Medicina, Universidad del Desarrollo, Chile

Follow-up Centre

China: Shanghai – Yan Wu (Coordinator), Lingling Feng, Jingjing Ni, Caixiu Du, Weiwei Fu
Chile: Centro de Estudios Clínicos, Instituto en Ciencias e Innovación en Medicina, ICIM, Facultad de Medicina- Clínica Alemana, Universidad del Desarrollo - Alejandra del Rio
Brazil: Hospital das Clinicas da Facultade de Medicina de Riberao Pretto – Bruna Rimoli, Rodrigo Cerantola
Sri Lanka: RemediumOne Ptd Ltd - Thanushanthan Jeevarajah, Madhushani Kannangara, Andrene Joseph, Chamath Nanayakkara

International Coordinating Centre (ICC) - The George Institute for Global Health

Project Management – Xiaoying Chen, Alejandra Malavera, Chunmiao Zhang, Zhao Yang, Brook Li, Zhuo Meng, Menglu Ouyang; *Data Management and Programming* – Leibo Liu, Yi Ning, Le Dong, Manuela Armenis; *Quality Assurance* - Joyce Lim, Helen Monaghan.

Regional Coordinating Centres (RCCs)

West China Hospital and The George Institute for Global Health (China): Lu Ma, Xin Hu, Xi Li, Rui Luo, Guojuan Cheng, Yilin Dong, Ziqin Liu, Shuihong Wang, Ying Zhang, Jipeng Cheng, Hui Shi, Wenjing Li, Langming Mou, Ping Yi, Chen Chen, Xue Chen

RemediumOne Pvt Ltd (Sri Lanka): ShalomiWeerawardena (Clinical Research Associate), Poornima Ellawala (Clinical Research Associate), Enalee Ranasinghe (Clinical Research Associate), Chrishmi Rodrigo (Clinical Research Associate).

Horus Co, LTd (Vietnam): Uyen Hong Ha (Project Manager), Linh Le Thi My (Project Manager), Yen Mai Bao (Clinical Research Associate), Duong Hoang Thi Thuy (Clinical Research Associate), Thu Nguyen Hoang (Study Coordinator), Hai Ngo Thanh (Clinical Research Associate).

University of Ilorin Teaching Hospital (Nigeria): KolawalaWahab (National Leader), Sunday Adeniyi (Project Manager).

Christian Medical College and Hospital, Ludhiana (India): Jeyaraj Pandian (National Leader), Megha Khanna (Project Manager).

Centro de Estudios Clínicos, Instituto en Ciencias e Innovación en Medicina, ICIM, Facultad de Medicina- Clínica Alemana, Universidad del Desarrollo (Chile): Paula Muñoz Venturelli (National Leader), Francisca González (Project Manager), Francisca Urrutia Goldsack (Clinical Research Associate), Alejandra Del Río (Clinical Research Associate).

The Aga Khan University (Pakistan): Mohammad Wasay (National Leader), Dilshad Begum (Project Manager), Anila Anjum (Study Coordinator).

INTERACT3 Principal Investigators and Coordinators (centre, with numbers of patients in parentheses)

Brazil

Hospital das Clinicas da Facultade de Medicina de Riberao Pretto (23): Octavio Pontes-Neto, Millene Camilo, Francisco Dias, Octavio Vincenzi, Rodrigo Cerantola; *Neurológica – Neurologista e Neurocirurgião em Joinville SC (6):* Carla Moro, Renata Santos, Nara Texeira, Alexandre Longo, Rafaela Liberato; *Hospital de Clínicas de Porto Alegre (5):* Sheila Martins, Arthur Pille, Bruna Chwal, Isabel Silva, Natacha Titton; *Moinhos de Vento (1)*: GustavoWeiss, Daissy Mora, Magda Martins, Leonardo Carbonera; *Faculty of Medicine of Botucatu, UNESP (1):* Rodrigo Bazan, Gabriel Modolo, Fernanda Winckler, Luana Miranda, Juli Souza.

Chile

Hospital Clinico Herminda Martin (Hospital de Chillan) (15): Alexis Rojo, Wilhelm Uslar, Lorena Medel; *Clínica Alemana de Santiago (8):* Paula Munoz Venturelli, Javiera Lopez, Diego Herrero, Pablo Lavados, Barbara Vargas Latorre; *Hospital Base San José de Osorno (7):* Nathalie Conejan, Tomas Esparza, Patricio Sotomayor; *Hospital Carlos Van Buren (6):* Denisse Wenger, Juan Pablo Gigoux, Aldo Letelier, Lilian Acevedo, Vivianne Moya; *Complejo Asistencial Dr. Victor Rios Ruiz (Hospital de Los Angeles) (4):* Cristian Figueroa, Nicol. Vallejos; *Hospital Santiago Oriente Dr. Luis Tisne (4):* Rodrigo Guerrero, Mauricio Velasquez; *Hospital Metropolitano La Florida (4):* Jose Vallejos, Kimerly Pallauta, Tamara Santibanez, Angelo Queirolo, Andrea Lobos.

China

Suining Central Hospital (224): Yongming Jiang, Weimin Li, Wei Huang, Ke Luo, Gangying Liu; *Liaoning Thrombus Disease Treatment Centre Combing TCM and Western Medicine (205):* Guanghai Tang, Guang Yang, Hongtao Jiang, Xu Zhang, Hongyan Jing; *Dazhu County People's Hospital (200):* Sheng Zhu, Bo Pu, Dong Lv, Hui Kang, Qiuping Hu; *Jiangsu Rudong County People's Hospital (200):* Xiaochun She, Xiaoming Jiang, Yanli Chen, Shenghua Yang, Jianjun He; *Mianyang Central Hospital (165):* Zongping Li, Gang Cheng, Hailin Huang, Xiaoyi Wang, Jianqiong Lin; *Zigong Fourth People's Hospital (162):* Minhui Chen, Chenghao Yang, Hao Ding, Yunliang Deng, Fei Luo; *No.987 Hospital of Joint Logistic Support Force (162):* Rongjun Zhang, Xiaofeng Wang, Hongbing Zhang, Xiaoliang Yang, Yang Zhang; *Dayi County People's Hospital (160):* Chengyi Yang, Yu He, Feng Liu, Rongjie Wang, Yuhui Zhang; *The First People's Hospital of Yuanping (160): Xiaodong Xin, Bin Feng, Wanru Hao, Chang Song, Yun Guo; Xuzhou Central Hospital (160):* Dehua Jiang, Jie Chen, Changtong Tang, Hongliang Zhu; *The First People's Hospital of Shenyang (159*)*:* Xin Li, Jin Cui, Haidong Xu, Boyang Li, Fusheng Tang; *Chongzhou People's Hospital (153):* Yuanbin Li, Min Gao, Bo Yang; *Chengdu Second People's Hospital (146):* Xuejun Xu, Bing Deng, Yi Zheng, Yuanhong Ge, Keyu Chen; *The Third Hospital of Mianyang (142):* Yang Liu, Xinshen Li, Tingting Zhong, Jianfeng Xu, Hai Zhang; *Liaocheng People's Hospital (142):* Jiyue Wang, Jianxin Zhu, Hanyu Sun, Fuhua Yu, Xueguang Zhang; *West China Hospital Sichuan University (126):* Chao You, Lu Ma, Xin Hu, Jianguo Xu, Xi Li; *The Second People's Hospital of Neijiang (121):* Mingsen Zhang, Bin Wang, Yiming Ma, Donglin Jiang, Jun Zhou; *The First People's Hospital of Neijiang (120):* Cong Liu, Wenhong Nie, Mingguo Li, Tao Tian, Yong Li; *Guangyuan Central Hospital (120):* Mingfang He, Xiaolong Tu, Zhengjun Wu, Hong Liu, Dongsheng Zhong;

Tianjin Medical University Central General Hospital (120): Rongcai Jiang, Jian Sun, Ye Tian, Yingsheng Wei, Shuo An; *Mianzhu People's Hospital (118):* Pingbo Wei, Le Luo, Bin Lin, Gang Liu, Yan Wen; *Renmin Hospital of Wuhan University (116):* Qiang Cai, Qianxue Chen, Pan Lei, Zhiyang Li, Meifang Zhang; *Nanchong Central Hospital (113):* Jiaquan He, Yan Chen; *The First Hospital of Kunming (112):* Jun Liu, Xinghai Liu, Junyan Li, Min Chen, Jing Wang; *Xinhua Hospital of Zhejiang Province (112):* Bingzhi Zhou, Baichun Ye, Jiancheng Zhang, Manyuan Zhang, Xuming Pan; *Wuhan Third Hospital-Tongren Hospital of Wuhan University (109):* Xiaoxiang Yu, Jian Xu, Qingbao Xiao, Yuefei Wang, Liang Tao; *Dezhou People's Hospital (93):* Lin Shi; *The People's Hospital of Leshan (91):* Niandong Zheng, Guoliang You, Bo Lei, Shu Chen, Honggang Wu; *Huashan Hospital of Fudan University Neurosurgery Department (88):* Jin Hu, Jianlan Zhao, Jian Yu, Qiang Yuan, Zhuoying Du; *Santai County People's Hospital (84):* Xielin Tang, Qianke Li, Shenghua Liu, Feilong Yang, Kui Xiao; *Chongqing Traditional Chinese Medicine Hospital (84):* Chao Luo, Guang Wang, Xudong Che, Zhipeng Teng, Wenwu Wan; *The Central Hospital of Wuhan (84):* Jun Li, Yu Liu, Mingbo Fan, Tao Zhang, Lun Cai; *The People's Hospital of Hejian (81):* Yuan Ma, Zhifeng Ma, Bin Li, Linlin He, Jinghui Li; *Dong'e People's Hospital (81):* Weibing Zhang, Shuxin Zhang, Hongzhen Zhang, Yingguang Dai; *The First People's Hospital of Shuangliu District, Chengdu (80):* Jun Lei, Lei Mao, Yiyang Huang, Zhi Zhou, Ping Chen; *The First People's Hospital of Longquanyi District, Chengdu (80):* Fang Chen, Pan Wei, Tiangui Li, Honglin Chen, Mengfei Zeng; *Chongqing Bishan District People's Hospital (80):* Kejie Mou, Jun Xue, Yong Jiang; *Affiliated Hospital of North Sichuan Medical College(80):* Xiaoping Tang, Tao Chen, Yalan Zhang, Yanbing Xu, Yuchen Gu; *Tianjin Fifth Central Hospital (80):* Lei Chen, Yujun Zhao, Bin Yang, Peng Kuai, Xi Wang; *Dancheng County People's Hospital(80):* Yuwang Yang, Xueling Hu, Huitian Zhang, Yintao Yang, Weifeng Wang; *People's Hospital of Ordos Dongsheng District (78):* Junyi Zhang, Wei Cheng, Xiaoxue Zhang, Xiaowen Ma, Qin He; *Zhangjiagang First People's Hospital (74):* Li Zhang, Rong Gao, Huixiang Liu, Jingwei Ye, Ping Xu; *Yantai Yuhuangding Hospital (73):* Xin Wu, Yuan Yuan, Peng Zou, Zhen Zhang, Jiyong Cheng; *Dujiangyan People's Hospital (70):* Zhangming Zhou, Yijun Zeng, Zhang Liang, Deming Du, Shui Yu; *The Second Affiliated Hospital of Suzhou University (67):* Yongjun Cao, Shoujiang You, Jiaping Xu, Zhichao Huang, Dongqin Chen; Sichuan *Mianyang 404 Hospital (56):* Wenfeng Xiao, Li Zhu, Miao Yuan; *The 904th Hospital of the Joint Logistics Support Force of the Chinese People's Liberation Army (55):* Yuhai Wang, Dongliang Shi, Xu Hu, Dingchao Xiang, Like Shi; *The First Hospital of Shanxi Medical University (53):* Hongqin Wang, Liu Yang, Wang Miao, Yiyi Hu, Yuchun Zhao; *Chongqing Emergency Medical Center (50):* Xi Hu, Yang Liu, Weiduo Zhou, Chao Sun; *Jingzhou Central Hospital (45):* Tao Chen, Dong Tang, Kun Yao, Jin You, Shishi Chen; *Nanhe County People's Hospital (44):* Jianmin Yao, Huanmei Li, Jinmei Liu, Ailin Bai; *The Second People's Hospital of Yibin (41):* Yong Yi, Qingshan Deng, Peng Luo, Han Wang, Jingcheng Jiang; *Zhongshan Hospital Affiliated to Xiamen University (40):* Qingwei Yang, Shunpo He; *Pangang Group General Hospital (39):* Jun Wang, Yu Chen, Hua He, Yuyang Deng; *Guangzhou First People's Hospital (38):* Zhikai Cao, Xuxia Yi, Jinbiao Luo; *Chengdu Fifth People's Hospital (37):* Shuang Luo, Min Gong, Li Liu, Xuejun Gao, Jia Liu; *The First Affiliated Hospital of Baotou Medical College (36):* Li'e Wu, Jia Zhang, Hongying Sun, Xinhui Li, Lu Jia; *Yaan People's Hospital (33):* Jianbing Wu, Jie Zhang, Huajun Zhang, Chunfu Du, Shun Li; *The First People's Hospital of Yibin (32):*

Xiaobin Yang, Jie He, Lei Liao; *Jinhua Municipal Central Hospital (31):* Gezhi Zhou, Wentao Dong, Yunxiang Chen, Xiaofeng Lin, Xujian Shui; *No.988 Hospital of Joint Logistic Support Force (31):* Peng Zhang, Yuan Zhao, Hongli Yang, Wenbin Zhao, Xiaoyi Zhang; *Zhongnan Hospital of Wuhan University (31):* Jincao Chen, Qian Wu, Xuan Dai; *Xinghua Traditional Chinese Medical Hospital (29):* Baogui Tang, Yinjuan Wang; *Peking University Third Hospital Yanqing Hospital (28):* Tao Liu, Haixia Zhang; *Wuhan No.1 Hospital (26):* Faliang Duan, Ming Luo; *Shijiazhuang People's Hospital (25):*Qingfang Jiao, Guoliang Lei, Dong Wang, Chunwang Song, Haopeng Tan; *People's Hospital of Deyang City(24):* Feng Ye, Xinghu Qin, Xiaolong Liang, Junling Liu, Lang Yang; *The First Affiliated Hospital of Chengdu Medical College (22):* Jie Yang, Yapeng Lin, Qian Yang, Xuntai Ma, Yinkuang Qi; *Hebei General Hospital (20):* Baogen Pan, Caixia Jiang, Zhanying Ye, Ce Dong, Xiongfei Yue; *People's Hospital of Xinjiang Uygur Autonomous Region (20):* Xiaopeng Yang, Tuoheti Maimaitiyiming, Jun Dong, Yonggang Wu, Feng Gao; *Tieling Central Hospital (19):* Deqiang Zhao, Xinghai Zhang; *The First People's Hospital of Yiliang (15):* Pengjun Wang, Hongbo Jiang, Jianping Li, Wei Zhang, Jing Chen; *Shanxi Dayi Hospital (15):* Haibo Tong, Yonghong Wang, Kaipeng Qiao; *The First Affiliated Hospital of Zhengzhou University (12):* Fuyou Guo, Mingchu Zhang, Yan Hu, Mengzhao Feng, Dengpan Song; *No.215 Hospital of Shanxi Nuclear Industry (12):* Yi Zuo, Shangjun Chen, Chao Qian, Baoming Li, Jingku Ma; *The Third People's Hospital of Chengdu (11):* Sunfu Zhang, Bin Kong, Xingyu Dong, Qiang Li, Sheng Fang; *People's Hospital of Qiandongnan Miao and Dong Autonomous (7):* Bin Lu, Yang Li, Zhen Zhang, Yongling Yang, Hong Yu; *Tiemei General Hospital of Liaoning Province Health Industry Group (7):* Huaiyu Sun, Yue Wang; *Qingdao Municipal Hospital (7):* Weimin Wang, Tong Li, Shengli Li, Zhiming Xu, Yongyi Wang; *Huashan Hospital of Fudan University Neurology Department (7):* Qiang Dong, Yuping Tang, Heling Chu, Ying Lu; *The First Affiliated Hospital of Suzhou University (5):* Zhong Wang, Xiaoou Sun; *The First People's Hospital of Yunnan Province (1):* Jianhua Zhao, Shuaifeng Yang, Xiying Qian.

India
KLES Dr. Prabhakar Kore Hospital & MRC (28): Aralikatte Saroja, Ravishankar Naik, Sandip Chindhi, Nakul Pampaniya, Kurubara Amaresh; *Government Medical College, Thiruvananthapuram (16):* Thomas Iype, Dileep R, Reeja Rajan, Praveen Panicker; *GNRC Hospital Dispur (12):* Rupjyoti Das, Nupur Choudhury, Pankaja Gohain; *Baptist Christian Hospital, Assam (5):* Jemin Webster, Biyol Pakma, Lalbiak Sangi; *St. Stephen's Hospital, Delhi (4):* Ivy Sebastian, Gaurav Aggrawal, Komal Raj, Deepankshi Rajoura; *Guru Gobind Singh Hospital, Faridkot (2):* Sulena Singh, Varun Aggrawal, Amit Narang.

Mexico
Instittuto Nacional de Neurologia y Neurocirugia (9): Antonio Arauz, Vanessa Cano, Diego López, Hector Valdéz, Roberto Toledo.

Nigeria
Ahmadu Bello University Teaching Hospital (29): Reginald Obiako, Sani Abubakar, Oguike Emeka, Balogun Olayemi, Melika Lois, Ibinaiye Philip, Olurishe Comfort O; *Lagos University Teaching Hospital (26):* Njideka Okubadejo, Osigwe Agabi, Oluwadamilola Ojo; *University of Ilorin Teaching Hospital (15):* Kolawole Wahab, Abiodun Bello, Oyinloye Ibukun, Olufemi Sanayaolu, Sunday Adeniyi, Abdulraheem Jimoh.

Pakistan

Aga Khan University (36): Mohammad Wasay, Dilshad Begum, Anila Anjum, Shanid Waheed, Ayeesha Kamal; *Shifa International Hospital (12):* Raja Shoiab, Fizza Orooj, Sadaf Majid, Taskeen Zehra Abdus Khan; *South City Hospital (1):* Ravi Shanker, Nadir Syed, Nashwa Ahmad.

Peru

Instituto Nacional de Ciencias Neurológicas (12): Carlos Abanto, Ana Valencia, Danny Barrientos, Jorge Ramirez, Pilar Calle.

Sri Lanka

Kurunegala Teaching Hospital (103): Dilum Palliyeguruge, Sumudu Muthucumarana, Shiroma Ratnayaka, Dilhara Ganihiarachchi, Arundathi Bandaranayake, S.D.B. Somaratne, Saumya Narayana, Sithara Gallage; *National Hospital of Sri Lanka (50):* Bimsara Senanayake, Udari Samarasiri, Dunya Luke, Mythily Sivpathasundaram, Vithoosan Sahadevan, Amani Rasmi, Yuran Deshaka; *Gampaha District General Hospital (24):* Nilukshi Fernando, Aruna Munasinghe, Kapilanga Rathnapriya, A.S. Nissanka, Kanchana Karunathilake, Isuru Gayan, Kaminda Wijenayake, Hasitha Gunasekara, Jagath Vidyarathne; *Jaffna Teaching Hospital (22):* Ajantha Keshavaraj, Kanagasabapathy Janarthanan, Arhivalaky Gerald Jeevathasan, Sivaram Sivamainthan, Mathyamuthan John Priyanth, Abirami John Priyanth; *Colombo South Teaching Hospital (16):* Thambippillai Rajendiran, Sanjeewa Alwis, Nushara Gunasekare, Vasundara Liyanarachchi; *Karapitiya Teaching Hospital (11):* Athula Dissanayake, Wimalasiri Uluwattage, Gimhani Ratnayake, Charika Rajinee, Sakura Jayawardana; *Kandy National Hospital (8):* Janaka Peiris, Ranjith Wicramasinghe, Chamila Fernando, Jessie Abbas, Nethmini Withanage, Makaranda Bandara.

Vietnam

Bach Mai Hospital (95): Duy Ton Mai, Van Chi Nguyen, Viet Phuong Dao, Xuan Trung Vuong, Tien Dung Nguyen, Trung Hieu Dinh, Ha Quan Phan, Quoc Viet Bui, Dinh Tho Phung, Quang ThoPham; *103 Military Hospital (20):* Dinh Dai Pham, Duc Thuan Do, Phuc Duc Dang, Minh Duc Dang, Dang Hai Nguyen; *Thong Nhat Hospital (16):* Thi Phuong Nga Nguyen, Quoc Huy Nguyen, Quoc Dai Pham, Quoc Vinh Chau, Vinh Thy Van Tai; *Thu Duc District Hospital (11):* Tran Vinh Le, Cong Tri Le, Ha Mai Khuong Tran, Huu Khan Nguyen, Hoang Minh Thao Nguyen; *Nguyen Tri Phuong Hospital (10):* Duc Chien Vo, Thai My Phuong Nguyen, Trung Thanh Tran, Thi Hanh Vi Vo, Hao Nhien Cao; *University Medical Hospital (3):* Ba Thang Nguyen, Thi Ngoc Suong Le, Thien Duc La, Chi Duc Pham, Huy Thai.

2. Study timelines

Date	Name of document / event	Summary of changes / notes
1 July 2017	INTERACT3 Protocol V1.1	
28 November 2017	DSMB First Meeting	Kick-off meeting<br>Protocol overview<br>DSMB charter review and finalisation<br>DSMB mock table review
12 December 2017	Recruitment	First participant screened at West China Hospital, China<br>First participant enrolled at West China Hospital, China
8 April 2018	INTERACT3 Protocol V2.0 Amendment 1	Extend the time limitation for each phase to 4 months (only in Asia)<br>Additional funding source from Takeda China<br>Set up a transition period of 7-10 days before entering intervention phase<br>Change to randomisation program to stratify by both country and size of site<br>Adjust the study timelines
6 November 2018	DSMB Second Meeting	Data from 1774 participants reviewed by DSMB members<br>Trial continuation recommended
12 August 2019	INTERACT3 Protocol V3.0 Amendment 2	Expand to 110 sites and set up the organisation of RCCs<br>Extend the study period to 2022<br>Add process evaluation and economic evaluation alongside the trial<br>Add inclusion criteria of intracerebral haemorrhage diagnosis within 6 hours from onset<br>Change opt-out approach to consent withdrawal<br>Recalculate sample size with target an average of 19 patients per site per phase
23 September 2019	DSMB Third Meeting	Data from 4427 participants reviewed by DSMB members<br>Trial continuation recommended
17 April 2020	MRC joint grant awarded	

17 June 2020	TSC First Meeting	New Trial Steering Committee (TSC) established as per MRC funding requirement to involve independent members and patient representatives<br>TSC charter review and finalisation<br>Safety reporting and DSMB report overview<br>Ethics Committee application in UK<br>Initiation of new countries
23 November 2020	TSC Second Meeting	Compliance to the new China regulatory – HGRA<br>Study progress oversight<br>Strategy over impact of COVID-19
28 April 2021	DSMB Fourth Meeting	Data from 6180 participants reviewed by DSMB members<br>Trial continuation recommended
25 May 2021	TSC Third Meeting	Trial progress oversight<br>Next DSMB meeting planned
18 August 2021	DSMB Fifth Meeting	Data reviewed from 6765 participants by DSMB members<br>Trial continuation recommended
28 October 2021	TSC Fourth Meeting	Trial progress oversight<br>Discussion on SAP writing issues – consideration of COVID impact<br>Publication plan
20 December 2021	INTERACT3 protocol published	Trials. 2021 Dec 20;22(1):943. doi: 10.1186/s13063-021-05881-7.
31 December 2021	Recruitment completion	7067 recruitment was achieved globally
17 August 2022	Patient follow-up completion	Last 6-month outcome assessment for participants
5 September 2022	INTERACT3 SAP published	Cerebrovasc Dis. 2022 Sep 5:1-4. doi: 10.1159/000526384.
17 November 2022	Database lock	
20 December 2022	Unblinding of data to Steering Committee	

3. Reporting of stepped wedge cluster randomized trials: checklist for an extension of the CONSORT 2010 statement

Supplementary materials 3: Checklist of information to include when reporting a stepped wedge cluster randomised trial (SW-CRT)

Topic	Item no	Checklist item	Page no
**Title and abstract**			
	1a	Identification as a SW-CRT in the title.	1
	1b	Structured summary of trial design, methods, results, and conclusions (see separate SW-CRT checklist for abstracts).	4-5
**Introduction**			
Background and objectives	2a	Scientific background. Rationale for using a cluster design and rationale for using a stepped wedge design.	9-10
	2b	Specific objectives or hypotheses.	10
**Methods**			
Trial design	3a	Description and diagram of trial design including definition of cluster, number of sequences, number of clusters randomised to each sequence, number of periods, duration of time between each step, and whether the participants assessed in different periods are the same people, different people, or a mixture.	10-14
	3b	Important changes to methods after trial commencement (such as eligibility criteria), with reasons.	11, 13
Participants	4a	Eligibility criteria for clusters and participants.	11-12
	4b	Settings and locations where the data were collected.	10, 14
Interventions	5	The intervention and control conditions with sufficient details to allow replication, including whether the intervention was maintained or repeated, and whether it was delivered at the cluster level, the individual participant level, or both.	13-14
Outcomes	6a	Completely defined prespecified primary and secondary outcome measures, including how and when they were assessed.	15
	6b	Any changes to trial outcomes after the trial commenced, with reasons.	15-16
Sample size	7a	How sample size was determined. Method of calculation and relevant parameters with sufficient detail so the calculation can be replicated. Assumptions made about correlations between outcomes of participants from the same cluster. (see separate checklist for SW-CRT sample size items).	15-16
	7b	When applicable, explanation of any interim analyses and stopping guidelines.	17
**Randomisation**			
Sequence generation	8a	Method used to generate the random allocation to the sequences of treatments.	12
	8b	Type of randomisation; details of any constrained randomisation or stratification, if used.	12
Allocation concealment mechanism	9	Specification that allocation was based on clusters; description of any methods used to conceal the allocation from the clusters until after recruitment.	12
Implementation	10a	Who generated the randomisation schedule, who enrolled clusters, and who assigned clusters to sequences.	12
	10b	Mechanism by which individual participants were included in clusters for the purposes of the trial (such as complete enumeration, random sampling; continuous recruitment or ascertainment; or recruitment at a fixed point in time), including who recruited or identified participants.	12
	10c	Whether, from whom and when consent was sought and for what; whether this differed between treatment conditions.	11
Blinding	11a	If done, who was blinded after assignment to sequences (eg, cluster level participants, individual level participants, those assessing outcomes) and how.	12
	11b	If relevant, description of the similarity of treatments.	13
Statistical methods	12a	Statistical methods used to compare treatment conditions for primary and secondary outcomes including how time effects, clustering and repeated measures were taken into account.	16-17
	12b	Methods for additional analyses, such as subgroup analyses, sensitivity analyses, and adjusted analyses.	17

Supplementary materials 3 (*Continued*)			
**Topic**	**Item no**	**Checklist item**	**Page no**
**Results**			
Participant flow (a diagram is strongly recommended)	13a	For each treatment condition or allocated sequence, the numbers of clusters and participants who were assessed for eligibility, were randomly assigned, received intended treatments, and were analysed for the primary outcome (see separate SW-CRT flow chart).	35
	13b	For each treatment condition or allocated sequence, losses and exclusions for both clusters and participants with reasons.	35, appendix
Recruitment	14a	Dates defining the steps, initiation of intervention, and deviations from planned dates. Dates defining recruitment and follow-up for participants.	18
	14b	Why the trial ended or was stopped.	18
Baseline data	15	Baseline characteristics for the individual and cluster levels as applicable for each treatment condition or allocated sequence.	39-40 appendix
Numbers analysed	16	The number of observations and clusters included in each analysis for each treatment condition and whether the analysis was according to the allocated schedule.	35
Outcomes and estimation	17a	For each primary and secondary outcome, results for each treatment condition, and the estimated effect size and its precision (such as 95% confidence interval); any correlations (or covariances) and time effects estimated in the analysis.	44
	17b	For binary outcomes, presentation of both absolute and relative effect sizes is recommended.	38, 44, 22
Ancillary analyses	18	Results of any other analyses performed, including subgroup analyses and adjusted analyses, distinguishing prespecified from exploratory.	appendix
Harms	19	Important harms or unintended effects in each treatment condition (for specific guidance see CONSORT for harms).	44 appendix
**Discussion**			
Limitations	20	Trial limitations, addressing sources of potential bias, imprecision, and, if relevant, multiplicity of analyses.	25-26
Generalisability	21	Generalisability (external validity, applicability) of the trial findings. Generalisability to clusters or individual participants, or both (as relevant).	25
Interpretation	22	Interpretation consistent with results, balancing benefits and harms, and considering other relevant evidence.	23-24
**Other information**			
Registration	23	Registration number and name of trial registry.	5
Protocol	24	Where the full trial protocol can be accessed, if available.	11
Funding	25	Sources of funding and other support (such as supply of drugs), and the role of funders.	6,27
Research ethics review	26	Whether the study was approved by a research ethics committee, with identification of the review committee(s). Justification for any waiver or modification of informed consent requirements.	11

This checklist has been taken from table 3 in *BMJ* 2018;363:k1614, as a standalone document for readers to print out or fill in electronically.

4. Agencies providing funding for the study

Funding from the Department of Health and Social Care, the Foreign, Commonwealth & Development Office, the Medical Research Council and Wellcome Trust (MR/T005009/1); the National Health and Medical Research Council (NHMRC) of Australia (APP1149987); the West China Hospital Outstanding Discipline Development 1-3-5 Program (ZY2016102); and Sichuan Credit Pharmaceutical Co., Ltd, and Takeda (China) International Trading Co., Ltd.

5. Screening procedures

Participating hospital sites were required to fill in a screen log which was set up in the database for all potentially eligible patients presenting with acute intracerebral haemorrhage (ICH) confirmed by brain imaging. The screening log will record patient's gender, initials, date and time of hospital arrival, date and time of stroke onset, date and time of CT scan, patient information form given, and consent obtained and patient's ineligibility.

6. Inclusion/exclusion criteria

Patient specific inclusion criteria: All patients were eligible if they fulfilled general eligibility

- aged ≥18 years
- Acute stroke syndrome that is due to presumed spontaneous ICH, defined as the sudden occurrence of bleeding into the parenchyma of the brain that may extend into the ventricles and, in rare cases, into the subarachnoid space, confirmed by clinical history and a CT scan within 6 hours of stroke onset with/without contrast, and if an CT angiogram is also undertaken as part of routine care. (NB Patients with ICH secondary to medical treatment [e.g. antiplatelet or antithrombotic therapy], are eligible, but ICH secondary to thrombolysis are ineligible).
- Presentation to hospital within 6 hours of stroke onset. (NB If the precise timing of the first symptoms or signs of the qualifying event are unknown then the time of onset will be taken as the last time at which the patient was known to be well).

Patients were *NOT be eligible* if there was one or more of the following:

- Definite evidence that the ICH is secondary to a structural abnormality in the brain (eg an AVM, intracranial aneurysm, tumour, trauma, or previous cerebral infarction) or previous thrombolysis.
- A high likelihood that the patient will not adhere to the study treatment and follow-up regimen. In each case, the decision about the patient's eligibility will be based on the attending clinician's interpretation of the above eligibility criteria.

All eligible patients will be provided with an approved patient information sheet (PIS) or patient responsible information sheet (PRIS) and consent form (CF) on admission for permission to collect their medical and personal information and to be contacted at 6 months follow-up.

7. Training of investigators

Two investigator meetings were provided for prior-study training in the protocol and Good Clinical Practice (GCP) procedures to site principal investigators and sub-investigators in May and June 2017 at Chengdu and Beijing, China, respectively. All INTERACT3 investigators

were in-person or online trained at site activation in the protocol except care bundle details, GCP procedures, and performance of the National institute for Health Stroke Scale (NIHSS) and modified Rankin scale (mRS) if they had no recent certification or training. After 7-10 days of recruitment completion in the usual care period, site investigators were trained in the care bundle interventions just before the site was switched to intervention period. Furthermore, a process evaluation was undertaken in 18 sites globally including investigator interview and focus group discussion to identify barriers and solutions for the implementation of care bundle interventions. In the intervention period, each site was provided at least twice online or onsite training about implementing care bundle intervention using materials of site performance as well as results derived from the process evaluation.

Dr Chen Chen, Shanghai East Hospital, Shanghai, China, was on-line certified in the use of mRS, whilst Drs Chen Chen and Xin Hu obtained an online certificate in the use of NIHSS. They provided training and certification to assessors in sites or follow-up center who had not received similar online training and certification. All such assessors received at least three individual sessions of mRS training via Zoom meetings during the study period. After each training session, the assessors received an examination to ensure that they were suitably qualified. Details of the content, frequency and evaluation of assessors is available upon request.

8. Schedule for monitoring of sites

Project management team undertook quality control activities necessary for the conduct of the trial in accordance with the protocols, applicable guidelines, and regulations. The first completed the target recruitment in the control phase and was about to step-up for the intervention phase. Subsequent monitoring visits took place as required by the ICC, but the interval for monitoring visits varied according to patient enrolment, quality issues, trial site compliance, or other trial site issues. All sites were monitored at least once per year. Any significant deviation from the planned monitoring timelines was explained and documented in the monitoring visit report, and the monitoring plan was amended if appropriate.

The monitoring visit served to obtain 100 source data verification of the following data for all enrolled patients: patient consent forms (patient consent forms were reviewed for compliance with ICH GCP); patient existence; diagnosis of intracerebral hemorrhage; and all serious adverse event forms to source verification.

For 10 to 30 of randomly selected enrolled patients, or patients identified by the International Coordinating Center (ICC), all data entered in the electronic case record form (eCRF) were verified against source data.

The INTERACT3study was affected by the COVID-19 pandemic. Because of the requirement for epidemic prevention policies to be enacted in many participating hospitals, project staff were unable to undertake monitoring activities directly at sites during certain periods in 2021 and 2022. In order to ensure the integrity and authenticity of key data, the project team in cooperation with staff in data management undertook risk-based monitoring of the data on the participants. The project team developed a series of checklist templates for key data variables to allow communications with site investigators about logical errors and other issues raised in

these data reports. Through video teleconference with investigators, relevant source data and medical record files were displayed for confirmation of data as correct.

At the end of the study, 121 sites had received at least 3 monitoring visits, which culminated in a total of 367 on-site monitoring and over 2000 monthly remote monitoring calls.

9. Definitions of protocol violations and deviations

Protocol deviation / violations were any unapproved changes, or departures from the study design or procedures of the INTERACT3 protocol, that were under the investigator's control and had not been reviewed and approved by the International Coordinating Center (ICC) or IRB/EC. Protocol deviation / violations had two categories: 'major (reportable) violations', and 'minor (non-reportable) violations' which were also called 'Protocol Deviations'.

Major (reportable) protocol violations

Major protocol violations were any unapproved changes in the research study design and/or procedures that were within the investigator's control, and not in accordance with the approved protocol, that may have affected the participant's rights, safety or well-being, or the completeness, accuracy, and reliability of the study data. All major violations were required to be reported to the IRB/EC, regulatory authority and/or sponsor, in keeping with relevant national guidance and conforming to national timelines for reporting. The ICC criteria for defining major violations included any of the following events:

1) The violation had harmed, or posed a significant or substantive risk of harm, to the research participant;
2) The violation had resulted in a change to the participant's clinical or emotional condition or status;
3) The violation had damaged the scientific completeness or soundness of the data collected for the study;
4) The violation was evidence of willful or knowing misconduct on the part of the investigator(s);
5) The violation involved serious or continuing noncompliance with federal, state or local regulations.

Examples of major protocol violations included, but were not limited to:

a) enrolment of a participant who did not meet the eligibility criteria;
b) failure to obtain informed consent prior to any study-specific procedure;
c) failure to follow protocol procedures that specifically related to intervention and the primary safety or efficacy endpoints of the study.

Minor (non-reportable) protocol violations (also called protocol deviations)

Minor protocol violations were any unapproved changes in the research study design and/or procedures that are within the investigator's control and not in accordance with the approved protocol that do not have a major impact on either the participant's rights, safety or well-being, or the completeness, accuracy, and reliability of the study data. Minor protocol violations were

not necessarily reportable to the IRB/EC. ICC criteria for minor violations included all of the following:

I. the violation did not harm or pose a significant risk of substantive harm to the research participant, and
II. the violation did not result in a change to the participant's clinical or emotional condition or status, and
III. the violation did not damage the completeness, accuracy and reliability of the data collected for the study, and
IV. the violation did not result from willful or knowing misconduct on the part of the investigator(s), and
V. the violation did not involve serious or continuing noncompliance with federal, state or local regulations.

Examples of minor protocol violations included, but are not limited to:<br>1) patient being unable to complete the self-administered quality of life questionnaire when they were capable of doing so;<br>2) follow-up visits / assessments were performed outside of protocol defined time points or time window.

10. Substudies

Effects of treatment on haematoma growth in ICH

The effects of treatment on haematoma expansion and other indices including perihaematoma oedema will be evaluated in a sub-sample of at least 1000 patients (the earliest 7 recruitments in control group and the earliest 7 recruitments in intensive group for each site). Apart from the CT scan at baseline, repeat CT scans (24±3 hours and 7 days) are required. The primary efficacy measure is proportional change ('growth') in haematoma volume at 24 hours. Clinical outcomes are assessed over 6 months.

CT imaging will be conducted according to standardised techniques and must be uploaded to the INTERACT3 server, either directly from the hospital site (if they have suitable broadband internet) or via the RCC office. The LCC will keep a hard copy in an uncompressed DICOM format onto a CD-ROM for monitor site verification. Trial management is facilitated by an established internet-based system. The brain imaging system allows assessment of abnormalities using computer-assisted multi-slice planimetric and voxel threshold techniques in MIStar version 3.2 (Apollo Medical Imaging Technology, Melbourne, Victoria, Australia). The system was built to store securely over 10,000 images acquired from participants.

The imaging data will be analysed centrally by trained experts who will be kept blind to the treatment allocation. During the reading process, approximately 5 and 5 of scans from each reviewer were randomly selected and re-assigned to the same reviewer and to another reviewer for interclass and intraclass reliability assessment, respectively.

The results of this substudy will be reported separately.

11. Complete list of major and minor protocol violations*

Code	Major or Minor	Description 1	Description 2	Intervention	Control	Total (N=7036)
301	MAJOR	Hospital arrival date	Not collected or recorded	0/3221 (0.0)	0/3815 (0.0)	0/7036 (0.0)
302	MAJOR	Hospital arrival time	Not collected or recorded	0/3221 (0.0)	0/3815 (0.0)	0/7036 (0.0)
303	MAJOR	Patient last seen well date and time (24 hour clock)	Not collected or recorded	0/3221 (0.0)	1/3815 (0.0)	1/7036 (0.0)
304	MAJOR	CT scans date & time	Not collected or recorded	3/3221 (0.1)	9/3815 (0.2)	12/7036 (0.2)
306	MAJOR	Inclusion in INTERACT3	Diagnosis not spontaneous ICH	0/3221 (0.0)	0/3815 (0.0)	0/7036 (0.0)
307	MAJOR	Inclusion in INTERACT3	Presentation out of 6 hours of last seen well (symptom onset)	28/3221 (0.9)	15/3815 (0.4)	43/7036 (0.6)
308	MAJOR	Consent	The informed consent form of this subject is lost due to improper storage by the investigator	5/3221 (0.2)	0/3815 (0.0)	5/7036 (0.1)
309	MAJOR	Consent	Consent from PR/subject obtained after 72 hours of admission only for the intervention	0/3221 (0.0)	4/3815 (0.1)	4/7036 (0.1)
311	MAJOR	Consent	Consent given by non-authorised people	1/3221 (0.0)	0/3815 (0.0)	1/7036 (0.0)
321	minor	Consent	Missing PR signature: PR signed in the wrong place.	0/3221 (0.0)	20/3815 (0.5)	20/7036 (0.3)
324	MAJOR	Consent	missing PR date	3/3221 (0.1)	0/3815 (0.0)	3/7036 (0.0)
330	MAJOR	Consent	Missing witness signature: witness didn't sign at all	3/3221 (0.1)	0/3815 (0.0)	3/7036 (0.0)
341	minor	Consent	Other	2/3221 (0.1)	0/3815 (0.0)	2/7036 (0.0)
344	minor	Consent	PISCF not approved by ethics (amendment does not affect subject safety)	0/3221 (0.0)	5/3815 (0.1)	5/7036 (0.1)
346	minor	Consent	Expired PISCF (current version does not affect subject safety)	14/3221 (0.4)	4/3815 (0.1)	18/7036 (0.3)
400	minor	Hospital Arrival Details	Not collected or recorded	157/3221 (4.9)	257/3815 (6.7)	414/7036 (5.9) [a]
401	minor	Baseline imaging scans	Not collected or recorded	0/3221 (0.0)	4/3815 (0.1)	4/7036 (0.1) [a]
402	minor	Medical history	Not collected or recorded	7/3221 (0.2)	88/3815 (2.3)	95/7036 (1.4) [a]
403	minor	Medications at time of admission	Not collected or recorded	0/3221 (0.0)	2/3815 (0.1)	2/7036 (0.0) [a]
404	minor	Admission details	Not collected or recorded	4/3221 (0.1)	6/3815 (0.2)	10/7036 (0.1) [a]
405	minor	Ethnicity	Not collected or recorded	0/3221 (0.0)	1/3815 (0.0)	1/7036 (0.0)
500	minor	Highest and lowest BP recorded during the first 24 hours	Not collected or recorded	5/3221 (0.2);	22/3815 (0.6)	27/7036 (0.4)
501	minor	Highest and lowest BGL recorded during the first 24 hours	Not collected or recorded	329/3221 (10.2)	797/3815 (20.9)	1126/7036 (16.0)

502	minor	Highest and lowest body temperature recorded during the first 24 hours	Not collected or recorded	10/3221 (0.3)	111/3815 (2.9)	121/7036 (1.7)
503	minor	INR measurement	Not collected or recorded	873/3221 (27.1)	1501/3815 (39.3)	2374/7036 (33.7)
505	minor	Medication used for anticoagulation reversal	Not collected or recorded	9/3221 (0.3)	20/3815 (0.5)	29/7036 (0.4)
506	minor	Time of anticoagulation reversal commence	Not collected or recorded	16/3221 (0.5)	31/3815 (0.8)	47/7036 (0.7)
507	minor	Time of intravenous infusion commence	Not collected or recorded	18/3221 (0.6)	33/3815 (0.9)	51/7036 (0.7)
508	minor	INR at (+/- 30 min) 3 hours after treatment of reversal	Not collected or recorded	18/3221 (0.6)	33/3815 (0.9)	51/7036 (0.7)
509	minor	INR at (+/- 30 min) 24 hours after treatment of reversal	Not collected or recorded	16/3221 (0.8)	32/3815 (0.8)	48/7036 (0.7)
510	minor	Repeat brain imaging undertaken	Not undertaken or collected	750/3221 (23.3)	1037/3815 (27.2)	1787/7036 (25.4)
511	minor	Date of repeat brain imaging undertaken	Not collected or recorded	0/2471 (0.0)	0/2778 (0.0)	0/5249 (0.0) [b]
512	minor	Time of repeat brain imaging undertaken	Not collected or recorded	0/2471 (0.0)	0/2778 (0.0)	0/5249 (0.0) [b]
513	minor	Hematoma volume not measured	Not collected or recorded	0/2471 (0.0)	0/2778 (0.0)	0/5249 (0.0) [b]
600	minor	BP recorded at set intervals when patient in both groups	Not collected or recorded	0/3221 (0.0)	41/3815 (1.1)	41/7036 (0.6) [c]
601	minor	Baseline BP time	Not collected or recorded	1/3221 (0.0)	44/3815 (1.2)	45/7036 (0.6)
604	minor	Date of intravenous antihypertensive	Not collected or recorded	0/3221 (0.0)	0/3815 (0.0)	0/5473 (0.0) [d]
605	minor	Time of intravenous antihypertensive	Not collected or recorded	1/2656 (0.0)	9/2817 (0.2)	10/5473 (0.2) [d]
606	minor	Time of intravenous antihypertensive treatment started during the first 24 hours	Not collected or recorded	0/2656 (0.0)	0/2817 (0.0)	0/5473 (0.0) [d]
607	minor	IV antihypertensive medications used	Not collected or recorded	0/2656 (0.0)	0/2817 (0.0)	0/5473 (0.0) [d]
608	minor	IV antihypertensive treatment stopped during the first 24 hours	Not collected or recorded	0/2656 (0.0)	0/2817 (0.0)	0/5473 (0.0) [d]
609	minor	Reason for stopping intravenous antihypertensive treatment	Not collected or recorded	0/2656 (0.0)	0/2817 (0.0)	0/5473 (0.0) [d]
610	minor	2-7 day BP measurement	Not collected or recorded	66/3221 (2.1)	93/3815 (2.4)	159/7036 (2.3) [c]
700	minor	BGL recorded at set intervals when patient in both groups	Not collected or recorded	272/1162 (23.4)	529/1393 (38.0)	801/2555 (31.4) [e]
	minor	Temperature recorded at set intervals when patient in both groups	Not collected or recorded	2/3221 (0.1)	79/3815 (2.1)	81/7036 (1.2)
703	minor	Temperature source	Not collected or recorded	1/3221 (0.0)	6/3815 (1.2)	7/7036 (0.1)
706	minor	Blood glucose and temperature recorded every (+/- 15 min) 4 hours in day2-3	Not collected or recorded	322/1162 (27.7)	521/1393 (37.4)	843/2555 (33.0) [e]

	minor	Temperature recorded every (+/- 15 min) 4 hours in day2-3	Not collected or recorded	327/3221 (10.2)	614/3815 (16.1)	941/7036 (13.4)
800	minor	Date of assessment	Not collected/ recorded	19/3221 (0.6)	38/3815 (1.0)	57/7036 (0.8)
801	minor	Is the patient alive	Not collected/ recorded	19/3221 (0.6)	37/3815 (1.0)	56/7036 (0.8)
802	minor	Other SAEs	Not collected or recorded	0/3119 (0.0)	3/3686 (0.1)	3/6805 (0.0) [f]
803	minor	Whether patient being discharged or transferred	Not collected or recorded	0/3119 (0.0)	4/3686 (0.1)	4/6805 (0.0) [f]
804	minor	Discharged or transferred location	Not collected or recorded	0/3221 (0.0)	2/3815 (0.1)	2/7036 (0.0)
805	minor	Date of discharged or transferred	Not collected or recorded	0/3221 (0.0)	3/3815 (0.1)	3/7036 (0.0)
806	minor	NIHSS score at day 7(or discharge if earlier)	Not collected or recorded	185/3221 (5.7)	267/3815 (7.0)	452/7036 (6.4)
807	minor	mRS score at day 7 (or discharge/death if earlier)	Not collected or recorded	31/3221 (1.0)	134 /3815 (3.5)	165/7036 (2.4)
808	minor	Hematoma volume within 7 days	Not collected or recorded	544/3221 (16.9)	492/3815 (12.9)	1036/7036 (14.7)
809	MAJOR	Care bundle during Day 2 to Day 7	Not collected or recorded	33/3221 (1.0)	40/3815 (1.1)	73/7036 (1.0) [a]
810	minor	Medication prior to death, discharge or 7 day follow-up	Not collected or recorded	27/3221 (0.8)	40/3815 (1.1)	67/7036(1.0) [a]
811	minor	Management prior to death, discharge or 7 day follow-up	Not collected or recorded	1472/3221 (45.7)	1896/3815 (49.7)	3368/7036 (47.9) [a]
812	MAJOR	Final diagnosis	Not collected or recorded	165/3221 (5.1)	202/3815 (5.3)	367/7036 (5.2)
813	minor	Clinicians aspects	Not collected or recorded	119/3221 (3.7)	146/3815 (3.8)	265/7036 (3.8) [a]
900	minor	Date of assessment	Date of assessment outside window (+/-200 days)	1116/3221 (34.7)	953/3815 (25.0)	2069/7064 (29.4)
902	MAJOR	mRS score @ 6 months	Not collected or recorded	334/3221 (10.4)	611/3815 (16.0)	945/7036 (13.4)
1001	MAJOR	SAE	Not reported within timeframe	1/3221 (0.0)	3/3815 (0.1)	4/7036 (0.1)
1004	MAJOR	SAE Event onset date	Not collected or recorded	1/3221 (0.0)	0/3815 (0.0)	5/7036 (0.0)
1105	minor	Miscellaneous	Miscellaneous	1/3221 (0.0)	1/3815 (0.0)	1/7036 (0.0)
1108	MAJOR	Patient lost to follow up	Patient lost to follow up, i.e. a patient whose survival status is unknown at 6 months (Form G)	222/3221 (6.9)	374/3815 (9.8)	596/7036 (8.5)

Data are n/N (%)

*BP denotes blood pressure, BGL blood glucose level, CT computered tomography, ICH intracerebral haemorrhage, INR international normalised ratio, IV intravenous, mRS modified Rankin scale, NIHSS National Institutes of Health Stroke Scale, PISCF patient information sheet and consent form, PR patient responsible, SAE serious adverse event

12. Sample size calculations

The study was designed with 90 power (p=0.05) to detect a 20 reduction in the odds (common odds ratio of 0.80) of a worse outcome using an ordinal logistic regression. Assuming a distribution in scores on the mRS in the usual care arm that is similar to those observed in the standard BP arm of the INTERACT2 trial - i.e. 7.6, 18.0, 18.8, 16.6, 19.0, 8.0 and 12.0 for scores of 0 to 6, respectively - this corresponds to a 5.6 absolute improvement in the proportion of patients experiencing a bad outcome (mRS of 3-6), from 55.6 down to 50. This also translates to a 10 relative risk reduction (relative risk of 0.90). This intervention assumes a greater treatment effect from the intensive care bundle over use of BP lowering alone (in INTERACT2, treatment effect was 4 absolute).

We anticipate recruiting a minimum of 110 sites in a stepped-wedge design consisting of 3 groups and 4 phases. Each group would therefore include approximately 36-37 sites. We assume an interclass correlation coefficient (ICC) of 0.044 between sites which is similar to that found in the INTERACT2, and the Head Position in Acute Stroke Trial (HeadPoST) across Chinese sites. To demonstrate a treatment effect with 90 power and a 2-sided type-I error rate of 5, each site would need to recruit an average of 18 patients per phase for a total sample size of 7,920 patients. Accounting for 5 of patients will have a missing outcome, each site would need to target an average of 19 patients per phase.

To allow for variability in the number of patients recruited at each site, with very large hospitals expected to recruit up to 50 patients per phase and smaller hospitals recruiting as little as 1 patient per phase, we derived an inflation factor using a conservative formula applicable to parallel cluster trials. Using this approach, the sample size would need to be inflated by a factor of approximately 1.3; thus leading to a sample size of up to 25 patients per site per phase (11,000 patients in total). Given that this is a very conservative scenario and that the effect of variability in cluster sizes is expected to be mostly mitigated by the stratification by size, we plan to target an average of 19 patients per site per phase for a total sample size of 8,360 patients. Assuming the worst case scenario for the effect of cluster size variability on power, this sample size would still provide at least 80 power to determine the proposed treatment effect.

13. Terms of reference of the Data and Safety Monitoring Board (DSMB)

The DSMB was responsible for: safeguarding the interests of trial participants; reviewing the research protocols and plans for data and safety monitoring; reviewing data monitoring reports provided by the study statistician; reviewing the progress of the study and monitoring adherence to the protocol, participant recruitment, outcomes, data quality, complications, and other issues related to participant safety; monitoring the assumptions underlying sample size calculations for the study (Steering Committee and Operations Committee were kept blinded) and to alert the investigators if they saw substantial departures as the data accumulated; ensuring that the study data and results of monitoring were kept confidential; assessing the safety and efficacy of the interventions; monitoring the overall conduct of the trial; providing recommendations about stopping or continuing the trial to the trial Steering Committee; contributing to enhancing the integrity of the trial; formulating recommendations in relation to the selection, recruitment, or retention of participants, or their management, or to improving adherence to protocol-specified regimens and retention of participants, and the procedures for data management and quality control.

The DSMB was advisory to the Steering Committee. The Steering Committee was responsible for promptly reviewing the DSMB recommendations, to deciding as to whether to continue or terminate the trial, and to determine whether amendments to the protocol or changes in study conduct were required.

The DSMB conducted both periodical safety reviews and formal interim analyses as follows:

- The first analysis meeting of the DSMB was planned and undertaken within 6 months after the first patient was enrolled to review initial safety data and finalise the format of reports.
- Subsequent safety review meetings were conducted approximately every 6-12 months, with regular safety reviews undertaken without formal testing of the efficacy data.
- Two 'Formal Interim Analysis' meetings were planned and undertaken, but the implications of the study design was that the data were unbalanced, with more patients in the control group than intervention group. The purpose of these analyses was to review data relating to treatment efficacy and quality of trial conduct in conjunction with safety data. All these reviews were held by teleconference.
- The DSMB was allowed to respond to specific requests made by the INTERACT3 Steering Committee, but none were made.

The trial Principal Investigator and other members of the Trial Operations Committee attended open sessions at the beginning of meetings and were available at the end of meetings to answer any urgent questions. The date of each DSMB meeting was made available to the unblinded statisticians with at least 6 weeks notice. The unblinded statisticians prepared the DSMB reports and attended the whole meeting to assist in interpretating the results.

For each DSMB meeting, both safety reviews and interim analysis; open and closed reports were provided. Open reports, available to all who attended the DSMB meeting, included data on recruitment; baseline characteristics (pooled by treatment regimen/phase); pooled data on eligibility violations; completeness of follow-up and compliance; major protocol changes; study accrual by month and by site, overall and by each component of the care bundle and including an assessment of whether recruitment targets were being met and whether enough randomised sites were recruiting patients; time between presentation and achievement of targets of goal-directed BP, blood glucose, body temperature and INR, respectively, by intervention group; the rate of achieving within in 1 hour after treatment the targets of goal-directed BP, blood glucose, body temperature and INR, respectively, by intervention group; any other relevant information (such as updated Cochrane reviews); pooled analysis of primary outcome; details of the number of pending and missing case report forms (including the number of seriously overdue follow-ups), and the number with outstanding data items. Closed reports, available only to those attending the closed sessions of the DSMB meeting, included analyses of primary and secondary efficacy endpoints, subgroup and baseline-adjusted analyses, analyses of serious adverse events, and open report analyses that are displayed by intervention group. The reports for the safety reviews were a subset of the reports prepared for the formal interim analysis.

The unblinded statistician(s) from The George Institute for Global Health prepared both the open and closed reports. The open and closed reports provided information that was accurate, with follow-up that was complete to within approximately 1 month of the date of the DSMB meeting. The reports were provided to DSMB members 2 weeks prior to the date of meetings.

During the period of recruitment into the study, interim analyses of the proportion of patients alive and independent, or dead (at hospital discharge and at 6 months), or with other major outcome events were supplied, in strictest confidence, to all members of the DSMB, along with any other analyses that the DSMB may have requested. In the light of these analyses, the DSMB was charged with advising the Principal Investigator if, in their view, the randomised comparisons had provided both:

- 'proof beyond reasonable doubt' that for all, or some, a treatment was clearly indicated or clearly contra-indicated, and
- evidence that might reasonably be expected to lead many clinicians conversant with the available evidence to materially change their practice regarding components of the goal-directed care bundle of active management strategies in patients with acute intracerebral haemorrhage.

A recommendation to discontinue INTERACT3 prematurely was to be based upon there being clear evidence that the treatment provides protection or caused harm for an important clinical outcome. The final recommendation to the Steering Committee was at the discretion of the DSMB but based upon agreed standards for the interpretation of interim analysis in clinical trials. The Steering Committee had responsibility for evaluating and implementing the recommendations provided by the DSMB.

A recommendation to modify INTERACT3 was accompanied by the maximum possible information that the DSMB could provide to the Steering Committee without affecting the integrity of the trial. Once again, the Steering Committee was responsibility for evaluating and implementing any recommendations as they considered appropriate. If additional expert opinion was to have been sought or additional analyses are required prior to making a recommendation, the DSMB will work to schedule another meeting at the earliest possible opportunity; this was not actioned.

The decision to stop the trial temporarily or indefinitely was considered in hand with ensuring safety for the trial participants and the impact premature termination will have on clinical practice. The Haybittle-Peto rule was used as a guide for ‘proof beyond reasonable doubt’ in the monitoring of both efficacy and safety information in the trial. The trial proceeded as planned.

The DSMB worked on the principle that a difference of at least 3 standard errors in an interim analysis of a major outcome event (e.g. death from all causes or independent survival at 6 months) between patients allocated to the intensive or the control group, would justify halting, or modifying the study before the planned completion of recruitment. This criterion (‘Peto rule’) has the practical advantage that the exact number of interim analyses is of less importance, and therefore no fixed schedule was proposed.

Following each DSMB meeting, a recommendation was made to the Steering Committee with the following options in the DSMB statement:

- modification of the study;
- termination of the study early on the basis of a clear and substantial evidence of benefit or the data suggests the risk of adverse events substantially outweighs the potential benefits;
- continue the study unchanged;
- additional expert review after which a recommendation were made.

14. Assessments of functional outcome and health-related quality of life

The primary outcome was functional outcome or recovery according to a shift in the range of scores on the modified Rankin scale (mRS), a global seven-level measure of functioning where scores 0 to 1 indicate a good outcome with minimal or no neurological symptoms, scores 2 to 5 indicate a poor outcome with increasing degree of disability, and death is 6. systematically. For the assessment of health-related quality of life (HRQoL), the EQ-5D-3L questionnaire was used, as assessed directly by a patient or by a proxy-responder. The descriptive system of the EQ-5D defines the state of general health across five dimensions (mobility, self-care, usual activities, pain/discomfort, and anxiety/depression) with three levels (no problems, some/moderate problems, and severe problems). The EQ-5D-3L utility score integrates the ratings of the 5 dimensions into a single score, calculated by using population-based preference weights for each subscale. If patients were unable to answer the questionnaire themselves, proxy-responders (i.e. their caregiver or doctor), were asked to rate the patient's HRQoL. The protocol did not stipulate specifically the process of proxy-responder selection; the decision was opportunistic that arose during a telephone (or face-to-face) interview between the responsible neurologically competent person (blinded to treatment arm) and the patient or caregiver at the scheduled time of follow-up. The trained staff were independent and blinded to the care to which patients received and other clinical information. Trained outcome assessors used an assigned telephone script. They were managed as part of the RCC in each country. The follow-up assessments were generally conducted by RCC research staff, but for practical reasons, a blinded assessor at each site could undertake the assessment of outcome. The final process of outcome assessment is outlined below

**Country**	**Type of follow-up**
Brazil	Central
China	Central
Chile	Central
Sri Lanka	Central
India	Per site
Pakistan	Per site
Nigeria	Per site
Vietnam	Per site
Mexico	Per site
Peru	Per site

15. Tables

Table S1. Patient enrolment by site over time from December 2017 to December 2021 (included in the modified intention-to-treat population)*

			Cluster assignment		Total number of patients		Patients randomised by phase†				
Country	Province/City	ID	Batch	Group	Screened	Randomised	Period 1	Period 2	Period 3	Period 4	Comments
China	Sichuan	1002	1	1	176	162	30	21	16	95	
China	Sichuan	1018	1	1	88	70	6	16	13	35	
China	Hebei	1106	1	1	94	81	18	32	14	17	
China	Hebei	1108	1	1	41	25	12	5		8	
China	Liaoning	1201	1	1	210	205	53	79	73		
China	Zhejiang	1310	1	1	32	31	6	7		18	
China	Shandong	1317	1	1	153	142	22	43	34	43	
China	Shandong	1319	1	1	116	81	20	13	20	28	
China	Henan	1403	1	1	155	80	23	13	17	27	
China	Guangdong	1501	1	1	72	38	11	6	4	17	
China	Sichuan	1001	1	2	679	126	38	36	18	34	
China	Sichuan	1015	1	2	127	118	35	23	39	21	
China	Sichuan	1023	1	2	104	84	25	15	13	31	
China	Sichuan	1024	1	2	101	80	23	17	14	26	
China	Tianjing	1103	1	2	206	80	23	18	28	11	
China	Shanxi	1109	1	2	209	160	50	31	47	32	
China	Neimenggu	1113	1	2	46	36	7	13	1	15	
China	Neimenggu	1114	1	2	89	78	7	34	14	23	
China	Shandong	1318	1	2	149	93	53	40			
China	Sichuan	1008	1	3	430	200	61	51	44	44	
China	Sichuan	1013	1	3	133	120	41	19	31	29	
China	Sichuan	1014	1	3	165	121	30	31	29	31	
China	Sichuan	1019	1	3	191	160	67	40	13	40	
China	Sichuan	1020	1	3	147	80	19	18	22	21	

China	Beijing	1102	1	3		43	28		15	13			
China	Hebei	1107	1	3		55	44		44				
China	Henan	1404	1	3		68	31		11	7	5	8	
China	Shaanxi	1601	1	3		202	162		48	45	29	40	
China	Sichuan	1007	2	1		224	165		40	53	51	21	
China	Sichuan	1009	2	1		162	153		12	45	32	64	
China	Sichuan	1011	2	1		47	33		10	3	3	17	
China	Sichuan	1012	2	1		43	41		31	10			
China	Sichuan	1016	2	1		66	56		18	26	12		
China	Sichuan	1028	2	1		117	80		40	40			
China	Sichuan	1032	2	1		84	80		20	1	6	53	
China	Shanxi	1112	2	1		27	15		10	2	1	2	
China	Shanghai	1301	2	1		94	88		17	39	32		
China	Sichuan	1017	2	2		138	120		42	14	28	36	
China	Sichuan	1021	2	2		146	146		48	29	49	20	
China	Jiangsu	1303	2	2		92	67		27	15	8	17	
China	Jiangsu	1306	2	2		415	159		60	18	47	34	
China	Zhejiang	1309	2	2		125	112		33	28	6	45	
China	Fujian	1311	2	2		145	40		13	7	10	10	
China	Sichuan	1003	2	3		33	32		17	8	7		
China	Sichuan	1005	2	3		259	224		43	17	89	75	
China	Sichuan	1006	2	3		138	113		79	25		9	
China	Sichuan	1026	2	3		111	39		15	5	8	11	
China	Shanxi	1111	2	3		124	53		53				
China	Jiangsu	1304	2	3		5	5		5				
China	Shanghai	1322	2	3		7	7		4	3			
China	Jiangsu	1324	2	3		206	200		42	52	56	50	
China	Henan	1401	2	3		14	12		9	3			
China	Hubei	1406	2	3		124	116		46	34	5	31	

China	Sichuan	1030	3	1		112	84		19	20	18	27	
China	Sichuan	1039	3	1		114	112		24	20	54	14	
China	Sichuan	1044	3	1		28	24		7	6	8	3	
China	Hebei	1115	3	1		23	19		19				
China	Jiangsu	1326	3	1		55	55		40	15			
China	Jiangsu	1330	3	1		71	29		13	12	4		
China	Shaanxi	1602	3	1		12	12		12				
China	Xinjiang	1603	3	1		21	20		11		9		
China	Sichuan	1035	3	2		80	50		47	1		2	
China	Sichuan	1036	3	2		72	37		24	13			
China	Sichuan	1038	3	2		10	7		4	3			
China	Sichuan	1043	3	2		11	11		7	4			
China	Sichuan	1045	3	2		154	142		36	49	45	12	
China	Shandong	1314	3	2		82	73		16	24	12	21	
China	Hubei	1405	3	2		110	31		19	12			
China	Hubei	1409	3	2		126	84		26	21	37		
China	Sichuan	1034	3	3		24	22		16	2	4		
China	Sichuan	1040	3	3		3	1		1				
China	Sichuan	1042	3	3		101	91		20	37	20	14	
China	Tianjing	1105	3	3		277	120		95	25			
China	Shandong	1316	3	3		11	7		6	1			
China	Jiangsu	1327	3	3		88	73		31	25		17	
China	Hubei	1410	3	3		115	109		56	34		19	
China	Sichuan	1046	4	1		15	15		7	8			
China	Hubei	1411	4	1		62	26		13	9	4		
China	Liaoning	1203	4	2		20	19		11	2	1	5	
China	Liaoning	1204	4	2		7	7		4	3			
China	Liaoning	1205	4	3		166	159		22	18	21	98	
China	Hubei	1412	4	3		85	45		5	16		24	

Chile	Santiago	2001	4	1		25	8		2			6	
Chile	Chillan	2006	4	1		74	15		6	9			
Chile	Región Metropolitana	2008	4	1		22	4		3		1		
Chile	Los Angeles	2004	4	2		12	4		1	3			
Chile	La Florida	2009	4	2		40	4		1	1	2		
Chile	Valparaiso	2002	4	3		20	6		2			4	
Chile	Los Lagos	2005	4	3		21	7		3	2		2	
Peru	Lima	3001	4	2		39	12		6		6		
Vietnam	Ho Chi Minh City	4004	4	1		11	3		3				
Vietnam	Ho Chi Minh City	4006	4	1		42	11		6	4		1	
Vietnam	Hanoi	4014	4	1		104	95		29	21	14	31	
Vietnam	Ho Chi Minh City	4016	4	2		44	16		5	5	2	4	
Vietnam	Ho Chi Minh City	4002	4	3		37	10		6	2	2		
Vietnam	Ha Dong	4015	4	3		60	20		11	1		8	
Pakistan	Islamabad	5003	4	1		28	12		3	1	5	3	
Pakistan	Karachi	5001	4	3		90	36		3	8	9	16	
Pakistan	Karachi	5004	4	3		1	1		1				
Mexico	Mexico City	6001	4	1		59	9		1			8	
Brazil	Joinville	7002	4	1		39	6		2			4	
Brazil	Porto Alegre	7003	4	2		5	5		1			4	
Brazil	Porto Alegre	7006	4	2		2	1			1			
Brazil	Riberao Pretto	7001	4	3		111	23		5	5	6	7	
Brazil	Botucatu	7008	4	3		7	1					1	
Nigeria	Lagos	8003	4	1		77	26		9	8	2	7	
Nigeria	Zaria, Nigerila	8002	4	2		30	29		5		3	21	
Nigeria	Ilorin	8001	4	3		34	15		3		2	10	
India	Faridkot	9101	4	1		7	2			2			
India	Dispur	9107	4	1		23	12			12			
India	Tezpur	9102	4	2		14	5		3	2			

India	Belgaum	9106	4	2		33	28		8	2	7	11	
India	Thiruvananthapuram	9108	4	3		18	16			1	1	14	
India	Delhi	9110	4	3		5	4				1	3	
Sri Lanka	Kandy	9204	4	1		9	8		1		1	6	
Sri Lanka	Galle	9206	4	1		40	11		6		2	3	
Sri Lanka	Colombo	9202	4	2		21	16		10	3	1	2	
Sri Lanka	Kurunegala	9203	4	2		127	103		15	13	21	54	
Sri Lanka	Jaffna	9205	4	2		36	22		5	2	4	11	
Sri Lanka	Colombo	9201	4	3		70	50		18	14	7	11	
Sri Lanka	Gampaha	9207	4	3		27	24		2	9	3	10	

[*]orange = control ‘usual care’; blue = intervention ‘care bundle’

[†]One site in China was excluded after 28 patients were enrolled as a necessary regulatory approval was unable to be obtained

Table S2. Patient enrolment by strata for sites in the modified intention-to-treat population

			Randomised patients during follow-up						
**Sequence**	**Sites enrolled (n)**	**Proposed size (n)**	**Total**	**Control**	**Intervention**	**Period 1**	**Period 2**	**Period 3**	**Period 4**
1	43	3631	2244	635	1609	635	601	450	558
2	37	3256	2205	1240	965	738	502	463	502
3	41	3855	2587	1940	647	955	571	414	647
Total	121	10742	7036	3815	3221	2328	1674	1327	1707

Table S3. Definition of calendar time windows for time trends

Calendar date	Month	3-month interval	6-month interval
Dec 2017 to Feb 2018	1-3	1	1
Mar 2018 to May 2018	4-6	2	1
Jun 2018 to Aug 2018	7-9	3	2
Sep 2018 to Nov 2018	10-12	4	2
Dec 2018 to Feb 2019	13-15	5	3
Mar 2019 to May 2019	16-18	6	3
Jun 2019 to Aug 2019	19-21	7	4
Sep 2019 to Nov2019	22-24	8	4
Dec 2019 to Feb 2020	25-27	9	5
Mar 2020 to May 2020	28-30	10	5
Jun 2020 to Aug 2020	31-33	11	6
Sep 2020 to Nov 2020	34-36	12	6
Dec 2020 to Feb 2021	37-39	13	7
Mar 2021 to May 2021	40-42	14	7
Jun 2021 to Aug 2021	43-45	15	8
Sep 2021 to Nov 2021	46-48	16	8
Dec 2021	49	17	8

Table S4. Reasons that patients with acute intracerebral haemorrhage were excluded from participating in the trial

**Reason**	**Frequency**
Presentation >6 h from the onset of symptoms	2419 (63.3%)
No consent	659 (17.2%)
Structural cause for the intracerebral haemorrhage	436 (11.4%)
Intracerebral haemorrhage secondary to trauma	250 (6.5%)
Age <18 years	32 (0.8%)
Other reason	25 (0.7%)
**Total**	**3821**

Table S5. Number of patients enrolled and with primary outcome data per group and per period, in the modified intention-to-treat population*

Sequence	Period 1	Period 2	Period 3	Period 4	Total
1 (43 sites)	558/635 (87.9)	524/601 (87.2)	391/450 (86.9)	501/558 (89.8)	1974/2244 (88.0)
2 (37 sites)	640/738 (86.7)	422/502 (84.1)	426/463 (92.0)	456/502 (90.8)	1944/2205 (88.2)
3 (41 sites)	825/955 (86.4)	525/571 (91.9)	393/414 (94.9)	594/647 (91.8)	2337/2587 (90.3)
Total (121 sites)	2023/2328 (86.9)	1471/1674 (87.9)	1210/1327 (91.2)	1551/1707 (90.9)	6255/7036 (88.9)

*Data are n/N (%). The denominator represents the number of subjects enrolled during the period. The numerator represents the number of subjects with a primary endpoint measure (modified Rankin scale at 6 months). Cells shaded in grey represent ‘usual care’ periods. Cells shaded in red represent ‘care bundle’ periods.

Table S6. Method of assessment of 6 month outcomes

Assessment type	Care bundle (N=3221)	Usual care (N=3815)
Phone to caregiver	2470/3121 (79.1)	2913/3683 (79.1)
Phone to patient	247/3121 (7.9)	255/3683 (6.9)
Phone to patient's doctor or medical practitioner	115/3121 (3.7)	110/3683 (3.0)
Face to face	32/3121 (1.0)	6/3683 (0.2)
Other	33/3121 (1.1)	23/3683 (0.6)
Refused to receive follow-up assessment	11/3121 (0.4)	39/3683 (1.1)
Lost to follow-up	213/3121 (6.8)	337/3683 (9.2)

Data are n/N (%)

Table S7. Additional demography, medical history, and use of medications in patients with acute intracerebral haemorrhage

Variable	Care bundle (N=3221)	Usual care (N=3815)
Place of residency at time of admission		
Own home	2965/3220 (92.1)	3485/3813 (91.4)
Family members home	210/3220 (6.5)	224/3813 (5.9)
Institutional care facility	8/3220 (0.2)	22/3813 (0.6)
Other	37/3220 (1.1)	82/3813 (2.2)
Medical history		
Known coagulation/haematological disorder	4/3221 (0.1)	9/3813 (0.2)
History of epilepsy	21/3221 (0.7)	21/3813 (0.6)
History of liver disease	26/3221 (0.8)	47/3813 (1.2)
Other major condition limiting daily activity	128/3221 (4.0)	139/3814 (3.6)
Antihypertension drugs used	1446/3221 (44.9)	1626/3813 (42.6)
Type of antihypertension		
ACE-I or ARB	344/1446 (23.8)	424/1626 (26.1)
Diuretic	74/1446 (5.1)	77/1626 (4.7)
Calcium channel blocker	765/1446 (52.9)	936/1626 (57.6)
Beta-blocker	104/1446 (7.2)	117/1626 (7.2)
Other	326/1446 (22.5)	265/1626 (16.3)
Blood glucose lowering agents used	250/3221 (7.8)	263/3814 (6.9)
Type of glucose lowering agents		
Oral agents	205/250 (82.0)	211/263 (80.2)
Insulin	56/250 (22.4)	72/263 (27.4)
Nitrates used	21/3221 (0.7)	14/3814 (0.4)
Anticoagulation among patients with elevated INR	11/25 (44.0)	15/59 (25.4)
Vitamin K antagonist	10/11 (90.9)	14/15 (93.3)
New oral anticoagulant†	1/11 (9.1)	1/15 (6.7)

ACE-I indicates angiotensin converting enzyme inhibitor, ARB angiotensin receptor blocker

*Data are n/N (%)

†Rivaroxaban

Table S8. Details of secondary causes of intracerebral haemorrhage

Characteristic	Care bundle (N=3221)	Usual care (N=3815)
AVM	22/3197 (0.7)	46/3775 (1.2)
Aneurysm	20/3197 (0.6)	41/3775 (1.1)
Other vascular abnormality	15/3197 (0.5)	17/3775 (0.5)
Tumour	0/3197 (0.0)	9/3775 (0.2)
Systematic disease	2/3197 (0.1)	9/3775 (0.2)
Anticoagulation	6/3197 (0.2)	11/3775 (0.3)
Other definite pathology	16/3197 (0.5)	12/3775 (0.3)
Uncertain aetiology	52/3197 (1.6)	98/3775 (2.6)

AVM denotes arteriovenous malformation

Table S9. Intravenous blood pressure lowering treatments administered in the first 24 hours and achieved levels of blood pressure*

**Characteristics**	**Care bundle (N=3221)**	**Usual care (N=3815)**
Any IV blood pressure medication	2542/3221 (78.9%)	2703/3811 (70.9%)
IV medication used		
urapidil	1607/2656 (60.5)	1744/2817 (61.9)
nicardipine	225/2656 (8.5)	213/2817 (7.6)
sodium nitroprusside	582/2656 (21.9)	587/2817 (20.8)
labetalol	376/2656 (14.2)	287/2817 (10.2)
nimodipine	161/2656 (6.1)	271/2817 (9.6)
clevidipine	50/2656 (1.9)	50/2817 (1.8)
metoprolol	20/2656 (0.8)	19/2817 (0.7)
hydralazine	33/2656 (1.2)	24/2817 (0.9)
atenolol	0/2656 (0.0)	0/2817 (0.0)
enalapril	16/2656 (0.6)	17/2817 (0.6)
clonidine	1/2656 (0.0)	0/2817 (0.0)
nifedipine	8/2656 (0.3)	11/2817 (0.4)
nitroglycerin	198/2656 (7.5)	204/2817 (7.2)
prazosin	0/2656 (0.0)	0/2817 (0.0)
isosorbide dinitrate	0/2656 (0.0)	0/2817 (0.0)
frusemide	50/2656 (1.9)	49/2817 (1.7)
Other	18/2656 (0.7)	18/2817 (0.6)
Measure		
Highest systolic blood pressure		
n	3220	3810
Mean (SD)	180.8 (25.2)	182.0 (25.6)
Min Max	113 280	100 284
Lowest systolic blood pressure		
n	3214	3788
Mean (SD)	118.5 (14.4)	121.0 (16.7)
Min Max	52 200	58 201

Data are n/N (%)

*IV denotes intravenous

Table S10. Details of all blood pressure lowering treatments between days 2 and 7*

**Characteristics**	**Care bundle (N=3221)**	**Usual care (N=3815)**
**Intravenous BP lowering treatment**	2131/3188 (66.8%)	2277/3775 (60.3%)
**IV medication used**		
urapidil	1393/2287 (60.9)	1535/2392 (64.2)
nicardipine	191/2287 (8.4)	176/2392 (7.4)
sodium nitroprusside	574/2287 (25.1)	563/2392 (23.5)
labetalol	284/2287 (12.4)	223/2392 (9.3)
nimodipine	202/2287 (8.8)	257/2392 (10.7)
clevidipine	80/2287 (3.5)	91/2392 (3.8)
metoprolol	23/2287 (1.0)	24/2392 (1.0)
hydralazine	28/2287 (1.2)	26/2392 (1.1)
atenolol	0/2287 (0.0)	0/2392 (0.0)
enalapril	12/2287 (0.5)	14/2392 (0.6)
clonidine	3/2287 (0.1)	1/2392 (0.0)
nifedipine	18/2287 (0.8)	17/2392 (0.7)
nitroglycerin	190/2287 (8.3)	184/2392 (7.7)
prazosin	1/2287 (0.0)	3/2392 (0.1)
isosorbide dinitrate	0/2287 (0.0)	1/2392 (0.0)
frusemide	80/2287 (3.5)	87/2392 (3.6)
Other	16/2287 (0.7)	19/2392 (0.8)
**Any oral blood pressure medication taken**	2267/3188 (71.1)	2547/3775 (67.5)
**Oral/topical medication used**		
ACE-I / ARB	1320/2267 (58.2)	1154/2547 (45.3)
diuretic	327/2267 (14.4)	252/2547 (9.9)
calcium channel blocker	1740/2267 (76.8)	2106/2547 (82.7)
beta blocker	207/2267 (9.1)	255/2547 (10.0)
Other	109/2267 (4.8)	88/2547 (3.5)

Data are n/N (%)

*ACE-I indicates angiotensin converting enzyme inhibitor, ARB angiotensin receptor blocker, BP blood pressure, IV intravenous

Table S11. Management and care administered until day 7

Characteristics	Care bundle (N=3221)	Usual care (N=3815)
**Ward/department where patient stayed longest**		
Neurosurgery	2317/3197 (72.5)	2946/3776 (78.0)
Neurology	380/3197 (11.9)	336/3776 (8.9)
Intensive care	341/3197 (10.7)	317/3776 (8.4)
Emergency department	14/3197 (0.4)	14/3776 (0.4)
Other	145/3197 (4.5)	163/3776 (4.3)
**Decompressive surgery**	844/3196 (26.4)	1016/3775 (26.9)
Craniotomy	468/844 (55.5)	567/1016 (55.8)
Craniectomy	17/844 (2.0)	29/1016 (2.9)
Endoscopy	28/844 (3.3)	40/1016 (3.9)
Aspiration	109/844 (12.9)	89/1016 (8.8)
Intraventricular drainage	172/844 (20.4)	180/1016 (17.7)
Catheterisation with/without lysis	31/844 (3.7)	52/1016 (5.1)
Other type	19/844 (2.3)	59/1016 (5.8)
**Other management**		
Intubation	706/3196 (22.1)	956/3775 (25.3)
Mechanical ventilation	646/3196 (20.2)	806/3775 (21.4)
Assisted feeding	1749/3196 (54.7)	1920/3775 (50.9)
Intensive care unit admission	1105/3196 (34.6)	1435/3775 (38.0)
Acute stroke unit admission	1159/3196 (36.3)	801/3775 (21.2)
Pneumatic calf compression	887/3196 (27.8)	786/3775 (20.8)
Intravenous traditional Chinese medicine	367/3196 (11.5)	415/3774 (11.0)
Intravenous neuroprotective agent	1784/3196 (55.8)	2215/3775 (58.7)
Physiotherapy	1103/3196 (34.5)	874/3775 (23.2)
Occupational therapy	549/3196 (17.2)	398/3775 (10.5)
Urinary catheter insertion	1862/3196 (58.3)	2248/3775 (59.5)
Anti-epilepsy drugs	817/3196 (25.6)	807/3775 (21.4)
Intravenous mannitol in first 24 h	504/2656 (19.0)	514/2817 (18.2)
Intravenous mannitol in days 2-7	632/2287 (27.6)	559/2392 (23.4)
Dexamethasone	124/3194 (3.9)	210/3775 (5.6)
Statins	223/3194 (7.0)	225/3775 (6.0)
Clinical decision to withdraw ‘active’ care	21/3196 (0.7)	26/3775 (0.7)

Data are n/N (%)

Table S12. Descriptive analysis of clinical outcomes

Characteristics	Care bundle (N=3221)	Usual care (N=3815)	Total (7036)
**mRS at 6 months**			
0 – no symptoms	247/2892 (8.5)	342/3363 (10.2)	589/6255 (9.4)
1 – no significant disability	907/2892 (31.4)	896/3363 (26.6)	1803/6255 (28.8)
2 – slight disability	185/2892 (6.4)	198/3363 (5.9)	383/6255 (6.1)
3- moderate disability	549/2892 (19.0)	571/3363 (17.0)	1120/6255 (17.9)
4- moderate-severe disability	340/2892 (11.8)	444/3363 (13.2)	784/6255 (12.5)
5- severe disability	257/2892 (8.9)	341/3363 (10.1)	598/6255 (9.6)
6- death	407/2892 (14.1)	571/3363 (17.0)	978/6255 (15.6)
**NIHSS score at day 7**			
<5	722/3136 (23.0)	936/3673 (25.5)	1658/6809 (24.4)
5-9	717/3136 (22.9)	721/3673 (19.6)	1438/6809 (21.1)
10-14	587/3136 (18.7)	664/3673 (18.1)	1251/6809 (18.4)
15-19	311/3136 (9.9)	417/3673 (11.4)	728/6809 (10.7)
20-24	206/3136 (6.6)	217/3673 (5.9)	423/6809 (6.2)
≥25	492/3136 (15.7)	592/3673 (16.1)	1084/6809 (15.9)
Death	101/3136 (3.2)	126/3673 (3.4)	227/6809 (3.3)
**NIHSS score at day 7 (continuous measure)**			
n	3035	3547	6582
Mean (SD)	12.9 (10.7)	13.0 (11.1)	13.0 (10.90)
Median (IQR)	10.0 (5.0, 18.0)	10.0 (4.0, 18.0)	10.0 (4.0; 18.0)
**Death or disability at 6 months**	1553/2892 (53.7)	1927/3363 (57.3)	3480/6255 (55.6)
**Death at 6 months**	407/2999 (13.6)	571/3441 (16.6)	978/6440 (15.2)
**Disability (3-5) in survivors at 6 months**	1146/2485 (46.1)	1356/2792 (48.6)	2502/5277 (47.4)
**EQ-5D-3L mobility (ordinal)**			
1= no problems in walking	1656/2485 (66.6)	1773/2792 (63.5)	3429/5277 (65.0)
2= some problems in walking	516/2485 (20.8)	586/2792 (21.0)	1102/5277 (20.9)

3= confined to bed	313/2485 (12.6)	433/2792 (15.5)	746/5277 (14.1)
**EQ-5D-3L self-care (ordinal)**			
1= no problems with self-care	1424/2485 (57.3)	1509/2792 (54.0)	2933/5277 (55.6)
2= some problems washing or dressing	541/2485 (21.8)	625/2792 (22.4)	1166/5277 (22.1)
3= unable to wash or dress	520/2485 (20.9)	658/2792 (23.6)	1178/5277 (22.3)
**EQ-5D-3L usual activities (ordinal)**			
1= no problems	1058/2485 (42.6)	1095/2792 (39.2)	2153/5277 (40.8)
2= some problems	759/2485 (30.5)	838/2792 (30.0)	1597/5277 (30.3)
3= unable to perform	668/2485 (26.9)	859/2792 (30.8)	1527/5277 (28.9)
**EQ-5D-3L pain/discomfort (ordinal)**			
1= no pain/discomfort	1768/2485 (71.1)	1916/2792 (68.6)	3684/5277 (69.8)
2= moderate pain/discomfort	704/2485 (28.3)	843/2792 (30.2)	1547/5277 (29.3)
3= extreme pain/discomfort	13/2485 (0.5)	33/2792 (1.2)	46/5277 (0.9)
**EQ-5D-3L anxiety/depression (ordinal)**			
1= not anxious or depressed	1967/2485 (79.2)	2127/2792 (76.2)	4094/5277 (77.6)
2= moderately anxious or depressed	498/2485 (20.0)	609/2792 (21.8)	1107/5277 (21.0)
3= I am extremely anxious or depressed	20/2485 (0.8)	56/2792 (2.0)	76/5277 (1.4)
**Overall health EQ-5D-3L visual analogue scale at 6 months**			
n	2485	2791	5276
Mean (SD)	68.3 (20.9)	67.3 (20.9)	67.8 (20.9)
Median (Q1; Q3)	70 (60; 80)	70 (50; 80)	70 (50; 80)
**EQ-5D-3L Utility Score* at 6 months**			
n	2892	3363	6255
Mean (SD)	0.620 (0.3642)	0.577 (0.3737)	0.597 (0.3699)
Median (Q1; Q3)	0.775 (0.292; 1.000)	0.683 (0.292; 0.887)	0.702 (0.292; 1.000)
**Hospital discharge by day 7***			
No	2899/3215 (90.2%)	3462/3801 (91.1%)	6361/7016 (90.7%)
Yes	316/3215 (9.8%)	339/3801 (8.9%)	655/7016 (9.3%)
**Place of residence at 6 months**			

Own home - independent	627/2485 (25.2)	677/2792 (24.2)	1304/5277 (24.7%)
Own home – with assistance	960/2485 (38.6)	1140/2792 (40.8)	2100/5277 (39.8%)
Family member's home	795/2485 (32.0)	842/2792 (30.2)	1637/5277 (31.0%)
Hospital	40/2485 (1.6)	69/2792 (2.5)	109/5277 (2.1%)
Care facility (eg nursing home, hostel, shelter, other)	60/2485 (2.4%)	64/2792 (2.3%)	124/5277 (2.3%)
Other	3/2485 (0.1%)	0/2792 (0.0%)	3/5277 (0.1%)

EQ-5D-3L denotes EuroGol quality of life instrument, mRS modified Rankin scale, NIHSS National Institutes of Health Stroke Scale

*Utility score calculated using China weight for Chinese and UK weight for other countries outside China. Score 0 assigned to deceased patients.

Table S13. Multiple imputation for primary outcome

				Primary model*		Secondary model†	
**Outcomes**	**Care bundle (n=3221)**	**Usual care (n=3815)**	**Total (N=7036)**	**OR (95% CI)**	**p value**	**OR (95% CI)**	**p value**
**Imputed mRS score at 6 months (MAR)**				0.86 (0.76 to 0.97)	0.017	0.85 (0.74 to 0.98)	0.028
0 - no symptoms	274 (8.5)	379 (9.9)	653 (9.3)				
1 - no significant disability	994 (30.9)	1012 (26.5)	2006 (28.5)				
2 - slight disability	205 (6.4)	224 (5.9)	428 (6.1)				
3 - moderate disability	610 (18.9)	652 (17.1)	1262 (17.9)				
4 - moderate severe disability	388 (12.1)	505 (13.2)	893 (12.7)				
5 - severe disability	295 (9.2)	392 (10.3)	687 (9.8)				
6 - death	455 (14.1)	651 (17.1)	1105 (15.7)				
**Imputed mRS score at 6 months (+1)**				0.88 (0.78 to 0.99)	0.034	0.86 (0.75 to 0.99)	0.033
0 - no symptoms	247 (7.7)	342 (9.0)	589 (8.4)				
1 - no significant disability	934 (29.0)	934 (24.5)	1867 (26.5)				
2 - slight disability	272 (8.4)	314 (8.2)	586 (8.3)				
3 - moderate disability	569 (17.7)	597 (15.6)	1165 (16.6)				
4 - moderate severe disability	401 (12.5)	525 (13.8)	926 (13.2)				
5 - severe disability	305 (9.5)	402 (10.5)	707 (10.1)				
6 - death	493 (15.3)	702 (18.4)	1195 (17.0)				
**Imputed mRS score at 6 months (Death)**				0.92 (0.81 to 1.03)	0.15	0.89 (0.78 to 1.02)	0.082
0 - no symptoms	247 (7.7)	342 (9.0)	589 (8.4)				
1 - no significant disability	907 (28.2)	896 (23.5)	1803 (25.6)				
2 - slight disability	185 (5.7)	198 (5.2)	383 (5.4)				
3 - moderate disability	549 (17.0)	571 (15.0)	1120 (15.9)				
4 - moderate severe disability	340 (10.6)	444 (11.6)	784 (11.1)				

				Primary model*		Secondary model†	
Outcomes	Care bundle (n=3221)	Usual care (n=3815)	Total (N=7036)	OR (95% CI)	p value	OR (95% CI)	p value
5 - severe disability	257 (8.0)	341 (8.9)	598 (8.5)				
6 - death	736 (22.9)	1023 (26.8)	1759 (25.0)				

Data are n/N (%)

mRS denotes modified Rankin scale

Results obtained using 100 multiple imputations using fully conditional specification and including the following variables: hospital arrival date, group allocation, treatment allocation, study periods, mRS at month 6, mRS at day 7, National Institutes of Health Stroke Scale (NIHSS) score at day 7, patient characteristics including age, sex, ethnic group, place of residence, pre-stroke mRS, occupation, education, history of intracerebral haemorrhage, history of acute ischaemic stroke, history of stroke of unknown type, coronary heart disease, atrial fibrillation, heart failure, hypertension, treatment of hypertension, diabetes mellitus, hypercholesterolaemia, known coagulation or other haematological disorder, epilepsy, liver disease, current smoker and regular alcohol consumptions, other major health conditions that limits daily activity, height, weight, systolic blood pressure, diastolic blood pressure, heart rate, blood glucose, body temperature, Glasgow coma scale (GCS) score, NIHSS on arrival, anticoagulation treatment, INR on arrival, admitted places, presence of haematoma, volume of haematoma, doctor’s opinion on patient’s survival in the next 48 hours. mRS distributions were obtained by averaging across the 100 imputed datasets

*Ordinal logistic regression for mRS, logistic regression for death/disability, linear regression for NIHSS and EQ-5D-3L, with a random effect for cluster (hospital site), a fixed effect indicating the group assignment of each cluster at each step, and a fixed categorical effect of 6-month interval.

†Further adjusted model with inclusion of country (grouped as China vs. India/Pakistan/Sri Lanka/Vietnam vs. Brazil/Peru/Chile/Mexico/Nigeria), pre-stroke mRS categorical score, age, sex, and baseline NIHSS score.

Table S14. Pre-specified sensitivity analyses of the primary and secondary outcomes

	Unadjusted model*				Adjusted model†			
**Outcomes**	**N**	**OR or MD (95% CI)**	**P value**	**ICC**	**N**	**OR or MD (95% CI)**	**p value**	**ICC**
**mRS score at 6 months**								
Common time trend	6255	0.86 (0.76, 0.97)	0.015	*0.049*	6069	0.84 (0.73, 0.97)	0.017	*0.067*
Multiple imputations (MAR)	7036	0.86 (0.76, 0.97)	0.017		7036	0.85 (0.74, 0.98)	0.028	
Multiple imputations (+1)	7036	0.88 (0.78, 0.99)	0.034		7036	0.86 (0.75, 0.99)	0.033	
Multiple imputations (Death)	7036	0.92 (0.81, 1.03)	0.15		7036	0.89 (0.78, 1.02)	0.082	
Varying time trends‡	6255	0.86 (0.76, 0.98)	0.025	*0.053*	6069	0.83 (0.72, 0.97)	0.017	*0.074*
**NIHSS score at Day 7 (ordinal)**								
Common time trend	6809	0.89 (0.77, 1.03)	0.12	*0.234*	6772	0.91 (0.78, 1.05)	0.20	*0.083*
Varying time trends‡	6809	0.88 (0.73, 1.07)	0.20	*0.258*	6772	0.86 (0.72, 1.04)	0.12	*0.113*
**NIHSS score at Day 7 (continuous)**								
Common time trend	6582	0.40 (-0.36, 1.17)	0.30	*0.203*	6567	-0.05 (-0.50, 0.39)	0.82	*0.051*
Varying time trends‡	6582	0.45 (-0.45, 1.35)	0.32	*0.220*	6567	-0.06 (-0.57, 0.45)	0.81	*0.070*
**Death or Disability (mRS 3-6) at Month 6**								
Common time trend	6255	0.89 (0.78, 1.02)	0.10	*0.035*	6069	0.83 (0.70, 0.99)	0.040	*0.046*
Varying time trends‡	6255	0.89 (0.78, 1.03)	0.12	*0.037*	6069	0.83 (0.69, 0.99)	0.038	*0.048*
**Death at Month 6**								
Common time trend	6440	0.77 (0.63, 0.95)	0.015	*0.074*	6250	0.84 (0.65, 1.07)	0.16	*0.079*
Varying time trends‡	6440	0.75 (0.60, 0.95)	0.015	*0.080*	6250	0.83 (0.63, 1.08)	0.17	*0.087*
**Disability (mRS 3-5) at Month 6**								
Common time trend	5277	0.96 (0.83, 1.11)	0.56	*0.025*	5132	0.86 (0.72, 1.02)	0.08	*0.044*
Varying time trends‡	5277	0.97 (0.83, 1.12)	0.66	*0.028*	5132	0.86 (0.72, 1.03)	0.10	*0.046*
**EQ-5D mobility (ordinal)**								
Common time trend	5277	0.87 (0.75, 1.01)	0.064	*0.018*	5132	0.89 (0.74, 1.06)	0.18	*0.038*
Varying time trends‡	5277	0.88 (0.75, 1.02)	0.084	*0.020*	5132	0.88 (0.74, 1.06)	0.18	*0.038*
**EQ-5D self-care (ordinal)**								

	Unadjusted model*				Adjusted model†			
**Outcomes**	**N**	**OR or MD (95% CI)**	**P value**	**ICC**	**N**	**OR or MD (95% CI)**	**p value**	**ICC**
Common time trend	5277	0.93 (0.82, 1.07)	0.32	*0.019*	5132	0.87 (0.74, 1.02)	0.093	*0.045*
Varying time trends‡	5277	0.93 (0.67, 1.30)	0.69	*0.019*	5132	0.86 (0.81, 0.92)	<0.0001	*0.045*
**EQ-5D usual activities (ordinal)**								
Common time trend	5277	0.97 (0.85, 1.10)	0.62	*0.024*	5132	0.91 (0.78, 1.06)	0.22	*0.046*
Varying time trends‡	5277	0.99 (0.86, 1.14)	0.85	*0.031*	5132	0.92 (0.78, 1.10)	0.36	*0.054*
**EQ-5D pain/discomfort (ordinal)**								
Common time trend	5277	0.78 (0.67, 0.91)	0.0016	*0.024*	5132	0.95 (0.81, 1.12)	0.54	*0.013*
Varying time trends‡	5277	0.82 (0.69, 0.98)	0.033	*0.039*	5132	0.98 (0.82, 1.17)	0.86	*0.026*
**EQ-5D anxiety/depression (ordinal)**								
Common time trend	5277	0.83 (0.69, 1.00)	0.046	*0.042*	5132	0.90 (0.74, 1.09)	0.29	*0.035*
Varying time trends‡	5277	0.86 (0.70, 1.04)	0.12	*0.049*	5132	0.93 (0.75, 1.14)	0.48	*0.042*
**Overall health visual analogue scale at month 6**								
Common time trend	5276	1.24 (-0.20, 2.67)	0.091	*0.019*	5131	1.35 (-0.23, 2.93)	0.094	*0.041*
Varying time trends‡	5276	1.16 (-0.33, 2.66)	0.13	*0.023*	5131	1.30 (-0.32, 2.93)	0.12	*0.044*
**EQ5D-3L utility score§ at Month 6**								
Common time trend	6255	0.04 (0.02, 0.07)	0.0008	*0.064*	6069	0.02 (-0.00, 0.05)	0.063	*0.080*
Varying time trends‡	6255	0.04 (0.02, 0.07)	0.0024	*0.067*	6069	0.02 (-0.00, 0.05)	0.080	*0.083*
**Time to hospital discharge (days) ¶**								
Common time trend	7016	0.13 (0.04, 0.22)	0.0034	*0.256*	6810	0.05 (-0.04, 0.14)	0.26	*0.132*
Varying time trends‡	7016	0.15 (0.06, 0.25)	0.0019	*0.250*	6810	0.05 (-0.04, 0.14)	0.24	*0.134*
**Hospital discharge at day 7**								
Common time trend	7016	0.72 (0.53, 0.98)	0.033	*0.327*	6810	1.27 (0.92, 1.76)	0.15	*0.135*
Varying time trends‡	7016	0.71 (0.52, 0.98)	0.040	*0.321*	6810	1.30 (0.92, 1.83)	0.13	*0.136*
**Home residence at 6 months**								
Common time trend	5277	0.94 (0.65, 1.35)	0.73	*0.033*	5132	1.04 (0.70, 1.54)	0.86	*0.029*
Varying time trends‡	5277	0.94 (0.64, 1.36)	0.74	*0.034*	5132	1.11 (0.75, 1.65)	0.60	*0.030*

*U-adjusted analysis, ordinal logistic regression for mRS, logistic regression for death/disability, linear regression for NIHSS and QoL utility score, with a random effect for cluster (hospital site), a fixed effect indicating the group assignment of each cluster at each step, and a fixed categorical effect of 6-month interval.

[†]Adjusted model adjusted for country (grouped as China vs. India/Pakistan/Sri Lanka/Vietnam vs. Brazil/Peru/Chile/Mexico/Nigeria), mRS before stroke, age, sex and baseline NIHSS score.

[‡]Time varying method adding random effect of time across sites.

[§]Utility score calculated using China weight for Chinese and UK weight for other countries outside China.

[¶]Duration of hospitalisation only for those who were discharged from hospital on or before day 7.

Figure S15. Relationship between intervention effect and calendar time using restricted cubic splines

3-month calendar time windows	1-month calendar time windows
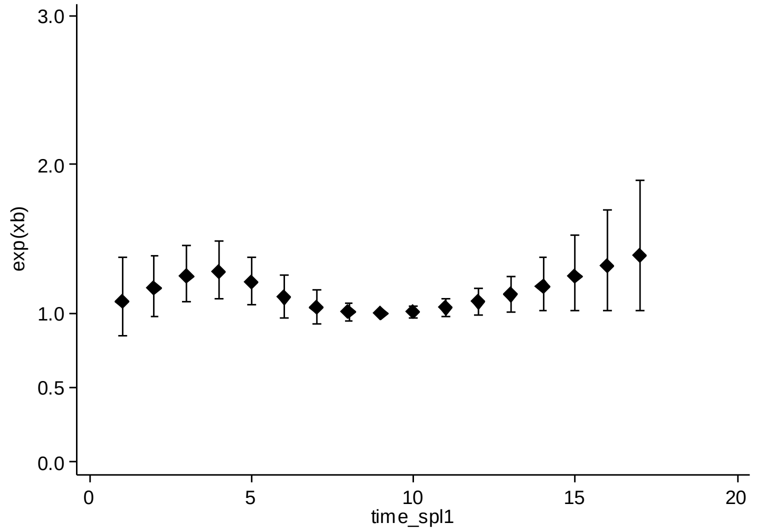	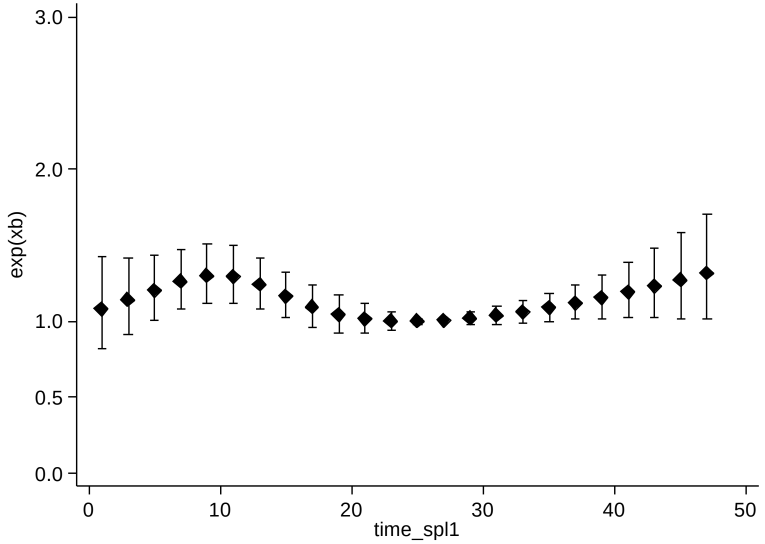

Footnote: X-axis represents the time point either in 3 month intervals (left) or 1 month intervals (right); Y-axis represents the odds ratio of time effect using time point Dec-2019 to Feb-2021 as reference point (left) and Jan-2020 as reference point (right).

Table S26. Post-hoc sensitivity analysis of clinical outcomes with time modelled using restricted cubic splines (RCS)

	Unadjusted model*				Adjusted model†			
**Outcomes**	**N**	**OR or MD (95%CI)**	**p value**	***ICC***	**N**	**OR or MD (95%CI)**	**p value**	***ICC***
**mRS score at 6 months**								
RCS using 3-month intervals	6255	0.84 (0.75, 0.95)	0.0058	*0.050*	6069	0.90 (0.78, 1.04)	0.15	*0.071*
RCS using 1-month intervals	6255	0.86 (0.76, 0.97)	0.013	*0.050*	6069	0.91 (0.79, 1.05)	0.18	*0.070*
**NIHSS score at Day 7 (ordinal)**								
RCS using 3-month intervals	6809	0.89 (0.77, 1.02)	0.093	*0.232*	6772	0.92 (0.80, 1.07)	0.29	*0.088*
RCS using 1-month intervals	6809	0.89 (0.77, 1.03)	0.12	*0.232*	6772	0.92 (0.79, 1.07)	0.26	*0.087*
**NIHSS score at Day 7 (continuous)**								
RCS using 3-month intervals	6582	0.31 (-0.45, 1.07)	0.42	*0.201*	6567	0.03 (-0.41, 0.48)	0.88	*0.052*
RCS using 1-month intervals	6582	0.35 (-0.41, 1.11)	0.37	*0.201*	6567	0.07 (-0.37, 0.51)	0.75	*0.050*
**Death or disability (mRS 3-6) at 6 months**								
RCS using 3-month intervals	6255	0.87 (0.76, 1.00)	0.044	*0.036*	6069	0.84 (0.70, 1.00)	0.048	*0.046*
RCS using 1-month intervals	6255	0.88 (0.77, 1.01)	0.074	*0.036*	6069	0.86 (0.72, 1.02)	0.076	*0.046*
**Death at 6 months**								
RCS using 3-month intervals	6440	0.74 (0.60, 0.90)	0.0034	*0.073*	6250	0.82 (0.64, 1.06)	0.13	*0.076*
RCS using 1-month intervals	6440	0.75 (0.61, 0.93)	0.0070	*0.073*	6250	0.82 (0.64, 1.06)	0.13	*0.076*
**Disability (mRS 3-5) at 6 months**								
RCS using 3-month intervals	5277	0.94 (0.82, 1.09)	0.40	*0.025*	5132	0.86 (0.72, 1.02)	0.085	*0.044*
RCS using 1-month intervals	5277	0.95 (0.82, 1.10)	0.50	*0.025*	5132	0.87 (0.73, 1.03)	0.12	*0.044*
**EQ-5D mobility (ordinal)**								
RCS using 3-month intervals	5277	0.87 (0.75, 1.00)	0.052	*0.019*	5132	0.91 (0.76, 1.08)	0.28	*0.038*
RCS using 1-month intervals	5277	0.88 (0.76, 1.01)	0.074	*0.019*	5132	0.93 (0.78, 1.10)	0.40	*0.038*
**EQ-5D self-care (ordinal)**								
RCS using 3-month intervals	5277	0.92 (0.81, 1.06)	0.24	*0.019*	5132	0.88 (0.75, 1.04)	0.13	*0.044*
RCS using 1-month intervals	5277	0.93 (0.81, 1.07)	0.31	*0.019*	5132	0.90 (0.76, 1.06)	0.19	*0.045*
**EQ-5D usual activities (ordinal)**								
RCS using 3-month intervals	5277	0.95 (0.83, 1.08)	0.40	*0.025*	5132	0.93 (0.79, 1.08)	0.34	*0.046*
RCS using 1-month intervals	5277	0.95 (0.83, 1.08)	0.46	*0.024*	5132	0.90 (0.77, 1.05)	0.19	*0.046*

Outcomes	Unadjusted model* N	OR or MD (95%CI)	p value	*ICC*	Adjusted model† N	OR or MD (95%CI)	p value	*ICC*
**EQ-5D pain/discomfort (ordinal)**								
RCS using 3-month intervals	5277	0.77 (0.66, 0.90)	0.0012	*0.025*	5132	0.94 (0.80, 1.10)	0.45	*0.013*
RCS using 1-month intervals	5277	0.77 (0.66, 0.90)	0.0009	*0.025*	5132	0.94 (0.80, 1.10)	0.44	*0.013*
**EQ-5D anxiety/depression (ordinal)**								
RCS using 3-month intervals	5277	0.82 (0.68, 0.98)	0.027	*0.046*	5132	0.87 (0.72, 1.05)	0.15	*0.038*
RCS using 1-month intervals	5277	0.82 (0.68, 0.98)	0.028	*0.047*	5132	0.88 (0.73, 1.07)	0.21	*0.038*
**Overall health visual analogue scale at 6 months**								
RCS using 3-month intervals	5276	1.26 (-0.17, 2.69)	0.084	*0.019*	5131	1.35 (-0.21, 2.92)	0.091	*0.040*
RCS using 1-month intervals	5276	1.16 (-0.28, 2.59)	0.11	*0.019*	5131	1.05 (-0.52, 2.61)	0.19	*0.038*
**EQ-5D-3L utility Score‡ at month 6**								
RCS using 3-month intervals	6255	0.05 (0.02, 0.07)	0.0001	*0.063*	6069	0.03 (0.00, 0.05)	0.023	*0.078*
RCS using 1-month intervals	6255	0.05 (0.02, 0.07)	0.0004	*0.064*	6069	0.02 (-0.00, 0.05)	0.054	*0.079*
**Time to hospital discharge (days)***								
RCS using 3-month intervals	7016	0.13 (0.04, 0.22)	0.0032	*0.249*	6810	0.02 (-0.06, 0.11)	0.60	*0.125*
RCS using 1-month intervals	7016	0.12 (0.03, 0.21)	0.0066	*0.252*	6810	0.02 (-0.07, 0.11)	0.65	*0.125*
**Hospital discharge at Day 7**								
RCS using 3-month intervals	7016	0.63 (0.47, 0.85)	0.0022	*0.313*	6810	1.30 (0.94, 1.81)	0.12	*0.133*
RCS using 1-month intervals	7016	0.64 (0.48, 0.85)	0.0027	*0.311*	6810	1.03 (0.75, 1.41)	0.86	*0.132*
**Residence at home at 6 months**								
RCS using 3-month intervals	5277	0.94 (0.65, 1.36)	0.75	*0.033*	5132	1.09 (0.73, 1.62)	0.67	*0.029*
RCS using 1-month intervals	5277	0.95 (0.66, 1.37)	0.79	*0.032*	5132	1.06 (0.71, 1.57)	0.78	*0.029*

EQ-5D-3L EuroQol health-related quality of life scale, mRS modified Rankin scale, NIHSS National Institutes of Health Stroke Scale

Unadjusted analysis, ordinal logistic regression for mRS, logistic regression for death/disability, linear regression for NIHSS and QoL utility score, with a random effect for cluster (hospital site), a fixed effect indicating the group assignment of each cluster at each step, and a fixed categorical effect of 6-month interval.

†Adjusted model adjusted for country (grouped as China vs. India/Pakistan/Sri Lanka/Vietnam vs. Brazil/Peru/Chile/Mexico/Nigeria), mRS before stroke, age, sex and baseline NIHSS score.

‡Utility score calculated using China weight for Chinese and UK weight for other countries outside China.

.

Table S17. Prespecified primary analysis with time modelled using the original study period (4 periods)

	Unadjusted model				Adjusted model[†]			
**Outcomes**	**N**	**OR or MD (95%CI)**	**p value**	**ICC**	**N**	**OR or MD (95%CI)**	**p value**	**ICC**
**mRS score at 6 months (ordinal)**								
Common time trend	6255	0.98 (0.83, 1.17)	0.84	*0.056*	6069	0.89 (0.74, 1.07)	0.21	*0.073*
Varying time trends	6255	0.96 (0.79, 1.16)	0.67	*0.062*	6069	0.83 (0.67, 1.03)	0.096	*0.079*
**NIHSS score at day 7 (ordinal)**								
Common time trend	6809	0.93 (0.78, 1.11)	0.44	*0.222*	6772	0.84 (0.70, 1.01)	0.069	*0.083*
Varying time trends	6809	0.96 (0.72, 1.28)	0.78	*0.250*	6772	0.82 (0.62, 1.08)	0.16	*0.110*
**NIHSS score at Day 7 (continuous)**								
Common time trend	6582	0.45 (-0.52, 1.41)	0.36	*0.201*	6567	0.28 (-0.30, 0.85)	0.35	*0.051*
Varying time trends	6582	0.52 (-0.83, 1.87)	0.45	0.218	6567	0.29 (-0.50, 1.07)	0.47	*0.077*
**Death or Disability (mRS 3-6) at Month 6 (binary)**								
Common time trend	6255	1.00 (0.82, 1.21)	0.97	*0.037*	6069	0.93 (0.74, 1.17)	0.54	*0.048*
Varying time trends	6255	1.00 (0.96, 1.03)	0.77	0.037	6069	0.91 (0.72, 1.16)	0.46	*0.049*
**Death at Month 6 (binary)**								
Common time trend	6440	0.94 (0.71, 1.26)	0.69	*0.093*	6250	0.84 (0.61, 1.16)	0.30	*0.082*
Varying time trends	6440	0.89 (0.64, 1.26)	0.52	0.104	6250	0.77 (0.53, 1.12)	0.17	*0.093*
**Disability (mRS 3-5) at Month 6 (binary)**								
Common time trend	5277	0.99 (0.81, 1.22)	0.95	*0.027*	5132	0.91 (0.72, 1.15)	0.44	*0.045*
Varying time trends	5277	0.99 (0.97, 1.02)	0.55	0.027		NA		
**EQ-5D mobility (ordinal)**								
Common time trend	5277	1.06 (0.85, 1.31)	0.62	*0.023*				
**EQ-5D self-care (ordinal)**								
Common time trend	5277	0.94 (0.77, 1.14)	0.52	*0.021*				
**EQ-5D usual activities (ordinal)**								
Common time trend	5277	0.96 (0.79, 1.17)	0.69	*0.032*				
**EQ-5D pain/discomfort (ordinal)**								
Common time trend	5277	0.77 (0.62, 0.96)	0.023	*0.029*				
**EQ-5D anxiety/depression (ordinal)**								

Outcomes	Unadjusted model N	OR or MD (95%CI)	p value	ICC	Adjusted model† N	OR or MD (95%CI)	p value	ICC
Common time trend	5277	0.66 (0.51, 0.85)	0.0014	*0.045*				
**Overall health visual analogue scale at Month 6 (continuous)**								
Common time trend	5276	1.72 (-0.39, 3.82)	0.11	*0.026*				
**EQ5D-3L Utility Score‡ at Month 6 (continuous)**								
Common time trend	6255	0.01 (-0.03, 0.04)	0.65	*0.084*				
**hospital discharge at day 7* (binary)**								
Common time trend	7016	0.85 (0.58, 1.24)	0.39	*0.374*				
Varying time trends								
**place of residence at month 6 (binary)**								
Common time trend	5277	0.91 (0.56, 1.49)	0.72	*0.030*				
Varying time trends								

EQ-5D-3L EuroQol health-related quality of life scale, mRS modified Rankin scale, NIHSS National Institutes of Health Stroke Scale

*Unadjusted analysis, ordinal logistic regression for mRS, logistic regression for death/disability, linear regression for NIHSS and QoL utility score, with a random effect for cluster (hospital site), a fixed effect indicating the group assignment of each cluster at each step, and a fixed categorical effect of 6-month interval.

†Adjusted model adjusted for country (grouped as China vs. India/Pakistan/Sri Lanka/Vietnam vs. Brazil/Peru/Chile/Mexico/Nigeria), mRS before stroke, age, sex and baseline NIHSS score.

‡Utility score calculated using China weight for Chinese and UK weight for other countries outside China.

Table S18. Family-wise error adjustment across secondary clinical outcomes using a sequential Holm-Sidak correction

Secondary outcomes	Ordered p values from the model	Number of comparisons remaining	Adjusted significance level (Holm-Sidak)	Conclusion
EQ-5D-3L utility score* (continuous)	0.0008	7	0.007	Remains significant
Hospital discharge by day 7 (binary)	0.034	6	0.009	Changes to non-significant
Death at 6 months (binary)	0.015	5	0.010	Changes to non-significant
Poor outcome (mRS 3-6) at 6 months (binary)	0.10	4	0.013	Remains non-significant
NIHSS at day 7 (ordinal)	0.12	3	0.017	Remains non-significant
Dependency at 6 months (binary)	0.56	2	0.025	Remains non-significant
Home residence at 6 months (binary)	0.73	1	0.05	Remains non-significant

*EQ-5D-3L denotes the EuroQol health-related quality of life questionnaire which covers 5 domains of health-related quality of life: mobility, self-care, usual activities, pain/discomfort, and anxiety/depression. Each domain has 3 graded levels of response: ‘no problems’, ‘moderate problems’, or ‘extreme problems’. Scores from these levels are combined to provide an overall health utility score that was calculated with population norms from the United Kingdom.

mRS denotes modified Rankin scale, NIHSS National Institutes of Health Stroke Scale, sICH symptomatic intracerebral haemorrhage

Table S19. Subgroup analysis for primary outcome on the range of scores on the modified Rankin scale

	Raw counts and percentage			Primary model		
**Characteristics**	**Intervention (N=3221)**	**Control (N=3815)**	**Total (N=7036)**	**Odds ratio (95%CI)**	**p value**	**p value for interaction**
**Age**						**0.25**
<60 years				0.93 (0.79, 1.09)	0.35	
0 - no symptoms	135/1255 (10.8)	180/1379 (13.1)	315/2634 (12.0)			
1 - no significant disability	433/1255 (34.5)	431/1379 (31.3)	864/2634 (32.8)			
2 - slight disability	95/1255 (7.6)	92/1379 (6.7)	187/2634 (7.1)			
3 - moderate disability	287/1255 (22.9)	274/1379 (19.9)	561/2634 (21.3)			
4 - moderate severe disability	121/1255 (9.6)	145/1379 (10.5)	266/2634 (10.1)			
5 - severe disability	76/1255 (6.1)	93/1379 (6.7)	169/2634 (6.4)			
6 - death	108/1255 (8.6)	164/1379 (11.9)	272/2634 (10.3)			
60-69 years				0.88 (0.73, 1.06)	0.18	
0 - no symptoms	56/813 (6.9)	92/1011 (9.1)	148/1824 (8.1)			
1 - no significant disability	261/813 (32.1)	266/1011 (26.3)	527/1824 (28.9)			
2 - slight disability	50/813 (6.2)	67/1011 (6.6)	117/1824 (6.4)			
3 - moderate disability	153/813 (18.8)	177/1011 (17.5)	330/1824 (18.1)			
4 - moderate severe disability	114/813 (14.0)	149/1011 (14.7)	263/1824 (14.4)			
5 - severe disability	78/813 (9.6)	107/1011 (10.6)	185/1824 (10.1)			
6 - death	101/813 (12.4)	153/1011 (15.1)	254/1824 (13.9)			
70-79 years				0.87 (0.70, 1.08)	0.23	
0 - no symptoms	42/604 (7.0)	58/694 (8.4)	100/1298 (7.7)			
1 - no significant disability	173/604 (28.6)	160/694 (23.1)	333/1298 (25.7)			
2 - slight disability	34/604 (5.6)	34/694 (4.9)	68/1298 (5.2)			
3 - moderate disability	82/604 (13.6)	89/694 (12.8)	171/1298 (13.2)			
4 - moderate severe disability	79/604 (13.1)	118/694 (17.0)	197/1298 (15.2)			
5 - severe disability	67/604 (11.1)	96/694 (13.8)	163/1298 (12.6)			

Characteristics	Raw counts and percentage<br>Intervention<br>(N=3221)	Control<br>(N=3815)	Total<br>(N=7036)	Primary model<br>Odds ratio (95%CI)	p value	p value for interaction
6 - death	127/604 (21.0)	139/694 (20.0)	266/1298 (20.5)			
≥80 years				0.64 (0.45, 0.90)	0.010	
0 - no symptoms	14/219 (6.4)	12/275 (4.4)	26/494 (5.3)			
1 - no significant disability	40/219 (18.3)	37/275 (13.5)	77/494 (15.6)			
2 - slight disability	6/219 (2.7)	5/275 (1.8)	11/494 (2.2)			
3 - moderate disability	26/219 (11.9)	30/275 (10.9)	56/494 (11.3)			
4 - moderate severe disability	26/219 (11.9)	32/275 (11.6)	58/494 (11.7)			
5 - severe disability	36/219 (16.4)	45/275 (16.4)	81/494 (16.4)			
6 - death	71/219 (32.4)	114/275 (41.5)	185/494 (37.4)			
**Sex**						**0.16**
Male				0.90 (0.78, 1.04)	0.15	
0 - no symptoms	158/1849 (8.5)	238/2122 (11.2)	396/3971 (10.0)			
1 - no significant disability	585/1849 (31.6)	594/2122 (28.0)	1179/3971 (29.7)			
2 - slight disability	121/1849 (6.5)	126/2122 (5.9)	247/3971 (6.2)			
3 - moderate disability	369/1849 (20.0)	340/2122 (16.0)	709/3971 (17.9)			
4 - moderate severe disability	198/1849 (10.7)	253/2122 (11.9)	451/3971 (11.4)			
5 - severe disability	157/1849 (8.5)	193/2122 (9.1)	350/3971 (8.8)			
6 - death	261/1849 (14.1)	378/2122 (17.8)	639/3971 (16.1)			
Female				0.79 (0.67, 0.94)	0.0067	
0 - no symptoms	89/1043 (8.5)	104/1241 (8.4)	193/2284 (8.5)			
1 - no significant disability	322/1043 (30.9)	302/1241 (24.3)	624/2284 (27.3)			
2 - slight disability	64/1043 (6.1)	72/1241 (5.8)	136/2284 (6.0)			
3 - moderate disability	180/1043 (17.3)	231/1241 (18.6)	411/2284 (18.0)			
4 - moderate severe disability	142/1043 (13.6)	191/1241 (15.4)	333/2284 (14.6)			

Characteristics	Raw counts and percentage<br>Intervention (N=3221)	Control (N=3815)	Total (N=7036)	Primary model<br>Odds ratio (95%CI)	p value	p value for interaction
5 - severe disability	100/1043 (9.6)	148/1241 (11.9)	248/2284 (10.9)			
6 - death	146/1043 (14.0)	193/1241 (15.6)	339/2284 (14.8)			
**Country/region**						**0.0090**
China				0.92 (0.80, 1.06)	0.24	
0 - no symptoms	199/2509 (7.9)	316/3078 (10.3)	515/5587 (9.2)			
1 - no significant disability	825/2509 (32.9)	850/3078 (27.6)	1675/5587 (30.0)			
2 - slight disability	129/2509 (5.1)	155/3078 (5.0)	284/5587 (5.1)			
3 - moderate disability	482/2509 (19.2)	526/3078 (17.1)	1008/5587 (18.0)			
4 - moderate severe disability	314/2509 (12.5)	409/3078 (13.3)	723/5587 (12.9)			
5 - severe disability	242/2509 (9.6)	330/3078 (10.7)	572/5587 (10.2)			
6 - death	318/2509 (12.7)	492/3078 (16.0)	810/5587 (14.5)			
India/Pakistan/Sri Lanka/Vietnam				0.93 (0.59, 1.47)	0.77	
0 - no symptoms	34/280 (12.1)	24/216 (11.1)	58/496 (11.7)			
1 - no significant disability	71/280 (25.4)	41/216 (19.0)	112/496 (22.6)			
2 - slight disability	43/280 (15.4)	37/216 (17.1)	80/496 (16.1)			
3 - moderate disability	43/280 (15.4)	33/216 (15.3)	76/496 (15.3)			
4 - moderate severe disability	15/280 (5.4)	24/216 (11.1)	39/496 (7.9)			
5 - severe disability	14/280 (5.0)	8/216 (3.7)	22/496 (4.4)			
6 - death	60/280 (21.4)	49/216 (22.7)	109/496 (22.0)			
Brazil/Peru/Chile/Mexico/Nigeria				0.36 (0.20, 0.67)	0.0011	
0 - no symptoms	14/103 (13.6)	2/69 (2.9)	16/172 (9.3)			
1 - no significant disability	11/103 (10.7)	5/69 (7.2)	16/172 (9.3)			
2 - slight disability	13/103 (12.6)	6/69 (8.7)	19/172 (11.0)			
3 - moderate disability	24/103 (23.3)	12/69 (17.4)	36/172 (20.9)			

Characteristics	Raw counts and percentage<br>Intervention<br>(N=3221)	Control<br>(N=3815)	Total<br>(N=7036)	Primary model<br>Odds ratio (95%CI)	p value	p value for interaction
4 - moderate severe disability	11/103 (10.7)	11/69 (15.9)	22/172 (12.8)			
5 - severe disability	1/103 (1.0)	3/69 (4.3)	4/172 (2.3)			
6 - death	29/103 (28.2)	30/69 (43.5)	59/172 (34.3)			
**NIHSS**						**0.070**
≥15				0.79(0.67, 0.94)	0.0064	
0 - no symptoms	57/1248 (4.6)	72/1481 (4.9)	129/2729 (4.7)			
1 - no significant disability	239/1248 (19.2)	256/1481 (17.3)	495/2729 (18.1)			
2 - slight disability	54/1248 (4.3)	63/1481 (4.3)	117/2729 (4.3)			
3 - moderate disability	251/1248 (20.1)	240/1481 (16.2)	491/2729 (18.0)			
4 - moderate severe disability	192/1248 (15.4)	219/1481 (14.8)	411/2729 (15.1)			
5 - severe disability	170/1248 (13.6)	211/1481 (14.2)	381/2729 (14.0)			
6 - death	285/1248 (22.8)	420/1481 (28.4)	705/2729 (25.8)			
<15				0.94 (0.81, 1.10)	0.46	
0 - no symptoms	179/1580 (11.3)	262/1774 (14.8)	441/3354 (13.1)			
1 - no significant disability	647/1580 (40.9)	618/1774 (34.8)	1265/3354 (37.7)			
2 - slight disability	126/1580 (8.0)	115/1774 (6.5)	241/3354 (7.2)			
3 - moderate disability	284/1580 (18.0)	323/1774 (18.2)	607/3354 (18.1)			
4 - moderate severe disability	142/1580 (9.0)	212/1774 (12.0)	354/3354 (10.6)			
5 - severe disability	83/1580 (5.3)	127/1774 (7.2)	210/3354 (6.3)			
6 - death	119/1580 (7.5)	117/1774 (6.6)	236/3354 (7.0)			
**Haematoma volume**						**0.62**
<15 ml				0.90 (0.76, 1.05)	0.18	
0 - no symptoms	166/1381 (12.0)	207/1451 (14.3)	373/2832 (13.2)			
1 - no significant disability	564/1381 (40.8)	528/1451 (36.4)	1092/2832 (38.6)			

Characteristics	Raw counts and percentage<br>Intervention (N=3221)	Control (N=3815)	Total (N=7036)	Primary model<br>Odds ratio (95%CI)	p value	p value for interaction
2 - slight disability	100/1381 (7.2)	85/1451 (5.9)	185/2832 (6.5)			
3 - moderate disability	235/1381 (17.0)	228/1451 (15.7)	463/2832 (16.3)			
4 - moderate severe disability	116/1381 (8.4)	160/1451 (11.0)	276/2832 (9.7)			
5 - severe disability	84/1381 (6.1)	100/1451 (6.9)	184/2832 (6.5)			
6 - death	116/1381 (8.4)	143/1451 (9.9)	259/2832 (9.1)			
15-29 ml				0.88 (0.72, 1.06)	0.18	
0 - no symptoms	52/718 (7.2)	75/842 (8.9)	127/1560 (8.1)			
1 - no significant disability	207/718 (28.8)	208/842 (24.7)	415/1560 (26.6)			
2 - slight disability	45/718 (6.3)	56/842 (6.7)	101/1560 (6.5)			
3 - moderate disability	166/718 (23.1)	167/842 (19.8)	333/1560 (21.3)			
4 - moderate severe disability	98/718 (13.6)	121/842 (14.4)	219/1560 (14.0)			
5 - severe disability	64/718 (8.9)	91/842 (10.8)	155/1560 (9.9)			
6 - death	86/718 (12.0)	124/842 (14.7)	210/1560 (13.5)			
≥30ml				0.80 (0.65, 0.98)	0.032	
0 - no symptoms	20/718 (2.8)	33/804 (4.1)	53/1522 (3.5)			
1 - no significant disability	113/718 (15.7)	93/804 (11.6)	206/1522 (13.5)			
2 - slight disability	36/718 (5.0)	28/804 (3.5)	64/1522 (4.2)			
3 - moderate disability	137/718 (19.1)	136/804 (16.9)	273/1522 (17.9)			
4 - moderate severe disability	118/718 (16.4)	128/804 (15.9)	246/1522 (16.2)			
5 - severe disability	100/718 (13.9)	135/804 (16.8)	235/1522 (15.4)			
6 - death	194/718 (27.0)	251/804 (31.2)	445/1522 (29.2)			
**Haematoma location**						**0.11**
Cortical				1.14 (0.83, 1.56)	0.43	
0 - no symptoms	23/275 (8.4)	36/293 (12.3)	59/568 (10.4)			

Characteristics	Raw counts and percentage<br>Intervention<br>(N=3221)	 Control<br>(N=3815)	 Total<br>(N=7036)	Primary model<br>Odds ratio (95%CI)	 p value	p value for interaction
1 - no significant disability	77/275 (28.0)	84/293 (28.7)	161/568 (28.3)			
2 - slight disability	21/275 (7.6)	21/293 (7.2)	42/568 (7.4)			
3 - moderate disability	50/275 (18.2)	47/293 (16.0)	97/568 (17.1)			
4 - moderate severe disability	26/275 (9.5)	25/293 (8.5)	51/568 (9.0)			
5 - severe disability	30/275 (10.9)	17/293 (5.8)	47/568 (8.3)			
6 - death	48/275 (17.5)	63/293 (21.5)	111/568 (19.5)			
Deep				0.82 (0.72, 0.94)	0.0034	
0 - no symptoms	188/2322 (8.1)	247/2612 (9.5)	435/4934 (8.8)			
1 - no significant disability	732/2322 (31.5)	673/2612 (25.8)	1405/4934 (28.5)			
2 - slight disability	152/2322 (6.5)	151/2612 (5.8)	303/4934 (6.1)			
3 - moderate disability	458/2322 (19.7)	458/2612 (17.5)	916/4934 (18.6)			
4 - moderate severe disability	287/2322 (12.4)	362/2612 (13.9)	649/4934 (13.2)			
5 - severe disability	199/2322 (8.6)	292/2612 (11.2)	491/4934 (10.0)			
6 - death	306/2322 (13.2)	429/2612 (16.4)	735/4934 (14.9)			
Brainstem/Cerebellum				0.95 (0.69, 1.30)	0.76	
0 - no symptoms	28/256 (10.9)	44/331 (13.3)	72/587 (12.3)			
1 - no significant disability	87/256 (34.0)	98/331 (29.6)	185/587 (31.5)			
2 - slight disability	12/256 (4.7)	19/331 (5.7)	31/587 (5.3)			
3 - moderate disability	35/256 (13.7)	41/331 (12.4)	76/587 (12.9)			
4 - moderate severe disability	23/256 (9.0)	39/331 (11.8)	62/587 (10.6)			
5 - severe disability	23/256 (9.0)	25/331 (7.6)	48/587 (8.2)			
6 - death	48/256 (18.8)	65/331 (19.6)	113/587 (19.3)			
**COVID period**						**0.013**
Before 30 January 2020				0.96 (0.82, 1.11)	0.56	

Characteristics	Raw counts and percentage<br>Intervention (N=3221)	Control (N=3815)	Total (N=7036)	Primary model<br>Odds ratio (95%CI)	p value	p value for interaction
0 - no symptoms	127/1570 (8.1)	293/2828 (10.4)	420/4398 (9.5)			
1 - no significant disability	512/1570 (32.6)	770/2828 (27.2)	1282/4398 (29.1)			
2 - slight disability	87/1570 (5.5)	148/2828 (5.2)	235/4398 (5.3)			
3 - moderate disability	294/1570 (18.7)	472/2828 (16.7)	766/4398 (17.4)			
4 - moderate severe disability	174/1570 (11.1)	390/2828 (13.8)	564/4398 (12.8)			
5 - severe disability	163/1570 (10.4)	300/2828 (10.6)	463/4398 (10.5)			
6 - death	213/1570 (13.6)	455/2828 (16.1)	668/4398 (15.2)			
After 30 January 2020				0.69 (0.56, 0.85)	0.0006	
0 - no symptoms	120/1322 (9.1)	49/535 (9.2)	169/1857 (9.1)			
1 - no significant disability	395/1322 (29.9)	126/535 (23.6)	521/1857 (28.1)			
2 - slight disability	98/1322 (7.4)	50/535 (9.3)	148/1857 (8.0)			
3 - moderate disability	255/1322 (19.3)	99/535 (18.5)	354/1857 (19.1)			
4 - moderate severe disability	166/1322 (12.6)	54/535 (10.1)	220/1857 (11.8)			
5 - severe disability	94/1322 (7.1)	41/535 (7.7)	135/1857 (7.3)			
6 - death	194/1322 (14.7)	116/535 (21.7)	310/1857 (16.7)			

CI denotes confidence interval, NIHSS National Institutes for Health Stroke Scale

Table S20. Baseline characteristics by region

Characteristic	China (N=6356)	India/Pakistan/SL/Vietnam (N=505)	Brazil/Peru/Chile/Mexico/Nigeria (N=175)	Total (N=7036)
Mean age, years	62.1 (12.5)	61.9 (12.5)	58.1 (14.6)	62.0 (12.6)
Female	2317 (36.5)	158 (31.3)	58 (33.1)	2533 (36.0)
Skilled occupation*	721/6354 (11)	101/503 (20)	61/170 (36)	885/7013 (16)
BMI†	24.0 (3.4)	23.9 (4.6)	28.3 (5.3)	23.9 (3.6)
Medical history				
History of hypertension	4408/6356 (69.4)	337/505 (66.7)	143/174 (82.2)	4911/7035 (69.5)
History of ICH	512/6356 (8.1)	23/505 (4.6)	6/174 (3.4)	541/7035 (7.7)
History of ischaemic stroke	516/6356 (8.1)	37/505 (7.3)	12/174 (6.9)	565/7035 (8.0)
History of coronary artery disease	168/6356 (2.6)	18/505 (3.6)	7/174 (4.0)	193/7035 (2.7)
History of heart failure	33/6356 (0.5)	3/505 (0.6)	7/174 (4.0)	43/7035 (0.6)
History of other heart disease	225/6356 (3.5)	21/505 (4.2)	8/174 (4.6)	254/7035 (3.6)
History of atrial fibrillation	67/6356 (1.1)	4/505 (0.8)	11/174 (6.3)	82/7035 (1.2)
History of diabetes mellitus	589/6356 (9.3)	109/505 (21.6)	31/174 (17.8)	729/7035 (10.4)
History of hypercholesterolaemia	126/6355 (2.0)	55/505 (10.9)	26/174 (14.9)	207/7034 (2.9)
Current smoker	1288/6356 (20.3)	54/505 (10.7)	17/173 (9.8)	1359/7034 (19.3)
Current alcohol consumption	1275/6356 (20.1)	85/505 (16.8)	34/173 (19.7)	1394/7034 (19.8)
mRS score of 0 before onset‡	4899/6356 (77.1)	349/439 (79.5)	135/174 (77.6)	5383/6969 (77.2)
Medications				
Antihypertensive medication	2742/6356 (43.1)	224/504 (44.4)	106/174 (60.9)	3072/7034 (43.7)
Blood glucose lowering agents	410/6356 (6.5)	78/505 (15.4)	25/174 (14.4)	513/7035 (7.3)
Statin/other lipid lowering agent	129/6356 (2.0)	72/505 (14.3)	21/174 (12.1)	222/7035 (3.2)
Aspirin/other antiplatelet agent	310/6356 (4.9)	50/505 (9.9)	18/174 (10.3)	378/7035 (5.4)
Anticoagulation agent	48/6356 (0.8)	7/505 (1.4)	10/174 (5.7)	65/7035 (0.9)
Mean SBP, mm Hg	174 (28)	180 (33)	186 (34)	175 (28)
Mean DBP, mm Hg	99 (17)	103 (20)	108 (24)	99 (18)
NIHSS score§	14 (7-23)	10 (6-16)	13 (8-21)	13 (7-22)
GCS score¶	12 (9-14)	15 (11-15)	14 (10-15)	12 (9-14)
Brain imaging features‖				
Haematoma on CT scan	6178/6356 (97.2)	503/505 (99.6)	174/174 (100.0)	6855/7035 (97.4)
Volume of haematoma	15 (8-30)	11 (5-24)	14 (7-37)	15 (8-30)

Side of haematoma				
left	3051/6174 (49.4)	262/503 (52.1)	90/174 (51.7)	3403/6851 (49.7)
Right	2849/6174 (46.1)	231/503 (45.9)	81/174 (46.6)	3161/6851 (46.1)
Midline	368/6174 (6.0)	21/503 (4.2)	4/174 (2.3)	393/6851 (5.7)
Location of the haematoma				
Deep	5113/6172 (82.8)	394/503 (78.3)	131/174 (75.3)	5638/6849 (82.3)
Cortical	507/6172 (8.2)	83/503 (16.5)	38/174 (21.8)	628/6849 (9.2)
Cerebellum	340/6172 (5.5)	21/503 (4.2)	8/174 (4.6)	369/6849 (5.4)
Brainstem	322/6172 (5.2)	22/503 (4.4)	7/174 (4.0)	351/6849 (5.1)
Intraventricular haemorrhage	1904/6353 (30.0)	130/505 (25.7)	59/174 (33.9)	2093/7032 (29.8)
Presumed aetiology‖				
Hypertension vasculopathy	5955/6314 (94.3)	468/495 (94.5)	151/163 (92.6)	6574/6972 (94.3)
Cerebral amyloid angiopathy	334/6314 (5.3)	2/495 (0.4)	9/163 (5.5)	345/6972 (4.9)
Other	25/6314 (0.4)	25/495 (5.1)	3/163 (1.8)	53/6972 (0.8)
Abnormal physiological parameters				
SBP ≥140 mm Hg	5741/6349 (90.4%)	420/466 (90.1%)	159/173 (91.9%)	6320/6988 (90.4%)
Blood glucose >7.8, mmol/L	2177/6091 (35.7%)	171/448 (38.2%)	65/172 (37.8%)	2413/6711 (36.0%)
Body temperature >37.5 °C	103/6313 (1.6%)	5/447 (1.1%)	12/170 (7.1%)	120/6930 (1.7%)
INR ≥1.5	68/6133 (1.1%)	7/456 (1.5%)	9/171 (5.3%)	84/6760 (1.2%)

Data are mean (SD), n (%), median (IQR), or n/N (%)

BMI denotes body mass index, CT computerised tomography, DBP diastolic blood pressure, GCS Glasgow coma scale, INR international normalised ratio, mRS modified Rankin scale, NIHSS National Institutes of Health Stroke Scale, SBP systolic blood pressure, SL Sri Lanka

*Skilled includes professional/executive, business, sales and service; unskilled includes driver, farmer/labourer, home duties, other

†Calculated by dividing weight (kg) by height (metres^2)

‡Scores on the mRS of functional recovery range from 0 (no symptoms) to 6 (death). A score of 0 indicates ‘no symptoms’ and a score 2 or less indicates functional independence.

§Scores on the NIHSS range from 0 to 42, with higher scores indicating more severe neurologic deficits.

¶Scores on the GCS range from 15 (normal) to 3 (deep coma).

‖Reported by clinician investigators

Table S21. Management of patients over 7 days by region

Characteristic	China (N=6384)		India/Pakistan/Sri Lanka/Vietnam (N=505)		Brazil/Peru/Chile/Mexico/Nigeria (N=175)	
	**Care bundle**	**Usual care**	**Care bundle**	**Usual care**	**Care bundle**	**Usual care**
**Location of admission**						
Neurosurgery	2310/2818 (82.0)	2940/3497 (84.1)	7/282 (2.5)	4/213 (1.9)	0/97 (0.0)	2/66 (3.0)
Neurology	156/2818 (5.5)	207/3497 (5.9)	156/282 (55.3)	88/213 (41.3)	68/97 (70.1)	41/66 (62.1)
Intensive care	306/2818 (10.9)	293/3497 (8.4)	26/282 (9.2)	14/213 (6.6)	9/97 (9.3)	10/66 (15.2)
Emergency department	0/2818 (0.0)	10/3497 (0.3)	4/282 (1.4)	1/213 (0.5)	10/97 (10.3)	3/66 (4.5)
Other	46/2818 (1.6)	47/3497 (1.3)	89/282 (31.6)	106/213 (49.8)	10/97 (10.3)	10/66 (15.2)
**Treatment in the first 24 hours**						
IV BP lowering	2331/2831 (82.3)	2635/3525 (74.8)	231/285 (81.1)	138/217 (63.6)	94/105 (89.5)	44/69 (63.8)
Glycaemic control	190/2831 (6.7)	220/3525 (6.2)	46/285 (16.1)	32/220 (14.5)	14/105 (13.3)	11/69 (15.9)
Antipyrexia treatment	280/2830 (9.9)	260/3524 (7.4)	22/284 (7.7)	14/216 (6.5)	12/105 (11.4)	6/69 (8.7)
Correction of abnormal coagulation	6/18 (33.3%)	17/50 (34.0%)	2/3 (66.7%)	3/4 (75.0%)	4/4 (100.0%)	4/5 (80.0%)
**Other management until day 7**						
Intravenous BP lowering	2081/2817 (73.9)	2279/3496 (65.2)	128/274 (46.7)	68/213 (31.9)	78/97 (80.4)	45/66 (68.2)
Oral BP lowering	1935/2817 (68.7)	2311/3496 (66.1)	255/274 (93.1)	185/213 (86.9)	77/97 (79.4)	51/66 (77.3)
Insulin	416/2817 (14.8)	390/3496 (11.2)	69/274 (25.2)	34/213 (16.0)	14/97 (14.4)	16/66 (24.2)
Hypothermia	632/2817 (22.4)	731/3496 (20.9)	33/274 (12.0)	19/213 (8.9)	4/97 (4.1)	1/66 (1.5)
PCC administered	329/2817 (11.7)	289/3496 (8.3)	0/274 (0.0)	0/213 (0.0)	3/97 (3.1)	0/66 (0.0)
Fresh frozen plasma	57/2817 (2.0)	102/3496 (2.9)	4/274 (1.5)	1/213 (0.5)	0/97 (0.0)	1/66 (1.5)
Vitamin K	147/2817 (5.2)	132/3496 (3.8)	26/274 (9.5)	29/213 (13.6)	1/97 (1.0)	2/66 (3.0)
Decompressive surgery	831/2818 (29.5)	1006/3496 (28.8)	6/281 (2.1)	5/213 (2.3)	7/97 (7.2)	5/66 (7.6)
Mechanical ventilation	619/2818 (22.0)	775/3496 (22.2)	20/281 (7.1)	15/213 (7.0)	7/97 (7.2)	16/66 (24.2)
Intensive care admission	1015/2818 (36.0)	1394/3496 (39.9)	63/281 (22.4)	26/213 (12.2)	27/97 (27.8)	15/66 (22.7)
Assisted feeding	1568/2818 (55.6)	1755/3496 (50.2)	133/281 (47.3)	123/213 (57.7)	48/97 (49.5)	42/66 (63.6)
Decision to withdraw active care	16/2818 (0.6)	17/3496 (0.5)	4/281 (1.4)	6/213 (2.8)	1/97 (1.0)	3/66 (4.5)

Table S22. Serious adverse events summary (investigator reported)

System Organ Class<br>Preferred Term	Care bundle<br>(N=3221)	Usual care<br>(N=3815)	Total<br>(N=7036)	p value*
All Coded AEs: number of events, number of subjects (%)	543 516 (16.0)	822 765 (20.1)	1365 1281 (18.2)	0.0098
Nervous system disorders	204 202 (6.3)	331 329 (8.6)	535 531 (7.5)	
Cerebral haemorrhage	162 160 (5.0)	246 245 (6.4)	408 405 (5.8)	
Ischaemic stroke	13 13 (0.4)	18 18 (0.5)	31 31 (0.4)	
Brain herniation	12 12 (0.4)	21 21 (0.6)	33 33 (0.5)	
Epilepsy	4 4 (0.1)	7 7 (0.2)	11 11 (0.2)	
Intracranial pressure increased	3 3 (0.1)	2 2 (0.1)	5 5 (0.1)	
Hydrocephalus	2 2 (0.1)	3 3 (0.1)	5 5 (0.1)	
Brain oedema	1 1 (0.0)	4 4 (0.1)	5 5 (0.1)	
Cerebral ventricle dilatation	1 1 (0.0)	1 1 (0.0)	2 2 (0.0)	
Cerebrovascular accident	1 1 (0.0)	11 11 (0.3)	12 12 (0.2)	
Cerebrovascular stenosis	1 1 (0.0)	0 0 (0.0)	1 1 (0.0)	
Encephalitis	1 1 (0.0)	0 0 (0.0)	1 1 (0.0)	
Facial paralysis	1 1 (0.0)	0 0 (0.0)	1 1 (0.0)	
Intraventricular haemorrhage	1 1 (0.0)	1 1 (0.0)	2 2 (0.0)	
Thalamus haemorrhage	1 1 (0.0)	0 0 (0.0)	1 1 (0.0)	
Brain death	0 0 (0.0)	1 1 (0.0)	1 1 (0.0)	
Brain stem haemorrhage	0 0 (0.0)	2 2 (0.1)	2 2 (0.0)	
CNS ventriculitis	0 0 (0.0)	1 1 (0.0)	1 1 (0.0)	
Coma	0 0 (0.0)	2 2 (0.1)	2 2 (0.0)	
Dizziness	0 0 (0.0)	2 2 (0.1)	2 2 (0.0)	
Headache	0 0 (0.0)	1 1 (0.0)	1 1 (0.0)	
Hemiplegia	0 0 (0.0)	1 1 (0.0)	1 1 (0.0)	
Muscular weakness	0 0 (0.0)	1 1 (0.0)	1 1 (0.0)	
Post concussion syndrome	0 0 (0.0)	1 1 (0.0)	1 1 (0.0)	

System Organ Class<br>Preferred Term	Care bundle<br>(N=3221)	Usual care<br>(N=3815)	Total<br>(N=7036)	p value*
Seizure	0 0 (0.0)	1 1 (0.0)	1 1 (0.0)	
Status epilepticus	0 0 (0.0)	3 3 (0.1)	3 3 (0.0)	
Superior sagittal sinus thrombosis	0 0 (0.0)	1 1 (0.0)	1 1 (0.0)	
General disorders and administration site conditions	200 199 (6.2)	238 236 (6.2)	438 435 (6.2)	
Death	133 133 (4.1)	123 123 (3.2)	256 256 (3.6)	
Condition aggravated	61 61 (1.9)	92 91 (2.4)	153 152 (2.2)	
Multiple organ dysfunction syndrome	5 5 (0.2)	15 15 (0.4)	20 20 (0.3)	
Infusion site oedema	1 1 (0.0)	0 0 (0.0)	1 1 (0.0)	
Adverse drug reaction	0 0 (0.0)	1 1 (0.0)	1 1 (0.0)	
Hyperpyrexia	0 0 (0.0)	1 1 (0.0)	1 1 (0.0)	
Oedema peripheral	0 0 (0.0)	2 2 (0.1)	2 2 (0.0)	
Puncture site haemorrhage	0 0 (0.0)	1 1 (0.0)	1 1 (0.0)	
Pyrexia	0 0 (0.0)	2 2 (0.1)	2 2 (0.0)	
Sudden death	0 0 (0.0)	1 1 (0.0)	1 1 (0.0)	
Respiratory, thoracic and mediastinal disorders	73 70 (2.2)	122 118 (3.1)	195 188 (2.7)	
Pneumonia	27 26 (0.8)	48 47 (1.2)	75 73 (1.0)	
Respiratory failure	18 18 (0.6)	34 34 (0.9)	52 52 (0.7)	
Cardio-respiratory arrest	12 12 (0.4)	5 5 (0.1)	17 17 (0.2)	
Pneumonia aspiration	6 5 (0.2)	11 11 (0.3)	17 16 (0.2)	
Lung neoplasm malignant	3 3 (0.1)	1 1 (0.0)	4 4 (0.1)	
Nasopharyngitis	2 2 (0.1)	4 4 (0.1)	6 6 (0.1)	
Asphyxia	1 1 (0.0)	3 3 (0.1)	4 4 (0.1)	
Chest discomfort	1 1 (0.0)	1 1 (0.0)	2 2 (0.0)	
Hypoxia	1 1 (0.0)	2 1 (0.0)	3 2 (0.0)	
Pulmonary oedema	1 1 (0.0)	0 0 (0.0)	1 1 (0.0)	
Upper respiratory tract infection	1 1 (0.0)	0 0 (0.0)	1 1 (0.0)	

System Organ Class<br>Preferred Term	Care bundle<br>(N=3221)	Usual care<br>(N=3815)	Total<br>(N=7036)	p value*
Acute respiratory failure	0 0 (0.0)	1 1 (0.0)	1 1 (0.0)	
Bronchitis	0 0 (0.0)	1 1 (0.0)	1 1 (0.0)	
Cough	0 0 (0.0)	1 1 (0.0)	1 1 (0.0)	
Dyspnoea	0 0 (0.0)	2 2 (0.1)	2 2 (0.0)	
Lower respiratory tract infection	0 0 (0.0)	1 1 (0.0)	1 1 (0.0)	
Obstructive airways disorder	0 0 (0.0)	1 1 (0.0)	1 1 (0.0)	
Pulmonary embolism	0 0 (0.0)	4 4 (0.1)	4 4 (0.1)	
Pulmonary thrombosis	0 0 (0.0)	1 1 (0.0)	1 1 (0.0)	
Respiratory arrest	0 0 (0.0)	1 1 (0.0)	1 1 (0.0)	
Vascular disorders	15 14 (0.4)	28 28 (0.7)	43 42 (0.6)	
Cerebral haemorrhage	5 5 (0.2)	1 1 (0.0)	6 6 (0.1)	
Hypertension	4 3 (0.1)	7 7 (0.2)	11 10 (0.1)	
Venous thrombosis limb	2 2 (0.1)	1 1 (0.0)	3 3 (0.0)	
Brain stem haemorrhage	1 1 (0.0)	1 1 (0.0)	2 2 (0.0)	
Circulatory collapse	1 1 (0.0)	7 7 (0.2)	8 8 (0.1)	
Intraventricular haemorrhage	1 1 (0.0)	1 1 (0.0)	2 2 (0.0)	
Labile blood pressure	1 1 (0.0)	0 0 (0.0)	1 1 (0.0)	
Aneurysm	0 0 (0.0)	1 1 (0.0)	1 1 (0.0)	
Aortic aneurysm	0 0 (0.0)	1 1 (0.0)	1 1 (0.0)	
Cerebral infarction	0 0 (0.0)	1 1 (0.0)	1 1 (0.0)	
Contusion	0 0 (0.0)	1 1 (0.0)	1 1 (0.0)	
Coronary artery disease	0 0 (0.0)	1 1 (0.0)	1 1 (0.0)	
Deep vein thrombosis	0 0 (0.0)	1 1 (0.0)	1 1 (0.0)	
Post procedural haemorrhage	0 0 (0.0)	1 1 (0.0)	1 1 (0.0)	
Septic shock	0 0 (0.0)	1 1 (0.0)	1 1 (0.0)	
Shock	0 0 (0.0)	2 2 (0.1)	2 2 (0.0)	

System Organ Class<br>Preferred Term	Care bundle<br>(N=3221)	Usual care<br>(N=3815)	Total<br>(N=7036)	p value*
Infections and infestations	9 9 (0.3)	7 7 (0.2)	16 16 (0.2)	
Urinary tract infection	6 6 (0.2)	2 2 (0.1)	8 8 (0.1)	
Acquired immunodeficiency syndrome	1 1 (0.0)	0 0 (0.0)	1 1 (0.0)	
Infection	1 1 (0.0)	0 0 (0.0)	1 1 (0.0)	
Meningitis bacterial	1 1 (0.0)	0 0 (0.0)	1 1 (0.0)	
Post procedural infection	0 0 (0.0)	2 2 (0.1)	2 2 (0.0)	
Pulmonary sepsis	0 0 (0.0)	2 2 (0.1)	2 2 (0.0)	
Sepsis	0 0 (0.0)	1 1 (0.0)	1 1 (0.0)	
Cardiac disorders	8 8 (0.2)	18 17 (0.4)	26 25 (0.4)	
Acute myocardial infarction	2 2 (0.1)	1 1 (0.0)	3 3 (0.0)	
Cardiac failure	2 2 (0.1)	2 2 (0.1)	4 4 (0.1)	
Cardio-respiratory arrest	2 2 (0.1)	2 2 (0.1)	4 4 (0.1)	
Cardiac arrest	1 1 (0.0)	6 5 (0.1)	7 6 (0.1)	
Cardiac disorder	1 1 (0.0)	2 2 (0.1)	3 3 (0.0)	
Atrial fibrillation	0 0 (0.0)	1 1 (0.0)	1 1 (0.0)	
Atrial thrombosis	0 0 (0.0)	1 1 (0.0)	1 1 (0.0)	
Cardiopulmonary failure	0 0 (0.0)	1 1 (0.0)	1 1 (0.0)	
Endocarditis	0 0 (0.0)	1 1 (0.0)	1 1 (0.0)	
Myocarditis	0 0 (0.0)	1 1 (0.0)	1 1 (0.0)	
Surgical and medical procedures	7 7 (0.2)	21 21 (0.6)	28 28 (0.4)	
Cranioplasty	2 2 (0.1)	1 1 (0.0)	3 3 (0.0)	
Hospitalisation	2 2 (0.1)	3 3 (0.1)	5 5 (0.1)	
Haematoma evacuation	1 1 (0.0)	2 2 (0.1)	3 3 (0.0)	
Subdural haematoma evacuation	1 1 (0.0)	0 0 (0.0)	1 1 (0.0)	
Surgery	1 1 (0.0)	2 2 (0.1)	3 3 (0.0)	
Bronchotomy	0 0 (0.0)	2 2 (0.1)	2 2 (0.0)	

System Organ Class<br>Preferred Term	Care bundle<br>(N=3221)	Usual care<br>(N=3815)	Total<br>(N=7036)	p value*
Decompressive craniectomy	0 0 (0.0)	1 1 (0.0)	1 1 (0.0)	
Endotracheal intubation	0 0 (0.0)	1 1 (0.0)	1 1 (0.0)	
Eye excision	0 0 (0.0)	1 1 (0.0)	1 1 (0.0)	
Gastric operation	0 0 (0.0)	1 1 (0.0)	1 1 (0.0)	
Intra-cerebral aneurysm operation	0 0 (0.0)	1 1 (0.0)	1 1 (0.0)	
Intracerebral haematoma evacuation	0 0 (0.0)	5 5 (0.1)	5 5 (0.1)	
Tracheostomy	0 0 (0.0)	1 1 (0.0)	1 1 (0.0)	
Renal and urinary disorders	5 5 (0.2)	15 15 (0.4)	20 20 (0.3)	
Acute kidney injury	3 3 (0.1)	6 6 (0.2)	9 9 (0.1)	
Nephrolithiasis	1 1 (0.0)	2 2 (0.1)	3 3 (0.0)	
Renal impairment	1 1 (0.0)	0 0 (0.0)	1 1 (0.0)	
Azotaemia	0 0 (0.0)	3 3 (0.1)	3 3 (0.0)	
End stage renal disease	0 0 (0.0)	1 1 (0.0)	1 1 (0.0)	
Renal failure	0 0 (0.0)	2 2 (0.1)	2 2 (0.0)	
Urinary retention	0 0 (0.0)	1 1 (0.0)	1 1 (0.0)	
Gastrointestinal disorders	4 4 (0.1)	12 12 (0.3)	16 16 (0.2)	
Diarrhoea	1 1 (0.0)	2 2 (0.1)	3 3 (0.0)	
Gastric ulcer haemorrhage	1 1 (0.0)	0 0 (0.0)	1 1 (0.0)	
Gastrointestinal haemorrhage	1 1 (0.0)	0 0 (0.0)	1 1 (0.0)	
Pancreatitis	1 1 (0.0)	1 1 (0.0)	2 2 (0.0)	
Abdominal discomfort	0 0 (0.0)	2 2 (0.1)	2 2 (0.0)	
Gastric mucosal lesion	0 0 (0.0)	1 1 (0.0)	1 1 (0.0)	
Gastritis	0 0 (0.0)	1 1 (0.0)	1 1 (0.0)	
Gastrointestinal carcinoma	0 0 (0.0)	1 1 (0.0)	1 1 (0.0)	
Intestinal pseudo-obstruction	0 0 (0.0)	1 1 (0.0)	1 1 (0.0)	
Oesophageal carcinoma	0 0 (0.0)	1 1 (0.0)	1 1 (0.0)	

System Organ Class<br>Preferred Term	Care bundle<br>(N=3221)	Usual care<br>(N=3815)	Total<br>(N=7036)	p value*
Pancreatitis acute	0 0 (0.0)	1 1 (0.0)	1 1 (0.0)	
Upper gastrointestinal haemorrhage	0 0 (0.0)	1 1 (0.0)	1 1 (0.0)	
Social circumstances	4 4 (0.1)	2 2 (0.1)	6 6 (0.1)	
Refusal of treatment by relative	4 4 (0.1)	2 2 (0.1)	6 6 (0.1)	
Injury, poisoning and procedural complications	3 3 (0.1)	1 1 (0.0)	4 4 (0.1)	
Fall	2 2 (0.1)	0 0 (0.0)	2 2 (0.0)	
Femur fracture	1 1 (0.0)	0 0 (0.0)	1 1 (0.0)	
Nephropathy toxic	0 0 (0.0)	1 1 (0.0)	1 1 (0.0)	
Musculoskeletal and connective tissue disorders	3 3 (0.1)	4 4 (0.1)	7 7 (0.1)	
Drop attacks	1 1 (0.0)	0 0 (0.0)	1 1 (0.0)	
Femur fracture	1 1 (0.0)	1 1 (0.0)	2 2 (0.0)	
Fracture	1 1 (0.0)	2 2 (0.1)	3 3 (0.0)	
Rib fracture	0 0 (0.0)	1 1 (0.0)	1 1 (0.0)	
Metabolism and nutrition disorders	2 2 (0.1)	2 2 (0.1)	4 4 (0.1)	
Decreased appetite	1 1 (0.0)	0 0 (0.0)	1 1 (0.0)	
Hypokalaemia	1 1 (0.0)	0 0 (0.0)	1 1 (0.0)	
Dehydration	0 0 (0.0)	1 1 (0.0)	1 1 (0.0)	
Hyperglycaemia	0 0 (0.0)	1 1 (0.0)	1 1 (0.0)	
Neoplasms benign, malignant and unspecified (incl cysts and polyps)	2 2 (0.1)	7 7 (0.2)	9 9 (0.1)	
Metastatic neoplasm	1 1 (0.0)	0 0 (0.0)	1 1 (0.0)	
Rectal cancer	1 1 (0.0)	0 0 (0.0)	1 1 (0.0)	
Brain neoplasm	0 0 (0.0)	1 1 (0.0)	1 1 (0.0)	

System Organ Class<br>Preferred Term	Care bundle<br>(N=3221)	Usual care<br>(N=3815)	Total<br>(N=7036)	p value*
Hepatic cancer	0 0 (0.0)	1 1 (0.0)	1 1 (0.0)	
Hepatocellular carcinoma	0 0 (0.0)	3 3 (0.1)	3 3 (0.0)	
Lung cancer metastatic	0 0 (0.0)	1 1 (0.0)	1 1 (0.0)	
Lymphoma	0 0 (0.0)	1 1 (0.0)	1 1 (0.0)	
Psychiatric disorders	2 2 (0.1)	3 3 (0.1)	5 5 (0.1)	
Delirium	1 1 (0.0)	0 0 (0.0)	1 1 (0.0)	
Poor quality sleep	1 1 (0.0)	0 0 (0.0)	1 1 (0.0)	
Anxiety	0 0 (0.0)	2 2 (0.1)	2 2 (0.0)	
Depression	0 0 (0.0)	1 1 (0.0)	1 1 (0.0)	
Investigations	1 1 (0.0)	3 3 (0.1)	4 4 (0.1)	
Oxygen saturation decreased	1 1 (0.0)	1 1 (0.0)	2 2 (0.0)	
Blood glucose decreased	0 0 (0.0)	1 1 (0.0)	1 1 (0.0)	
Blood glucose increased	0 0 (0.0)	1 1 (0.0)	1 1 (0.0)	
Skin and subcutaneous tissue disorders	1 1 (0.0)	2 2 (0.1)	3 3 (0.0)	
Decubitus ulcer	1 1 (0.0)	1 1 (0.0)	2 2 (0.0)	
Skin haemorrhage	0 0 (0.0)	1 1 (0.0)	1 1 (0.0)	
Blood and lymphatic system disorders	0 0 (0.0)	1 1 (0.0)	1 1 (0.0)	
Thrombocytopenia	0 0 (0.0)	1 1 (0.0)	1 1 (0.0)	
Endocrine disorders	0 0 (0.0)	1 1 (0.0)	1 1 (0.0)	
Diabetic complication	0 0 (0.0)	1 1 (0.0)	1 1 (0.0)	
Hepatobiliary disorders	0 0 (0.0)	2 2 (0.1)	2 2 (0.0)	
Cholelithiasis	0 0 (0.0)	1 1 (0.0)	1 1 (0.0)	

System Organ Class<br>Preferred Term	Care bundle<br>(N=3221)	Usual care<br>(N=3815)	Total<br>(N=7036)	p value*
Jaundice	0 0 (0.0)	1 1 (0.0)	1 1 (0.0)	
Immune system disorders	0 0 (0.0)	1 1 (0.0)	1 1 (0.0)	
Alveolitis allergic	0 0 (0.0)	1 1 (0.0)	1 1 (0.0)	
Pregnancy, puerperium and perinatal conditions	0 0 (0.0)	1 1 (0.0)	1 1 (0.0)	
Premature delivery	0 0 (0.0)	1 1 (0.0)	1 1 (0.0)	

Table S23. Causes of death

System Organ Class<br>Preferred Term	Care bundle<br>(N=3221)	Usual care<br>(N=3815)	Total<br>(N=7036)
All Coded Death events: number of patients (%)	361 (11.2%)	556 (14.6%)	917 (13.0%)
Nervous system disorders	163 (5.1%)	274 (7.2%)	437 (6.2%)
Cerebral haemorrhage	142 (4.4%)	215 (5.6%)	357 (5.1%)
Brain herniation	7 (0.2%)	24 (0.6%)	31 (0.4%)
Cerebrovascular accident	4 (0.1%)	11 (0.3%)	15 (0.2%)
Intracranial pressure increased	4 (0.1%)	4 (0.1%)	8 (0.1%)
Ischaemic stroke	2 (0.1%)	8 (0.2%)	10 (0.1%)
Brain stem haemorrhage	1 (0.0%)	3 (0.1%)	4 (0.1%)
Brain stem syndrome	1 (0.0%)	1 (0.0%)	2 (0.0%)
Intraventricular haemorrhage	1 (0.0%)	2 (0.1%)	3 (0.0%)
Thalamus haemorrhage	1 (0.0%)	0 (0.0%)	1 (0.0%)
Brain oedema	0 (0.0%)	3 (0.1%)	3 (0.0%)
Epilepsy	0 (0.0%)	1 (0.0%)	1 (0.0%)
Hydrocephalus	0 (0.0%)	2 (0.1%)	2 (0.0%)
General disorders and administration site conditions	129 (4.0%)	172 (4.5%)	301 (4.3%)
Death	68 (2.1%)	89 (2.3%)	157 (2.2%)
Condition aggravated	52 (1.6%)	62 (1.6%)	114 (1.6%)
Multiple organ dysfunction syndrome	9 (0.3%)	17 (0.4%)	26 (0.4%)
Brain death	0 (0.0%)	1 (0.0%)	1 (0.0%)
Hyperpyrexia	0 (0.0%)	1 (0.0%)	1 (0.0%)
Puncture site haemorrhage	0 (0.0%)	1 (0.0%)	1 (0.0%)
Sudden death	0 (0.0%)	1 (0.0%)	1 (0.0%)

System Organ Class<br>Preferred Term	Care bundle<br>(N=3221)	Usual care<br>(N=3815)	Total<br>(N=7036)
Respiratory, thoracic and mediastinal disorders	46 (1.4%)	70 (1.8%)	116 (1.6%)
Cardio-respiratory arrest	15 (0.5%)	4 (0.1%)	19 (0.3%)
Respiratory failure	13 (0.4%)	37 (1.0%)	50 (0.7%)
Pneumonia	12 (0.4%)	14 (0.4%)	26 (0.4%)
Pneumonia aspiration	3 (0.1%)	7 (0.2%)	10 (0.1%)
Asphyxia	1 (0.0%)	1 (0.0%)	2 (0.0%)
Lung neoplasm malignant	1 (0.0%)	1 (0.0%)	2 (0.0%)
Pulmonary thrombosis	1 (0.0%)	1 (0.0%)	2 (0.0%)
Acute respiratory failure	0 (0.0%)	1 (0.0%)	1 (0.0%)
Pulmonary embolism	0 (0.0%)	3 (0.1%)	3 (0.0%)
Respiratory arrest	0 (0.0%)	1 (0.0%)	1 (0.0%)
Cardiac disorders	9 (0.3%)	14 (0.4%)	23 (0.3%)
Cardiopulmonary failure	6 (0.2%)	5 (0.1%)	11 (0.2%)
Cardiac arrest	1 (0.0%)	4 (0.1%)	5 (0.1%)
Cardiac disorder	1 (0.0%)	1 (0.0%)	2 (0.0%)
Cardio-respiratory arrest	1 (0.0%)	3 (0.1%)	4 (0.1%)
Cardiac failure	0 (0.0%)	1 (0.0%)	1 (0.0%)
Vascular disorders	8 (0.2%)	9 (0.2%)	17 (0.2%)
Cerebral haemorrhage	6 (0.2%)	1 (0.0%)	7 (0.1%)
Circulatory collapse	1 (0.0%)	7 (0.2%)	8 (0.1%)
Myocardial infarction	1 (0.0%)	0 (0.0%)	1 (0.0%)
Intraventricular haemorrhage	0 (0.0%)	1 (0.0%)	1 (0.0%)
Gastrointestinal disorders	2 (0.1%)	3 (0.1%)	5 (0.1%)
Gastric ulcer haemorrhage	1 (0.0%)	0 (0.0%)	1 (0.0%)

System Organ Class<br>Preferred Term	Care bundle<br>(N=3221)	Usual care<br>(N=3815)	Total<br>(N=7036)
Gastrointestinal haemorrhage	1 (0.0%)	0 (0.0%)	1 (0.0%)
Diarrhoea	0 (0.0%)	1 (0.0%)	1 (0.0%)
Gastric mucosal lesion	0 (0.0%)	1 (0.0%)	1 (0.0%)
Gastrointestinal carcinoma	0 (0.0%)	1 (0.0%)	1 (0.0%)
Social circumstances	2 (0.1%)	0 (0.0%)	2 (0.0%)
Refusal of treatment by relative	2 (0.1%)	0 (0.0%)	2 (0.0%)
Neoplasms benign, malignant and unspecified (incl cysts and polyps)	1 (0.0%)	6 (0.2%)	7 (0.1%)
Rectal cancer	1 (0.0%)	0 (0.0%)	1 (0.0%)
Brain neoplasm	0 (0.0%)	1 (0.0%)	1 (0.0%)
Hepatic cancer	0 (0.0%)	1 (0.0%)	1 (0.0%)
Hepatocellular carcinoma	0 (0.0%)	3 (0.1%)	3 (0.0%)
Lung cancer metastatic	0 (0.0%)	1 (0.0%)	1 (0.0%)
Skin and subcutaneous tissue disorders	1 (0.0%)	0 (0.0%)	1 (0.0%)
Decubitus ulcer	1 (0.0%)	0 (0.0%)	1 (0.0%)
Endocrine disorders	0 (0.0%)	1 (0.0%)	1 (0.0%)
Diabetic complication	0 (0.0%)	1 (0.0%)	1 (0.0%)
Infections and infestations	0 (0.0%)	3 (0.1%)	3 (0.0%)
Post procedural infection	0 (0.0%)	1 (0.0%)	1 (0.0%)
Sepsis	0 (0.0%)	2 (0.1%)	2 (0.0%)
Psychiatric disorders	0 (0.0%)	1 (0.0%)	1 (0.0%)
Depression	0 (0.0%)	1 (0.0%)	1 (0.0%)

System Organ Class<br>Preferred Term	Care bundle<br>(N=3221)	Usual care<br>(N=3815)	Total<br>(N=7036)
Renal and urinary disorders	0 (0.0%)	3 (0.1%)	3 (0.0%)
Acute kidney injury	0 (0.0%)	1 (0.0%)	1 (0.0%)
Azotaemia	0 (0.0%)	1 (0.0%)	1 (0.0%)
Renal failure	0 (0.0%)	1 (0.0%)	1 (0.0%)

Figures

Figure S1. Patient enrolment from December 2017 to December 2021, by randomised batches of hospital sites

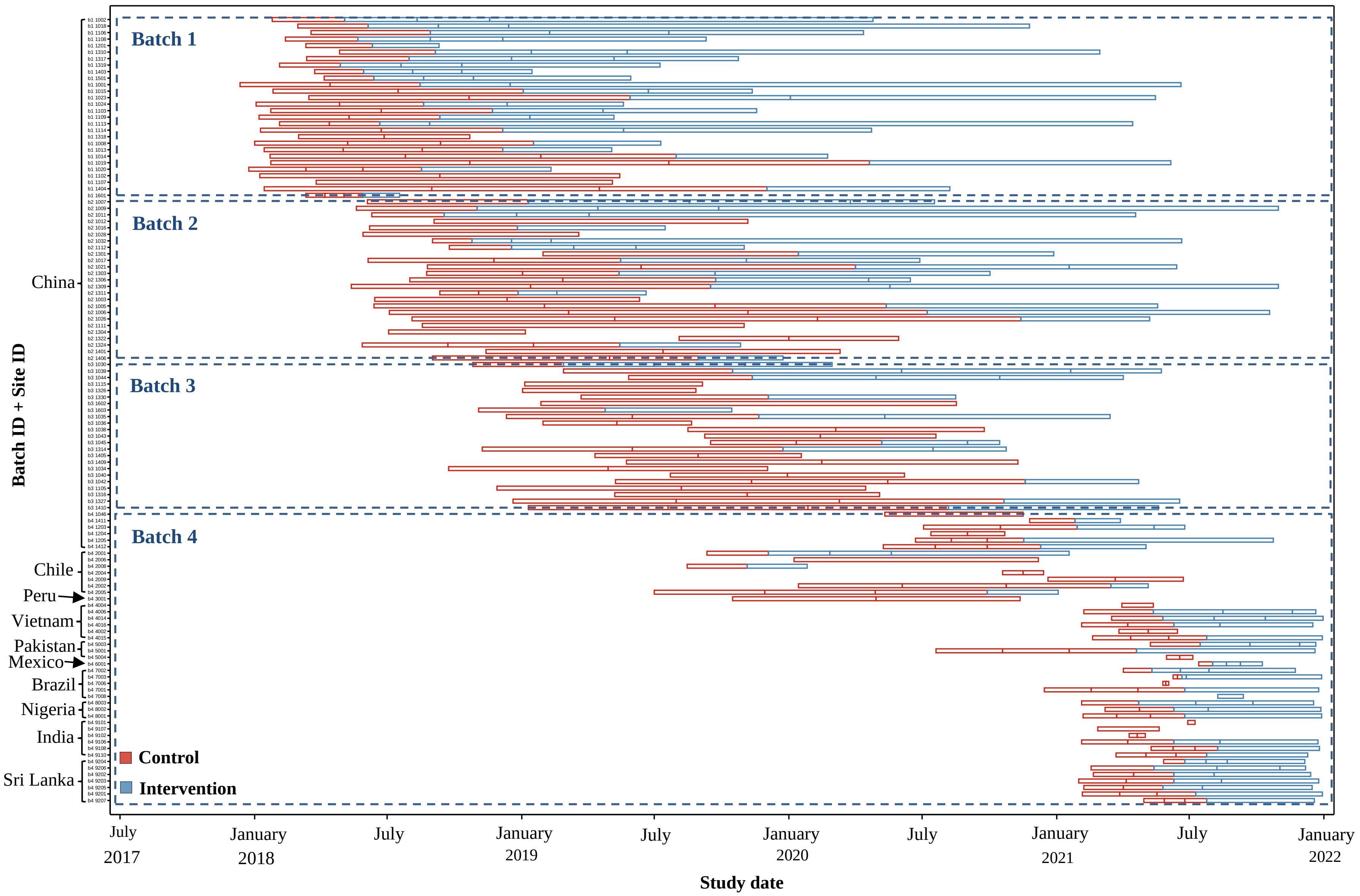

Figure S2. Patient enrolment from December 2017 to December 2021, by date of enrolment for randomised patient

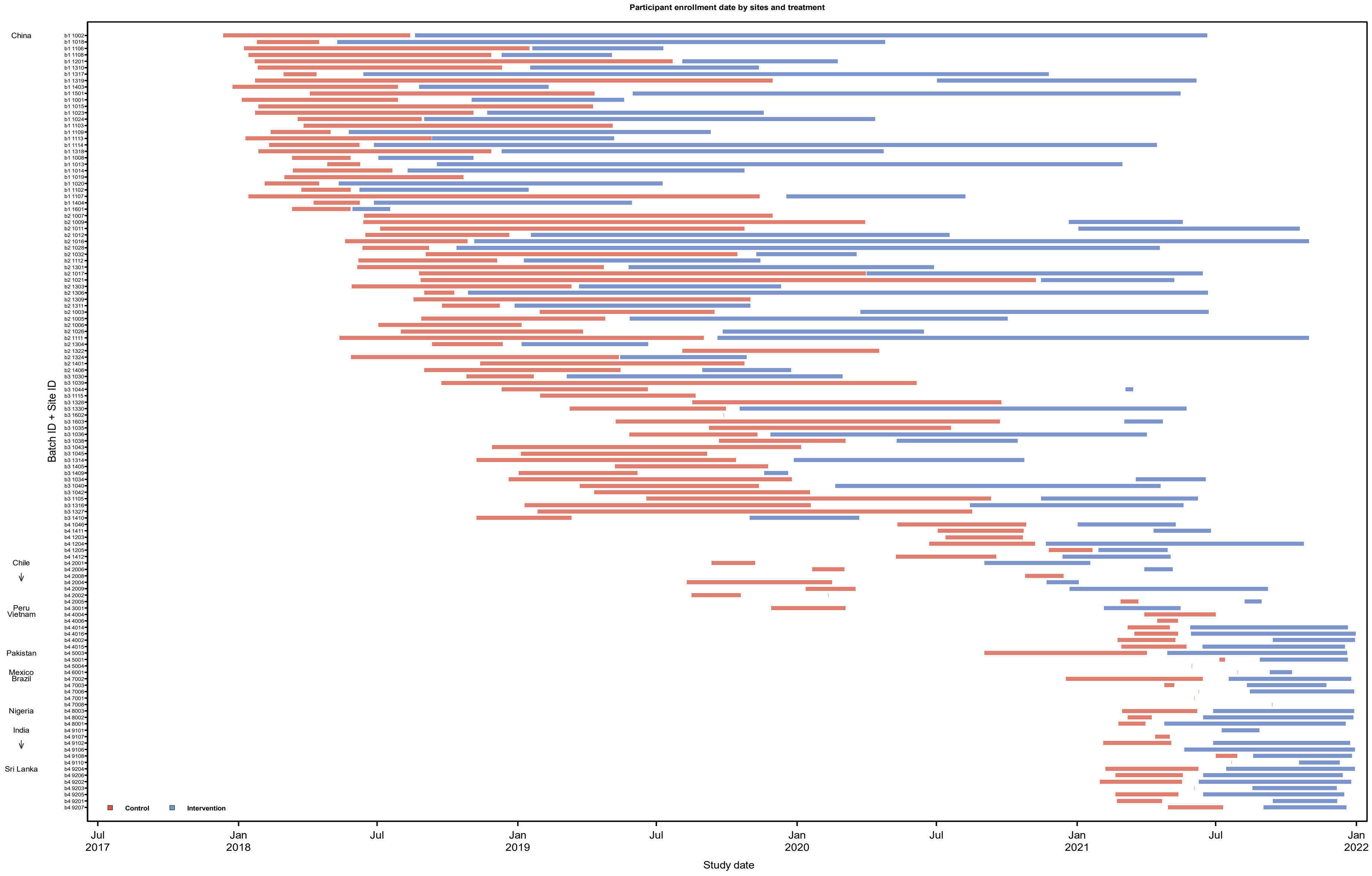

Figure S3. Boxplot of clinician-reported haematoma volume in patients at baseline and follow-up at 24 hours and 7 days

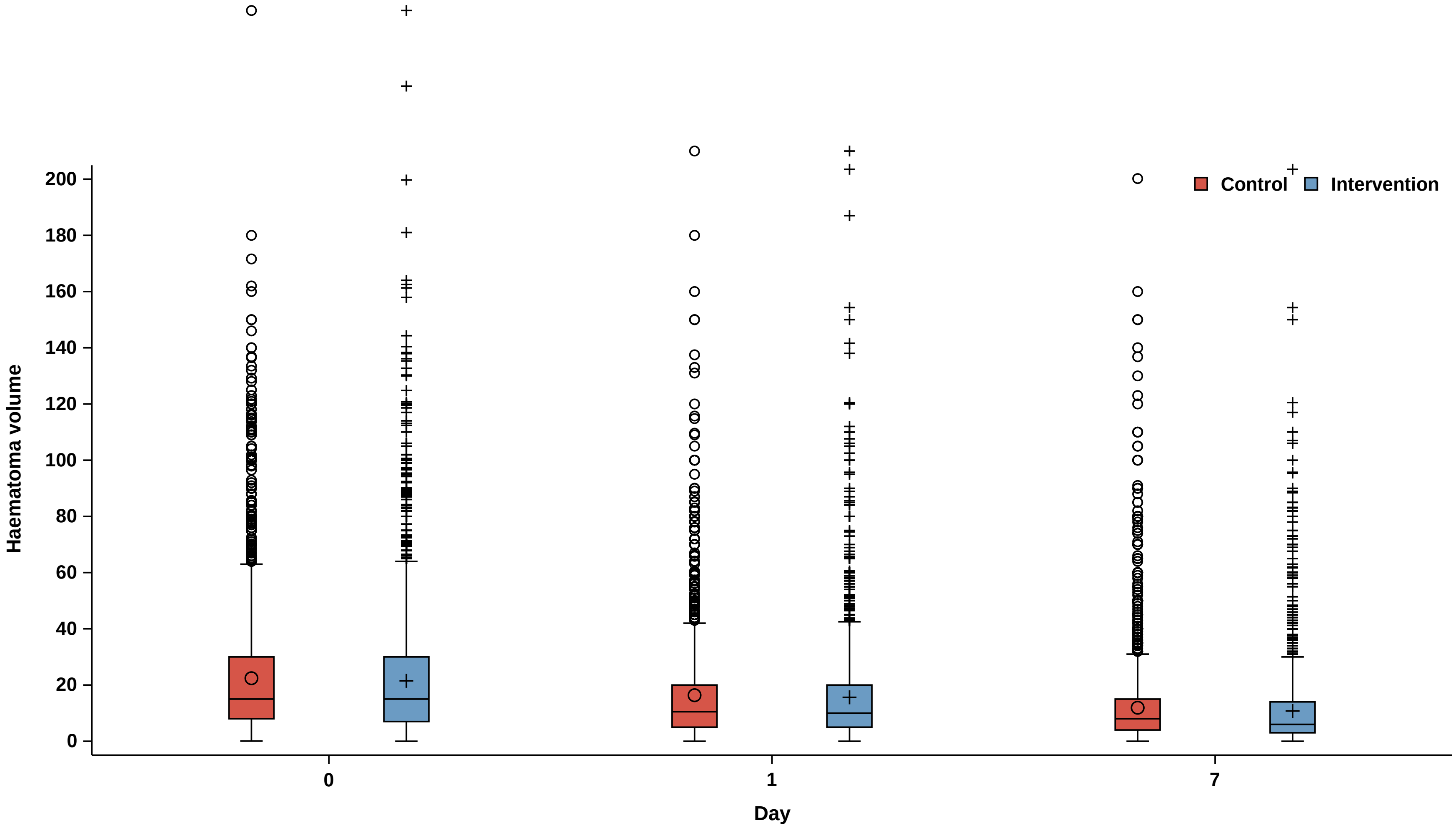

Number Mean (StdDev):			
Control	3523 22.4 (22.49)	2714 16.4 (17.05)	3323 11.9 (14.84)
Intervention	3131 21.5 (22.43)	2433 15.6 (17.21)	2676 10.8 (14.50)

Figure S4. Boxplot of NIHSS by follow-up assessment*

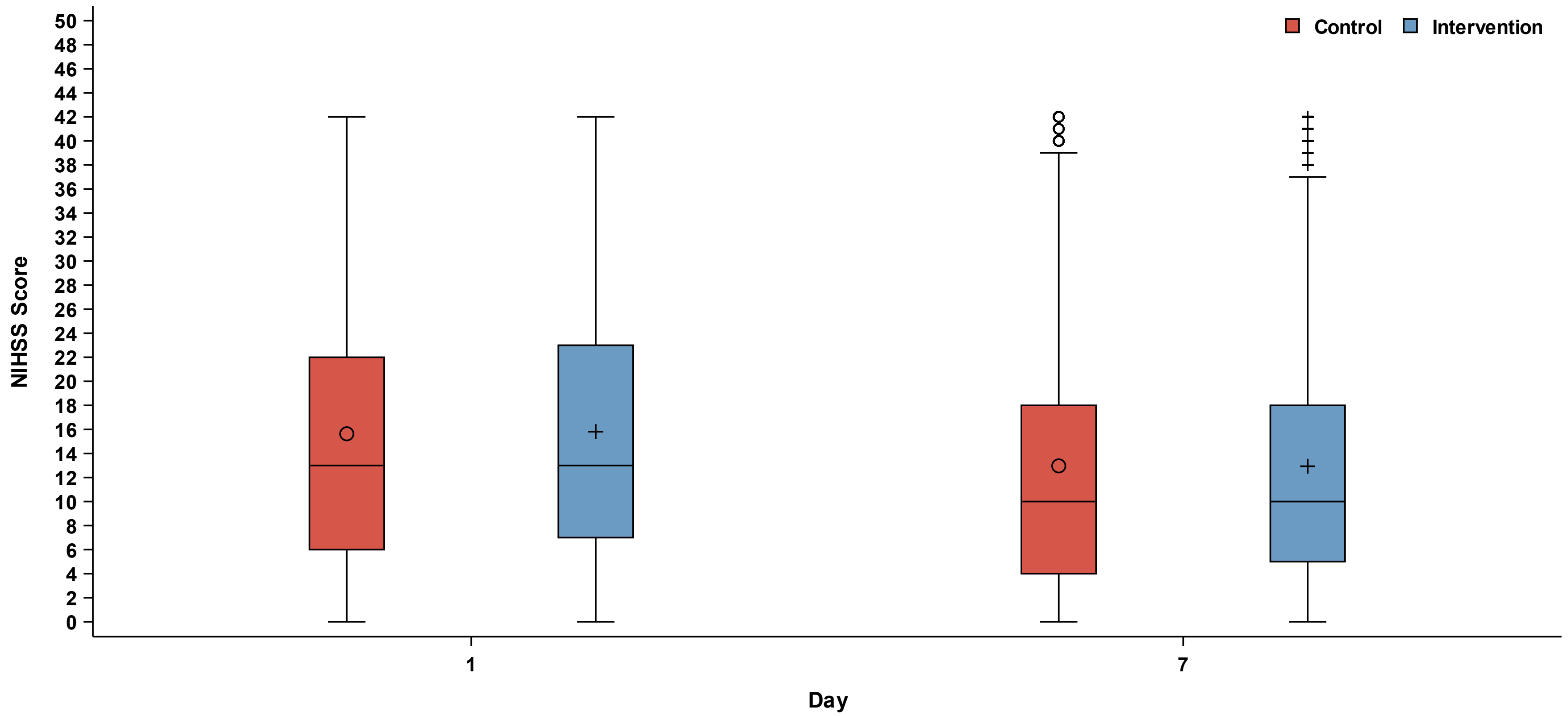

Number Mean (StdDev):		
Control	3693 15.6 (11.46)	3547 13.0 (11.07)
Intervention	3149 15.8 (11.05)	3035 12.9 (10.70)

*NIHSS denotes National Institutes of Health Stroke Scale

Figure S5. Kaplan Meier curve of mortality over 6 months

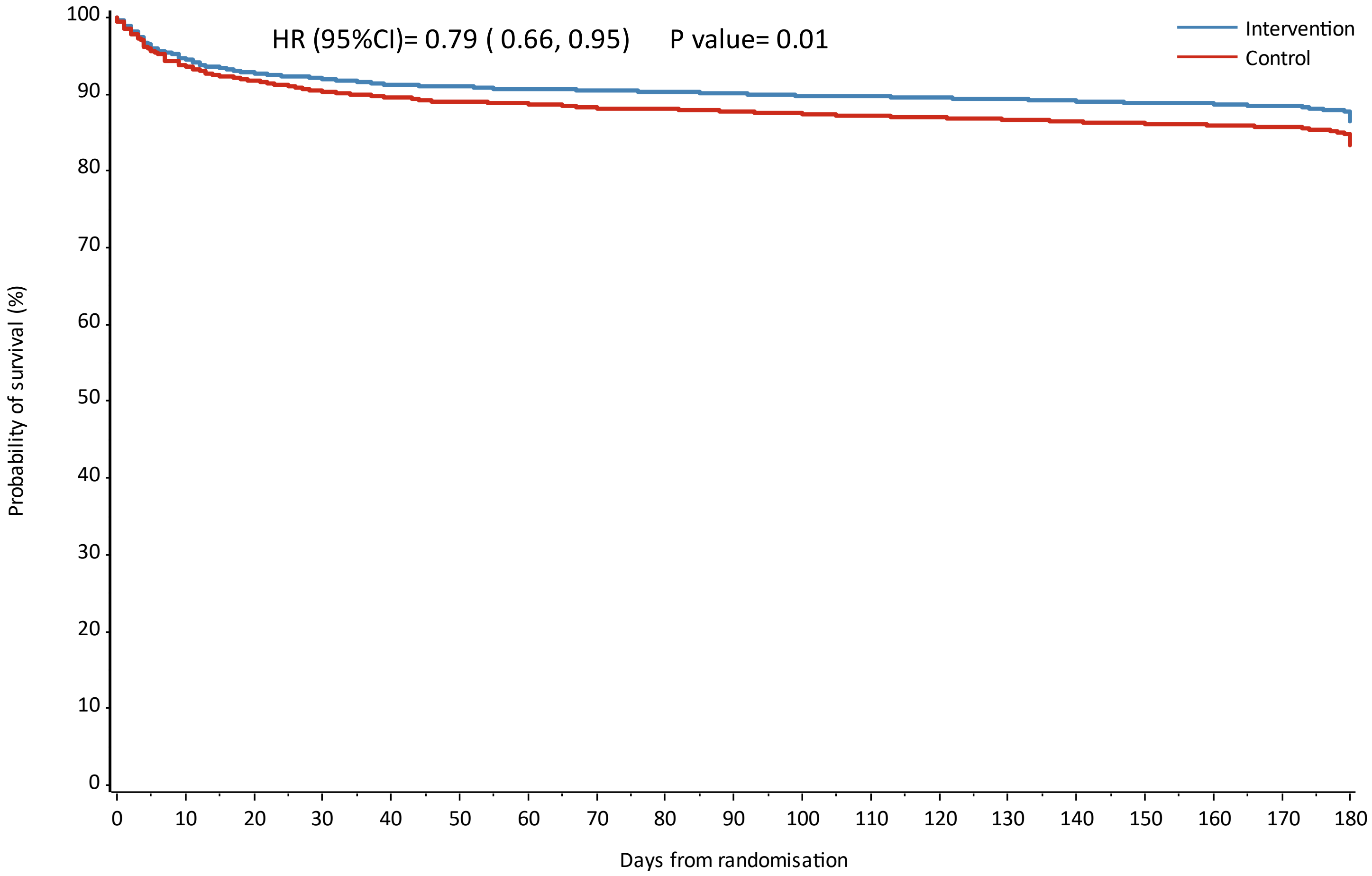

No. at Risk

Control	3420	3228	3158	3112	3084	3062	3054	3036	3030	3019	3010	3001	2992	2982	2973	2966	2958	2948	2920
Intervention	2988	2839	2787	2761	2737	2728	2719	2714	2710	2701	2694	2691	2685	2681	2673	2666	2662	2655	2632

Figure S6. Forest plot for subgroup analysis of primary outcome (shift in scores on the modified Rankin scale) at 6 months*

	Number of subjects	Odds Ratio (95% CI)	p-value for interaction
Age			0.25
<60	2634	0.93 (0.79 , 1.09)	
60-<70	1824	0.88 (0.73 , 1.06)	
70-<80	1298	0.87 (0.70 , 1.08)	
≥ 80	494	0.64 (0.45 , 0.90)	
Sex			0.16
Male	3971	0.90 (0.78 , 1.04)	
Female	2284	0.79 (0.67 , 0.94)	
Country			0.009
China	5587	0.92 (0.80 , 1.06)	
India/Pakistan/Sri Lanka/Vietnam	496	0.93 (0.59 , 1.47)	
Brazil/Peru/Chile/Mexico/Nigeria	172	0.36 (0.20 , 0.67)	
NIHSS			0.07
≥15	2729	0.79 (0.67 , 0.94)	
<15	3354	0.94 (0.81 , 1.10)	
Haematoma volume			0.62
<15 ml	2832	0.90 (0.77 , 1.05)	
15-<30 ml	1560	0.87 (0.72 , 1.06)	
≥30ml	1520	0.80 (0.65 , 0.98)	
Haematoma location			0.11
Cortical	568	1.14 (0.83 , 1.56)	
Deep	4934	0.82 (0.72 , 0.94)	
Brainstem/Cerebellum	587	0.95 (0.69 , 1.30)	
Covid			0.013
Before 30Jan2020	4398	0.96 (0.82 , 1.11)	
After 30Jan2020	1857	0.69 (0.56 , 0.85)	

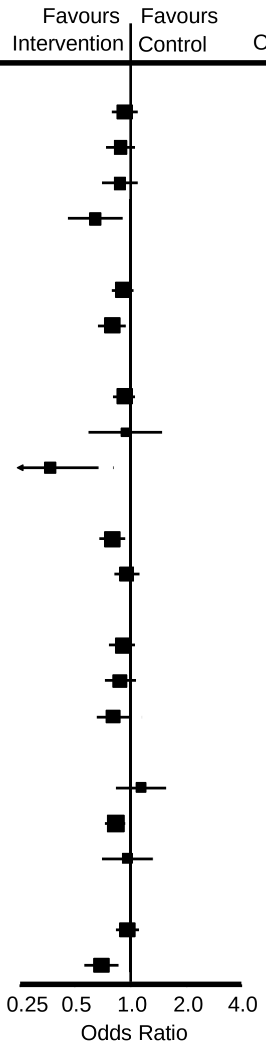

*CI denotes confidence interval, mRS modified Rankin scale, NIHSS National Institutes of Health Stroke Scale

Figure S7. Forest plot for subgroup analysis of primary outcome (shift in scores on the modified Rankin scale) at 6 months, China sites only*

	Number of subjects	Odds Ratio (95% CI)	p-value for interaction
Age			0.393
<60	2338	0.99 (0.83 , 1.18)	
60-<70	1641	0.96 (0.79 , 1.17)	
70-<80	1166	0.94 (0.74 , 1.19)	
80+	439	0.71 (0.49 , 1.02)	
Sex			0.476
Male	3514	0.95 (0.82 , 1.11)	
Female	2073	0.89 (0.74 , 1.07)	
NIHSS			0.029
>=15	2529	0.84 (0.70 , 1.00)	
<15	2982	1.04 (0.88 , 1.23)	
Haematoma volume			0.317
<15 ml	2523	1.02 (0.86 , 1.21)	
15-<30 ml	1456	0.94 (0.77 , 1.15)	
>=30ml	1392	0.84 (0.68 , 1.04)	
Haematoma location			0.104
Cortical	448	1.31 (0.92 , 1.87)	
Deep	4436	0.90 (0.78 , 1.03)	
Brainstem/Cerebellum	540	0.96 (0.69 , 1.34)	
Covid			0.120
Before 30Jan2020	4383	0.97 (0.84 , 1.13)	
After 30Jan2020	1204	0.75 (0.56 , 1.01)	

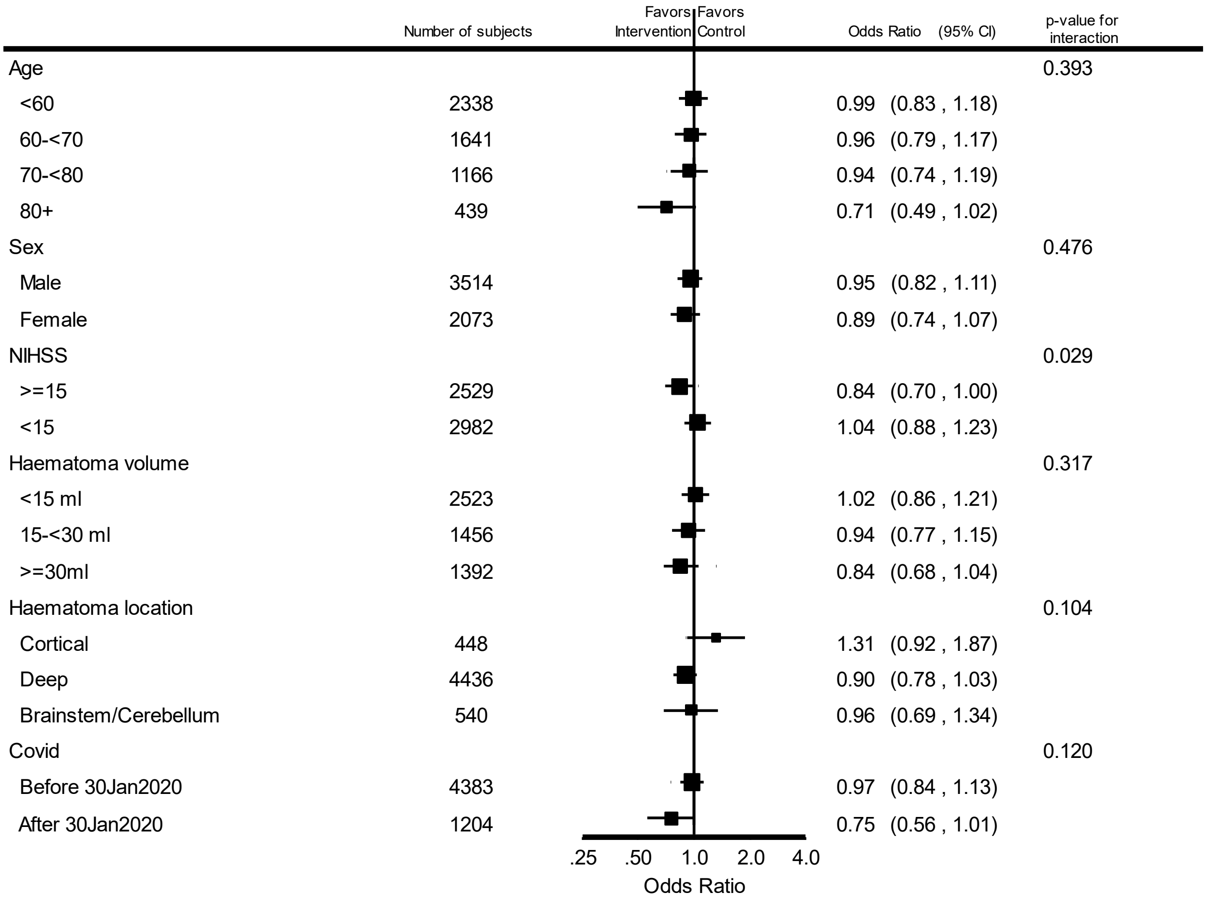

*CI denotes confidence interval, mRS modified Rankin scale, NIHSS National Institutes of Health Stroke Scale

Figure S8. Blood pressure plots by region

(a) China

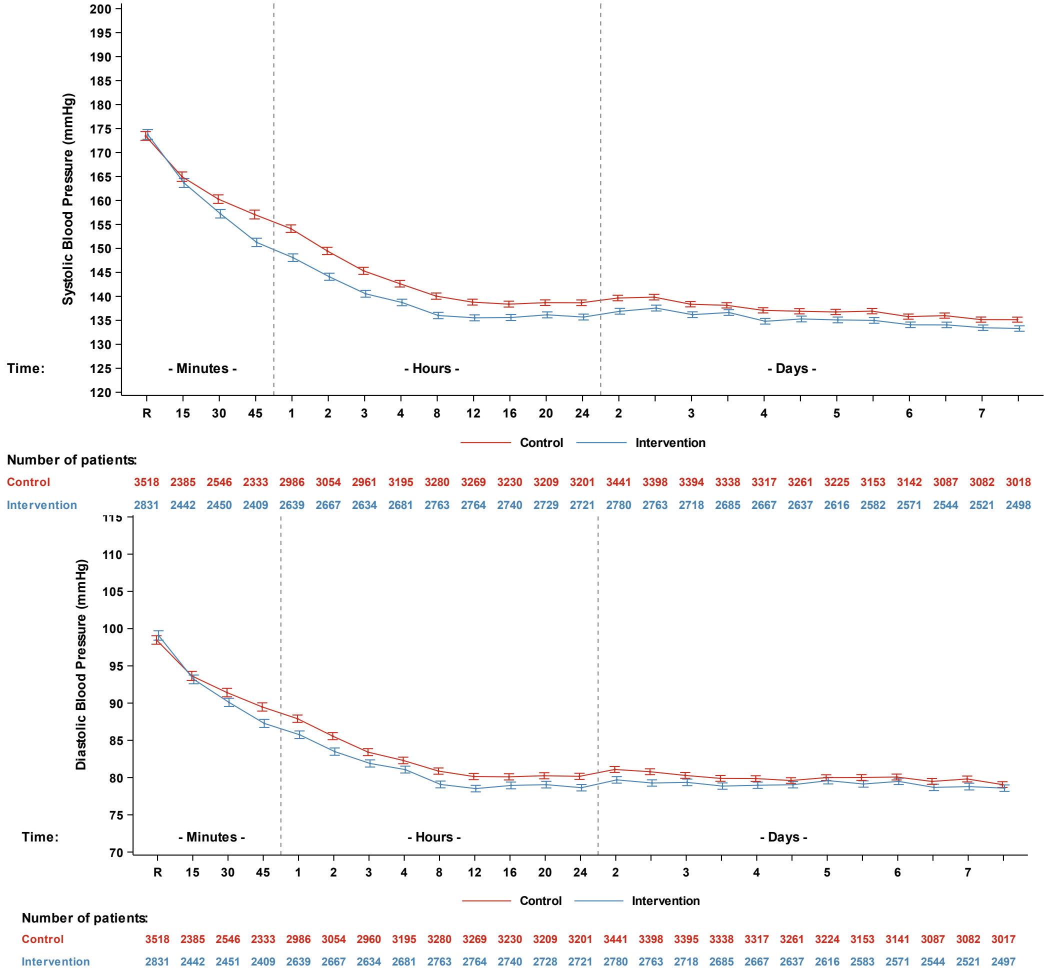

(b) India/Pakistan/Sri Lanka/Vietnam

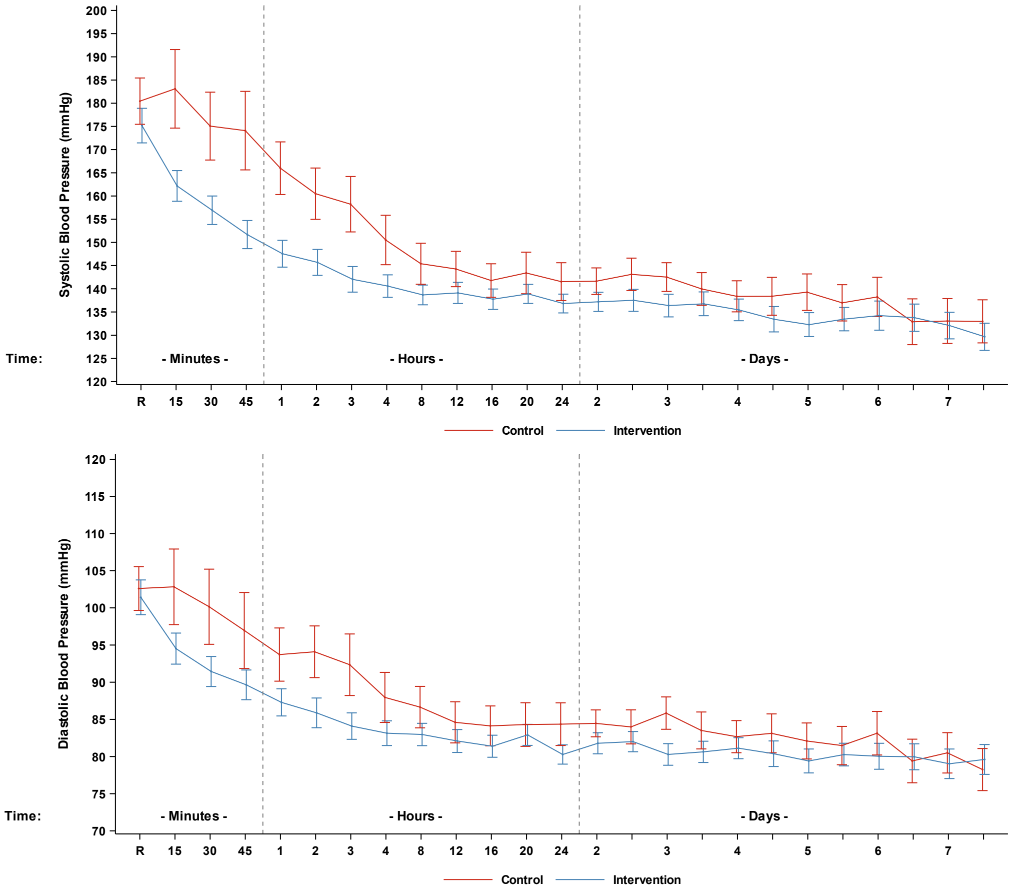

Number of patients:

Control	181	55	57	56	100	104	82	97	111	100	105	92	73	195	143	172	118	137	92	107	69	79	52	58	44
Intervention	285	273	275	276	279	284	284	284	284	280	278	274	273	270	253	242	206	200	174	168	148	143	123	117	100

(c) Brazil/Peru/Chile/Mexico/Nigeria

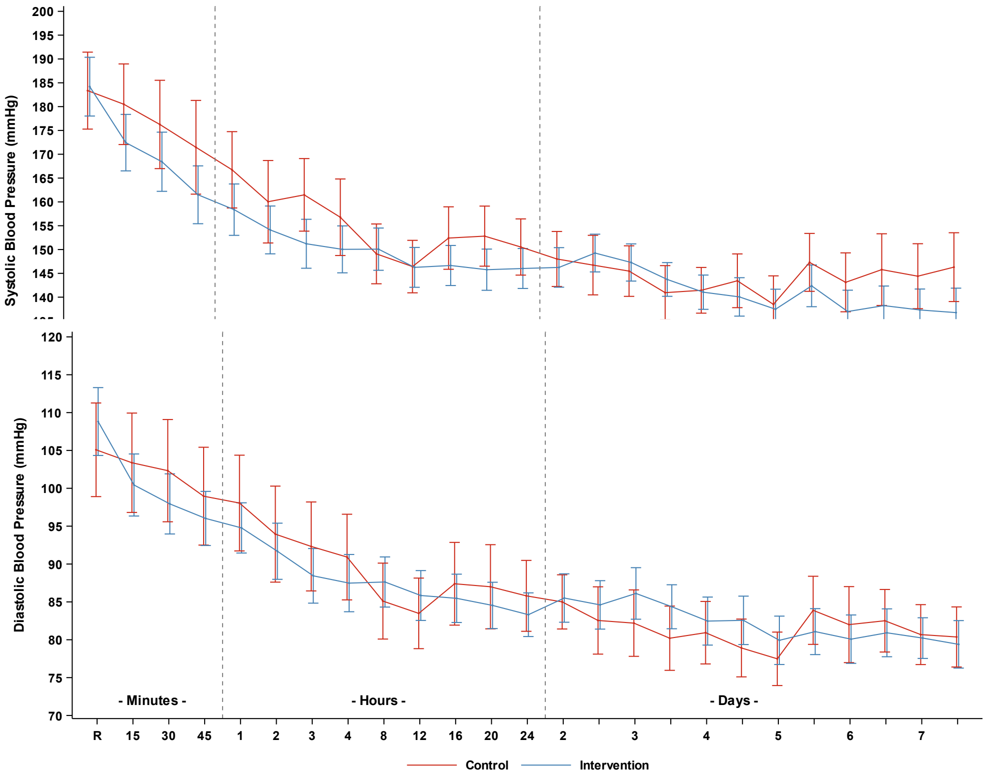

Number of patients:

	R	15	30	45	1	2	3	4	8	12	16	20	24	2		3		4		5		6		7	
Control	69	56	51	52	57	60	59	63	66	64	61	64	64	63	64	61	59	59	59	58	53	51	51	47	46
Intervention	105	100	101	100	100	102	100	102	102	102	100	99	97	98	96	95	92	90	87	81	79	76	74	73	71

19. Study protocol

INTEnsive *care bundle with blood pressure* Reduction in Acute Cerebral haemorrhage Trial

An investigator initiated and conducted, international, multicentre, stepped wedge cluster randomised study of a care bundle of physiological control strategies in acute intracerebral haemorrhage

STUDY PROTOCOL

(Version 3.0– 12 August 2019)

CONTACT DETAILS

INTERACT3 Central Coordinating Centre

The George Institute for Global Health, China

Tel +8610 8280 0577

Fax +8610 8280 0177

Email: INTERACT3@georgeinsitute.org.cn

This protocol has been developed by the INTERACT3 Steering Committee and its contents are the intellectual property of this group. It is an offence to reproduce or use the information and data in this protocol for any purpose other than the INTERACT3 study without prior approval from the project office of the INTERACT3 study.

NVESTIGATOR AGREEMENT

I have read the following protocol:

Protocol Title: The third, INTEnsive care bundle with blood pressure Reduction in Acute Cerebral haemorrhage Trial

Version and Date: Version 3.0– 12 August 2019

I have read this protocol and associated procedure manuals and agree that it contains all the necessary details for carrying out the study. I will conduct the study as outlined herein and will complete the study within the time designated.

I will provide copies of the protocol and all pertinent information to all individuals responsible to me who assist in the conduct of the study. I will discuss this material with them to ensure that they are fully informed regarding the study intervention and the conduct of the study.

____

Investigator's Signature Date (Day / Month / Year)

____

Name of Investigator (Printed)

____

Name of Institution (Printed)

Principal Investigator

**Co-Principal Investigator**<br>Professor Craig Anderson<br>The George Institute for Global Health, China	12 August 2019<br>*Date*	*Signature*
**Co-Principal Investigator**<br>Professor Chao You<br>Department of Neurosurgery, West China Hospital, Sichuan University	12 August 2019<br>*Date*	*Signature*
**Trial Statistician**<br>A/Professor Laurent Billot<br>The George Institute for Global Health, Australia	12 August 2019<br>*Date*	*Signature*

1. CONTENTS

1. CONTENTS **91**
2. ADMINISTRATIVE INFORMATION **94**
Protocol History 94
Trial/Study Registration 94
Funding 94
Study Management & Oversight 94
Glossary of Abbreviations and Terms 96
3. PROTOCOL SYNOPSIS **98**
4. BACKGROUND AND RATIONALE **99**
Overview 99
Background 99
Global burden of ICH 99
Progress in treatment strategies for ICH 99
BP and outcome in ICH 99
Glucose and outcome in ICH 102
Body temperature and outcome in ICH 102
Reversal of anticoagulation 103
Early seizures and outcome of ICH 104
Advantages of cluster clinical trials for evaluating complex systems of care interventions 104
Summary 105
5. AIMS AND HYPOTHESES **105**
Primary aim: 105
Secondary aims: 105
6. METHODS **106**
Overall design 106
Study population 107
Inclusion and exclusion criteria 108
Ethical issues 108
Institutional ethics committee approval and consent 108
Definition of participant and assessment of selection bias 111
Confidentiality and privacy 111
Study site selection, participation and responsibilities of staff 111
Intervention 113
Goal-directed care bundle of active management group 113
Study outcomes 116

Data collection and follow-up 116

Screening and Enrolment log (Form A) 116

Patient Contact Details log 117

Baseline (Form B) 117

Day 1 (Form C) 117

BP and 72 hours BG / T Monitoring Chart (Form D and E) 117

Day 7 or discharge if earlier (Form F) 118

6 months Follow-up Blinded Assessment (Forms G) 118

7. SAFETY 119

Data and Safety Monitoring Board (DSMB) 119

Serious Adverse Events (SAEs) 119

Recording and Reporting 120

8. QUALITY ASSURANCE 120

Monitoring of sites 121

Collection and Storage of Essential Documents 121

9. PROCESS EVALUATION 122

10. ECONOMIC EVALUATION 123

11. DATA MANAGEMENT 123

12. STATISTICAL CONSIDERATIONS 124

Sample Size 124

13. SUBSTUDY (SELECTED SITES ONLY) 125

Effects of treatment of haematoma Change in ICH 125

14. PUBLICATIONS, REPORTS AND DATA SHARING 125

15. STUDY ORGANISATION 126

Participating Sites 126

Study Organisational Chart 127

16. FUNDING 127

17. TIMELINES 128

18. REFERENCES 129

APPENDIX 1 - WORLD MEDICAL ASSOCIATION DECLARATION OF HELSINKI 133

APPENDIX 2 - BP management protocol 138

Appendix 2A - BP protocol for centres with Urapidil (China) 138

Appendix 2B - BP protocol for centres with Phentolamine (China) 140

Appendix 2C - BP management protocol WITHOUT Labetalol (out of China) 142

Appendix 2D - BP management protocol for centres WITH labetalol (out of China) 144

Appendix 2E- BP protocol for centres with Clevidipine (out of China) 146

Appendix 2F - Additional IV Medication for BP Use in China 148

APPENDIX 3 – Health Scales 150

Glasgow Coma Scale (GCS) 150

NIH Stroke scale (National Institute of Health Stroke Scale) 151

Modified Rankin scale (mRS) 156

Simplified mRs 157

EQ- 5D 158

APPENDIX 4 – Process evaluation interview guides and templates 160

Process Evaluation Interview Guide 160

Non-participant observation documentary notes 162

Focus group discussion guide 164

Patient Interview Guide 167

2. ADMINISTRATIVE INFORMATION

Protocol History

Version Number	Version Date	Summary of Revisions Made:
1.1	12 July 2017	Original
2.0	8 April 2018	1. Extend the time limitation for each phase to 4 months (only in Asia); 2. Additional funding source from Takeda China; 3. Set up a transition period of 7-10 days before entering intervention phases; 4. Change to randomization program stratified by both country and size of site; 5. Adjust the study timelines.
3.0	12 August 2019	1. Expand to 110 sites and set organisation of RCCs; 2. Extend the study timeline to 2022; 3. Add process evaluation and economic evaluation alongside the trial; 4. Add inclusion criteria of ICH diagnosis within 6 hours from onset; 5. Change opt-out approach to consent withdrawal; 6. Recalculate sample size with target an average of 19 patients per site per phase for a total sample size of 8,360 patients;

Trial/Study Registration

The study has been registered with ClinicalTrials.gov (NCT03209258), and Chinese Trial Registry (ChiCTR-IOC-17011787).

Funding

This study receives its principle funding from the following grants:
1) The West China Hospital Outstanding Discipline Development 1-3-5 Program (ZY2016102)
2) Program Grant from the National Health and Medical Research Council (NHMRC) of Australia (APP1149987)

Funding is also from Sichuan Credit Pharmaceutical CO., LTD and Takeda (China) International Trading Co., Ltd.

Study Management & Oversight

INTERACT3 is an investigator initiated and conducted study managed by The George Institute for Global Health in Beijing, China (GI China). The study will be overseen by a committee of international experts in the fields of neurology, neurosurgery, hypertension, cardiovascular disease, epidemiology and clinical trials.

Steering Committee (SC): The Steering Committee will comprise of the principle investigators, national leaders from all countries, and expert academic researchers, with the chairman held by an independent SC member. The SC is responsible for the execution of the study design, protocol, data collection and analysis plan, as well as publications. The SC has the right to appoint new members and co-opt others to add to the integrity of the conduct of the study and analyses. The SC will also include the grant holders and its members are to be confirmed. The steering committee charter will govern the steering committee.

Central Coordinating Centre (CCC): The CCC is based at GI China and is supported by project staff. *Responsibilities:* Day-to-day management of the study, data and project management, committee coordination, assistance with ethics committee and regulatory applications, protocol and procedures for training of participating sites, overseeing of initiation visits and activation of participating centres, monitoring of data quality and adherence to protocol, applicable guidelines and regulations, preparation of study data for analysis and publication.

Glossary of Abbreviations and Terms

ACE-I	Angiotensin converting enzyme inhibitor
ARB	Angiotensin II receptor blocker
BG	Blood glucose
BP	Blood pressure
CCB	Calcium channel blocker
CCC	Central coordinating centre
CCT	Cluster controlled trial
CI	Confidence interval
CRF/eCRF	Case record form/electronic CRF
CSVD	Cerebral small vessel disease
CT	Computed tomography
CV	Cardiovascular
DALY	Disability-adjusted-life-years
DSMB	Data and safety monitoring board
EC	Ethics committee
ECG	Electrocardiogram
ED	Emergency department
EQ-5D	EuroQoL Group 5-dimension self-report questionnaire
FFP	Fresh frozen plasma
GCP	Good Clinical Practice
GCS	Glasgow coma scale
GEE	Generalised estimating equations
GI China	The George Institute for Global Health, China
GOS	Glasgow outcome scale
GOSE	Extended Glasgow outcome scale
HOQ	Hospital organisation questionnaire
HR	Heart rate
HREC	Human research ethics committee
HRQoL	Health-related quality of life
IC	Informed consent
ICH	Intracerebral haemorrhage

ICH-GCP	ICH Guidelines for Good Clinical Practice
ICMJE	International Committee of Medical Journal Editors
ICP	Intracranial pressure
ICU	Intensive care unit
INR	International normalised ratio
INTERACT2	The second phase, INTEnsive blood pressure Reduction in Acute Cerebral haemorrhage Trial
INTERACT3	The third, INTEnsive care bundle with blood pressure Reduction in Acute Cerebral haemorrhage Trial
IRB	Institutional review board
LFTs	Liver function tests
MRI	Magnetic resonance imaging
mRS	Modified Rankin scale
NIHSS	National Institute of Health Stroke Scale
NYHA	New York Heart Association
OC	Operations committee
OR	Odds ratio
PCC	Prothrombin complex concentrate
PI	Principal investigator
PIS	Patient information sheet
RCC	Regional Coordinating Centre
RR	Risk ratio
SAE	Serious adverse event
SAP	Statistics analysis plan
SBP	Systolic BP
SC	Steering committee
SIV	Site initiation visit
SOP	Standard Operation Process
TGI	The George Institute for Global Health
TIA	Transient ischaemic attack
TMF	Trial master file
TTM	Targeted temperature management
UAR	Unexpected adverse reaction

3. PROTOCOL SYNOPSIS

**Main Sponsors:** The George Institute for Global Health China; West China Hospital, Chengdu	**Trial Registration number: Clinicaltrials.gov (NCT03209258)**
**Title of Study:** The third, INTEnsive care bundle with blood pressure Reduction in Acute Cerebral haemorrhage Trial (INTERACT3)	
**Study Duration:** 4 years	**Clinical Phase:** IV
**Objectives:** To determine the effectiveness of a goal-directed care bundle of active management involving early physiological control (intensive blood pressure [BP] lowering, glycemic control, and early treatment of pyrexia) and reversal of anticoagulation, versus usual standard of care, on functional outcome in patients with acute spontaneous intracerebral haemorrhage (ICH).	
**Number of planned participants:** 110 hospital sites, 8360 patients	
**Study Design:** International, multicentre, stepped wedge (4 phases/3 steps), cluster randomised clinical trial to assess a multifaceted package of care in a broad range of patients with acute ICH.	
A cluster clinical trial design involves implementation of a guideline-recommended intervention package applied to patients with ICH as part of routine care. Patients are only excluded if they refuse to have details of their management included and/or participate in the follow-up procedures.<br>**Study site inclusion criteria:** Organised systems of acute stroke care; no established comprehensive protocols for the management of ICH; suitable location, infrastructure and willingness to participate in clinical research; large volume of ICH patients (approx. 100 per year).<br>**Patient inclusion criteria:** Adults (≥18 years) with acute stroke due to primary ICH confirmed by imaging (copy of the brain imaging report to be submitted to the CCC labelled with study ID and with personal identifiers removed) and admitted hospital within 6 hours of the onset of symptoms.	
**Outcome measures**<br>Primary outcome: Functional recovery according to an ordinal shift analysis of the full range of scores on the modified Rankin scale (mRS) scores at 6 months.<br>Secondary outcomes: Functional recovery according to a shift analysis of scores on the National Institutes of Health Stroke Scale (NIHSS) at 7 days. The following at 6-months: poor outcome defined by mRS scores of 3-6; separately on death and disability (mRS 3-5); health-related quality of life (HRQoL) using the EuroQoL Group 5-Dimension self-report questionnaire (EQ-5D); duration of hospitalisation; and residence.<br>Safety: Serious adverse events during follow-up.	
**Study Intervention**<br>**Usual care group:** For patients in the usual-care group, decisions about the location of care delivery, investigations, monitoring, and all treatments will be made by the treating clinical team. Data will be collected regarding the management of patients, including insertion of invasive monitoring devices, intravenous-fluid resuscitation, BP lowering, vasoactive support, glycaemic control, mechanical ventilation, neurosurgery, and other supportive therapy.<br>**Interventional group:** The project team will use a range of implementation methods to introduce a goal-directed care bundle that involves the rapid correction (<1 hour) of physiological variables as soon as the abnormality is recognised and for the control to be maintained in patients for 7 days or hospital discharge (or death, if sooner):<br>(1) intensive BP lowering to systolic target of <140mmHg;<br>(2) glucose control target 6.1-7.8 mmol/l for non-diabetic; 7.8-10.0 mmol/l for diabetic patients;<br>(3) treatment of pyrexia to a target body temperature ≤37.5 ℃;<br>(4) reversal of anticoagulation to target INR <1.5 involving use of vitamin K and prothrombin complex concentrate (PCC) or alternatively, fresh frozen plasma (FFP).	

4. BACKGROUND AND RATIONALE

Overview

The fundamental principle underlying the medical management of acute intracerebral haemorrhage (ICH) is *an active process of early control of abnormal physiological parameters* through appropriate and timely administration of specific drugs and resuscitation measures. INTERACT3 aims to evaluate the effectiveness of implementing a goal-directed, multi-facetted, care bundle of physiological management in patients with acute ICH.

Background

Global burden of ICH

Acute spontaneous non-traumatic ICH is the most severe and least treatable type of stroke, which affects several million people in the world each year.[1, 2] ICH accounts for 10% of strokes in high-income countries, but up to 50% in low- and middle-income countries, where the prevalence of hypertension is high.[1, 3] In contrast to declines in rates of acute ischaemic stroke, the incidence of ICH has remained stable in recent decades and its outcome remains poor, with about two thirds of patients either dying or being disabled by the disease.[1] On a global scale, ICH accounts for a greater lost disability-adjusted-life-years (DALYs; lost-productive-life-years) than that of acute ischaemic stroke, because of poor outcomes and tendency to affect people of working age.[2] ICH survivors remain at very high risk of repeat CV events, with the risk of recurrent ICH and other serious ischaemic cerebral or cardiac CV events totalling about 10% per annum[4]. The frequency of cognitive decline and dementia is also high, due to the common underlying vasculopathy of cerebral small vessel disease (CSVD).[5]

Progress in treatment strategies for ICH

Despite the heavy burden of ICH, progress has been slow in established clear evidence of benefit for specific management strategies. The role of decompressive surgery, for example, varies around the world depending the availability and organisation of services, and acceptance of the large body of randomised evidence which has not clearly defined the characteristics of patients who have the most to gain from such intervention. The medical management of ICH is, therefore, primarily supportive, directed at providing a systematic clinical assessment that includes ensuring there is no underlying treatable structural lesion; good control of blood pressure (BP) and other physiological parameters including reversal of anticoagulation; early management of complications; rehabilitation to promote recovery; and effective secondary prevention. In particular, there is good evidence of potential beneficial effects of early intensive BP lowering therapy on functional recovery,[6] tight glucose control using insulin infusions in mainly surgical critical care patients,[7] a structured assessment and management of several parameters (managing glucose, fever, and swallowing dysfunction).[8]

BP and outcome in ICH

Significance of elevated BP in acute stroke Elevated BP or 'hypertension' (ie systolic >140mmHg) is very common after ICH (at least 60% of cases), and it is a strong predictor of haematoma growth, perihaematomal oedema, and subsequent neurological worsening and poor outcomes, including death and disability.[9, 10] Multiple potential pathophysiological mechanisms may account for elevated BP (e.g. stress, pain, pre-existing hypertension, and increased intracranial pressure).

The main phase, Intensive Blood Pressure Reduction in Acute Intracerebral Haemorrhage Trial (INTERACT2) INTERACT2 was a landmark study which resolved much of the uncertainty over the management of elevated BP in ICH and, for the first time, provided support for an effective medical strategy.[6] The study included 2839 patients who were randomly assigned to intensive (to a target systolic level of <140 mmHg within 1 hour) or guideline-recommended (target systolic level <180 mmHg) BP lowering treatment within 6 hours of ICH. Among 2794 participants for whom the primary outcome of death and major disability could be determined, 719 (52.0%) of 1382 participants receiving intensive treatment, as compared with 785 (55.6%) of 1412 receiving guideline-recommended treatment, had a primary outcome (odds ratio [OR] 0.87, 95% confidence interval [CI], 0.75 to 1.01; P=0.06). However, in the intensive group, there was significantly better functional recovery at 90 days according to an ordinal analysis of the modified Rankin scale (mRS) scores (OR for greater disability, 0.87; 95% CI 0.77 to 1.00; P=0.04) and participants reported better health-related quality of life (HRQoL). Moreover, intensive BP lowering was shown to be safe, with no difference in mortality (12.0%) or serious adverse events (SAEs) between randomised groups.

The second Antihypertensive Treatment for Acute Cerebral Hemorrhage (ATACH-II) study The recently completed ATACH-II study[11] compared ‘very early’ (<4.5 hours of onset) and ‘very rapid and intensive’ (systolic BP <140 mmHg with intravenous nicardipine for 24 hours) BP lowering with standard BP management (systolic BP of 140-180 mmHg). Death or disability (mRS scores 4 to 6; the primary outcome) at 90 days was 38.7% in the very intensive and 37.7% in the standard BP lowering groups (adjusted relative risk [RR] 1.04, 95%CI 0.85-1.27; P=0.72). While there was no overall significant difference in treatment related SAEs within 72 hours, significantly more renal adverse events emerged over the initial 7 days (9.0% vs. 4.0%; P=0.002) and borderline more SAEs during 90 days in the very intensive group (adjusted RR 1.30, 95%CI 1.00-1.69; P=0.05). Interestingly, the percentage of patients with haematoma growth (defined as >33% increase in ICH volume over the initial 24 hours) was lower (18.9%) in the very intensive group than in the standard treatment group (24.4%; adjusted RR 0.78, 95%CI 0.58-1.03; P=0.08).

Ongoing uncertainties over the efficacy of early intensive BP lowering in ICH The main results of INTERACT2 with respect to the primary outcome may not be considered as robust as some people may wish. However, if formed the basis for updating the Guidelines of the American Heart Association (AHA) / American Stroke Association (ASA) in recommending that ICH patients should have their BP controlled to a target of <140mmHg (Class IIa; Level of Evidence B). Even so, uncertainty persists over the management of elevated BP in acute ICH, and this impacts on clinical practice, so that any potential benefits of such a treatment strategy are not being passed on to patients. The reasons for this are outlined below.

- ***Different results of INTERACT2 and ATACH-II*** The neutral result of ATACH-II has created confusion amongst some clinicians who may not fully appreciate differences in the BP management protocols and treatment parameters to INTERACT2 that might explain the differing outcomes between the two studies. ATACH-II showed there were more SAE events during 90 days in intensive BP treatment group where the achieved mean minimum SBP was <130 mmHg (129 mmHg at 0-2 hours and 122 mmHg at 2-24 hours). Moreover, the protocol-defined level for cessation of intravenous BP lowering in INTERACT2 was <130 mmHg, whereas it was <110 mmHg in ATACH-II. A sub-analysis of INTERACT2 showed that achieved post-randomisation mean systolic BP of 130-139 mmHg during the initial 24 hours was associated with the best outcome for ICH patients; but also that there was a modest increase in poor outcome for levels <130 mmHg.[12] These results imply that very rapid and intensive BP lowering to treatment targets <130 mmHg in patients with very high BP could refute any potential treatment benefits.

- ***No clear time-relation of BP control on the clinical outcomes*** The most plausible mechanistic explanation for the benefits of early intensive BP lowering are on attenuation of haematoma expansion. Since most ICH expansion occurs within the first few hours of onset, the earlier BP can be controlled, the greater the likelihood of benefit. However, INTERACT2 showed no clear heterogeneity in the treatment effect on the primary outcome between patients randomised before and after 3 hours from ICH onset. Rather, there were only non-significant trends of intensive BP lowering in relation to time, intensity, and 'degree of control' of BP on haematoma growth.
- ***No clear overall treatment effect on haematoma growth*** INTERACT2 showed only a modest non-significant treatment effect on haematoma growth (overall, absolute adjusted between-group difference of 1.4 mL). This raises the potential for other mechanisms, such as on cerebral oedema, haematoma contraction, and 'neuroprotection'. However, the size of this effect on haematoma growth was similar to that seen in the pilot phase INTERACT1 (i.e. providing independent confirmation), and was comparable to that seen with the haemostatic agent recombinant tissue Factor VIIa. Similarly, ATACH-II only showed a trend towards a reduction in haematoma growth at 24 hours. The lack of a clear mechanistic effect of early intensive BP lowering creates uncertainty over the clinical benefit and raises the possibility that the effects of BP lowering treatment take several hours or even days to fully manifest.
- ***No clear superiority of any agent over another*** INTERACT2 was a pragmatic non-regulatory trial designed to evaluate a management strategy rather than a particular agent. While the range of agents included across different health care settings enhances the generalisability of the results, it also raises concerns about treatment interactions and variability across agents, since the ATACH-II used a standard intravenous nicardipine BP lowering regime.
- ***Patients with severe ICH or planned for early surgery were generally excluded from trials*** INTERACT2 and ATACH-II specifically excluded patients deemed to have a poor prognosis or requiring early decompressive surgery. As a consequence, most included patients with mild-moderate size haematoma volumes. Only one recently published study, which specifically examined the effects of intensive peri-operative BP lowering using a similar protocol to that of INTERACT2, showed no differential treatment effects but as it involved only 200 neurosurgical patients was under-powered for hard clinical endpoints. Further research involving these patients is important to guide clinical practice.
- ***A 90-day outcome is too short to assess full recovery from ICH*** It is recognised that stroke patients, and in particular those with ICH, continue to improve over 12 months or more. Moreover, recover for ICH patients has a different trajectory to those with ischaemic stroke. Thus, the benefits of BP lowering therapy, and other medical management strategies, are likely to be fully appreciated over a period longer than the conventional 90-day mark that has been used in stroke trials, mostly for regulatory purposes.

Potential barriers to implementation of early intensive BP lowering into clinical practice

The gap between best evidence and evidence-based practice is well recognised. Failure to translate new knowledge into clinical practice and decision making in health care is a major barrier preventing people from receiving the benefit of advances in biomedical sciences. The factors that impede the efficient transfer of well-substantiated clinical research, such as from INTERACT2, into clinical practice are myriad and complex, covering system issues and barriers in knowledge, attitude and behaviour: scepticism and mistrust of the data, as well as uncertainty about its applicability to practice; ongoing caution in rapidly lowering BP due to ongoing concerns over safety, particularly on cerebral blood flow; behavioural change may hindered by external pressures that favour the inertia of the status quo - includes environmental factors such as the need to commit resources to the process of initiating a new treatment protocol, medico-legal concerns, and institutional and regulatory issues; and logistical difficulties in controlling BP due to complexities in switching from bolus to infusion, and effective the monitoring of patients (system issues). Implementation science involves understanding and overcoming the barriers and leavers to translating research evidence into practice.

Glucose and outcome in ICH

Hyperglycaemia is very common after acute ICH. This occurs irrespective of the presence of underlying diabetes mellitus, and predicts increased risk of haematoma expansion,[13, 14] perihaematomal oedema,[15] death and poor functional outcome.[16, 17] Both the INTERACT2 study cohort and a large multicentre Chinese registry study that included a broad range of ICH patients have shown a near continuous positive relationship between blood glucose levels and poor outcome. As these studies have adjusted for baseline severity of the condition, hyperglycaemic appears to exert a direct adverse consequence on the brain and is not related to acute stress reaction and/or the brain injury inducing autonomic and metabolic changes. The mechanisms underlying poor functional outcomes induced by hyperglycaemia are not yet clear. Animal studies suggest that elevated blood glucose exerts deleterious effect on brain through inducing neuronal apoptosis, increasing superoxide and inflammatory cytokines production, down-regulating the aquaporin-4 (AQP-4) expression and exacerbating perihematomal cell death in the brain.

No clinical trial of glycaemia control in acute ICH In the absence of clinical trials, there is uncertainty as to whether tight glycaemic control after acute ICH improves clinical outcomes. Moreover, there is no solid criteria around which treatment for elevated blood glucose should be initiated and to what target level should be achieved. The American Diabetes Association recommends a target glucose range of 7.8-10.0mmol/l (140-180 mg/dl) for the majority of critically ill patients receiving insulin therapy for persistent hyperglycaemia (Grade A).[18] A meta-analysis has shown no additional benefit from tighter glycaemic control (blood glucose:4.4-6.1mmol/l) in critically ill patients, but a fivefold more greater risk of harmful hypoglycaemia at this level compared to mild (7.8-10.0mmol/l) or very mild control (10.0-12.2mmol/l).[19]

Body temperature and outcome in ICH

Fever (pyrexia) is common in ICH and is associated with poor outcome. Fever is common in all types of brain injury,[20-22] including ICH, especially where there is intraventricular haemorrhage (IVH). The frequency of fever (defined as temperature ≥37.5 ℃) is as high as 90% in first 72 hours, and decreased with time.[23] In those who survive to 72 hours, fever remains a predictor of poor outcome.[24] With each 1 °C increase in body temperature there is a doubling in the risk of poor outcome.[25] Early onset fever has been shown to be an independent predictor of early neurological deterioration, suggesting a relationship with secondary neurologic injury.[26] Multiple factors are likely to contribute to fever in ICH including systemic infection and inflammation around the hematoma.[27] Excitotoxicity

from amino-acid and free radical production, cytoskeletal proteolysis, inhibition of protein kinases, and activation of certain matrix metalloproteinases, could compromise the blood-brain barrier and worsen cerebral oedema.

Trials of temperature control after acute stroke Two promising therapeutic strategies are: (1) treatment of fever to sustain normothermia (<37.5 ℃) or more aggressive and invasive (2) targeted temperature management (TTM) inducing hypothermia (core body temperature ≤36.5 ℃). Guidelines[28, 29] recommend antipyretic treatment for stroke patients based on the consistent association between increasing body temperature and poor neurological outcome. However, there are no precise recommendations on the methods for fever control, pharmacological or mechanistic, due to a lack of randomised evidence. One large clinical trial of prophylactic use of acetaminophen (6g/day oral/rectal or placebo) (the Paracetamol [Acetaminophen] in Stroke [PAIS-1] study)[30] of 1400 patients, of whom 11% had an ICH, showed not effect (adjusted OR 1·20, 95% CI 0·96–1·50) but in the subgroup of patients with fever at baseline (37-39 ℃), paracetamol increased the odds of functional improvement (OR 1.43; 95%CI 1.02-1.97). These data indicate that early control of fever but not prophylaxis, could improve clinical outcomes. The ongoing PAIS-2 study is evaluating the use of high-dose paracetamol in patients with acute stroke whose body temperature is ≥ 36.5 ℃.[31]

In the Australian cluster randomised controlled trail - Quality in Acute Stroke Care (QASC) study[8] - acute stroke units that received treatment protocols related to the management of fever, hyperglycaemia, and swallowing dysfunction showed improved outcomes. In this study, body temperature was monitored and charted every 4 hours for 72 hours after admission and if a patient's temperature reached ≥37.5℃ he/she was treated with paracetamol (intravenous, per rectum, or oral), unless clinically contraindicated. The results showed that, irrespective of stroke severity, intervention group of patients had improved functional outcomes. Although the contribution of fever management and other individual components of the package cannot be shown, the study suggests that the use of protocols addressing aspect of nursing care are beneficial.

Reversal of anticoagulation

Anticoagulation associated ICH has a poor prognosis Vitamin-K antagonists, mainly warfarin, have a 7-10 fold increased risk of ICH,[32] and the resulting haematoma has a larger volume, a greater and more protracted expansion, and results in a higher case-fatality (up to 50%) than for other types of ICH.[33, 34] Thus, rapid replenishment (avoiding delays for coagulation test results[35]) of the vitamin-K dependent coagulation factors (II, VII, IX and X) is considered essential to reverse the anticoagulant effects, but the benefit of this approach has never been demonstrated. The rapid reversal international normalised ratio (INR) to <1.3, achieved within 4 hours, was associated with a low rate of haematoma expansion in a large German retrospective cohort study.[36] Other treatment options include use of fresh frozen plasma (FFP) or prothrombin complex concentrate (PCC). Advantages of PCC include the small infusion volume (lower risk of congestive heart failure) and rapid infusion speed enabling rapid reversal, whereas FFP requires thawing before infusion (as it is kept frozen), thus delaying INR normalisation. Moreover, 30 mg/kg 4-factor PCC has been shown superior over 20 ml/kg FFP in reversing anticoagulation within 3 hours after the start of treatment (OR 30.6, 95% CI 4.7-197.9) with higher odds of significant haematoma expansion in the FFP group (OR 3.8%, 95% CI 1.1-16.0).[37] A large multinational pooled analysis of 16 stroke centres suggests that the combination of FFP and PCC might further improve outcome compared to PCC alone.[38] Besides repletion of coagulation factors, 5-10 mg of vitamin K should also be administered

intravenously to restore production of vitamin-K dependent coagulation factors.[29] INR should be checked at regular intervals because of the long half-life of warfarin.

Early seizures and outcome of ICH

The frequency of early seizures (<7 days of ICH onset) varies considerably (1-14%) across studies, and is even higher if subclinical seizure activity is considered; these rates are greater than for acute ischaemic stroke.[39-41] The majority of early seizures in ICH occur in the first 24 hours; ≤90% occur in the first 3 days.[41-43] Early seizures are thought to arise from acute disruption of brain integrity and biochemical disturbances (e.g. release of excitatory neurotransmitters, direct toxic effects of blood degradation products), and cortical involvement appears to be a key risk factor.[40, 44]

More recent literature in ICH suggests that during a protracted follow-up period, early seizures are associated with late seizures and the development of epilepsy. [39, 45, 46] Early seizures (most of which were nonconvulsive) were not only independently associated with early haematoma expansion [42] but a trend toward worse functional outcomes.[43] However, as the data are limited, it is uncertain whether early seizures are simply a proxy for the extent of brain injury or independently cause additional harm. Additionally, studies have failed to convincingly show that early seizures are associated with mortality in sICH.[40, 47]

In the absence of randomized controlled trials of ICH patients demonstrating a robust benefit from seizure prophylaxis, primary prevention of seizures after ICH by means of prophylactic use of anti-epileptic drugs is a still a matter of debate and is not recommended by current guidelines.[28, 29] Although there is extensive circumstantial and theoretical evidence that seizures are harmful for the acutely injured brain, the use of anti-epilepsy medication has been associated with worse outcome after ICH. No prospective randomized trials have been performed to determine whether treatment can improve neurological recovery and long-term outcomes. It is recommended that clinical seizures should be treated with antiepileptic drug, but there is insufficient evidence about how, when, and for whom, anti-epilepsy drugs should be given. In particular, as to whether the treatment with anti-epilepsy drugs should be initiated immediately after an early single seizure which may have arisen from the acute brain injury which may resolve over days remains unclear.

Advantages of cluster clinical trials for evaluating complex systems of care interventions

While randomised controlled trials of individual patients are the gold standard for obtaining reliable assessments of treatment effects, they are complex, demanding and costly undertakings that often undertaken over a long period of time. These issues are particular pertinent to patients with ICH, as medical interventions are likely to have modest effects requiring evaluations in studies with sample sizes approaching several thousand patients. Another drawback to individual patient clinical trials is selection bias from the inclusion of patients with a better prognosis related to inclusion criteria and well controlled background care and management offered to participants. This often raises concerns over the external validity of the results to the broader clinical population. Cluster controlled trials (CCTs) can overcome these issues by randomising groups of participants to avoid contamination of the intervention under investigation between patients, and allow evaluation of the implementation of multi-faceted system-wide changes in management strategies.[48-50] The most important implication of randomising clusters rather than individuals is that observations from individuals within a cluster may be correlated. A stepped-wedge design is a type of crossover design in which different cluster switch treatments at different time points but only in one direction – from control to intervention.[51, 52] Figure 1 provides an outline of a stepped wedge CCT involving 4 exposure phases across 3 steps. The stepped wedge design offers a solution to logistical and ethical problems, and may provide sample size efficiency gains over a traditional parallel cluster design. Since all clusters eventually

receive the intervention, there may be good buy-in from clinician investigators and avoidance of ethical concerns that a patient group failed to receive the intervention and potential benefits.

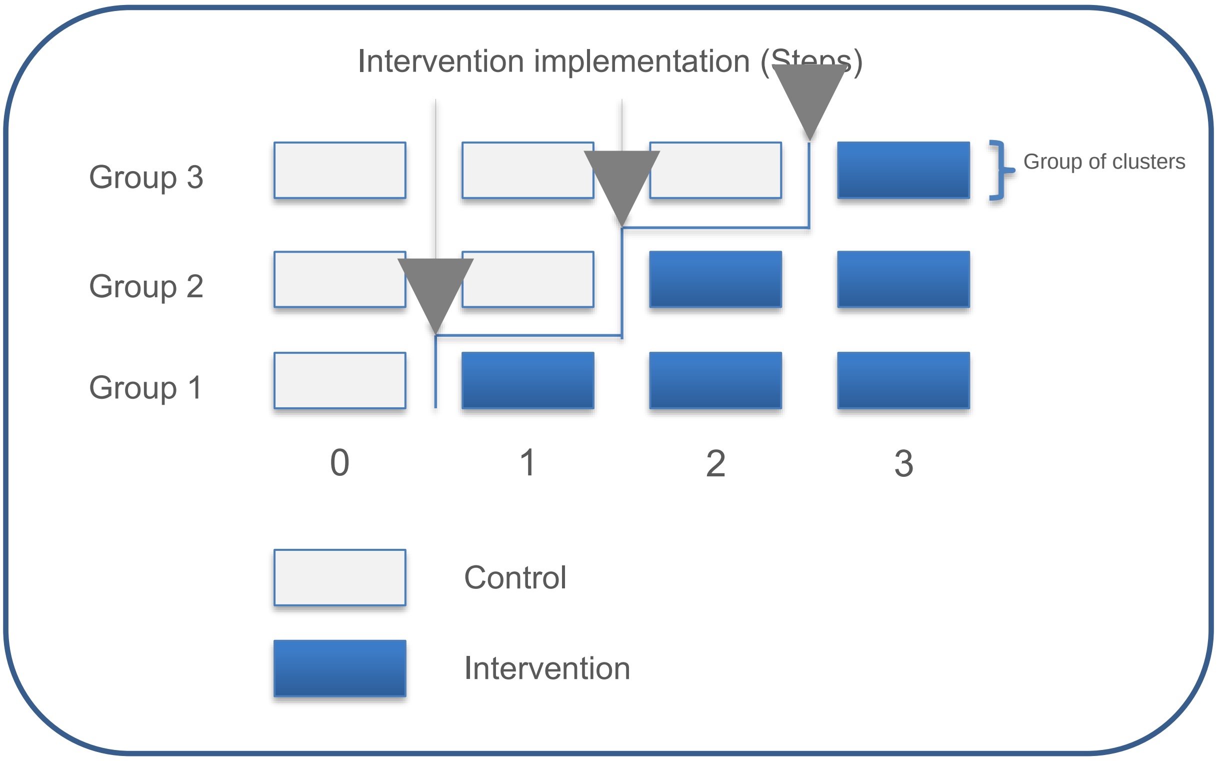

Fig. 1 Stepped wedge design

Summary

Given the complex and serious nature of ICH, reliable evidence is required to guide health care delivery and policy. There is now compelling evidence that stroke can be effectively prevented and managed.[28, 29, 53, 54] However, audits of hospital administrative data for stroke[55, 56] indicate poor adherence to many Level I recommendations. Reliable data on ICH management and outcomes could improve quality of care. We have chosen to evaluate the effectiveness of a goal-directed care bundle of active management involving early physiological control (intensive BP lowering, glycaemic control, and treatment of pyrexia) and reversal of anticoagulation, against usual standard of care, in a prospective, international, multi-site, stepped-wedge, cluster randomised design, with central blind outcome assessment. *The study aims to provide evaluation of the intervention in a broad range of patients, including those undergoing surgery, who have often been excluded from trials of early intensive BP lowering.*

5. AIMS AND HYPOTHESES

Primary aim: To compare with usual care, that a goal-directed care bundle of active management involving early physiological control (intensive BP lowering, glycaemic control, and treatment of pyrexia) and reversal of anticoagulation improves functional outcomes in ICH (i.e. null hypothesis - there is no difference in functional outcomes between treatment groups).

Secondary aims: To determine compared to usual care, the effectiveness of a goal-directed care bundle of active management involving early physiological control (intensive BP lowering, glycaemic

control, and treatment of pyrexia) and reversal of anticoagulation on poor outcome of death and dependency, separately on death and physical function, HRQoL, days of hospitalisation, and living circumstances.

6. METHODS

Overall design

An international, multicentre, prospective, stepped-wedge, cluster randomised, blinded outcome assessment study will be conducted through a global network of investigators. Hospitals will be eligible if they do not have established protocols for the management of ICH or they use different protocols from the bundle under investigation and are comfortable switching to the proposed bundle. The stepped-wedge cluster randomised design has been chosen to avoid contamination, facilitate hospital-wide implementation and maximise adherence, as the intervention issues under investigation would become usual standard of care. The process of one direction (from control to intervention) is to facilitate the goal-directed protocol being applied into clinical practice. The stepped-wedge design means that all hospitals will be randomly allocated to 3 groups. In phase 1, all hospitals will be observed under usual care 'control' conditions according to usual management of ICH patients. In phase 2, the first cluster of hospitals (group 1) will start receiving the intervention (the care bundle), and then sequentially, groups 2 and 3 will start receiving the interventional package in phase 3 and 4, respectively so that by Phase 4, all hospitals will have the intervention, with those in Group 1 having the intervention for longest; and those in Group 3, the shortest. In each phase, hospitals are to manage at target of average 19 consecutive ICH patients, but this could vary from 1 to 50 patients according to expected numbers of ICH across hospitals. The target number for each phase will be pre-determined and agreed to with each hospital. This is also an time limit for each of the phases all sites to ensure an orderly completion of the study. For Group 1, the time limit is 3 months (4 months in China) after initiation; for Groups 2 and 3, the time periods are 6 months and 9 months (8 and 12 months in China), after activation, respectively. For example, a site assigned to Group 1 whose recruitment number is pre-determined as 30 patients is required change over to intensive care-bundle phase after 3 month (4 months in China) even if it has not achieved the target of 30 patients recruited into usual care. Data collection will occur at baseline, the first 24 hours, and at the time of hospital discharge, day 7 (or death), and at 6 months (end of follow-up). Patients will be asked to consent to being contacted at some future date to examine long-term outcomes, according to available resources. Randomised allocation of intervention will be assigned by a statistician not otherwise involved in the study according to a statistical program stratified by the country and size of site.

The study design is summarised in the following schema.

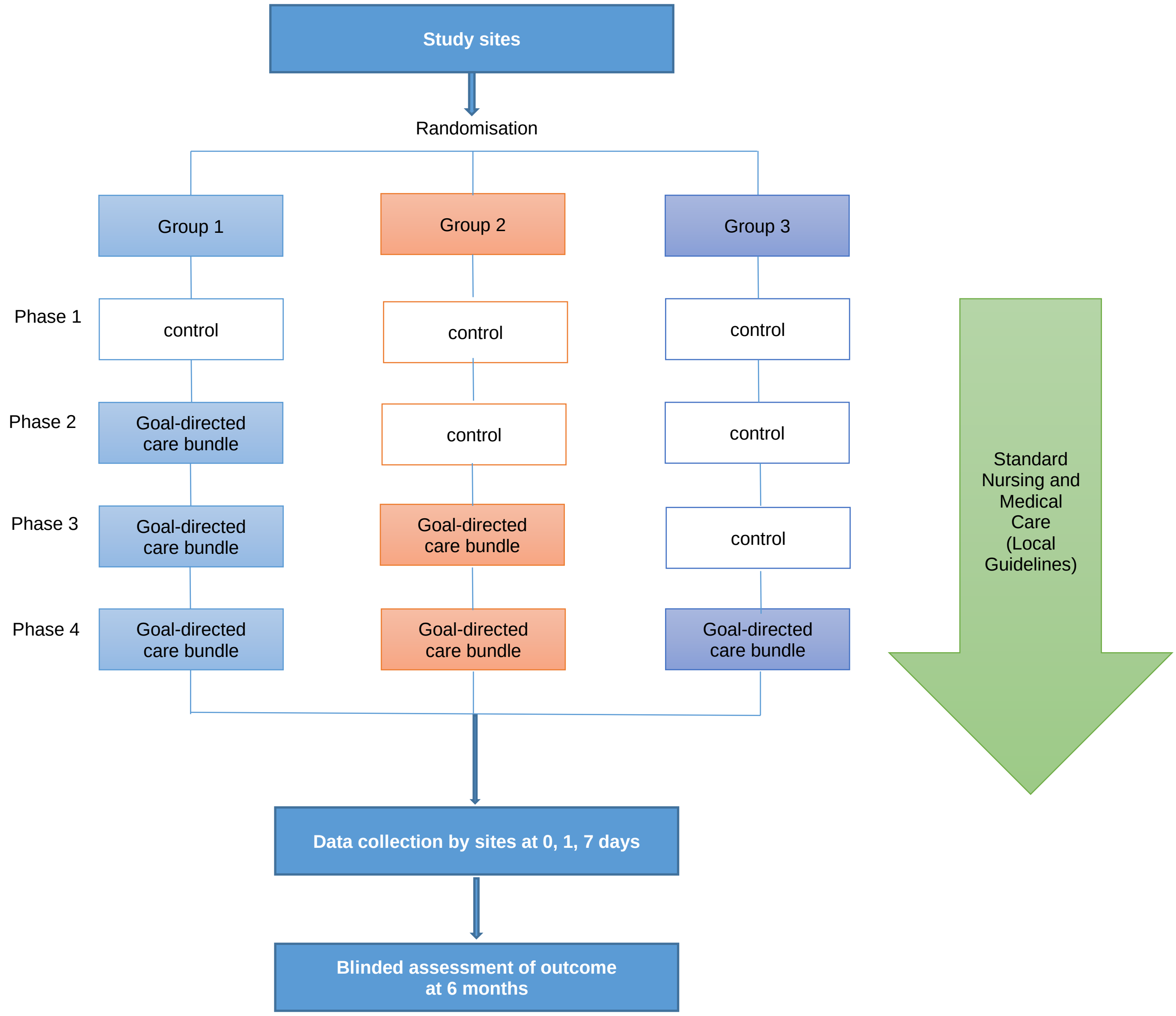

Study population

INTERACT3:

Hospital sites will be eligible if they do not have established formal organisational protocols for the management of ICH or they use different protocols under investigation from the care bundle described in this study and are comfortable switching to the proposed bundle. All patients presenting to participating sites with acute ICH will be included in a register during the study period, and those who fulfil eligibility criteria will be included in the study. Primary responsibility for recruitment of patients will lie with the Principal Investigator (PI) at each centre. It is anticipated that successful recruitment will require the active involvement of staff in the emergency department, since rapid referral of patients early after stroke onset is required. Rate limiting steps after presentation are anticipated to include:

- completion of CT scan;
- assessment of patients who would be eligible to receive goal-directed treatment;

- receipt of consent for data collection (INTERACT3), but this can occur after the patient has been admitted to hospital and they are clinically stable;

Inclusion and exclusion criteria

To be eligible for inclusion in INTERACT3, patients ARE to satisfy all of the following criteria:

- aged ≥18 years
- Acute stroke syndrome that is due to presumed spontaneous ICH, defined as the sudden occurrence of bleeding into the parenchyma of the brain that may extend into the ventricles and, in rare cases, into the subarachnoid space, confirmed by clinical history and a CT scan within 6 hours of stroke onset with/without contrast, and if an CT angiogram is also undertaken as part of routine care. (NB Patients with ICH secondary to medical treatment [e.g. antiplatelet or antithrombotic therapy], are eligible, but ICH secondary to thrombolysis are ineligible).
- Presentation to hospital within 6 hours of stroke onset. (NB If the precise timing of the first symptoms or signs of the qualifying event are unknown then the time of onset will be taken as the last time at which the patient was known to be well).

Patients will NOT be eligible if there is one or more of the following:

- Definite evidence that the ICH is secondary to a structural abnormality in the brain (eg an AVM, intracranial aneurysm, tumour, trauma, or previous cerebral infarction) or previous thrombolysis.
- A high likelihood that the patient will not adhere to the study treatment and follow-up regimen. In each case, the decision about the patient's eligibility will be based on the attending clinician's interpretation of the above eligibility criteria.

All acute ICH patients screened for the study that are not included, as well as recruited patients, must be recorded on the screening/ enrolment log. Sites are requested to provide information on any death known among non-participants registered on the screening/enrolment log up during the 6 months of follow-up. This information is to be uploaded to the ICC database. All eligible patients will be provided with an approved patient information sheet (PIS) or patient responsible information sheet (PRIS) and consent form (CF) on admission for permission to collect their medical and personal information and to be contacted at 6 months follow-up. A participant is anyone for whom consent is obtained for data collection and follow-up as early as possible after his/her admission to hospital.[57]

Hospital sites will continue to enrol ICH patients under standard of usual care management until they are informed of crossing over to implementation of the care bundle based on reaching a required target number of patients and readiness to change.

Ethical issues

Institutional ethics committee approval and consent

This study will be conducted in compliance with the principles outlined in the World Medical Association's Declaration of Helsinki (see Appendix 1). Each participating site must obtain written approval(s) from their Hospital Research Ethics Committee (EC) (e.g. Institutional Review Board [IRB]), and other relevant regional or national bodies, if applicable, before patient recruitment can commence. It is the responsibility of the PI of each participating site to report any protocol amendments, SAE reports, and routine reporting according to the requirements specified by policies and the written approval of the EC.

A mixed consent process is proposed, according to local/national rules and regulations, for the following protocol:

- Cluster Guardian consent or appropriate approval (e.g. signed by General Manager or Chief Executive of hospital, or Head of Neurology/Neurosurgery/Stroke Department) for patients to receive the randomised care bundle to be implemented for acute ICH patients in Emergency Department, Stroke Unit, Intensive Care Unit or Neurology/Neurosurgery Wards.
- With one of the following:
  - Individual standard consent for the collection of data through in-person assessment and data extraction from medical records during the hospital stay and follow-up, and for release of personalised information for research purposes to allow centralised follow-up at 6 months following admission, or
  - Opt-out consent for collection of data through in-person assessment and data extraction from medical records during the hospital stay and follow-up, and for release of personalised information for research purposes to allow centralised follow-up at 6 months after admission.

Need to recruit consecutive patients: As is usual in trials for practice change implemented at the cluster level, all consecutive patients admitted with acute ICH meeting the study criteria will be treated to the random assigned care bundle as usual medical treatment. This is to ensure there is minimal bias due to patient selection in the study.

Reasons for use of cluster guardian consent: Consent under the cluster guardian format or appropriate approval is necessary to avoid confusing control standards for busy clinicians caring for multiple patients; to allow implementation of interventions as soon as possible as part of routine care; and reduce responder bias in patients (or surrogates) as a result of thinking that they have received 'non-standard'. The use of cluster consent is further supported by multiple guideline recommendations towards use of the following strategies with minimal harm and potential benefits:

- Intensive BP lowing to systolic target of <140mmHg within 1 hour, monitoring of blood glucose and avoidance of hyperglycaemia, treatment of fever, and early reversal of anticoagulation for patients with acute ICH admission, but where there is still uncertainty about effectiveness and patient subgroups who have the most to gain from such treatment (hence levels of recommendations are level B or C, rather than A);
- Various forms of care (bundle) of active management are within the routine workload of medical and nursing staff;
- Insulin infusion may be required for patients whose glucose levels ≥7.8 mmol/L without previous diabetes or ≥10 mmol/L with a diabetes history, again without the spectrum of routine care with substantial randomised evidence to indicate these targets are safe.
- No other aspects of patient care will be changed.

All INTERACT3 participating patients (or legal surrogates) will be given an information sheet that 'the hospital is participating in research that is assessing medical treatment to standardise ICH care around the world' and the need for data collection (in-hospital) and follow-up assessment. An information statement will be given to the patient and the implications for consenting to the study will be explained by a clinician familiar with the study protocol.

The standard withdrawal of consent or the opt-out consent process will be used should a participant decline to participate at any time during the study either for the collection of medical information or the 6 months follow-ups. The opt-out consent is a more robust method to ensure that data on the

primary endpoint is as complete as possible. The consent process used for sites will be according to the local approval received.

Cluster guardian responsibilities *include*: (i) act as the advocate in the best interest of the patient cluster in mind; (ii) approve the randomised intervention becoming the new usual medical treatments at the relevant departments in the hospital during the study period; and (iii) complete a cluster guardian CF indicating approval of the hospital to adhere to the intervention during the study period.

Patient consent process: All patients to be given a PIS and CF as soon as possible following their admission, either on admission or ideally within the first 72 hours. The PIS will outline that the hospital (site) is "participating in research that is assessing nursing practice to standardise stroke care around the world" and that there is a need for data collection (in-hospital) and follow-up assessment at 6 months. The patient will have the opportunity to discuss and seek explanation from a clinician familiar with the study protocol and notify of the local approved consent process for the collection of their data (in-hospital and/or follow-up assessments). This process will be fully documented, dated and signed in the patient's file.

For standard consent, ideally the patient will need to be consented within 72 hours of admission.

For consent withdrawal, the patient can withdraw at any time during the study period, by returning the signed withdrawal form to the site or RCC. The contact details (email, fax and postal address) for returning the return withdrawal form will be included in the PIS/PRIS and CF.

Person responsible consent: The majority of patients with acute ICH will require emergency care, and the nature of the condition means that many patients are unwell and unable to comprehend the information that must be given to them. If the patient is not fully competent, for example because of a reduced level of consciousness, cognition or confusion, the patient's "person responsible" will be approached and will be provided with the person responsible information sheet (PRIS) to read and act on the patient's behalf. Under the Guardianship Act of 1987 in Australia, a "person responsible" is the legally appointed guardian, their spouse or de-facto spouse or same sex partner; or if there is none, their unpaid carer; or if there is none, their relative or friend who has a close relationship with the person.

In situations where the patient is unable to provide consent or opt-out consent, the person responsible consent or opt-out can be obtained, and the patient will be made aware of this as soon as they are well enough before 7 days or at discharge from hospital, so that they have an opportunity to discuss and seek explanation from a clinician familiar on the study protocol. They will be given the opportunity to re-consent or opt-out of the collection of their data (in-hospital and/or follow-up assessments) during their stay in hospital or as soon as they are able to decide, according to the local approved consent process.

If the patient is dying or is still unable to record their personal consent by the completion of the follow-up assessments, the consent or consent withdrawal given by their person responsible will stand and study data will be retained or removed. The reason for not being able to obtain the patient's consent will be documented, dated and signed in the patient's file.

Withdraw consent: All patients and/or person responsible at participating sites will be given the information sheet as soon as possible following admission. Withdraw consent will be clarified as a component of the PIS and PRIS, as outlined below.

- All patients and/or their person responsible will be made aware that they retain the right to withdraw the database collection,

- Details will be given about where they can obtain further information, including contact details of the RCC.
- If the patient and/or their person responsible decide at any time during the inpatient admission (or any time until the end of follow-up) that they wish for any personal details about themselves or their in-hospital care record, or during follow-up, not be included in the study, they may request removal of their data from the database. The requested can be assigned by completing and returning the consent withdrawal form provided. Acknowledgment of receipt and removal of their data from the database will be sent to the patient and/or their person responsible, by mail or email.
- The PI will ensure that all staff at the site is made aware of the consent withdrawal in protocol.

Definition of participant and assessment of selection bias

A participant is any subject for whom consent for data collection has been obtained. The care bundle intervention is expected to commence as soon as possible after a subject (participant) is considered to have fulfilled the inclusion criteria, including with a diagnosis of acute ICH being made by brain imaging in the ED within 6 hours from stroke onset. Thus, ICH patients become participants as early as possible after presentation in the ED and before admission to the stroke unit or other designated ward for the management of these patients. However, as selection bias may compromise the integrity of the study, assessments will be made of the frequency and profile of non-participants and of early participants who later opt-out of data collection.

- In this regard, ethics committees are requested to approve the collection of several key case-mix variables (age, sex, time from onset, pathology and severity) on non-participants and those participants who opt-out of in-hospital data collection and/or follow-up.

Confidentiality and privacy

Every precaution should be taken to respect the privacy of patients in the conduct of the study. Only de-identified data will be used for statistical analyses and publication of results to maintain patient confidentiality. However, as a part of centralised follow-up service, the ICC at The George Institute for Global Health (GI) and RCCs will use contact sources recorded by the sites to do the outcome assessments. Only name, phone numbers, next of kin and GP contact (if applicable) will be sent to the follow-up centre to do the follow-up assessment. The information will be encrypted and password protected using the MS Excel lock form before sending by email in batches. This information will be included in the PIS and PRIS. In the course of monitoring data quality and adherence to the study protocol, the monitor will refer to medical records at the participating hospital. This information will be included in the PIS and PRIS. All individual and site information will be de-identified in reporting data and results to protect the confidentiality of participants.

Study site selection, participation and responsibilities of staff

Study network: The study will be conducted in approximately 110 hospitals (sites) in China, Chile, Peru, Mexico, Brazil, Vietnam, Pakistan, Nigeria and Iraq. Hospitals in other countries may be invited to join according to interest, feasibility and resourcing. A similar study is being planned in the United Kingdom and arrangements are being made for pooled meta-analysis of data from both trials.

Site eligibility criteria: To participate, sites are required to fulfil certain eligibility criteria:

- Have an established acute stroke care program with a geographically-defined area for the management of ICH patients (i.e. in an acute stroke unit, neurosurgical unit, neurology unit), defined by (i) having staff organised as part of a coordinated multidisciplinary team; (ii) having

staff with some knowledge and skills in the stroke management; (iii) have staff interested and capable of participating in research; and (iv) not currently having written protocols for the management of common problems in ICH.

- Have a sufficient number of patients with acute ICH to ensure feasibility of recruitment within a reasonable time frame (i.e. admit approximately 100 ICH patients per annum).
- Obtain necessary ethics committee and other relevant approvals from hospital management;
- Agree to enter data via the internet to a secure server based in China;
- Nominate key research staff assigned to the project to obtain consent from participants;
- undertake the necessary pre-study training programs;
- comply with the requirements of good clinical practice (GCP);
- agree to the following conditions:
  - adhere to the protocol;
  - collect data on patients' stay in hospital;
  - record any SAEs noted during the 6 months of follow-up of patients;
  - Sign a contract agreement before the commencement of recruitment.

Responsibilities of the site PI are:

- have sufficient staff resources to participate in the study,
- ensure all sub-investigators and nursing staff and coordinators undertake the necessary pre-study training programs regarding the protocol and positioning of the patients,
- agree to comply with GCP requirements,
- obtain agreements from all relevant departments to comply with the intervention for all ICH patients during the study period,
- submit and obtain EC approval and ensure compliance to EC requirements,
- work with the RCC to obtain Cluster Guardian consent for conduct of the study.

Responsibilities of the nominated Local Champion include the following:

- receive training on the protocol, and to 'champion' the study at the hospital,
- provide refresher training for existing and new staff on the intervention on a regular basis,
- conduct spot checks adherence to the randomised intervention,
- conduct a Process Evaluation questionnaire, as per the monitoring plan, to ensure the staff are able to comply with the protocol (intervention fidelity),
- assist in problem-solving and propose solutions to resolve any local issue that may pose a barrier to the adherence to the intervention,
- oversee the data collection,
- conduct spot checks of the patient screening and enrolment log (Form A)
- liaise with the RCC on issues of adherence and quality.

In the selection of suitable sites, there is an expression of interest (EOI) questionnaire, available on line to collect preliminary information about the site, so the RCC can ascertain the suitability of the site to participate in the study.

Hospital organisational questionnaire: To assist the implementation of the intervention in each site, it is important to know the organisational structure of the site and the various areas within the site that will be involved in the intervention. Therefore, each site will have to complete a Hospital Organisation Questionnaire (HOQ) to assist the RCC prepare the training and site initiation visit.

Site initiation process: Each site will be sent a Hospital Organisation Questionnaire (HOQ) to be completed and returned to the RCC. Arrangements for a site initiation visit (SIV) will be made with the site PI following receipt of the completed HOQ. The designated monitor from the RCC will review the HOQ and arrange the visit and training of staff. The visit to the site is the planning stage before commencement of the study to initially present the protocol to all the staff and investigators who will be involved in the study, tour the various areas such as the ED, ICU and stroke ward(s), collect all essential documents, and review any barriers and issues to compliance of the protocol and future care bundle intervention. Before a site can be initiated to commence the study, they need to have submitted/completed all essential documents, obtained ethics approvals, signed agreements and received initial training. Once the site has completed the necessary control usual care observational period, they will receive training on the application of the care bundle intervention and subsequently will receive performance monitoring and feedback on their adherence to the intervention during the course of the study. This will be provided by the RCC. A transition period of 7-10 days will be used between completion of the control period and commencement of the intervention.

Randomisation of sites

The unit of randomisation of is the hospital site; randomly assigned by a statistician not otherwise involved in the study to 3 groups using a pre-specified randomisation schedule with permuted blocks. Participating sites will be stratified according to country and size of site.

When the site is ready, they will be notified of the randomised group within 2 weeks of an agreed date of commencing the study.

All eligible stroke patients presenting to the hospital from the start date are to be prospectively and consecutively enrolled.

Intervention

All hospitals are required to adhere to the protocol for all randomised patients. It is anticipated that there will be broad comparability of the regimens used in participating centres within each county. As the trial is an assessment of care bundle of physiological management, there is some flexibility in the use of particular BP lowering agents and antipyretic agents to achieve targets. All patients received continuous noninvasive cardiac monitoring, including ECG, oxygen saturation, heart rate and arterial blood pressure. Routine laboratory tests will be performed on admission and at least once during the following 7 days; in the case of significant abnormalities, they were taken daily.

Goal-directed care bundle of active management group including early intensive BP lowing, intensive glucose control, early treatment of pyrexia and reversal of anticoagulation.

- ***Early intensive BP lowering*** The aim is to achieve a systolic BP level <140mmHg within 1 hour of treatment and to maintain this BP level for the next 7 days or hospital discharge should this occur earlier. Intravenous BP lowering is to commence as soon as possible upon admission, and thus commenced, in most cases, in the ED where patients can stay and receive intervention and

monitoring until clinically stable. Intravenous bolus (or maintenance infusion) treatment would then be continued in an acute stroke unit or other monitored facility, although a high intensity nursing care and monitoring facility (ie an intensive care unit) is likely to be required for use of an intravenous infusion of a BP lowering agent in most sites. It is expected that intravenous therapy will continue to be required during the initiation of oral antihypertensive therapy, in order to maintain the systolic BP levels of less than 140 mmHg. However, a systolic BP of 130 mmHg is considered to be the lower limit for the cessation of intravenous BP lowering therapy.

- Intravenous treatment protocols, based on Urapidil in China and available medications in other countries, are provided in Appendices 2A to 2D. The intravenous treatment will be titrated against regular BP monitoring to achieve a target systolic BP (<140 mmHg). It is anticipated that intravenous control of systolic BP will be continued for a minimum of 24 hours and possibly up to 48 hours.
- Oral treatment will commence as soon as feasible. The switch from intravenous to oral BP lowering treatment will be made at the discretion of the responsible physician, depending upon the control and stability of the BP and the clinical status of the patient. It is anticipated that oral treatment will be started by 24 hours. An oral treatment protocol is provided in Appendices 2A to 2D. Combination treatment with an ACE inhibitor (or ARB) and diuretic will be recommended on top of other therapy as the first line oral treatment on the basis of the results of the PROGRESS trial and established best practice for the long-term prevention of BP-related events in patients with cerebrovascular disease. The oral treatment protocol will also include a defined strategy for titration of treatment to achieve effective early systolic BP control once oral treatment is commenced. If the patient is unable to swallow, treatment should be administered via nasogastric tube.

For the intervention group, the goal is to maintain systolic BP levels of <140 mmHg for 7 days of hospital stay. If the patient is transferred to another hospital facility within 7 days, then attempts should be made to continue therapy to achieve the systolic BP target of <140 mmHg. The target systolic BP after hospital discharge remains <140 mmHg, as per guideline-based recommendations for high risk vascular disease patients.

- ***Intensive glucose control*** The aim is to achieve a target blood glucose level of 6.1-7.8 mmol/l for non-diabetic patients and 7.8-10.0mmol/l for diabetic patients and to maintain this BG level for the next 7 days or hospital discharge should this occur earlier.

  At the time of admission, blood glucose level and HbA1C will be tested in all patients. Patients with increased HbA1C will be diagnosed as diabetics, undiagnosed diabetics, or pre-diabetics.

  In the intensive-treatment group, a continuous infusion of insulin (50IU in 50ml of 0.9% sodium chloride with the use of a pump) should be started as soon as possible if the blood glucose level exceeded 7.8mmol/l in non-diabetics and 10mmol/l in diabetics. The infusion adjusted to maintain the level at a value between 6.1 and 7.8mmol/l for non-diabetics, and 7.8 and 10.0mmol/l for diabetics.

  In the conventional-treatment group, an insulin infusion started only if the blood glucose level exceeded 12mmol/l, and the infusion was adjusted to maintain the level at a value between 10 and 12mmol/l.

  Adjustments of the insulin dose were based on measurements of whole-blood glucose, performed at one- to four-hour intervals with the use of a glucose analyser. The dose was adjusted according to a strict algorithm by a team of intensive care nurses, assisted by a physician.

On admission, all patients were fed continuously with intravenous glucose. The next day, total parenteral, combined parenteral and enteral, or total enteral feeding was instituted according to a standardized schedule, with 20-30 non-protein kilocalories per kilogram of body weight per 24 hours and a balanced composition (including 0.13 to 0.26 g of nitrogen per kilogram per 24 hours and 20 to 40 percent of nonprotein calories in the form of lipids). Total enteral feeding was attempted as early as possible.

- ***Treatment of pyrexia*** The aim is to achieve a core body temperature level <37.5℃ within 1 hour of treatment and to maintain this temperature level for the next 7 days or hospital discharge should this occur earlier. Measurement of temperature is according to standard practice.

  Patients allocated to intensive group will receive measurement of body temperature every 4 hours with 72 hours after admission. Patients with an increase in body temperature to ≥37.5℃ will commence fever treatment immediately. A recommended 4-step treatment is shown in Figure 3. At levels 1 and 2, patients received a single drug of 500 mg of paracetamol and metamizole (oral or rectal), respectively. In cases of nonresponse to antipyretic drugs, external cooling with calf packing was applied for 60 min at level 3, and finally an intravenous infusion of 500 ml of cooled (4 ℃) saline (0.9% NaCl) over 30 min was performed at level 4. The last step was reserved for patients without clinical signs of congestive heart failure or a history of dyspnea (NYHA III or IV). Measurement of body temperature was performed 60 min after the patient entered each level. Body temperature ≥37.5 ℃ after completion of the last step resulted in a drop back to level 1. The procedure limited treatment to a maximum of 4 complete cycles within 24 hours.

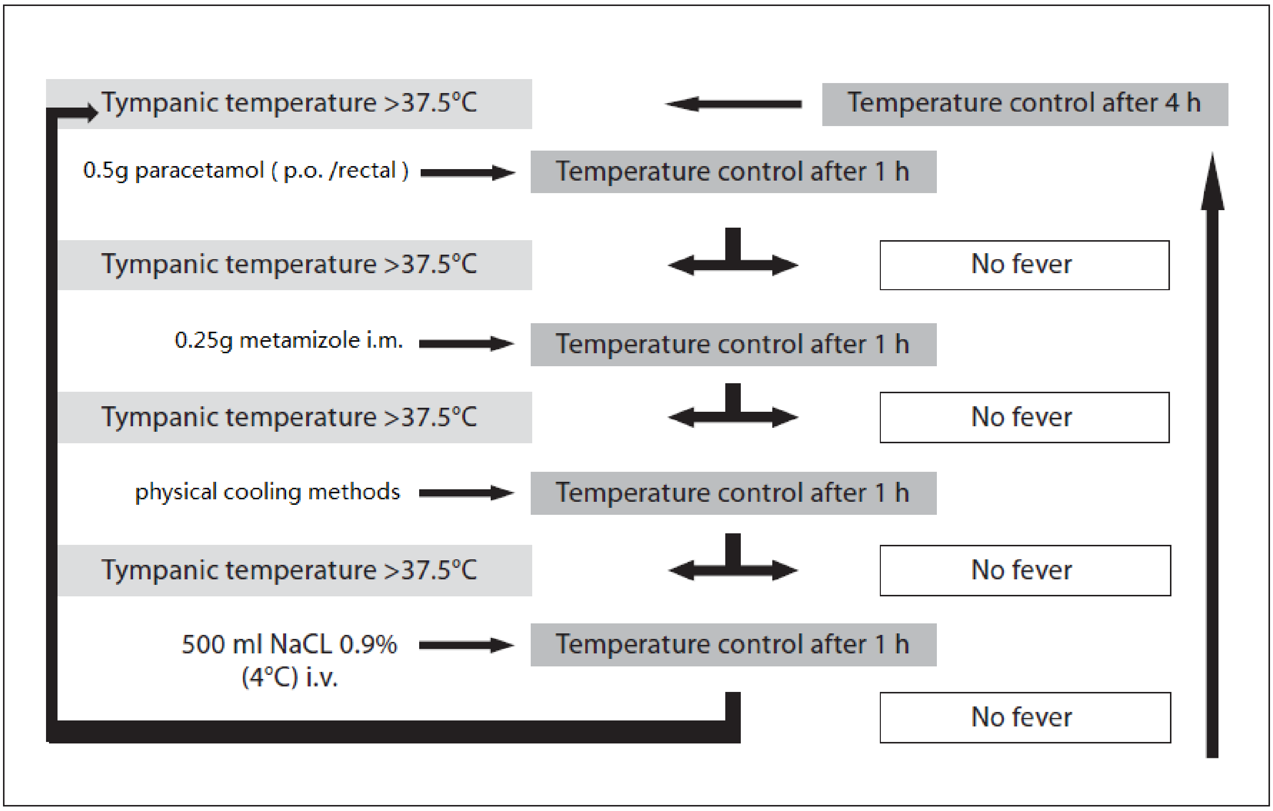

Fig.3 The SOP of antipyretic treatment after ICH. Fever will be treated by following a sequence of 4 interventions with intensive control of body temperature.

- ***Reversal of anticoagulation:*** The aim is to achieve INR<1.5 within 1 hour of treatment and to maintain this level for the next 7 days or hospital discharge should this occur earlier..

  All patients with suspicious ICH should be checked blood INR immediately. Those allocated to intensive group with elevated INR (>1.5) should be given 20 mL/kg of intravenous FFP (after blood group typing or by using AB group plasma supplied by local transfusion units) or 30 IU/kg of intravenous four-factor PCC according to hospital applicability within 1 hour after diagnosis; 5-10 mg of vitamin K will also be administered intravenously and slowly. The speed of the infusion of FFP or PCC should be as fast as the condition of the patient would allow. Patients with an INR greater than 1·5 at 3 h after the start of treatment received PCC (if INR≤2.0, 10 IU/kg; if INR>2.0, 30IU/kg) as a rescue treatment. INR should be checked at regular intervals.

Study outcomes

Primary Outcome: The primary outcome will be the mRS at 6 months analysed as an ordinal outcome (categories 0 to 6).[58]

Secondary Outcomes: Death or dependency measured by NIHSS[59] at 7 days; The following at 6-months: poor outcome defined by mRS scores of 3-6; separately on death and disability (mRS 3-5); health-related quality of life (HRQoL) using the EuroQoL Group 5-Dimension self-report questionnaire (EQ-5D); duration of hospitalisation; and residence.

Safety: Serious adverse events during follow-up.

Data collection and follow-up

Sites are required to collect data on patients at: admission (baseline); 72 hours monitoring chart; separation (Day 1; Day 7 or at discharge if earlier, transfer from the hospital or death) and all SAEs including death until the 6 months of follow-up. Sites will also be requested to provide data on any known deaths among non-participants from the screening/enrolment log prior to separation from hospital and up to the 6 months follow-up. The 6 months assessments will be conducted by an appropriately trained independent outcome assessor using an assigned telephone script, who will be kept blinded to how patients were treated. The assessor will be managed by the regional centre in each country, at the centre or within the sites.

All patients will be analysed according to the “intention to treat” principle. Data collection will be kept to a minimum to ensure rapid enrolment and follow-up of patients in the context of routine practice.

Study management will be facilitated by an established internet-based system. Table 1 illustrates the schedule and nature of the data collection required during the study period. The paper version of the case reports forms (CRFs) will be supplied together with a procedure manual, as a reference and a database completion guideline of each data element with definitions for the hospitals to ensure accuracy and consistency of data collection.

All data entry will be completed using an internet-based data management system. It will be password protected with individual log-ins, with sites and role assignment at regional and site levels to ensure security, privacy and confidentiality between sites and country. The internet-based data management system will allow for real time data entry, query generation for values entered outside the valid ranges and consistency checking.

Screening and Enrolment log (Form A)

Each participating site will register all patients who present to ED, during the intervention period, with a clinical diagnosis of ICH into the *Screening and Enrolment* log (Form A). The log will record patient's gender, initials, date and time of hospital arrival, date and time of stroke onset, date and time of CT scan, patient information form given and consent obtained and patient's ineligibility. This form is also used as the screening log to record the patients who are ineligible for the study together with the main reason as to why a patient was excluded. Such case mix and vital status data is vital to assess potential selection bias through non-participants.

There will be a removable section at the bottom of the log for hospital use only to match the eligible patient for data collection and follow-up. The paper-based log will be a source document to be locked in the filling cabinet with the patient files, for monitoring of the data entered into the database. The log can be used by the Research Coordinator (or designated RCC staff), PI and the ICC to monitor and identify specific barriers to recruitment of eligible and ineligible patients.

Patient Contact Details log

Each centre will keep a record of the contact details and information of next-of-kin for all patients recruited into the study before hospital discharge. This will be kept with the patient file in the participating centre in a locked filing cabinet in accordance with local privacy and confidential policies. This will be kept at the site and used by the local Research Coordinator and PI to make contact with the patient or person responsible in any event or issues arising from the study

Baseline (Form B)

For eligible patients, their baseline information will be collected such as:

- BP, HR and Body Temperature at arrival
- Ethnicity
- Medical history
- Medications at time of admission
- Pre-morbid estimation of mRS at admission
- Basic laboratory test results (especially Blood Glucose, HbA1c, INR)
- Neurological severity according to the GCS and NIHSS
- Brain imaging features (hematoma volume and location)

Day 1 (Form C)

On day 1, the following information is to be entered into the database, using the patient medical records and other source documents

- Highest and lowest BP, Glucose, Body Core Temperature during 24 hours after admission
- Volume of haematoma at 24 hours
- INR at 3 and 24 hours
- Record of reversal medicine of anticoagulation

BP and 72 hours BG / T Monitoring Chart (Form D and E)

As usual nursing care during the study period, the main assessments in the first 72 hours (BP in 7 days) will be used to check adherence to the allocated care bundle parameters. Recording of the times of initiation, duration and types of treatments, together with the reason(s) for any deviations from protocol, are to be recorded and is crucial for monitoring performance. BP and BG and administered medicine and dose will be recorded every 1- 4 hours according to whether it reaches

the goal and is stationary. BP and BG data with definite measure time can be collected and recorded from admission either to ED or to the ward/unit where requested measurement frequency can be done. The start timing of data collection for this form at each site should be consistent through control to intervention phase and should be predetermined and recorded in RCC file.

Day 7 or discharge if earlier (Form F)

On day 7 or at discharge, transfer to another hospital or death, the following information is to be entered into the database, using the patient medical records and other source documents

- NIHSS[59] score
- Dependency assessed with the mRS[58]
- Medications and key specific treatment received during hospitalisation
- Other aspects of acute stroke management during hospitalisation
- Date of discharge or transfer from hospital
- Final diagnosis

6 months Follow-up Blinded Assessment (Forms G)

Standardisation of outcome assessment: These follow-up assessments will be conducted by the RCC staff, or blinded assessor at each site, who will be trained to collect the outcome data systematically. The trained staff will be independent and blinded to the stroke care which the patient received and other clinical information.

All surviving patients will be contacted by an appropriately trained outcome assessor using an assigned telephone script, who will be kept blinded to where patients were treated. The assessor will be managed by the RCC in each country.

The information to be collected at follow-up assessments are:

- Dependency assessed with the mRS[58]
- HRQoL measured by the EQ5D[60]
- Patient living status (e.g. home, high care)
- Any complications or hospitalisation since going home
- Any medication currently used

Death

Patients who die prior to any of the above scheduled assessments, cause of death documentation copies of post-mortem reports, hospital record entry or death certificate if possible, should be kept with the Patient file at the site to assist in study monitoring by the ICC. An SAE form is to be completed for all deaths during the study period, stating the primary and underlying cause of death.

**Evaluation**	**Screen + Enrolment Log**	**Baseline**	**Day 1**	**72 Hour Monitoring Chart**	**7 days / Discharge / Transfer / Death**	**6 month follow-up**
**Forms**	**A**	**B**	**C**	**D/E**	**F**	**G**
Screen	X					
Eligibility	X					
Consent/re-consent	X					

Contact details for follow-up	X					
Vital signs	X	X	X		X	
Physical parameters monitoring				X		
GCS		X				
NIHSS		X			X	
Medical history		X				
mRS					X	X
EQ-5D						X
Routine blood tests		X			X	
Brain imaging		X	X		X	
Standard stroke care		X			X	
Final diagnosis					X	
Medications in use			X	X	X	X
SAEs			X		X	X

7. SAFETY

Data and Safety Monitoring Board (DSMB)

The DSMB will review the safety, ethics and outcomes of the study.

Responsibilities: Monitor blinded response variables and safety outcomes for early dramatic benefits or potential harmful effects using the approach developed by Sir Richard Peto for safety monitoring and provide reports to the SC on recommendations to continue or temporarily halt recruitment to the study. The DSMB will be governed by a charter that will outline their responsibilities, procedures and confidentiality. They will review unblinded data from the study at regular intervals during follow-up and monitor BP differences between the two groups, drop-out, and event rates. The first meeting will be held within 3-6 months after the start of the study recruitment. One or two formal interim analyses will be planned to review data relating to treatment efficacy, participant safety and quality of trial conduct. Prior to the first interim analysis a detailed interim Statistics Analysis Plan (SAP) will be developed to:

- Describe the methodology to be used in the statistical analyses.
- Specify rules if the study is to be halted.
- Specify rules on data handling conventions used to perform the analyses.
- Describe the procedure to be used to account for missing data.
- Outline interim analysis.

Serious Adverse Events (SAEs)

The mechanisms for reporting and notifying SAE are based on the guidelines adopted by the ICH-GCP. As defined by the WHO International Drug Monitoring Centre (1994), an SAE is any untoward medical occurrence that:

- results in death.
- is life threatening in the opinion of the PI (i.e. the patient was at risk of death at the time of the event; it does not refer to an event that might hypothetically have caused death had it been more severe).
- requires inpatient hospitalisation or prolongation of existing hospitalization.
- results in persistent or significant disability or incapacity.
- results in congenital anomaly or birth defect (Note that the females in the study population are likely to be post-menopausal).
- is an important medical event in the opinion of the PI that is not immediately life-threatening and does not result in death or hospitalisation but which may jeopardise the patient or may require intervention to prevent one of the other outcomes listed above.

Recording and Reporting

As ICH is a disease with a high rate of death, disability, surgical intervention, and various associated complications, it would be an enormous job for investigators to report all SAEs, most of which are part of the natural history and management of the condition, according to standard ICH-GCP reporting requirements. Moreover, INTERACT3 is different from prior trials of ICH by including many cases with severe forms of disease receiving neurosurgery.

For the INTERACT3 study, all of the management of patients is within the range of routine clinical practice and guideline recommendations, and the risk of serious reactions related to the intervention package under investigation is small. Therefore, most SAEs will be caused by the disease itself and its complications rather than the interventions that are already being undertaken to varying degrees in routine clinical practice all over the world.

An SAE form (Form X) will be provided for the recording of the event to the ICC. The details of the SAE will include the event diagnosis, classification of the event using standard definitions, the PI's opinion on the causal relationship to the care bundle, and the timing of the event. The PI will be responsible for ensuring that details of investigations and outcomes of an SAE are finalised. The SAE should be documented in the medical records or patient file, and signed and dated by the investigator, for audit and monitoring. All SAE will be reviewed by a medical monitor assigned to the trial. Safety outcomes will be reported to the presiding ethics committees in line with their requirements every 6 months, as well as for review by the DMSB at each meeting.

8. QUALITY ASSURANCE

The study will be conducted at research-experienced sites, in accordance with the ICH Guidelines for Good Clinical Practice and all relevant local, national and international guidelines and regulations. RCCs will be set-up in the various countries to facilitate the compliance and translation of the protocol to local regulations. The RCC will receive training and assistance to set-up documentation required for the study in accordance to ICH-GCP from the ICC. There will be regularly support meetings/teleconference between the RCC and ICC staff. The ICC will provide Standard Operating

Procedures (SOP) to the RCC and sites to assist the compliance to ICH-GCP and the protocol. Manuals and Guidelines will be developed by the ICC in liaison with the Operations committee.

Monitoring of sites

Regional based clinical research monitors will perform online and on site data verification and monitor the conduct of the study. During the study period, the monitor will make a minimum of 3 visits to the sites; (i) the first visit is to check that there are adequate facilities and resources to conduct the study and implementation of the intervention and training of research and nursing staff, (ii) refresher site training and check adherence to the protocol (iii) at close out on completion of the last patient including the 12-months assessment. More visits may be arranged if required depending if there are site issues.

The nominated "local champion" will assist to assess compliance to the care bundle at their site by providing on-going training, solutions to local barriers and ad hoc checking of the protocol and data recordings.

In addition, the regional based clinical research monitors will perform regular online monitoring and liaise with the site coordinator to check and correct any discrepancies or incorrect data entered.

Monitoring the sites serves to confirm adherence to the protocol and Good Clinical Practice (GCP) Guidelines, relevant local and regional ethical requirements, data accuracy and quality.

This will be achieved via monitoring of 10–30% random selection of patients during the study period, and at close out. Monitors will conduct remote checking of information entered into the database on a regular basis and visits to site minimum twice, during the study to confirm against source document:

- the existence of all patients;
- compliance with the procedure of allocated care bundle;
- any unreported SAEs;
- any unreported protocol violations or deviation
- correct/collect outstanding/missing data; and
- selected variables
- patient or person responsible information sheet has been given and process documented in the medical records.

In addition, the study may also be audited by external government or regulatory authorities. Access to CRFs, source documents and other files, must be made available at all participating sites for monitoring and audit purposes at mutually arranged times during the course of the study and after its completion.

Collection and Storage of Essential Documents

At completion of the study, the site will have to ensure that there are plans in place for the long-term storage of all the relevant data and source documentation (for 15 years).

The RCC will work with the site to ensure this is in place during the study. All RCC documentation will also need to be stored until transfer to the ICC for archiving.

Prior to the initiation of the study at any participating site, the PI, Co-Investigator(s) and Research Nurse(s)and other designated research staff) will be trained on the study procedures by the RCC. In

addition, all investigators will be provided with materials detailing all the study procedures. Before activating the study at each site, the following documents must be provided to the ICC via the RCC:

- the PI's up-to-date curriculum vitae (CV), in English signed and dated
- signed confidentiality agreement
- signed investigator agreement
- the formal ethics approval
- the signed clinical trials agreement
- the signed cluster guardian consent
- approved version of the patient information and CF
- approved version of the protocol (translation if applicable)

The CVs, GCP, NIHSS and mRS certification, of all staff involved in the study, listed in the delegation log, must also be signed, dated and provided to the ICC via the RCC during the course of the study.

9. PROCESS EVALUATION

The intervention in the INTERACT3 study is a goal-directed care bundle that includes intensive BP lowering, glycaemic control, treatment of pyrexia and reversal of anticoagulation. In order to explore how the care bundle, a complex intervention, is implemented, as well as to understand clinicians' perspectives, a prospective process evaluation will be conducted alongside the trial implementation. Intervention fidelity, reach, dose, adaption, feasibility and appropriateness of the goal-directed care bundle will be evaluated within the trial. Contextual conditions (current policies, settings resources etc.) that may have impacted on the quality of the implementation will be assessed.

Mixed methods will be used to address the objectives of process evaluation. The evaluation questions and indicators for evaluation are informed by the Medical Research Council (MRC) [61] process evaluation framework. The Normalisation Process Theory (NPT), which has been used for assessing how guidelines are embedded into routine practice, will serve as a conceptual framework to explore systematically how overall and for each of the interventions in INTERACT3 were adopted into patient care.[62] To assess the implementation and mechanism of impact of the interventions, semi-structured interviews and non-participants observations will be conducted among the primary implementers (physicians and nurses) using structured interview guides and observation templates. Focused group discussions will be conducted with project operation staff and site principal investigators to explore facilitators and barriers to the care bundle delivery by different stakeholders. Observational records, including hospital organisation questionnaires, routine monitoring data, field notes and case report forms, will also be used to triangulate qualitative findings to assess implementation quality, acceptability of the care bundle, and contextual factors, that may have impacted on outcomes.

Sites involved in the process evaluations will be determined by purposive sampling in accordance with the pre-specified criteria (e.g. geographical location [urban vs. semi-urban, level of hospital [tertiary vs secondary], type of unit [neurological vs. neurosurgery], operation performance [high vs low-medium) to achieve representativeness. Considering the stepped-wedge design, the qualitative data will be collected at an early intervention phase after crossover, ideally at the time 5-10 patients have been enrolled but will depend on the randomisation group and volume of the sampling site. It is estimated that there the sampling will involve 4-6 implementers (unit manager, clinicians and

nurses) and 2-4 patients being interviewed from each site. However, the final number of interviews will be determined by saturation of the themes and resources permitting. A survey (based on the Normalisation Process Theory) to assess the overall implementation will be administered by the research staff at close out of the site.

Interviews and focus group discussions will be conducted face-to-face, audio recorded, professionally translated and transcribed verbatim, with informed consent from each participant. This data will be saved electronically in a secure system that will be password protected and only accessible to specified members of the research team. Interview transcripts and audio files will be uploaded into the software program NVivo V.9 to assist with data management.

Results of the process evaluation will be used to monitor and document project implementation as well as identify barriers which the coordinating team could potentially help address in a timely fashion, such as increasing clinician's confidence in management through additional training. A full process evaluation protocol is being developed which will provide more detailed information.

10. ECONOMIC EVALUATION

A multi-country within-trial economic evaluation will be conducted, from each healthcare system's perspective. Healthcare utilisation costs incurred within the initial hospital visit will be estimated using administratively collected hospital records. These records consist of clinical tests, staffing time (as measured by their salary), medications and length of stay within hospital. As part of their 6-month follow up, patients will be asked about any additional costs incurred with hospital readmissions, pharmaceutical costs and outpatient visits, as well as time taken off from work. Health-related quality of life as measured by the EQ-5D-3L will be used as the primary outcome at the 6-month period. To account for the correlation between costs and outcomes between individuals within a cluster (hospital sites), we will use multilevel modelling taking clusters as random-effects and time fixed-effects on costs and outcomes to generate an incremental cost-effectiveness ratio (cost per quality-adjusted life year). Individual-level uncertainty will be conducted through 1,000 bootstrap replications to capture the uncertainty around the probability that the INTERACT3 trial is cost-effective at different willingness to pay thresholds.

To capture costs and outcomes beyond the trial, a decision-analytic model will be developed to enable long-term morbidity, quality of life and survival to be simulated. It will draw on the literature and available databases to determine the transition probabilities between health states and the cost and quality of life associated with each. Incremental cost per Quality Adjusted Life Year gained will be determined by the model to determine the average costs and outcomes, discounted at appropriate rate, accrued in both arms to compare.

11. DATA MANAGEMENT

The internet based data management system is managed at the George Institute for Global Health, which has extensive experience in clinical trial data capture and security. The George Institute has in place system security SOP with VeriSign SSL digital certification and encrypted HTTPS connection. (IT-SOP-105 v1.4). Registration and data entry will be performed at the participating sites via the password protected, via the encrypted HTTPS connection. Only staff listed in the delegation log will be given unique individual password to access the internet-based data management system.

This system, developed at the CCC for data capturing and the data variables will have logic checks within the acceptable ranges and mandatory fields to ensure accuracy and reduce missing data. Reports and data query management will also be included in the system to assist with centralised online monitoring by the CCC and the RCC.

Paper CRFs will be provided for sites preferring to use these for the initial collection of data. These forms will be used as source document and will need to be signed and dated by the investigator completing the form.

All computerised forms will be electronically signed (by use of the unique password) by the authorised study staff and all changes made following the initial entry will have an electronic dated audit trail. It is the requirement that the collection of data and transfer of information for the 6 months follow-up assessment has to be approved by the local IRB for each site.

12. STATISTICAL CONSIDERATIONS

Sample Size

The study is designed with 90% power (p=0.05) to detect a 20% reduction in the odds (common odds ratio of 0.80) of a worse outcome using an ordinal logistic regression. Assuming a distribution of mRS in the usual care arm that is similar to the one observed in the standard BP arm of the INTERACT2 trial - i.e. 7.6%, 18.0%, 18.8%, 16.6%, 19.0%, 8.0% and 12.0% for scores of 0 to 6, respectively -, this corresponds to a 5.6% absolute improvement in the proportion of patients experiencing a bad outcome (mRS of 3-6), from 55.6% down to 50%. This also translates to a 10% relative risk reduction (relative risk of 0.90). This intervention assumes a greater treatment effect from the intensive care bundle over use of BP lowering alone (in INTERACT2, treatment effect was 4% absolute).

We anticipate recruiting a minimum of 110 sites in a stepped-wedge design consisting of 3 groups and 4 phases. Each group would therefore include approximately 36-37 sites. We assume an interclass correlation coefficient (ICC) of 0.044 between sites which is similar to that found in the INTERACT2 and recently completed Head Position in Acute Stroke Trial (HeadPoST) trials across Chinese sites. To demonstrate a treatment effect with 90% power and a 2-sided type-I error rate of 5%, each site would need to recruit an average of 18 patients per phase for a total sample size of 7,920 patients. Assuming that 5% of patients will have a missing outcome, each site would need to target an average of 19 patients per phase.

To allow for variability in the number of patients recruited at each site, with very large hospitals expected to recruit up to 50 patients per phase and smaller hospitals recruiting as little as 1 patient per phase, we derived an inflation factor using a conservative formula applicable to parallel cluster trials.[63] Using this approach, the sample size would need to be inflated by a factor of approximately 1.3; thus leading to a sample size of up to 25 patients per site per phase (11,000 patients in total). Given that this is a very conservative scenario and that the effect of variability in cluster sizes is expected to be mostly mitigated by the stratification by size,[64] we plan to target an average of 19 patients per site per phase for a total sample size of 8,360 patients. Assuming the worst case scenario for the effect of cluster size variability on power, this sample size would still provide at least 80% power.

All analyses will be undertaken at the patient level on an intention-to-treat basis at each centre using Generalised Estimating Equations (GEE) or random-effects regression to account for clustering. The primary outcome of a shift (improvement) in scores on the mRS [65] at 6 months will be analysed by means of an ordinal logistic regression), with mRS as a dependent variable with 7 levels (0 [no residual symptom] to 6 [death]). The secondary outcome of the NIHSS[59] score at 7 days will also be analysed similarly as the NIHSS[59] is considered as an ordinal endpoint with 7 levels.[66] Binary secondary outcomes will be analysed by means of standard GEE or random-effects regression with

a logistic link and/or time-to-event type endpoints using the Cox model with a sandwich formula or a frailty model.[67] For continuous outcomes random intercept linear regression model will be used.

The primary analysis will essentially be unadjusted but adjusted analyses can be carried out for the primary endpoint and secondary outcomes. All analysis will be adjusted for clustering within centre and for secular trends. No adjustment for multiplicity is planned as there are only a small number of pre-specified efficacy outcomes being investigated. A detailed analysis plan including mock tables will be finalised before study ending.

Descriptive statistics will be provided for safety data. The number of patients reporting any SAEs and the occurrence of specific SAEs will be tabulated. Tests of a treatment effect on specific SAE may be attempted using tests adjusted for clustering.

13. SUBSTUDY (SELECTED SITES ONLY)

Effects of treatment of haematoma Change in ICH

The effects of treatment on haematoma expansion and other indices including oedema will be evaluated in a sub-sample of 1000 patients (the earliest 7 recruitments in control group and the earliest 7 recruitments in intensive group for each site). Apart from the CT scan at baseline, repeat CT scans (24±3 hours and 7 days) are required. CT imaging will be conducted according to standardised techniques and must be uploaded to the INTERACT3 server, either directly from the hospital site (if they have suitable broadband internet) or via the RCC office. The LCC will keep a hard copy in an uncompressed DICOM format onto a CD-ROM for monitor site verification. Trial management is facilitated by an established internet-based system. The imaging data will be analysed centrally by experts who will be kept blind to the treatment allocation. The primary efficacy measure was proportional change (‘growth’) in haematoma volume at 24 hours. Clinical outcomes were assessed over 6 months.

14. PUBLICATIONS, REPORTS AND DATA SHARING

Publication of the main reports from the study will be in the name of the INTERACT3 Collaborative Investigators. Full editorial control will reside with a Writing Committee approved by the Steering Committee (SC).

Investigators have the right to publish or present the results of the study. However as this is a multicentre study, investigators must agree not to publish or publicly present any interim results of the study without the prior written permission of the SC. Investigators further must agree to provide the SC at least 30 days prior notice of any submission for publication or presentation for review, copies of abstract or manuscripts (including without limitation, text and PowerPoint presentation slides and any other texts of translations or medial presentations) that reports any study results.

The SC shall have the right to review and comment with regard to the accuracy of the information, the protection of the rights of any individual, ensure a fair and balance representation and in compliance with appropriate regulations. If there is any disagreement, the investigator will agree to meet with a SC member in an effort to discuss and resolve any disagreements.

Writing Committees will be formed from members of the various committees, statisticians, research fellows and investigators. They will prepare the main reports of the study to be published in the name of “INTERACT3 Investigators” with credit assigned to the collaborating investigators and other research staff. Presentations of the study findings will be made at national and international meetings concerned with the management of stroke cardiovascular disease, and hypertension.

Authors of publications must meet the International Committee of Medical Journal Editors (ICMJE) guidelines for authorship that follow:

- authors must make substantial contributions to the conception and design of the study, acquisition of data, or analysis of data and interpretation of results;
- authors must draft the publication or, during draft review, provide contributions (data analysis, interpretation, or other important intellectual content) leading to significant revision of the manuscript with agreement by the other authors;
- authors must provide approval of the final draft version of the manuscript before it is submitted to the journal for publication.

All contributors who do not meet the 3 criteria for authorship should be listed in an acknowledgments section within the publication, if allowed by the journal, per ICMJE guidelines for acknowledgement.

At the completion of the study, a copy of the INTERACT3 dataset will be given to the Department of Neurosurgery, West China Hospital, for secondary analysis according to an agreed analysis plan with The George Institute. In addition, data will be shared with other investigators on the study, and investigators from other institutions around the world, according to a strict data sharing agreement. Data sharing will be available from 12 months after publication of the main results. Investigators are to make a formal request for data sharing through the Research Office of The George Institute.

15. STUDY ORGANISATION

Central international coordination is from GI China, and the study will be overseen by an International SC comprised of world experts in the fields of stroke, neurocritical care, neurology, geriatrics, cardiovascular epidemiology and clinical trials. The CCC will also be supported by key grant holders and regional experts in the Operations Committee (OC). The PIs of participating hospitals (see study organisational chart) will be administratively tied through a structure designed to enhance effective communication, collaboration and study monitoring by maintaining operations through adherence to a common protocol.

Participating Sites

The *clinical network* includes sites in China, Chile, Peru, Mexico, Brazil, Vietnam, Pakistan, Nigeria and Iraq. The sites will involve emergency department, intensive care units, neurology wards and acute stroke units in the cluster intervention and consecutive registration of patients.

Responsibilities: Overall management and compliance with the study protocol and ICH-GCP guidelines at the site; study “champion” recruitment and training; protocol and intervention training of all staff, consecutive patient enrolment and implementation of intervention on admission, complete and quality data collection into the internet-based data management system, data query resolutions, obtain approval and liaison with local Hospital Research Ethics Committee/Institutional Review Board, adherence to local ethics guidelines and reporting requirements, serious adverse event reporting to local Hospital Research Ethics Committee/Institutional Review Board and the ICC in accordance with protocol.

Study Organisational Chart

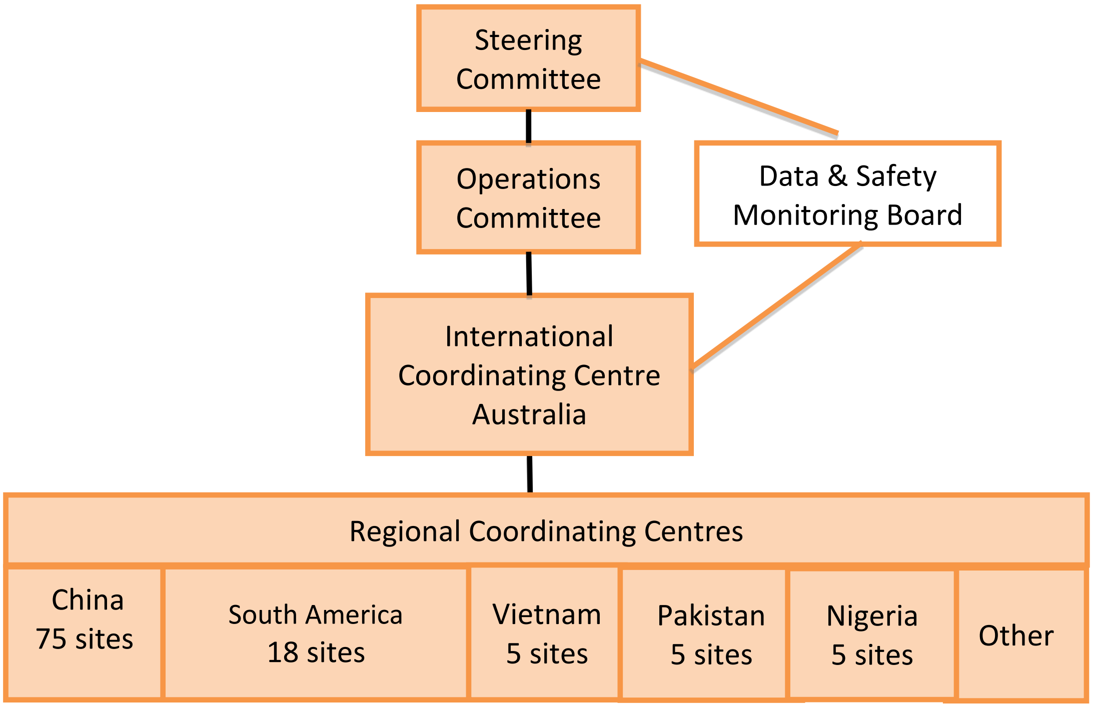

16. FUNDING

This study receives its principle funding from the following grants:

1) The West China Hospital Outstanding Discipline Development 1-3-5 Program (ZY2016102)
2) Program Grant from the National Health and Medical Research Council (NHMRC) of Australia (APP1149987)

Funding is also from Sichuan Credit Pharmaceutical CO., LTD, and Takeda (China) International Trading Co., Ltd

17. TIMELINES

Milestones for main study	2017	2018	2019	2020	2021	2022
Set-up of regional centres						
Ethics applications						
Training of sites						
Commence intervention and recruitment						
Outcomes assessment						
Close out						
Analysis and Results						
Presentation and publication of main result						x

18. REFERENCES

1. van Asch CJ, Luitse MJ, Rinkel GJ, van der Tweel I, Algra A, Klijn CJ. Incidence, case fatality, and functional outcome of intracerebral haemorrhage over time, according to age, sex, and ethnic origin: a systematic review and meta-analysis. Lancet neurology 2010;9:167-176.
2. Krishnamurthi RV, Feigin VL, Forouzanfar MH, et al. Global and regional burden of first-ever ischaemic and haemorrhagic stroke during 1990-2010: findings from the Global Burden of Disease Study 2010. The Lancet Global health 2013;1:e259-281.
3. Smith EE, Gurol ME, Eng JA, et al. White matter lesions, cognition, and recurrent hemorrhage in lobar intracerebral hemorrhage. Neurology 2004;63:1606-1612.
4. Poon MT, Fonville AF, Al-Shahi Salman R. Long-term prognosis after intracerebral haemorrhage: systematic review and meta-analysis. Journal of neurology, neurosurgery, and psychiatry 2014;85:660-667.
5. Pendlebury ST, Rothwell PM. Prevalence, incidence, and factors associated with pre-stroke and post-stroke dementia: a systematic review and meta-analysis. Lancet neurology 2009;8:1006-1018.
6. Anderson CS, Heeley E, Huang Y, et al. Rapid blood-pressure lowering in patients with acute intracerebral hemorrhage. The New England journal of medicine 2013;368:2355-2365.
7. van den Berghe G, Wouters P, Weekers F, et al. Intensive insulin therapy in critically ill patients. The New England journal of medicine 2001;345:1359-1367.
8. Middleton S, McElduff P, Ward J, et al. Implementation of evidence-based treatment protocols to manage fever, hyperglycaemia, and swallowing dysfunction in acute stroke (QASC): a cluster randomised controlled trial. The Lancet 2011;378:1699-1706.
9. Willmot M, Leonardi-Bee J, Bath PM. High blood pressure in acute stroke and subsequent outcome: a systematic review. Hypertension 2004;43:18-24.
10. Qureshi AI, Ezzeddine MA, Nasar A, et al. Prevalence of elevated blood pressure in 563,704 adult patients with stroke presenting to the ED in the United States. The American journal of emergency medicine 2007;25:32-38.
11. Qureshi AI, Palesch YY, Barsan WG, et al. Intensive Blood-Pressure Lowering in Patients with Acute Cerebral Hemorrhage. The New England journal of medicine 2016;375:1033-1043.
12. Arima H, Heeley E, Delcourt C, et al. Optimal achieved blood pressure in acute intracerebral hemorrhage: INTERACT2. Neurology 2015;84:464-471.
13. Kazui S, Minematsu K, Yamamoto H, Sawada T, Yamaguchi T. Predisposing factors to enlargement of spontaneous intracerebral hematoma. Stroke; a journal of cerebral circulation 1997;28:2370-2375.
14. Broderick JP, Diringer MN, Hill MD, et al. Determinants of intracerebral hemorrhage growth: an exploratory analysis. Stroke; a journal of cerebral circulation 2007;38:1072-1075.
15. Song EC, Chu K, Jeong SW, et al. Hyperglycemia exacerbates brain edema and perihematomal cell death after intracerebral hemorrhage. Stroke; a journal of cerebral circulation 2003;34:2215-2220.
16. Kimura K, Iguchi Y, Inoue T, et al. Hyperglycemia independently increases the risk of early death in acute spontaneous intracerebral hemorrhage. Journal of the neurological sciences 2007;255:90-94.
17. Tan X, He J, Li L, et al. Early hyperglycaemia and the early-term death in patients with spontaneous intracerebral haemorrhage: a meta-analysis. Internal medicine journal 2014;44:254-260.
18. Standards of medical care in diabetes--2015: summary of revisions. Diabetes care 2015;38 Suppl:S4.
19. Yamada T, Shojima N, Noma H, Yamauchi T, Kadowaki T. Glycemic control, mortality, and hypoglycemia in critically ill patients: a systematic review and network meta-analysis of randomized controlled trials. Intensive care medicine 2016.

20. Greer DM, Funk SE, Reaven NL, Ouzounelli M, Uman GC. Impact of fever on outcome in patients with stroke and neurologic injury: a comprehensive meta-analysis. Stroke; a journal of cerebral circulation 2008;39:3029-3035.
21. Badjatia N. Fever control in the neuro-ICU: why, who, and when? Current opinion in critical care 2009;15:79-82.
22. Sacho RH, Vail A, Rainey T, King AT, Childs C. The effect of spontaneous alterations in brain temperature on outcome: a prospective observational cohort study in patients with severe traumatic brain injury. Journal of neurotrauma 2010;27:2157-2164.
23. Schwarz S, Hafner K, Aschoff A, Schwab S. Incidence and prognostic significance of fever following intracerebral hemorrhage. Neurology 2000;54:354-361.
24. Rincon F, Lyden P, Mayer SA. Relationship between temperature, hematoma growth, and functional outcome after intracerebral hemorrhage. Neurocritical care 2013;18:45-53.
25. Reith J, Jorgensen HS, Pedersen PM, et al. Body temperature in acute stroke: relation to stroke severity, infarct size, mortality, and outcome. Lancet 1996;347:422-425.
26. Leira R, Davalos A, Silva Y, et al. Early neurologic deterioration in intracerebral hemorrhage: predictors and associated factors. Neurology 2004;63:461-467.
27. Michenfelder JD, Milde JH. The relationship among canine brain temperature, metabolism, and function during hypothermia. Anesthesiology 1991;75:130-136.
28. Steiner T, Al-Shahi Salman R, Beer R, et al. European Stroke Organisation (ESO) guidelines for the management of spontaneous intracerebral hemorrhage. International journal of stroke : official journal of the International Stroke Society 2014;9:840-855.
29. Hemphill JC, Greenberg SM, Anderson CS, et al. Guidelines for the Management of Spontaneous Intracerebral Hemorrhage. Stroke; a journal of cerebral circulation 2015;46:2032-2060.
30. den Hertog HM, van der Worp HB, van Gemert HM, et al. The Paracetamol (Acetaminophen) In Stroke (PAIS) trial: a multicentre, randomised, placebo-controlled, phase III trial. Lancet neurology 2009;8:434-440.
31. de Ridder IR, de Jong FJ, den Hertog HM, et al. Paracetamol (Acetaminophen) in stroke 2 (PAIS 2): protocol for a randomized, placebo-controlled, double-blind clinical trial to assess the effect of high-dose paracetamol on functional outcome in patients with acute stroke and a body temperature of 36.5 degrees C or above. International journal of stroke : official journal of the International Stroke Society 2015;10:457-462.
32. Steiner T, Rosand J, Diringer M. Intracerebral hemorrhage associated with oral anticoagulant therapy: current practices and unresolved questions. Stroke; a journal of cerebral circulation 2006;37:256-262.
33. Flaherty ML, Haverbusch M, Sekar P, et al. Location and outcome of anticoagulant-associated intracerebral hemorrhage. Neurocritical care 2006;5:197-201.
34. Rosand J, Eckman MH, Knudsen KA, Singer DE, Greenberg SM. The effect of warfarin and intensity of anticoagulation on outcome of intracerebral hemorrhage. Archives of internal medicine 2004;164:880-884.
35. Parry-Jones A. Cutting delays in reversing anticoagulation after intracerebral haemorrhage: three key changes at a UK comprehensive stroke centre. BMJ quality improvement reports 2015;4.
36. Kuramatsu JB, Gerner ST, Schellinger PD, et al. Anticoagulant reversal, blood pressure levels, and anticoagulant resumption in patients with anticoagulation-related intracerebral hemorrhage. JAMA : the journal of the American Medical Association 2015;313:824-836.
37. Steiner T, Poli S, Griebe M, et al. Fresh frozen plasma versus prothrombin complex concentrate in patients with intracranial haemorrhage related to vitamin K antagonists (INCH): a randomised trial. Lancet neurology 2016;15:566-573.
38. Parry-Jones AR, Di Napoli M, Goldstein JN, et al. Reversal strategies for vitamin K antagonists in acute intracerebral hemorrhage. Annals of neurology 2015;78:54-62.

39. de Greef BT, Schreuder FH, Vlooswijk MC, et al. Early seizures after intracerebral hemorrhage predict drug-resistant epilepsy. Journal of neurology 2015;262:541-546.
40. De Herdt V, Dumont F, Henon H, et al. Early seizures in intracerebral hemorrhage: incidence, associated factors, and outcome. Neurology 2011;77:1794-1800.
41. Bladin CF, Alexandrov AV, Bellavance A, et al. Seizures after stroke: a prospective multicenter study. Archives of neurology 2000;57:1617-1622.
42. Claassen J, Jette N, Chum F, et al. Electrographic seizures and periodic discharges after intracerebral hemorrhage. Neurology 2007;69:1356-1365.
43. Vespa PM, O'Phelan K, Shah M, et al. Acute seizures after intracerebral hemorrhage: a factor in progressive midline shift and outcome. Neurology 2003;60:1441-1446.
44. Passero S, Rocchi R, Rossi S, Ulivelli M, Vatti G. Seizures after spontaneous supratentorial intracerebral hemorrhage. Epilepsia 2002;43:1175-1180.
45. Arntz R, Rutten-Jacobs L, Maaijwee N, et al. Post-stroke epilepsy in young adults: a long-term follow-up study. PLoS One 2013;8:e55498.
46. Ferlazzo E, Gasparini S, Beghi E, et al. Epilepsy in cerebrovascular diseases: Review of experimental and clinical data with meta-analysis of risk factors. Epilepsia 2016;57:1205-1214.
47. Li Z, Zhao X, Wang Y, et al. Association between seizures and outcomes among intracerebral hemorrhage patients: the China National Stroke Registry. Journal of stroke and cerebrovascular diseases : the official journal of National Stroke Association 2015;24:455-464.
48. Donner A, Donald A. Analysis of data arising from a stratified design with the cluster as unit of randomization. Statistics in medicine 1987;6:43-52.
49. Donner A. Sample size requirements for stratified cluster randomization designs. Statistics in medicine 1992;11:743-750.
50. Fayers PM, Jordhoy MS, Kaasa S. Cluster-randomized trials. Palliative medicine 2002;16:69-70.
51. Barker D, McElduff P, D'Este C, Campbell MJ. Stepped wedge cluster randomised trials: a review of the statistical methodology used and available. BMC medical research methodology 2016;16:69.
52. Hussey MA, Hughes JP. Design and analysis of stepped wedge cluster randomized trials. Contemporary clinical trials 2007;28:182-191.
53. Hanger HC, Wilkinson T, Keeling S, Sainbury R. New Zealand guideline for management of stroke. The New Zealand medical journal 2004;117:U863.
54. Foundation NS. Clinical guidelines for stroke management 2010. 2010.
55. Cadilhac D, Kilkenny D, Churiloc L, Harris D, Lalor E. Identification of a reliable subset of process indicators for clinical audit in stroke care: an example from Australia. Clinical Audit 2010:67-77.
56. Harris D, Cadilhac D, Hankey GJ, Hillier S, Kilkenny D, Lalor E. National Stroke Audit: The Australian Experience. Clinical Audit 2010:25-31.
57. Devos P, Preiser JC. Tight blood glucose control: a recommendation applicable to any critically ill patient? Critical care 2004;8:427-429.
58. Saver JL. Optimal end points for acute stroke therapy trials: best ways to measure treatment effects of drugs and devices. Stroke 2011;42:2356-2362.
59. Brott T, Adams HP, Jr., Olinger CP, et al. Measurements of acute cerebral infarction: a clinical examination scale. Stroke 1989;20:864-870.
60. Rabin R, de Charro F. EQ-5D: a measure of health status from the EuroQol Group. Ann Med 2001;33:337-343.
61. Moore GF, Audrey S, Barker M, Bond L, Bonell C, Hardeman W, et al. Process evaluation of complex interventions: Medical research council guidance. *BMJ : British Medical Journal*. 2015;350
62. May C, Finch T. Implementing, embedding, and integrating practices: An outline of normalization process theory. *Sociology*. 2009;43:535-554

63. Girling AJ. Relative efficiency of unequal cluster sizes in stepped wedge and other trial designs under longitudinal or cross-sectional sampling.Stat Med. 2018 Dec 30;37(30):4652-4664. doi: 10.1002/sim.7943.
64. Martin JT, Hemming K, Girling A. The impact of varying cluster size in cross-sectional stepped-wedge cluster randomised trials. BMC Med Res Methodol. 2019 Jun 14;19(1):123. doi: 10.1186/s12874-019-0760-6
65. Bruno A, Shah N, Lin C, et al. Improving modified Rankin Scale assessment with a simplified questionnaire. Stroke 2010;41:1048-1050.
66. Kerr DM, Fulton RL, Lees KR. Seven-day NIHSS is a sensitive outcome measure for exploratory clinical trials in acute stroke: evidence from the Virtual International Stroke Trials Archive. Stroke 2012;43:1401-1403.
67. Lin DY, Wei LJ. The robust inference for the Cox proportional hazard model. J Am Stat Assoc 1989;84:1074-1078.

APPENDIX 1 - WORLD MEDICAL ASSOCIATION DECLARATION OF HELSINKI

Ethical Principles for Medical Research Involving Human Subjects

Adopted by the 18th WMA General Assembly, Helsinki, Finland, June 1964, and amended by the
29th WMA General Assembly, Tokyo, Japan, October 1975
35th WMA General Assembly, Venice, Italy, October 1983
41st WMA General Assembly, Hong Kong, September 1989
48th WMA General Assembly, Somerset West, Republic of South Africa, October 1996
and the 52nd WMA General Assembly, Edinburgh, Scotland, October 2000
Note of Clarification on Paragraph 29 added by the WMA General Assembly, Washington 2002
Note of Clarification on Paragraph 30 added by the WMA General Assembly, Tokyo 2004

A. INTRODUCTION

1. The World Medical Association has developed the Declaration of Helsinki as a statement of ethical principles to provide guidance to physicians and other participants in medical research involving human subjects. Medical research involving human subjects includes research on identifiable human material or identifiable data.

2. It is the duty of the physician to promote and safeguard the health of the people. The physician's knowledge and conscience are dedicated to the fulfilment of this duty.

3. The Declaration of Geneva of the World Medical Association binds the physician with the words, "The health of my patient will be my first consideration," and the International Code of Medical Ethics declares that, "A physician shall act only in the patient's interest when providing medical care which might have the effect of weakening the physical and mental condition of the patient."

4. Medical progress is based on research which ultimately must rest in part on experimentation involving human subjects.

5. In medical research on human subjects, considerations related to the well-being of the human subject should take precedence over the interests of science and society.

6. The primary purpose of medical research involving human subjects is to improve prophylactic, diagnostic and therapeutic procedures and the understanding of the aetiology and pathogenesis of disease. Even the best proven prophylactic, diagnostic, and therapeutic methods must continuously be challenged through research for their effectiveness, efficiency, accessibility and quality.

7. In current medical practice and in medical research, most prophylactic, diagnostic and therapeutic procedures involve risks and burdens.

8. Medical research is subject to ethical standards that promote respect for all human beings and protect their health and rights. Some research populations are vulnerable and need special protection. The particular needs of the economically and medically disadvantaged must be recognized. Special attention is also required for those who cannot give or refuse consent for themselves, for those who may be subject to giving consent under duress, for those who will not benefit personally from the research and for those for whom the research is combined with care.

9. Research Investigators should be aware of the ethical, legal and regulatory requirements for research on human subjects in their own countries as well as applicable international requirements. No national ethical, legal or regulatory requirement should be allowed to reduce or eliminate any of the protections for human subjects set forth in this Declaration.

B. BASIC PRINCIPLES FOR ALL MEDICAL RESEARCH

10. It is the duty of the physician in medical research to protect the life, health, privacy, and dignity of the human subject.

11. Medical research involving human subjects must conform to generally accepted scientific principles, be based on a thorough knowledge of the scientific literature, other relevant sources of information, and on adequate laboratory and, where appropriate, animal experimentation.

12. Appropriate caution must be exercised in the conduct of research which may affect the environment, and the welfare of animals used for research must be respected.

13. The design and performance of each experimental procedure involving human subjects should be clearly formulated in an experimental protocol. This protocol should be submitted for consideration, comment, guidance, and where appropriate, approval to a specially appointed ethical review committee, which must be independent of the investigator, the sponsor or any other kind of undue influence. This independent committee should be in conformity with the laws and regulations of the country in which the research experiment is performed. The committee has the right to monitor ongoing trials. The researcher has the obligation to provide monitoring information to the committee, especially any serious adverse events. The researcher should also submit to the committee, for review, information regarding funding, sponsors, institutional affiliations, other potential conflicts of interest and incentives for subjects.

14. The research protocol should always contain a statement of the ethical considerations involved and should indicate that there is compliance with the principles enunciated in this Declaration.

15. Medical research involving human subjects should be conducted only by scientifically qualified persons and under the supervision of a clinically competent medical person. The responsibility for the human subject must always rest with a medically qualified person and never rest on the subject of the research, even though the subject has given consent.

16. Every medical research project involving human subjects should be preceded by careful assessment of predictable risks and burdens in comparison with foreseeable benefits to the subject or to others. This does not preclude the participation of healthy volunteers in medical research. The design of all studies should be publicly available.

17. Physicians should abstain from engaging in research projects involving human subjects unless they are confident that the risks involved have been adequately assessed and can be satisfactorily managed. Physicians should cease any investigation if the risks are found to outweigh the potential benefits or if there is conclusive proof of positive and beneficial results.
18. Medical research involving human subjects should only be conducted if the importance of the objective outweighs the inherent risks and burdens to the subject. This is especially important when the human subjects are healthy volunteers.
19. Medical research is only justified if there is a reasonable likelihood that the populations in which the research is carried out stand to benefit from the results of the research.
20. The subjects must be volunteers and informed participants in the research project.
21. The right of research subjects to safeguard their integrity must always be respected. Every precaution should be taken to respect the privacy of the subject, the confidentiality of the patient's information and to minimize the impact of the study on the subject's physical and mental integrity and on the personality of the subject.
22. In any research on human beings, each potential subject must be adequately informed of the aims, methods, sources of funding, any possible conflicts of interest, institutional affiliations of the researcher, the anticipated benefits and potential risks of the study and the discomfort it may entail. The subject should be informed of the right to abstain from participation in the study or to withdraw consent to participate at any time without reprisal. After ensuring that the subject has understood the information, the physician should then obtain the subject's freely-given informed consent, preferably in writing. If the consent cannot be obtained in writing, the non-written consent must be formally documented and witnessed.
23. When obtaining informed consent for the research project the physician should be particularly cautious if the subject is in a dependent relationship with the physician or may consent under duress. In that case the informed consent should be obtained by a well-informed physician who is not engaged in the investigation and who is completely independent of this relationship.
24. For a research subject who is legally incompetent, physically or mentally incapable of giving consent or is a legally incompetent minor, the investigator must obtain informed consent from the legally authorized representative in accordance with applicable law. These groups should not be included in research unless the research is necessary to promote the health of the population represented and this research cannot instead be performed on legally competent persons.
25. When a subject deemed legally incompetent, such as a minor child, is able to give assent to decisions about participation in research, the investigator must obtain that assent in addition to the consent of the legally authorized representative.

26. Research on individuals from whom it is not possible to obtain consent, including proxy or advance consent, should be done only if the physical/mental condition that prevents obtaining informed consent is a necessary characteristic of the research population. The specific reasons for involving research subjects with a condition that renders them unable to give informed consent should be stated in the experimental protocol for consideration and approval of the review committee. The protocol should state that consent to remain in the research should be obtained as soon as possible from the individual or a legally authorized surrogate.

27. Both authors and publishers have ethical obligations. In publication of the results of research, the investigators are obliged to preserve the accuracy of the results. Negative as well as positive results should be published or otherwise publicly available. Sources of funding, institutional affiliations and any possible conflicts of interest should be declared in the publication. Reports of experimentation not in accordance with the principles laid down in this Declaration should not be accepted for publication.

C. ADDITIONAL PRINCIPLES FOR MEDICAL RESEARCH COMBINED WITH MEDICAL CARE

28. The physician may combine medical research with medical care, only to the extent that the research is justified by its potential prophylactic, diagnostic or therapeutic value. When medical research is combined with medical care, additional standards apply to protect the patients who are research subjects.

29. The benefits, risks, burdens and effectiveness of a new method should be tested against those of the best current prophylactic, diagnostic, and therapeutic methods. This does not exclude the use of placebo, or no treatment, in studies where no proven prophylactic, diagnostic or therapeutic method exists. See footnote

30. At the conclusion of the study, every patient entered into the study should be assured of access to the best proven prophylactic, diagnostic and therapeutic methods identified by the study. See footnote

31. The physician should fully inform the patient which aspects of the care are related to the research. The refusal of a patient to participate in a study must never interfere with the patient-physician relationship.

32. In the treatment of a patient, where proven prophylactic, diagnostic and therapeutic methods do not exist or have been ineffective, the physician, with informed consent from the patient, must be free to use unproven or new prophylactic, diagnostic and therapeutic measures, if in the physician's judgement it offers hope of saving life, re-establishing health or alleviating suffering. Where possible, these measures should be made the object of research, designed to evaluate their safety and efficacy. In all cases, new information should be recorded and, where appropriate, published. The other relevant guidelines of this Declaration should be followed.

Note: **Note of clarification on paragraph 29 of the WMA Declaration of Helsinki**

The WMA hereby reaffirms its position that extreme care must be taken in making use of a placebo-controlled trial and that in general this methodology should only be used in the absence of existing proven therapy. However, a placebo-controlled trial may be ethically acceptable, even if proven therapy is available, under the following circumstances:

- Where for compelling and scientifically sound methodological reasons its use is necessary to determine the efficacy or safety of a prophylactic, diagnostic or therapeutic method; or
- Where a prophylactic, diagnostic or therapeutic method is being investigated for a minor condition and the patients who receive placebo will not be subject to any additional risk of serious or irreversible harm.

All other provisions of the Declaration of Helsinki must be adhered to, especially the need for appropriate ethical and scientific review.

Note: **Note of clarification on paragraph 30 of the WMA Declaration of Helsinki**

The WMA hereby reaffirms its position that it is necessary during the study planning process to identify post-trial access by study participants to prophylactic, diagnostic and therapeutic procedures identified as beneficial in the study or access to other appropriate care. Post-trial access arrangements or other care must be described in the study protocol so the ethical review committee may consider such arrangements during its review.

The Declaration of Helsinki (Document 17.C) is an official policy document of the World Medical Association, the global representative body for physicians. It was first adopted in 1964 (Helsinki, Finland) and revised in 1975 (Tokyo, Japan), 1983 (Venice, Italy), 1989 (Hong Kong), 1996 (Somerset-West, South Africa) and 2000 (Edinburgh, Scotland). Note of clarification on Paragraph 29 added by the WMA General Assembly, Washington 2002.

APPENDIX 2 - BP management protocol

Appendix 2A - BP protocol for centres with Urapidil (China)

**Early intensive BP lowering group**	**TREATMENT PROTOCOL**
**INITIAL therapy**	
BP Target	**SBP 130-140 mmHg** reached within 60 minutes after diagnosis of ICH
Monitoring	• Continuous HR monitoring<br>• Record BP/HR q 5 mins during active treatment, then q 15 min for first hour, q 30 min for next 5 hours and then hourly to 24 h
Urapidil (IV)	• Urapidil test dose: 5 mg IV bolus over 1 minute<br>• If SBP ≥ 140 mmHg and HR >55 bpm, repeat 5 mg bolus in 5 minutes<br>• 10-25 mg IV push q 5 mins until target SBP reached (< 140mmHg) or HR <55 bpm<br>• **If HR increases by >15 bpm or is >90 bpm, add IV beta blocker**
Hydralazine (IV)	If BP persistently >140 mmHg:<br>• ADD Hydralazine with a test dose: 5 mg IV bolus over 1 minute<br>• If SBP ≥ 140 mmHg, repeat 5 mg IV bolus in 5 minutes<br>• If SBP still > 140mmHg, give 10 mg IV bolus q 5 mins until target SBP reached. Increase to 20 mg bolus if required<br>• Maximum hydralazine dose = 240mg/24 hours
Glyceryl Trinitrate (Topical)	If BP persistently > 140 mmHg:<br>• ADD topical glyceryl trinitrate (paste or patch) at a rate of 5-10 mg/24hour<br>(≈200-400 μg/hour). NB: also known as topical nitroglycerin
Continuous IV Infusions (requires ICU admission)	If BP persistently >140 mmHg: **NB: It is recognized that many sites will proceed directly to urapidil infusion following an initial bolus.**<br>• Urapidil infusion 5-30 mg/hour<br>• If target still not reached, ADD infusion of hydralazine 50-150 μg/min OR<br>glyceryl trinitrate 1-100 μg/Kg/min

**MAINTENANCE therapy**	
BP Target	Maintenance of SBP 130-140 mmHg
Monitoring	Once SBP is under target (confirmed by 4 readings 15 minutes apart):<br>• Record BP/HR q 30 minutes for 5 hours and then q 1 h for 18 h.

IV treatment prn	If SBP exceeds 140mmHg at any point:<br>• Give Urapidil (10-25 mg) **and/or** hydralazine (10-20 mg) boluses. BP and HR should then be recorded 5 and 15 minutes later<br>• If SBP is 130-140mmHg, Urapidil 10-25 mg (dose dependent on initial response) should be administered q 6 hours for the first 24 hours after symptom onset (total of 3 doses)<br>• If SBP ≤ 120 mmHg or HR < 55 bpm, then cease treatment<br>• **If HR increases by >15 bpm or is >90 bpm, add IV beta blocker**<br>• Note: urapidil and hydralazine may be used together during the maintenance phase
Oral treatment	Start treatment by 24 hours (use nasogastric if required)<br>• If not contraindicated and no other drug is specifically indicated, start combination therapy of ACEI + diuretics in addition to previous anti-hypertensives

Note: Monoamine oxidase inhibitors are not recommended with this BP lowering agent and phosphodiesterase inhibitors must not be used with GTN.

Key to abbreviations: ACEI – Angiotensin converting enzyme inhibitor; BP – blood pressure; bpm – beats per minute; HR – heart rate; ICU – intensive care unit; q – every; prn – as required; μg/Kg/min – micrograms per kilogram per minute; μg/min – micrograms per minute.

Appendix 2B - BP protocol for centres with Phentolamine (China)

Early intensive BP lowering group	TREATMENT PROTOCOL
**INITIAL therapy**	
BP Target	**SBP 130-140 mmHg** reached within 60 minutes after diagnosis of ICH
Monitoring	• Continuous HR monitoring<br>• Record BP/HR q 5 mins during <u>active</u> treatment, then q 15 min for first hour, q 30 min for next 5 hours and then hourly to 24 h
Phentolamine (IV)	• Phentolamine test dose: 2.5 mg IV bolus over 1 minute<br>• If SBP ≥ 150 mmHg and HR >55 bpm, repeat 2.5 mg bolus in 5 minutes<br>• 5 mg IV push q 5 mins until target SBP reached (< 140mmHg) or HR <55 bpm<br>• **If HR increases by >15 bpm or is >90 bpm, add IV beta blocker**
Hydralazine (IV)	If BP persistently >150 mmHg:<br>• ADD Hydralazine with a test dose: 5 mg IV bolus over 1 minute<br>• If SBP ≥ 150 mmHg, repeat 5 mg IV bolus in 5 minutes<br>• If SBP still >150mmHg, give 10 mg IV bolus q 5 mins until target SBP reached. Increase to 20 mg bolus if required<br>• Maximum hydralazine dose = 240mg/24 hours
Glyceryl Trinitrate (Topical)	If BP persistently >150 mmHg:<br>• ADD topical glyceryl trinitrate (paste or patch) at a rate of 5-10 mg/24hour (≈200-400 µg/hour).<br>NB: also known as topical nitroglycerin
Continuous IV Infusions (requires ICU admission)	If BP persistently >150 mmHg:<br>• Phentolamine infusion 0.2-5 mg/minute<br>• If target still not reached, ADD infusion of hydralazine 50-150 µg/min OR<br>glyceryl trinitrate 1-100 µg/Kg/min

MAINTENANCE therapy	
BP Target	Maintenance of SBP 140-150 mmHg
Monitoring	Once SBP is under target (confirmed by 4 readings 15 minutes apart):<br>• Record BP/HR q 30 minutes for 5 hours and then q 1 h for 18 h.

IV treatment prn	If SBP exceeds 150mmHg at any point:<br>• Give Phentolamine (5 mg) **and/or** hydralazine (10-20 mg) boluses. BP and HR should then be recorded 5 and 15 minutes later<br>• If SBP is 140-150mmHg, Phentolamine 5 mg (dose dependent on initial response) should be administered q 6 hours for the first 24 hours after symptom onset (total of 3 doses)<br>• If SBP ≤ 130 mmHg or HR <55 bpm, then cease treatment<br>• **If HR increases by >15 bpm or is >90 bpm, add IV beta blocker**<br>• Note: phentolamine and hydralazine may be used together during the maintenance phase
Oral treatment	Start treatment by 24 hours (use nasogastric if required)<br>• If not contraindicated and no other drug is specifically indicated, start combination therapy of ACEI + diuretics in addition to previous anti-hypertensives

Note: Monoamine oxidase inhibitors are not recommended with this BP lowering agent and phosphodiesterase inhibitors must not be used with GTN.

Key to abbreviations: ACEI – Angiotensin converting enzyme inhibitor; BP – blood pressure; bpm – beats per minute; HR – heart rate; ICU – intensive care unit; q – every; prn – as required; μg/Kg/min – micrograms per kilogram per minute; μg/min – micrograms per minute.

Appendix 2C - BP management protocol WITHOUT Labetalol （out of China）

Early intensive BP lowering group	TREATMENT PROTOCOL
**INITIAL therapy**	
BP Target	**SBP 130-140 mmHg** reached within 60 minutes after diagnosis of ICH
Monitoring	• Continuous HR monitoring<br>• Record BP/HR q 5 mins during active treatment, then q 15 min for first hour, q 30 min for next 5 hours and then hourly to 24 h
Hydralazine (IV)	• Hydralazine test dose: 5 mg IV bolus over 1 minute<br>• If SBP ≥ 140 mmHg, repeat 5 mg IV bolus in 5 minutes<br>• If SBP still > 140mmHg, give 10 mg IV bolus q 5 mins until target SBP reached<br>• Increase to 20 mg bolus if required<br>• Maximum hydralazine dose = 240mg
Metoprolol (IV)	If BP persistently > 140 mmHg:<br>• ADD Metoprolol 5 mg IV bolus over 3-5 minutes, repeat 5mg bolus in 5 minutes x 2 if necessary but do NOT give if HR<55bpm
Glyceryl Trinitrate (topical)	If BP persistently > 140 mmHg:<br>ADD topical glyceryl trinitrate (paste or patch) at a rate of 5-10 mg/24 hour (≈ 200-400 μg/hour). NB: also known as topical nitroglycerin
Continuous IV Infusions (requires ICU admission)	If BP persistently > 140 mmHg:<br>• Start infusion of hydralazine - 50-150 μg/min<br>• If target still not reached ADD infusion of glyceryl trinitrate 1-100 μg/Kg/min<br>• OR start infusion of Nicardipine 5-15 mg/hour

**MAINTENANCE therapy**	
BP Target	Maintenance of SBP 130-140 mmHg
Monitoring	Once SBP is under target (confirmed by 4 readings 15 minutes apart):<br>• Record BP/HR q 30 minutes for 5 hours and then q 1 h for 18 h
IV treatment prn	If SBP exceeds 140mmHg at any point:<br>• Give Hydralazine 10-20 mg boluses. BP and HR should then be recorded 5 and 15 minutes after each bolus<br>• If SBP is 130-140mmHg, give further Hydralazine 10-20 mg boluses (dependent on initial dose) q 6 hours for first 24 hours (total of 3 doses)<br>• If SBP ≤ 130 mmHg, cease therapy
Oral treatment	Start treatment by 24 hours (use nasogastric if required)

	• If not contraindicated and no other drug is specifically indicated, start combination therapy of ACEI + diuretics ± previous antihypertensives

Note: Monoamine oxidase inhibitors are not recommended with this BP lowering agent and phosphodiesterase inhibitors must not be used with GTN.

Key to abbreviations:

ACEI – Angiotensin converting enzyme inhibitor; BP – blood pressure; bpm – beats per minute; HR – heart rate; ICU – intensive care unit; q – every; prn – as required; μg/Kg/min – micrograms per kilogram per minute; μg/min – micrograms per minute.

Appendix 2D - BP management protocol for centres WITH labetalol (out of China)

Early intensive BP lowering group	TREATMENT PROTOCOL
**INITIAL therapy**	
BP Target	**SBP 130-140 mmHg** reached within 60 minutes after diagnosis of ICH
Monitoring	• Continuous HR monitoring<br>• Record BP/HR q 5 mins during active treatment, then q 15 min for first hour, q 30 min for next 5 hours and then hourly to 24 h
Labetalol (IV)	• Labetalol test dose: 10 mg IV bolus over 1 minute<br>• If SBP ≥ 140 mmHg and HR > 55 bpm, repeat 10 mg bolus in 5 minutes.<br>• 20 mg IV push q 5 mins until target SBP reached (< 140mmHg) or HR <55 bpm; increase to 40 mg bolus if required<br>• Maximum labetalol dose: 300 mg / 24 hours
Hydralazine (IV)	If BP persistently > 140 mmHg:<br>• ADD Hydralazine with a test dose: 5 mg IV bolus over 1 minute<br>• If SBP ≥ 140 mmHg, repeat 5 mg IV bolus in 5 minutes<br>• If SBP still >140mmHg, give 10 mg IV bolus q 5 mins until target SBP reached. Increase to 20 mg bolus if required<br>• Maximum hydralazine dose = 240mg/24 hours
Glyceryl Trinitrate (Topical)	If BP persistently > 140mmHg:<br>• ADD topical glyceryl trinitrate (paste or patch) at a rate of 5-10 mg/24hour<br>(≈200-400 µg/hour). NB: also known as topical nitroglycerin
Continuous IV Infusions (requires ICU admission)	If BP persistently > 140mmHg:<br>• Labetalol infusion 2-8 mg/min to a maximum of 300 mg/24 hours (consider this if response to labetalol boluses is adequate but brief)<br>• If target still not reached, ADD infusion of hydralazine 50-150 µg/min OR<br>glyceryl trinitrate 1-100 µg/Kg/min<br>• OR start infusion of Nicardipine 5-15 mg/hour
**MAINTENANCE therapy**	
BP Target	Maintenance of SBP 130-140 mmHg
Monitoring	Once SBP is under target (confirmed by 4 readings 15 minutes apart):<br>• Record BP/HR q 30 minutes for 5 hours and then q 1 h for 18 h

IV treatment prn	If SBP exceeds 140mmHg at any point:<br>• Give Labetalol (20-40 mg) **and/or** hydralazine (10-20 mg) boluses. BP and HR should then be recorded 5 and 15 minutes later<br>• If SBP is 130-140mmHg, Labetalol 10-40 mg (dose dependent on initial response) should be administered q 6 hours for the first 24 hours after symptom onset (total of 3 doses)<br>• If SBP ≤ 130 mmHg or HR < 55 bpm, then cease treatment.<br>• Maximum labetalol dose: 300 mg/24 hours<br>• **Note: labetalol and hydralazine may be used together during the maintenance phase**
Oral treatment	Start treatment by 24 hours (use nasogastric if required).<br>• If not contraindicated and no other drug is specifically indicated, start combination therapy of ACEI + diuretics in addition to previous anti-hypertensives

Note: Monoamine oxidase inhibitors are not recommended with labetalol. Phosphodiesterase inhibitors must not be used with GTN.

Key to abbreviations: ACEI – Angiotensin converting enzyme inhibitor; BP – blood pressure; bpm – beats per minute; HR – heart rate; ICU – intensive care unit; q – every; prn – as required; μg/Kg/min – micrograms per kilogram per minute; μg/min – micrograms per minute.

Appendix 2E- BP protocol for centres with Clevidipine （out of China）

**Early intensive BP lowering group**	**TREATMENT PROTOCOL**
**INITIAL therapy**	
BP Target	**SBP 130-140 mmHg** reached within 60 minutes of commencing rtPA
Monitoring	• Continuous HR monitoring<br>• Record BP/HR q 2 mins during <u>active</u> treatment, then q 15 min for first hour, q 30 min for next 5 hours and then hourly to 24 h
Clevidipine (Continuous IV Infusion)<br>*(requires ICU admission)*	• Clevidipine initiation dose: 2 mg/hour continuous IV for the first 1.5minuts<br>• If SBP ≥140mmHg, DOUBLE the dose every 2-10 minutes (4, 8, 16 and then 32 mg/hour)<br>• Maximum dose = 32.0mg/hour
Hydralazine (IV)	If BP persistently >140mmHg:<br>• ADD Hydralazine with a test dose: 5 mg IV bolus over 1 minute<br>• If SBP ≥140mmHg, repeat 5 mg IV bolus in 5 minutes<br>• If SBP still > 140mmHg, give 10 mg IV bolus q 5 mins until target SBP reached. Increase to 20 mg bolus if required<br>• Maximum hydralazine dose = 240mg/24 hours
Glyceryl Trinitrate (Topical)	If BP persistently >140mmHg:<br>• ADD topical glyceryl trinitrate (paste or patch) at a rate of 5-10 mg/24hour<br>(≈200-400 μg/hour). NB: also known as topical nitroglycerin
Continuous IV Infusions (requires ICU admission)	If BP persistently >140mmHg:<br>• ADD infusion of hydralazine 50-150 μg/min OR<br>glyceryl trinitrate 1-100 μg/Kg/min
**MAINTENANCE therapy**	
BP Target	Maintenance of SBP 130-140 mmHg
Monitoring	Once SBP is under target (confirmed by 4 readings 15 minutes apart):<br>• Record BP/HR q 30 minutes for 5 hours and then q 1 h for 18 h.
Continuous IV treatment prn	If SBP exceeds 140mmHg at any point:<br>• DOUBLE the dose of Clevidipine every 2-10 minutes (4, 8, 16 and then maximum dose of 32 mg/hour)<br>• If SBP is 130-140mmHg, Keep the dose of Clevidipine<br>• If SBP ≤ 120 mmHg or HR < 55 bpm, then HALVE the dose of Clevidipine every 2-10 minutes and then cease treatment<br>• **If HR increases by >15 bpm or is >90 bpm, add IV beta blocker**
Oral treatment	Start treatment by 24 hours (use nasogastric if required)

	• If not contraindicated and no other drug is specifically indicated, start combination therapy of ACEI + diuretics in addition to previous anti-hypertensives

Note: Monoamine oxidase inhibitors are not recommended with this BP lowering agent and phosphodiesterase inhibitors must not be used with GTN.

Key to abbreviations: ACEI – Angiotensin converting enzyme inhibitor; BP – blood pressure; bpm – beats per minute; HR – heart rate; ICU – intensive care unit; q – every; prn – as required; mg/hour – milligram per hour; µg/Kg/min – micrograms per kilogram per minute; µg/min – micrograms per minute.

Appendix 2F - Additional IV Medication for BP Use in China

The drugs listed in this Appendix are additional medications for BP lowering that can be used in China sites.

1. Suggested IV medication for BP lowering

1) Esmolol

Dosage and administration:

Bolus or infusion: It is recommended that an initial loading dose of 0.5 milligrams/kg body weight (500 micrograms/kg) infused over a one-minute duration, followed by a maintenance infusion of 0.05 milligrams/kg/min (50 micrograms/kg/min) for the next 4 minutes. If it is efficacious, the maintenance infusion may be continued at 0.05 mg/kg/min. If an adequate therapeutic effect is not observed, repeat the same loading dosage and follow with a maintenance infusion. The maintenance infusion may be continued at 0.05 mg/kg/min or increased step wise (e.g. 0.1 mg/kg/min, 0.15 mg/kg/min or a maximum of 0.2 mg/kg/min) with each step being maintained for 4 or more minutes. The maintenance infusion may be increased to a maximum of 0.3 mg/kg/min. Maintenance dosages above 200 □g/kg/min (0.2 mg/kg/min) have not been shown to have significantly increased benefits.

2) Enalaprilat

Dosage and administration:

Therapy should be individualised. For patients on diuretic therapy, the dosage of enalaprilat should be reduced. Dose in hypertension is 1.25 mg every six hours administered intravenously over a five minute period. Doses higher than 5 mg every six hours are not suggested.

2. IV medication for BP lowering which can also be used

1) Diltiazem

Dosage and administration:

An initial dose of 10 mg or 0.5 mg - 0.25 mg/kg body weight infused within 3 minutes can be used. Diltiazem should be diluted in normal or glucose solutions to a concentration of 1% before use. This dose can be repeated after 15 minutes. A maintenance infusion of 5 □g - 15 □g/kg/min is also permitted.

2) Nitroglyceride

Dosage and administration:

Nitroglyceride injection 10 mg is diluted in 0.9% normal solution 500 ml or 5% glucose solution 500 ml. The initial dose of nitroglyceride is 5 drops/min, and under close BP monitoring may increase by 5 drops/min every 3-5 minutes. If the dose of 20 drops/min is still not efficacious, 10 drops/min can be added every 3-5 minutes. Doses usually can be from 5 to 50 drops/min.

Note: Phosphodiesterase inhibitors must not be used with GTN.

3) Nimodipine

Dosage and administration:

Nimodipine 50 ml/50 mg should be put in a micro pump and infused in a constant speed 4 ml/hour, once a day. Usually it can be used for 5 to 14 days. Then, change to oral nimodipine. However, the BP lowering effect of oral nimodopine is not obvious.

4) Frusemide

Dosage and administration:

The usual initial dose of furosemide is 20-80 mg. If needed, the same dose can be repeated every 2 hours. The total dosage cannot be more than 1 g/d. If it is not effective, the dose should not be increased, to avoid renal toxicity.

APPENDIX 3 – Health Scales

Glasgow Coma Scale (GCS)

Assessment	Measure	Score
Eye opening (E)	4= Spontaneous<br>3= To sound<br>2= To pain<br>1= Never	
Verbal response (V)	5= Oriented<br>4= Confused conversation<br>3= Inappropriate words<br>2= Incomprehensible sounds<br>1= None	
Motor response (M)	6= Obeys command<br>5= Localises pain<br>4= Withdrawal flexion<br>3= Abnormal flexion<br>2= Extension<br>1= None	
**TOTAL**		**……. / 15**<br>(E + M + V)

NB. If the patient is intubated the verbal response should be scored 1.

When scoring the motor response, assess the response for the extremities of side unaffected by partial or complete paralysis.

NIH Stroke scale (National Institute of Health Stroke Scale)

Assessment	Response	Score
**1a. Level of Consciousness:** The investigator must choose a response, even if a full evaluation is prevented by such obstacles as an endotracheal tube, language barrier, orotracheal trauma/bandages. A 3 is scored only if the patient makes no movement (other than reflexive posturing) in response to noxious stimulation.	0 = Alert; keenly responsive.<br>1 = Not alert, but arousable by minor stimulation to obey, answer, or respond.<br>2 = Not alert, requires repeated stimulation to attend, or is obtunded and requires strong or painful stimulation to make movements (not stereotyped).<br>3 = Responds only with reflex motor or autonomic effects or totally unresponsive, flaccid, areflexic.	
**1b. LOC Questions:** The patient is asked the month and his/her age. The answer must be correct - there is no partial credit for being close. Aphasic and stuporous patients who do not comprehend the questions will score 2. Patients unable to speak because of endotracheal intubation, orotracheal trauma, severe dysarthria from any cause, language barrier or any other problem not secondary to aphasia are given a 1. It is important that only the initial answer be graded and that the examiner not "help" the patient with verbal or non-verbal cues.	0 = Answers both questions correctly.<br>1 = Answers one question correctly.<br>2 = Answers neither question correctly.	
**1c. LOC Commands:** The patient is asked to open and close the eyes and then to grip and release the non-paretic hand. Substitute another one step command if the hands cannot be used. Credit is given if an unequivocal attempt is made but not completed due to weakness. If the patient does not respond to command, the task should be demonstrated to them (pantomime) and score the result (i.e., follows none, one or two commands). Patients with trauma, amputation, or other physical impediments should be given suitable one-step commands. Only the first attempt is scored.	0 = Performs both tasks correctly.<br>1 = Performs one task correctly.<br>2 = Performs neither task correctly.	
**2. Best Gaze:** Only horizontal eye movements will be tested. Voluntary or reflexive (oculocephalic) eye movements will be scored but caloric testing is not done. If the patient has a conjugate deviation of	0 = Normal.<br>1 = Partial gaze palsy. This score is given when gaze is abnormal in one or both eyes, but where forced deviation or total gaze paresis are not present.	

Assessment	Response	Score
the eyes that can be overcome by voluntary or reflexive activity, the score will be 1. If a patient has an isolated peripheral nerve paresis (CN III, IV or VI) score a 1. Gaze is testable in all aphasic patients. Patients with ocular trauma, bandages, pre-existing blindness or other disorder of visual acuity or fields should be tested with reflexive movements and a choice made by the investigator. Establishing eye contact and then moving about the patient from side to side will occasionally clarify the presence of a partial gaze palsy.	2 = Forced deviation, or total gaze paresis not overcome by the oculocephalic maneuver.	
**3. Visual:**<br>Visual fields (upper and lower quadrants) are tested by confrontation, using finger counting or visual threat as appropriate. Patient must be encouraged, but if they look at the side of the moving fingers appropriately, this can be scored as normal. If there is unilateral blindness or enucleation, visual fields in the remaining eye are scored. Score 1 only if a clear-cut asymmetry, including quadrantanopia is found. If patient is blind from any cause score 3. Double simultaneous stimulation is performed at this point. If there is extinction patient receives a 1 and the results are used to answer question 11.	0 = No visual loss.<br>1 = Partial hemianopia.<br>2 = Complete hemianopia.<br>3 = Bilateral hemianopia (blind including cortical blindness).	
**4. Facial Palsy:**<br>Ask, or use pantomime to encourage the patient to show teeth or raise eyebrows and close eyes. Score symmetry of grimace in response to noxious stimuli in the poorly responsive or non-comprehending patient. If facial trauma/bandages, orotracheal tube, tape or other physical barrier obscures the face, these should be removed to the extent possible.	0 = Normal symmetrical movement.<br>1 = Minor paralysis (flattened nasolabial fold, asymmetry on smiling).<br>2 = Partial paralysis (total or near total paralysis of lower face).<br>3 = Complete paralysis of one or both sides (absence of facial movement in the upper and lower face).	

Assessment	Response	Score
**5 & 6. Motor Arm and Leg:** The limb is placed in the appropriate position: extend the arms (palms down) 90 degrees (if sitting) or 45 degrees (if supine) and the leg 30 degrees (always tested supine). Drift is scored if the arm falls before 10 seconds or the leg before 5 seconds. The aphasic patient is encouraged using urgency in the voice and pantomime but not noxious stimulation. Each limb is tested in turn, beginning with the non-paretic arm. Only in the case of amputation or joint fusion at the shoulder or hip may the score be "9" and the examiner must clearly write the explanation for scoring as a "9".	0 = No drift, limb holds 90 (or 45) degrees for full 10 seconds.<br>1 = Drift, Limb holds 90 (or 45) degrees, but drifts down before full 10 seconds; does not hit bed or other support.<br>2 = Some effort against gravity, limb cannot get to or maintain (if cued) 90 (or 45) degrees, drifts down to bed, but has some effort against gravity.<br>3 = No effort against gravity, limb falls.<br>4 = No movement<br>9 = Amputation, joint fusion explain:	-
	**5a. Left Arm**	
	**5b. Right Arm**	
	0 = No drift, leg holds 30 degrees position for full 5 seconds.<br>1 = Drift, leg falls by the end of the 5 second period but does not hit bed.<br>2 = Some effort against gravity; leg falls to bed by 5 seconds, but has some effort against gravity.<br>3 = No effort against gravity, leg falls to bed immediately.<br>4 = No movement.<br>9 = Amputation, joint fusion explain:	-
	**6a. Left Leg**	
	**6b. Right Leg**	
**7. Limb Ataxia:** This item is aimed at finding evidence of a unilateral cerebellar lesion. Test with eyes open. In case of visual defect, insure testing is done in intact visual field. The finger-nose-finger and heel-shin tests are performed on both sides, and ataxia is scored only if present out of proportion to weakness. Ataxia is absent in the patient who cannot understand or is paralyzed. Only in the case of amputation or joint fusion may the item be scored "9", and the examiner must clearly write the explanation for not scoring. In case of blindness test by touching nose from extended arm position.	0 = Absent .<br>1 = Present in one limb .<br>2 = Present in two limbs If present, is ataxia in?	
	**Right arm** 1 = Yes 2 = No<br>9 = amputation or joint fusion, explain: ________________	-
	**Left arm** 1 = Yes 2 = No<br>9 = amputation or joint fusion, explain : ________________	-
	**Right leg** 1 = Yes 2 = No<br>9 = amputation or joint fusion, explain: ________________	-
	**Left leg** 1 = Yes 2 = No<br>9 = amputation or joint fusion, explain: ________________	-

Assessment	Response	Score
**8. Sensory:**<br>Sensation or grimace to pin prick when tested, or withdrawal from noxious stimulus in the obtunded or aphasic patient. Only sensory loss attributed to stroke is scored as abnormal and the examiner should test as many body areas [arms (not hands), legs, trunk, face] as needed to accurately check for hemisensory loss. A score of 2, "severe or total," should only be given when a severe or total loss of sensation can be clearly demonstrated. Stuporous and aphasic patients will therefore probably score 1 or 0. The patient with brain stem stroke who has bilateral loss of sensation is scored 2. If the patient does not respond and is quadriplegic score 2. Patients in coma (item 1a=3) are arbitrarily given a 2 on this item.	0 = Normal; no sensory loss.<br>1 = Mild to moderate sensory loss; patient feels pinprick is less sharp or is dull on the affected side; or there is a loss of superficial pain with pinprick but patient is aware he/she is being touched.<br>2 = Severe to total sensory loss; patient is not aware of being touched in the face, arm, and leg.	
**9. Best Language:**<br>A great deal of information about comprehension will be obtained during the preceding sections of the examination. The patient is asked to describe what is happening in the attached picture, to name the items on the attached naming sheet, and to read from the attached list of sentences. Comprehension is judged from responses here as well as to all of the commands in the preceding general neurological exam. If visual loss interferes with the tests, ask the patient to identify objects placed in the hand, repeat, and produce speech. The intubated patient should be asked to write. The patient in coma (question 1a=3) will arbitrarily score 3 on this item. The examiner must choose a score in the patient with stupor or limited cooperation but a score of 3 should be used only if the patient is mute and follows no one step commands.	0 = No aphasia, normal.<br>1 = Mild to moderate aphasia; some obvious loss of fluency or facility of comprehension, without significant limitation on ideas expressed or form of expression. Reduction of speech and/or comprehension, however, makes conversation about provided material difficult or impossible. For example in conversation about provided materials examiner can identify picture or naming card from patient's response.<br>2 = Severe aphasia; all communication is through fragmentary expression; great need for inference, questioning, and guessing by the listener. Range of information that can be exchanged is limited; listener carries burden of communication. Examiner cannot identify materials provided from patient response.<br>3 = Mute, global aphasia; no usable speech or auditory comprehension.	
**10. Dysarthria:**<br>If patient is thought to be normal an adequate sample of speech must be obtained by asking patient to read or repeat words from the attached list. If the patient has severe aphasia, the clarity of	0 = Normal.<br>1 = Mild to moderate; patient slurs at least some words and, at worst, can be understood with some difficulty.	

Assessment	Response	Score
articulation of spontaneous speech can be rated. Only if the patient is intubated or has other physical barrier to producing speech, may the item be scored "9", and the examiner must clearly write an explanation for not scoring. Do not tell the patient why he/she is being tested.	2 = Severe; patient's speech is so slurred as to be unintelligible in the absence of or out of proportion to any dysphasia, or is mute/anarthric.<br>9 = Intubated or other physical barrier, explain: ________________	
**11. Extinction and Inattention (formerly Neglect):**<br>Sufficient information to identify neglect may be obtained during the prior testing. If the patient has a severe visual loss preventing visual double simultaneous stimulation, and the cutaneous stimuli are normal, the score is normal. If the patient has aphasia but does appear to attend to both sides, the score is normal. The presence of visual spatial neglect or anosagnosia may also be taken as evidence of abnormality. Since the abnormality is scored only if present, the item is never untestable.	0 = No abnormality.<br>1 = Visual, tactile, auditory, spatial, or personal inattention or extinction to bilateral simultaneous stimulation in one of the sensory modalities.<br>2 = Profound hemi-inattention or hemi-inattention to more than one modality. Does not recognize own hand or orients to only one side of space.	
**TOTAL**		**/**
Additional item, not a part of the NIH Stroke Scale score.		-
**A. Distal Motor Function:** The patient's hand is held up at the forearm by the examiner and patient is asked to extend his/her fingers as much as possible. If the patient can't or doesn't extend the fingers the examiner places the fingers in full extension and observes for any flexion movement for 5 seconds. The patient's first attempts only are graded. Repetition of the instructions or of the testing is prohibited.	0 = Normal (No flexion after 5 seconds).<br>1 = At least some extension after 5 seconds, but not fully extended. Any movement of the fingers which is not command is not scored.<br>2 = No voluntary extension after 5 seconds. Movements of the fingers at another time are not scored.<br>**a. Left Arm**<br>**b. Right Arm**	-

Modified Rankin scale (mRS)

		Score
0 =	No symptoms at all.	
1 =	No significant disability despite symptoms, able to carry out all usual duties and activities	
2 =	Slight disability, unable to carry out all previous activities but able to look after own affairs without assistance.	
3 =	Moderate disability requiring some help, but able to walk without Assistance.	
4 =	Moderate severe disability, unable to walk without assistance and unable to attend to own bodily needs without assistance.	
5 =	Severe disability, bedridden incontinent, and requiring constant nursing care and attention.	
6 =	Dead.	
		**/ 6**

Simplified mRs

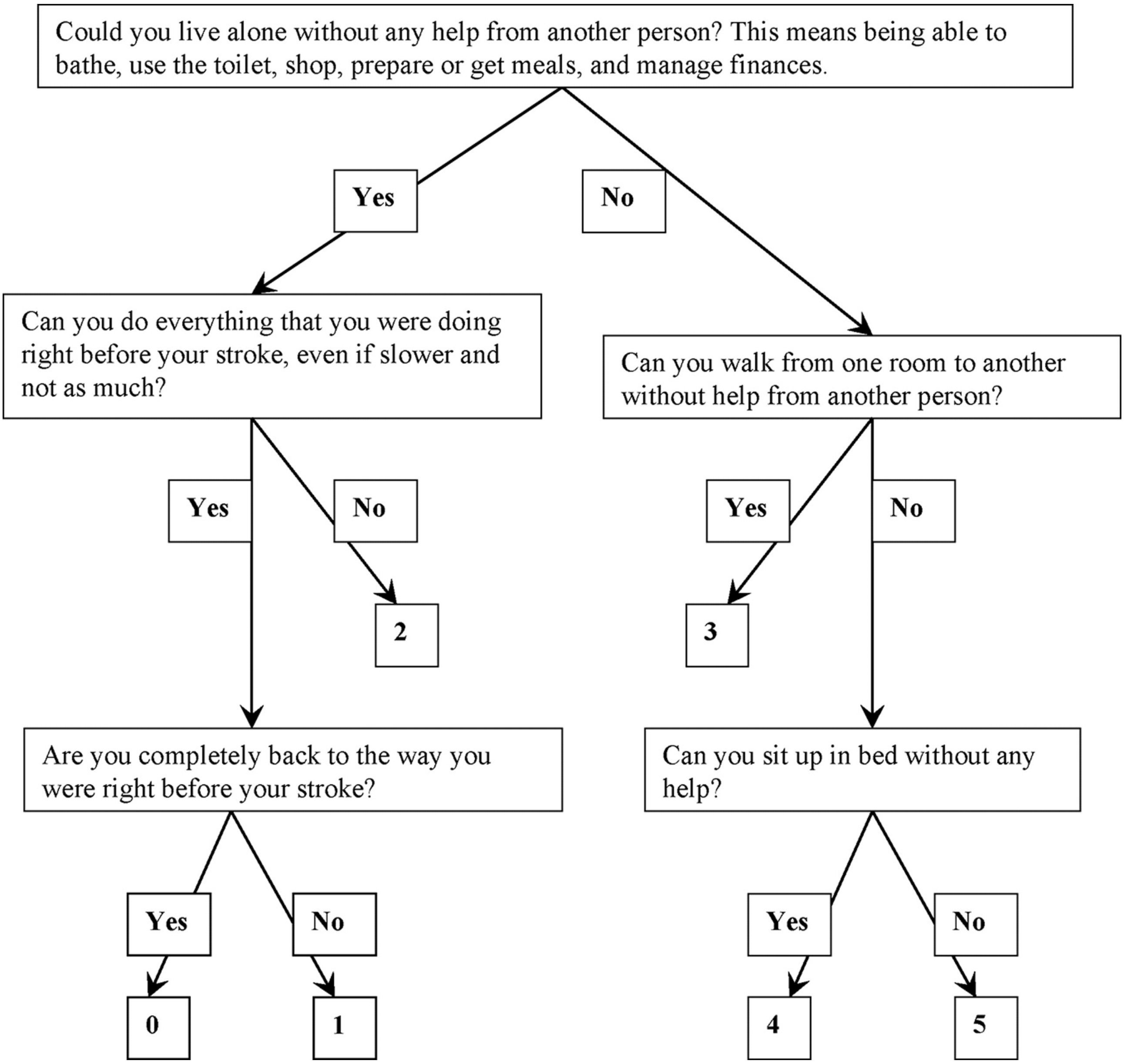

EQ- 5D

Please complete the form:

- ❖ Neatly and legibly
- ❖ Write in **black ink** and press firmly
- ❖ Each question must be completed
- ❖ Place ☑ in appropriate boxes

Please indicate which statements best describe your own health state today, by placing a tick <u>in one box in each group</u> below,

Mobility

Thinking about your health today, which of the following statements best describes your mobility?

☐ I have no problems walking about

☐ I have some problems in walking about

☐ I am confined to bed

Self-care

Thinking about your health today, which of the following statements best describes your self-care?

☐ I have no problems with self-care

☐ I have some problems washing or dressing myself

☐ I am unable to wash or dress myself

Usual activity

Thinking about your health today, which of the following statements best describes your usual activities such as work, study, housework, family or leisure activities?

☐ I have no problems with performing my usual activities

☐ I have some problems performing my usual activities

☐ I am unable to perform my usual activities.

Pain/discomfort

Thinking about your health today, which of the following statements best describes any pain or discomfort you may be experiencing?

☐ I have no pain or discomfort

☐ I have moderate pain or discomfort

☐ I have extreme pain or discomfort

Anxiety/depression

Thinking about your health today, which of the following statements best describes any anxiety and depression you may be experiencing?

- ☐ I am not anxious or depressed
- ☐ I am moderately anxious or depressed
- ☐ I am extremely anxious or depressed

APPENDIX 4 – Process evaluation interview guides and templates

Process Evaluation Interview Guide

Section One
Warm-up questions:<br>• Name<br>• What is your role at the hospital, and what are your main responsibilities?
Section Two
Perceptions about the intervention (NPT domain: coherence)<br>• How did you hear about INTERACT3? Did the PI tell you about it?<br>• What did you think was the aim of the INTERACT3 study?<br>(probes: any concerns regarding clinical aspects of the intensive blood pressure reduction in stroke care? any clinical concerns regarding the care bundle in INTERACT3?)<br>• When you compare the care bundle in INTERACT3 and routine care in your hospital, which one was easier to deliver? Please explain why.<br>• What do you think about the training provided by CRAs for this INTERACT 3 study? (probe: did we need to more training once you were in the intervention phase?)
Section Three
Implementation/delivery and actions needed (NPT domain: cognitive participation and collective action)<br>Reach<br>• How have you found patient recruitment? Have you had any difficulties in recruiting patients for INTERACT3 at your hospital? Please explain why and can you give me an example?<br>• Did you recruit all eligible patients? Why or why not?<br>Intervention Fidelity<br>• How was the cross over to the INTERACT care bundle? Did you experience any difficulties? (probes: staff understanding or concerns, patients' and carers' understanding or concerns, or other?)<br>• Did the patients receive all the components of care bundle? (probe: If not, please explain why, separate components of BP lowering, glycaemic control, treatment of pyrexia, and reversal of anticoagulation).<br>• You have since recruited 5-10 patients, has there been any changes in how you implemented the care bundle? (probe: separate components)

• How well were the care bundle targets reached in the first hour when abnormal blood pressure, blood sugar level, body temperature and INR were detected? (Probe: reasons for not achieving the targets, provide an example)<br><br><u>Implementation</u><br><br>• What difficulties have you experienced in implementing INTERACT3 so far?<br>(i.e. Such as paperwork, data entry, communication with other health professionals/departments, equipment, What were the patients' and their carers responses when you implemented the care bundle?)<br>• What could we do to support the implementation of INTERACT 3 at your hospital? (probe: BP lowering guidance, data entry)<br><br><u>Context/external factors</u><br><br>• Were there any external factors that affected the implementation of the care bundle to the patients? (e.g. availability of medication, any changes or events at your hospital during INTERACT3 that impacted on participation or recruitment?<br>• If we want to implement the care bundle as a guideline widely in hospitals across your country, what would be the main barriers ? Please explain.
Section Four
Perceived effects (NPT domain: reflexive monitoring)<br><br>• How well do you think INTERACT3 worked overall in your hospital?<br>• What is the role of PI? What was done by PI regarding the project process? (probe: What additional support could have helped from PI or from the clinical trial team?)<br>• Do you have any suggestions on how to improve recruitment and implementation of the INTERACT 3 bundle? (probe: do they anticipate any further issues)<br>• Concluding question: Do you have any other thoughts about INTERACT3 besides what we talked about above?<br>(Thank you so much for your participation in this research and for sharing your insights)

Non-participant observation documentary notes

Name of observer:

Date:

Length of time of observation:

Site No. / Location(s) of observation:

Trial implementation	Description	Comments
Enrolment procedure		
-if eligible patients being recruited		
-inform consent procedure (any difficulties in these procedures? e.g. patient or family concerns, etc.)		
Care bundle (intervention)		
-workload (number of patients, number of ward rounds, number of observations or inspections on patients)		
-interactions/ communication with patients/family surrogates		
-interactions between the clinicians and nurses<br><br>-Is care bundle implementation in accordance with the order prescribed by clinicians?		
-what intervention were delivered?<br><br>-Observe and record the procedure of blood pressure lowering/blood		

glucose control/temperature control/anticoagulant reversal<br><br>-Is the intervention implemented in accordance with the protocol?<br><br>-any barriers to implement each components of the complex interventions (etc. medication use, equipment, staffs)		
-what reactions of the patients regarding to the interventions implementation<br><br>-patient cooperation		

Focus group discussion guide

Facilitator's welcome, introduction, and instructions to participants

Welcome and thank you for taking part in this focus group. You have been asked to participate as your point of view is important to the INTERACT3 process evaluation.

Introduction: This focus group discussion is designed to assess your current thoughts and feelings about the intervention implementation in INTERACT3 project. The focus group discussion will take no more than 1.5 hour.

Anonymity: The discussion will be audio-recorded. I would like to assure you that the discussion will be anonymous. The record will be kept safely in a locked facility until they are transcribed word for word, then they will be destroyed. The transcribed notes of the focus group will contain no information that would allow individual subjects to be linked to specific statements. You should try to answer and comment as accurately and truthfully as possible. I and the other focus group participants would appreciate it if you would refrain from discussing the comments of other group members outside the focus group. If there are any questions or discussions that you do not wish to answer or participate in, you do not have to do so; however please try to answer and be as involved as possible.

Information consent: Before we start our discussion, I would like you to express your decision to participate. If you are fully understand above information and agree to participate in this interview, please repeat:

"I fully understand the background, aim, procedure, risk and benefit of participating the discussion for INTERACT3 process evaluation. I have enough time and chance to raise questions and satisfied with the answers. I agree to participate in this discussion. I acknowledge I can reject to participate at any time with no reason."

Ground rules

- The most important rule is that only one person speaks at a time. There may be a temptation to jump in when someone is talking but please wait until they have finished.
- There are no right or wrong answers
- You do not have to speak in any particular order
- When you do have something to say, please do so. There are many of you in the group and it is important that I obtain the views of each of you
- You do not have to agree with the views of other people in the group
- Does anyone have any questions? (answers).
- OK, let's begin

Warm up

Introductory question

I am just going to give you a couple of minutes to think about your experience of participating the INTERACT3 project. Is anyone happy to share his or her experience?

Guiding questions for CRAs

- What are the attitudes of you and clinicians towards the intervention from your training and communication? (What did clinician think/say/do?)
- What drove the positive/negative reaction? If negative, how could it be rectified?
- What has been challenging
  (e.g. any difficulties did you have to deliver trainings for physicians? any difficulties did you meet when monitoring the sites (include daily monitor, remote monitor and on-site monitor)?
- How many hours did you spend for INTERACT3 training (include on-site training, remote training via phones, email or messages)? How well did you think of the training you provided to the implementers (those who recruit patients and implement care bundle)?
- What are the physician's aspects / feedbacks on how and why to implement the care bundle and for whom the care bundle works for? (any concerns regarding to the interventions implementation?)
- Does the physicians involve in the program over time? If they withdrawal, what was the reason?
- Were there any barriers or facilitators did you think affect the implementation of care bundle by physicians? *Make examples and explain. (have you noted any difference across sites?)*

  How can we support you in your role better?

Guiding questions for PIs

- What are your attitudes towards the interventions in INTERACT3? (What did clinician think/say/do?)
- What do you think about the aims of having the care bundle for ICH patients?
- Do you think the intervention will improve the outcome of patients? If not, why not? (specified in each components of the intervention)
- What are the main issues around implementation of the care bundle in your ward? (implementation process, cooperation with ED, interaction between clinicians and nurses) What are the barriers to implement? What are the enablers?
- How far will existing work practices and the division of labor have to be changed or adapted to implement the intervention?
- Is any challenges you met when conducting the project in your ward (explore patient involvement, clinician teamwork and communication, data collection)? Are there any factors we can assist with?
- Did you feel comfortable to implement this intervention as routine care? Do you think this intervention can be widely used at a national level? Is the intervention consistent with the workplace and overall organization?

Concluding question

- Of all the things we've discussed today, what would you say are the most important issues you would like to express about the implementation? Are there any other things you would like to raise about the INTERACT3 that we haven't covered?

Patient Interview Guide

Purpose of the interview: *understand patient/carer perspectives of the goal-directed care bundle, especially what intervention was actually received, thoughts and cooperation of increased monitoring and concerns of participating in INTERACT3 trial.*

Questions classification:

- *Initial broad descriptive questions*
  - *Probing questions are guide only, depends on patients response on the broad questions. Don't have to ask all these questions exactly as written*

Section One
Health care experience<br>• Can you tell me about your stroke?<br>• How was the care in the hospital you received?<br>• How have you been since being discharged? Have you assessed any rehabilitation services and follow up with health providers?<br>Probing questions:<br>○ What are some of the good/bad things about your health care?<br>○ What type of support you get from family, community or social groups with looking after your health?<br>○ What kind of roles/responsibilities do you have in your family?
Section Two
Perspectives on the goal-directed care bundle<br>• What are your thoughts about your current treatment for your stroke?<br>• Can you describe the treatment you have received?<br>Probing questions:<br>○ What is the frequency of your blood pressure/blood glucose level/body temperature monitoring? Who is providing these monitor? Do you think it is okay to have this kind of monitoring?<br>○ What was good of the treatment?<br>○ What was not helpful?<br>○ How can your doctor or the treatment team improve your current care?<br>○ What has been most important in helping you getting better?
Section Three
Specific components of the care bundle if not covered<br>• Can you tell me your understanding of following care in specific:

-intensive blood pressure lowering

-glycaemic control

-fever control

-reversal of anticoagulation

Probing questions:

- Based on your understanding, do you have any concerns of intensive blood pressure reduction?
- What is your thought of regular monitoring glucose level?
- Can you describe any time or situation when you didn't want to receive the care bundle?

<u>Cooperation</u>

- What is your cooperation with health care providers of receiving the care bundle?

Probing questions:

Can you describe any time or situation when you didn't want to cooperate with doctors/nurses for your treatment?

Section Four

<u>General views about the trial</u>

- What are your thoughts about INTERACT3 trial in general?

Probing questions:

- Tell me how you think the study worked and what it was hoping to achieve?

<u>Recruitment/participating</u>

- Tell me about your thoughts of participating in this trial
- What is being informed for participating in this trial?

Probing questions:

- What were the things that made you want to participate in the study initially?
- What were the benefits/concerns to you of participating in this study?
- Did you know there will be increased monitoring in the bundle care?
- Were there any things that may have stopped you from participating initially?
- Did you feel that you could withdraw at any time?
- Did you know whom to contact if you had any concerns about the trial?
- What were your thoughts about your privacy throughout this study?

Section Five

Concluding questions<br>• Are there things which we can do better to improve the study or the running trial?<br>• Is there something else your would like to say if we have not covered in this interview?
**Thanks for your time!**

INTEnsive care bundle with blood pressure Reduction in Acute Cerebral haemorrhage Trial: a stepped-wedge cluster clinical trial

Statistical Analysis Plan

Version: 1.2 (final)

Date: 16 July 2022

Authors:

Laurent Billot, Lili Song, Xin Hu, Lu Ma, Chao You, Craig Anderson, on behalf of the INTERACT3 Investigators

Corresponding author:

Professor Laurent Billot

The George Institute for Global Health

Faculty of Medicine and Health, UNSW, Sydney, Australia

lbillot@georgeinstitute.org

1. Administrative information

1.1 Study identifiers

- Protocol Number: Version: 3.0, Date: 12 August 2019
- ClinicalTrials.gov register Identifier: NCT03209258
- Chinese Clinical Trial Registry: ChiCTR-IOC-17011787

1.2 Revision history

Version	Date	Details
0.1 (draft)	9 September 2021	First (internal) draft by Laurent Billot (LB), not circulated
0.2 (draft)	18 November 2021	First full draft. Sent to C Anderson and L Song for review. Did not include table shells.
0.3 (draft)	15 December 2021	Table shells added
0.4 (draft)	11 January 2022	Integrated further comments from Craig and Lili.
1.0 (final)	12 January 2022	Clarified the analysis of death and dependency. Sent to all authors for approval and sign-off.

1.3 Contributors to the statistical analysis plan

1.3.1 Roles and responsibilities

Name and ORCID	Affiliation	Role on study	SAP contribution
Prof Laurent Billot	The George Institute for Global Health, Faculty of Medicine, UNSW Sydney	Study statistician	Prepared initial draft and revisions
Dr Lili Song	The George Institute China;<br>The George Institute for Global Health, Faculty of Medicine, UNSW Sydney	Co-investigator<br>Project Lead	Reviewed all versions
Prof Craig Anderson	The George Institute for Global Health, Faculty of Medicine, UNSW Sydney	Principal Investigator	Reviewed all versions
Prof Chao You	Department of Neurosurgery, West China Hospital, Sichuan University, Chengdu, China	Principal Investigator	Reviewed final version
Dr Xin Hu	Department of Neurosurgery, West China Hospital, Sichuan University, Chengdu, China	Project office	Reviewed final version
Dr Lu Ma	Department of Neurosurgery, West China Hospital, Sichuan University, Chengdu, China	Project office	Reviewed final version

1.3.2 Approvals

The undersigned have reviewed this plan and approve it as final. They find it to be consistent with the requirements of the protocol as it applies to their respective areas. They also find it to be compliant with International Conference on Harmonisation (ICH-E9) principles and in particular, confirm that this analysis plan was developed in a completely blinded manner (i.e. without knowledge of the effect of the intervention[s] being assessed).

	16 July 2022		16 July 2022
Signature	Date	Signature	Date
**Professor Laurent Billot**		**Doctor Lili Song**	
	16 July 2022		16 July 2022
Signature	Date	Signature	Date
**Doctor Xin Hu**		**Doctor Lu Ma**	
	16 July 2022		16 July 2022
Signature	Date	Signature	Date
**Professor Chao Yu**		**Professor Craig Anderson**	

Contents

1 Administrative information 171

1.1 Study identifiers....171

1.2 Revision history 171

1.3 Contributors to the statistical analysis plan 171

1.3.1 Roles and responsibilities 171

1.3.2 Approvals....172

2 Introduction....176

2.1 Study synopsis 176

2.2 Study population 176

2.2.1 Site eligibility criteria 176

2.2.2 Patient eligibility criteria....176

2.3 Interventions 177

2.3.1 Intervention arm....177

2.3.2 Usual Care arm 177

2.3.3 Background standard of care 177

2.4 Outcomes 177

2.4.1 Primary outcome 178

2.4.2 Secondary outcomes 178

2.4.3 Safety outcomes 178

2.5 Randomisation and blinding....178

2.6 Statistical hypotheses....178

2.7 Sample size 178

3 Statistical analysis....179

3.1 Statistical principles....179

3.1.1 Level of statistical significance 179

3.1.2 Statistical software 180

3.2 Analysis populations....180

3.3 Impact of COVID 180

3.4 Changes from the protocol....180

3.5 Baseline analyses....181

3.5.1 Cluster characteristics 181

3.5.2 Patient characteristics 181

3.6 Assesments and interventions conducted between Day 1 and Day 7 181
3.7 Laboratory values and vital signs 181
3.8 Analysis of the primary outcome 182
3.8.1 Main analysis 182
3.8.2 Allowing time trends to vary by cluster....183
3.8.3 Adjusted analyses 183
3.8.4 Subgroup analyses 184
3.8.5 Treatment of missing data....185
3.9 Analysis of secondary outcomes 185
3.9.1 NIHSS score at 7 days 186
3.9.2 Binary analysis of mRS at 6 months....186
3.9.3 HRQoL 186
3.9.4 Duration of hospitalisation 186
3.9.5 Residence....187
3.9.6 SAEs 187
4 References....188
5 Proposed outputs 190
5.1 Tables....190
5.2 Figures 213
5.3 Listings 216
6 Proposed content and timing of primary and subsequent publications....217

Tables

Table 1. Number of subjects enrolled and with primary outcome data per group and per period 190
Table 2. Baseline characteristics.. 191
Table 3. Medical history 197
Table 4. Medications at time of admission 198
Table 5. Laboratory and vital measures recorded during the first 24 hours...................................... 200
Table 6. Antihypertensive and hypothermia treatments administered during the first 24 hours 202
Table 7. Management care administered until Day 7 203
Table 8. Details of hypertensive treatments between Days 2 and 7 205
Table 9. Treatment targets... 206
Table 10. Clinical outcomes.. 207
Table 11. SAEs.. 209
Table 12. Causes of death 210
Table 13. Protocol violations and deviations 211
Table 14. Form of assessment of 6-month outcomes... 212

Figures

Figure 1. CONSORT diagram.. 213
Figure 2. Start and stop dates of each period with number recruited per site per country.......................... 214
Figure 3. Systolic blood pressure over time 215
Figure 4. Diastolic blood pressure over time.. 215
Figure 5. Blood glucose over time 215
Figure 6. Body temperature over time... 215
Figure 7. Grotta bar charts of mRS.. 215
Figure 8: Boxplot of hematoma volume by follow-up assessment.. 215
Figure 9: Boxplot of NIHSS by follow-up assessment.. 215
Figure 10. Forest plot for subgroup analysis of mRS at 6 months 215
Figure 11. Cumulative incidence function of time to hospital discharge.. 215

Listings

Listing 1. Protocol deviations 216

2 Introduction

2.1 Study synopsis

The INTEnsive care bundle with blood pressure Reduction in Acute Cerebral Haemorrhage trial (INTERACT3) is an international, multicentre, stepped-wedge (4 phases/3 steps), cluster randomised trial undertaken to assess a multifaceted intervention (i.e. Care Bundle) in a broad range of patients with acute spontaneous 'non-traumatic' intracerebral haemorrhage (ICH). The key objective is to determine the effectiveness of a goal-directed Care Bundle of time-critical protocols of active management that involve early physiological control (intensive blood pressure [BP] lowering, glycaemic control, and early treatment of pyrexia) and the reversal of anticoagulation therapy, compared with usual standard of care, on functional outcome in patients with ICH. The full protocol has been published in 2021.[1]

Patient enrolment commenced in December 2017 and was completed in December 2021. As of 31 December 2021, there have been over 7000 patients enrolled at a total of 122 activated sites in Brazil, China, Chile, India, Mexico, Nigeria, Pakistan, Peru, Sri Lanka, and Vietnam. The activation of sites has finished. Patient follow-up will therefore be completed by June 2022, and the study closed out by November 2022. The main results are planned to be published in early 2023.

2.2 Study population

2.2.1 Site eligibility criteria

Hospital sites were eligible if they did not have established formal organisational protocols for the management of ICH, or if they used protocols different to those proposed in the study and approved a switch to the proposed interventional bundle as part of an intervention standard of care.

2.2.2 Patient eligibility criteria

- **Inclusion**
  - Age ≥18 years
  - Acute stroke syndrome presumed to be due to spontaneous ICH, defined as the sudden occurrence of bleeding into the parenchyma of the brain that may extend into the ventricles and, in rare cases, into the subarachnoid space, confirmed by clinical history and CT brain imaging. (NB Patients with ICH secondary to medical treatment [e.g. antiplatelet or antithrombotic therapy], are eligible, but ICH secondary to thrombolysis are ineligible)
  - Presentation to hospital within 6 hours of stroke onset. (NB If the precise timing of the first symptoms or signs of the qualifying event are unknown then the time of onset will be taken as the last time at which the patient was known to be well)

- **Exclusions**
  - Definite evidence that the ICH is secondary to a structural abnormality in the brain (e.g. an arteriovenous malformation [AVM], intracranial aneurysm, tumour, trauma, or previous cerebral infarction) or previous thrombolysis/thrombectomy
  - A high likelihood that the patient will not adhere to the study treatment and follow-up regimen

In each case, the decision about the patient's eligibility was based on the attending clinician investigator's interpretation of the above eligibility criteria.

2.3 Interventions

2.3.1 Intervention arm

As soon as a site was activated, the project team used a range of implementation methods to introduce a goal-directed care bundle for application to consecutive eligible patients as a policy of usual care that involved the rapid correction (<1 hour) of physiological variables as soon as the abnormalities was recognised, and for the target to be maintained in patients for 7 days or hospital discharge (or death, if sooner). These interventions are:

1. intensive BP lowering to a systolic target of <140mmHg;
2. glucose control to a target of 6.1-7.8 mmol/L and 7.8-10.0 mmol/L for patients without and with diabetes, respectively;
3. treatment of pyrexia by any means to a target body temperature ≤37.5 ℃;
4. reversal of anticoagulation to target INR <1.5 involving the use of vitamin K and prothrombin complex concentrate (PCC) or alternatively, fresh frozen plasma (FFP).

2.3.2 Usual Care arm

As soon as a site was activated, patients in the Usual Care group received standard of care. The decisions about the location of such care delivery, investigations, monitoring, and all treatments, was at the discretion of the treating clinical team.

2.3.3 Background standard of care

Data collected in all patients included their management, including the insertion of invasive monitoring devices, intravenous fluid resuscitation, BP lowering, vasoactive support, glycaemic control, mechanical ventilation, neurosurgery, and other supportive therapy.

2.4 Outcomes

The study outcomes are:

2.4.1 Primary outcome

- The primary outcome is according to scores on the modified Rankin scale (mRS) at 6 months, analysed as an ordinal outcome (categories 0 to 6).

2.4.2 Secondary outcomes

- Death or dependency according to severity of neurological impairment on the National Institute of Health Stroke Scale (NIHSS) at 7 days
- The following at 6 months:
    - Poor outcome, defined by mRS scores of 3-6;
    - Death;
    - Disability (mRS scores 3-5);
    - Health-related quality of life (HRQoL) using the EuroQoL Group 5-Dimension self-report questionnaire (EQ-5D);
    - Duration of hospitalisation;
    - Residence.

2.4.3 Safety outcomes

- Serious adverse events (SAEs) during follow-up

2.5 Randomisation and blinding

The unit of randomisation was the hospital site, randomly assigned by a statistician not otherwise involved in the study to 3 groups using a pre-specified randomisation schedule with permuted blocks. Participating sites were stratified according to country and estimated recruitment capacity (i.e. size) of the site.

2.6 Statistical hypotheses

The primary statistical hypotheses are as follows:

- **Null hypothesis**: no difference in mRS scores at 6 months between those randomised to Care Bundle and Usual Care, that is odds ratio (OR) (Care Bundle vs Usual Care) = 1
- **Alternative hypothesis (2-sided)**: OR (Intervention vs Usual Care) ≠ 1, with the expectation participants randomised to the Care Bundle would have a lower/better mRS score (OR <1).

2.7 Sample size

The study is designed with 90% power (p=0.05) to detect a 20% reduction in the odds (common OR of 0.80) of a worse outcome using an ordinal logistic regression. Assuming a distribution of mRS in the usual care arm that is similar to the one observed in the standard BP arm of the second phase of Intensive Blood Pressure Reduction in Acute Cerebral Haemorrhage Trial (INTERACT2),[2] that is 7.6%, 18.0%, 18.8%, 16.6%, 19.0%, 8.0% and 12.0% for mRS scores of 0 to 6, respectively, which corresponds to a 5.6% absolute improvement in the proportion of patients experiencing a bad outcome (mRS of 3-6), from 55.6% down to 50%. This also translates to a modest 10% relative risk reduction (relative risk of 0.90).

The study was planned to recruit 110 sites in a stepped-wedge design, consisting of 3 groups and 4 phases. Each group would, therefore, include approximately 36 sites. Assuming an intraclass correlation coefficient (ICC) of 0.044, which is similar to that found in the INTERACT2[2] and Head Position in Acute Stroke Trial (HeadPoST),[3] which included large numbers of hospitals in China, each site would be required to recruit an average of 18 patients per phase. Accounting for 5% of participants with a missing outcome, each site would need to target an average of 19 patients per phase, thus leading to a total sample size of 8,360 patients (110 sites × 4 phases × 19 patients).

The sample size calculation was performed using PASS 2019,[4] tests for two ordered categorical variables[5]and by subsequently applying the design effect corresponding to the stepped-wedge design.[6]

3 Statistical analysis

3.1 Statistical principles

3.1.1 Level of statistical significance

Two formal interim analysis were planned and undertaken using the Haybittle-Peto stopping rule (3 standard-deviations) for efficacy.[7] The first interim analysis was conducted in September 2019 with 3813 randomised participants, the second in August 2021 with 6152 randomised participants. In both occasions, the DSMB recommended continuation of the study. Given the conservative rule used and the negligible amount of type-I error rate spent at the two interim analyses, the significance threshold will remain at 5% for the final analysis.

Final analyses of the primary outcome, including sensitivity analysis, will all be conducted using a two-sided significance level of 5%.

For the seven secondary clinical outcomes, we will control the family-wise error rate by applying a sequential Holm-Sidak correction.[8] Briefly, the approach consists of ordering all p values from smallest to largest, and then comparing them to an adjusted level of significance calculated as $1-(1-0.05)^{1/C}$, where C indicates the number of comparisons that remain. In the case of seven secondary outcomes, the smallest p value would be

compared to $1-(1-0.05)^{1/7}$, the second p value to $1-(1-0.05)^{1/6}$, and so on, with the last one being compared to 1-(1-0.05) (i.e. 0.05). The sequential testing procedure stops as soon as a p value fails to reach the corrected significance level. This will apply only to the primary analysis of the secondary outcomes (i.e. not to the adjusted models or the models with varying time trends). For HRQoL, it will only apply to the analysis of the overall score.

No multiplicity correction will be applied to other statistical tests.

3.1.2 Statistical software

Analyses will be conducted primarily using SAS software (version 9.3 or above).

3.2 Analysis populations

Due to the stepped-wedge design, the group allocation for a patient is determined by the site and by the period during which they participated, regardless of treatment adherence.

The intention-to-treat analysis set will be used to assess both effectiveness and safety. The flow of patients through the study will be displayed in a CONSORT diagram (Figure 1). Details will be obtained as to the type of assessment performed (phone, face-to-face, etc.) and analyses will be undertaken according to recent reporting guidelines for stepped-wedge cluster randomised trials.[9]

Per-protocol analyses and other analyses adjusting for compliance are planned as part of secondary publications.

3.3 Impact of COVID

The COVID pandemic, which emerged during conduct of the trial in China and activation of sites elsewhere in the world, had a significant impact on recruitment in multiple countries. Thus, several analyses will be undertaken to assess the potential impact of COVID on the INTERACT3 intervention. These analyses will include baseline and time adjustments as well as subgroup analysis. We have defined two periods in relation to COVID: ‘before’ (30 January 2020) vs ‘during/after’ (from 1 February 2020) the pandemic. Deaths due to COVID will also be reported.

3.4 Changes from the protocol

In the published protocol,[1] it was stated that models will be adjusted for site size which was a stratification variable. Given that size was defined differently in China compared to other countries, and that some strata contain small numbers of sites, we have subsequently decided not to include size site as a covariate.

Although we indicated that no adjustment for multiplicity would be performed, we now feel that adjusting the family-wise error rate across the seven secondary outcomes would be more appropriate.

Both these changes are made in a blinded manner and independent of any trial result.

3.5 Baseline analyses

3.5.1 Cluster characteristics

Description of the cluster characteristics (e.g. location, size) will be presented by treatment group. Discrete variables will be summarised by frequencies and percentages. Percentages will be calculated according to the number of clusters with available data. Continuous variables will be summarised by using mean and standard deviation (SD), and median and interquartile range (Q1-Q3).

3.5.2 Patient characteristics

Description of the baseline characteristics will be presented by treatment group and by study period. Discrete variables will be summarised by frequencies and percentages. Percentages will be calculated according to the number of patients in whom data are available. Continuous variables will be summarised by using mean and SD, and median and interquartile range (Q1-Q3). No adjustment for clustering will be applied when summarising baseline characteristics. To assess whether the profile of patients enrolled changed over time due to COVID, we will also describe baseline characteristics according to COVID period (see Section 3.3). Baseline measures will include all socio-demographic, clinical and medical information collected at baseline.

3.6 Assessments and interventions conducted between Day 1 and Day 7

All assessments performed and interventions received between Day 1 and Day 7 will be described by intervention group. No formal statistical tests are planned for these variables.

Protocol deviations will be categorised and reported as the number and proportion of subjects experiencing a deviation. A listing of all protocol deviations will be provided.

3.7 Laboratory values and vital signs

Laboratory values and vital signs collected repeatedly over the first 24 hours and 7 days will be described using longitudinal mean plots and analysed using repeated-measure linear mixed models. This will apply to systolic BP, diastolic BP, blood glucose, and body temperature. The intervention effect will be estimated using a similar approach as the primary analysis model described in Section 3.8.1, but with the following differences:

(a) given the continuous nature of the outcome, a normal distribution and identity link will be used;

(b) the model will include all available post-baseline measurements per subject with within-subject correlations modelled using a repeated effect with a compound-symmetry covariance structure, and;

(c) the baseline value of the outcome will be added as a fixed covariate.

The effect of the intervention at 1 hour and 24 hours will be estimated from the model as the adjusted mean difference and 95% CI.

The proportion of patients reaching each treatment target (e.g. systolic BP <140 mmHg) will be reported by treatment group. For these analyses, the denominators will consist in patients who were eligible for the corresponding target. For example, only patients with a systolic BP $\geqslant$140mmHg at baseline will be included in the analysis of systolic BP target. For those reaching target, we will also describe the time take to reach the corresponding target. No formal statistical tests will be performed on these metrics.

These analyses are only applicable to the subset of subjects meeting the relevant eligibility criteria at baseline (e.g. glucose control was only applicable to subjects outside of the recommended glucose targets at baseline) and will include all data available.

3.8 Analysis of the primary outcome

The primary outcome of the mRS at 6 months will be analysed as an ordinal variable with 7 levels. The primary intervention effect will be estimated as the OR of a higher mRS between the intervention arm and the usual care arm obtained from an ordinal logistic model (defined below).

3.8.1 Main analysis

The primary analysis will be conducted by extending the approach developed by Hussey and Hughes for the analysis of stepped-wedge trials[10] to an ordinal outcome. We will be using an ordinal logistic regression with a random effect for cluster (hospital site), a fixed effect indicating the group assignment of each cluster at each step, and a fixed categorical effect of time (each step). The corresponding cumulative logit model can be written as follows:

$$\text{logit}(P\{Y_{ijk} \leqslant m\}) = \mu_m + \theta X_{ij} + \beta_j + c_i + e_{ijk} \qquad \text{(model 1)}$$

where:

Y_{ijk} denotes the outcome of subject *k* in period *j* within cluster *i*

μ_m is the intercept corresponding to a mRS score of *m* (*m*=0, 1, 2, 3, 4, 5, 6)

X_{ij} is a binary variable indicating group allocation during period j for cluster i with X_{ij} = 1 for intervention and X_{ij} = 0 for control

θ is the parameter of interest estimating the fixed effect of the intervention and where $\exp(\theta)$ is the OR

β_j is a fixed categorical period effect

c_i is a random cluster effect with $c_i \sim N(0,\sigma_c^2)$

e_{ijk} are individual-level errors following a multinomial distribution

The effect of the intervention will be presented as the OR of a worse outcome and its 95% confidence interval (CI) using the usual care arm as the reference (i.e., where an OR greater than unity corresponds to an increase in mRS in the intervention arm compared to the usual care arm).

Randomisation was stratified by country and size of site, defined as the expected number of recruited patients. Given the large number of strata, and that the primary analysis is already adjusted by site, the main analysis model will not further adjust for country or size of site. Further adjustments will, however, be performed as sensitivity analyses (see Section 3.8.3).

We will test the proportional-odds assumption in Model 1 using a score test. In case of violation, we will proceed with the analysis and interpret the intervention odds ratio as an average effect across all mRS levels with the understanding that it may not be constant across all levels. This will be complemented by a graphical assessment of shifts across categories using bar plots as well as a binary analysis (see Section 3.9.2). As a sensitivity analysis, we will apply partial proportional odds logistic regression, relaxing the proportional odds assumption for covariates where it does not hold.

3.8.2 Allowing time trends to vary by cluster

The base model developed by Hussey and Hughes makes the implicit assumption that the effect of time is common across all clusters. Given the large number of clusters and that sites entered the study at different times, an additional model will be run to allow the effect of time to vary randomly across sites, as described by Hemming et al.[11] This will be done by adding v_{ij}, a random interaction between period and cluster with $v_{ij} \sim N(0,\sigma_v^2)$ to Model 1, which becomes:

$$\text{logit}(P\{Y_{ijk} \leqslant m\}) = \mu_m + \theta X_{ij} + \beta_j + c_i + v_{ij} + e_{ijk} \qquad \text{(model 2)}$$

The addition of a random period-by-cluster interaction will allow the intracluster correlation to vary depending on whether subjects from the same cluster are from the same period or from a different period. This model will also include adjustment by country and size of site.

3.8.3 Adjusted analyses

The two models described in Sections 3.8.1 and 3.8.2 will be re-run after adding the following individual baseline covariates as fixed effects:

- Country (grouped as China vs. India/Pakistan/Sri Lanka/Vietnam vs. Brazil/Peru/Chile/Mexico/Nigeria)
- mRS before stroke (categorical variable)
- Age (continuous)
- Sex (male vs female)
- Baseline NIHSS score (continuous)

Should the examination of baseline characteristics by COVID period reveal important changes in patient characteristics over time (see Section 3.5.2), additional baseline variables will be added as covariates. This decision will be made whilst blind to the intervention effect and, if implemented, would lead to two additional adjusted models (i.e. adding to the previous two models which include country, mRS, age, sex and NIHSS score).

3.8.4 Subgroup analyses

The following subgroup analyses are planned for the primary outcome, regardless of the statistical significance for the primary analysis:

- Age: <60, 60-<70, 70-<80, 80+
- Sex: male vs. female
- Country: China vs. India/Pakistan/Sri Lanka/Vietnam vs. Brazil/Peru/Chile/Mexico/Nigeria
- NIHSS: <15 vs. ≥15
- Baseline haematoma volume: <15 ml vs. 15-<30ml vs. ≥30ml
- Baseline haematoma location: cortical vs. deep vs. brainstem/cerebellar/primary ventricular. In case of multiple locations, the following hierarchy will be used to allocate a 'primary' location: 1) Deep, 2) Cortical, 3) Other.

Each subgroup analysis will be performed by adding the subgroup variable as well as its interaction with the intervention as fixed effects to the logistic regression model used for the primary analysis (see Section 3.8.1). Within each subgroup, summary measures will include raw counts and percentages within each treatment arm, as well as the OR for treatment effect with 95% CI. The results will be displayed on a forest plot including the P value for heterogeneity corresponding to the interaction term between the intervention and the subgroup variable.

Other subgroup analyses for exploratory purposes include:

- Country: by individual countries and grouped by economic development (low-middle vs. high-middle [including high income country, Chile])
- Site: by size (small [<500 beds] vs. medium [500-999] beds vs. large [1000+] beds) and type (tertiary vs. secondary)
- Time from onset: by hour

3.8.5 Treatment of missing data

The proportion of data missing for the primary outcome (mRS at 6 months) will be described overall, by study period and by COVID period (see Section 3.3), while blinded to the intervention. Reasons for missing data will be described overall and by intervention arm. In case of non-negligible amounts of missing data (>10%) or changes in missing data patterns overtime, we will use controlled multiple imputations to assess under what conditions the results change, and how plausible these conditions are, using the approach described by Cro et al.[12]

We will first run an imputation model under the missing at random (MAR) assumption. This MAR imputation model will use fully conditional specification (FCS)[13] and will include the following variables: mRS at 6 months, the NIHSS and mRS scores at 7 days (or hospital discharge, if sooner), a variable indicating the cluster (hospital), a variable indicating the stepped-wedge period, a variable indicating the intervention, and all key socio-demographic, clinical, and medical baseline variables. The mRS at 6 months and NIHSS at 7 days will be imputed using an ordinal logistic model. Other variables will be imputed using either linear regression (for continuous/ordinal variables) or a discriminant function method (for nominal variables). One hundred sets of imputed data will be created and analysed using the model described in Section 3.8.1. Estimates of the treatment effect (e^{θ} in Model 1) and its standard errors will be combined to obtain a pooled common OR and 95% CI.

Using the same 100 sets of imputed data as our base, we will then assume different mRS levels for subjects who had missing mRS data at 6 months and had their mRS value imputed. We will assume that those with a missing mRS were more likely to have a poorer outcome than those with a non-missing mRS; we will therefore add 1 to their imputed mRS score (with a maximum score of 6). We will then analyse the 100 modified-imputed dataset and combine the results using the same strategy as for the base set of imputed data. As an additional sensitivity analysis, we will impute all missing mRS score at 6 months with a score of 6, thus assuming all subjects with missing data have died. While this assumption is unlikely to hold in most cases, it is plausible that some subjects may have become uncontactable due to death after hospital discharge.

3.9 Analysis of secondary outcomes

3.9.1 NIHSS score at 7 days

The NIHSS score at 7 days will be categorized into 7 levels (<5, 5-9, 10-14, 15-19, 20-24, ⩾25, and death), and analysed using the same method as the mRS score described in Section 3.8.1. As a sensitivity analysis, the NIHSS score will also be analysed as a continuous variable using an unadjusted hierarchal linear regression model similar to Model 1, but assuming a normal distribution and an identity link function. The effect of the intervention will be presented as the mean difference and associated 95% CI. We will also apply the model with varying time trends (Section 3.8.2) as well as the covariate adjustments described in Section 3.8.3; however, no subgroup or imputed analysis will be performed on this outcome.

3.9.2 Binary analysis of mRS at 6 months

A binary analysis of the mRS at 6 months will be performed by dichotomizing the mRS as either 'poor' (scores 3-6) or 'favourable' (scores 0-2) outcomes. This analysis will be conducted using an unadjusted hierarchical logistic regression similar to Model 1 (see Section 3.8.1), but this time with a binomial outcome and a logit link function. The effect of the intervention will be presented as the OR of a poor outcome with associated 95% CI. We will also apply the model with varying time trends (Section 3.8.2) as well as the covariate adjustments described in Section 3.8.3; however, no subgroup or imputed analysis will be performed on this outcome. A similar analysis will be performed on mortality alone (mRS of 6) and on dependency alone (mRS 3-5). For dependency alone, the analysis will be restricted to subjects who are alive at 6 months (mRS 0-5).

3.9.3 HRQoL

Each of the five EQ-5D dimensions will be analysed via ordinal logistic regression using the same model as for the primary analysis of mRS at 6 months (Section 3.8.1). The visual analogous scale (score of 0 to 100) will be analysed using the same approach but with linear regression (i.e. assuming a normal distribution and an identity link function). We only plan to apply the primary model to these HRQoL outcomes (i.e. neither the model with varying time trends, nor the adjusted or subgroup analyses). Further analyses of EQ-5D will be undertaken as part of the economic evaluations, which is outside the scope of this SAP.

3.9.4 Duration of hospitalisation

Duration of hospitalisation will be analysed as time to discharge censored at 6 months or when the subject was last known to be alive and in hospital, whichever is earlier. It will be summarized using cumulative incidence functions treating mortality as a competing risk. Medians and quartiles of time to discharge will be obtained from the cumulative incidence functions. The effect of the intervention will be estimated as the hazard ratio (Intervention divided by control) and its 95%CI obtained from a Cox model of the cause-specific hazard which estimates the risk of discharge in subjects who are still alive and have not yet been discharged.[14] Similar to the primary analysis model (Section 3.8.1), the Cox model will include a fixed categorical period

effect as well as a binary variable indicating the group allocation during each period in each cluster. To model potential within-cluster correlations, we will use a shared-parameter frailty Cox model with a random site effect.[15] We only plan to apply the primary model to this outcome (i.e. neither the model with varying time trends, nor the adjusted or subgroup analyses).

3.9.5 Residence

The patient's place of residence at 6 months will be described using all possible categories then analysed as a binary outcome using the same approach as for the binary analysis of the mRS at 6 months (Section 3.9.2). For the purpose of the model, residence will be defined as follows:

(1) home: own home (independent or with assistance) or family member's home

(2) institution: hospital, care facility or other

We only plan to apply the primary model to this outcome (i.e. neither the model with varying time trends, nor the adjusted or subgroup analyses).

3.9.6 SAEs

SAEs will be summarised as the number of events as well as the number and proportion of patients experiencing at least one event. This will be done overall and by category of event according to Medical Dictionary for Regulatory Activities (MeDRA) system organ classes and preferred terms. The overall proportion of patients with SAEs in the intervention and control arms will be compared using logistic regression as in the binary analysis of mRS (see Section 3.9.2). Primary and underlying causes of deaths will be summarised by treatment arm with no formal test.

4 References

1. Song L, Hu X, Ma L, Chen X, Ouyang M, Billot L, et al. INTEnsive care bundle with blood pressure reduction in acute cerebral hemorrhage trial (INTERACT3): study protocol for a pragmatic stepped-wedge cluster-randomized controlled trial. *Trials* 2021;22:943.

2. Anderson CS, Heeley E, Huang Y, Wang J, Stapf C, Delcourt C, et al. Rapid blood-pressure lowering in patients with acute intracerebral hemorrhage. *N Engl J Med* 2013;368:2355-65.

3. Anderson CS, Arima H, Lavados P, Billot L, Hackett ML, Olavarria VV, et al. Cluster-randomized, crossover trial of head positioning in acute stroke. *New Engl J Med* 2017;376:2437-47.

4. PASS 2019. Power analysis and sample size software. NCSS, LLC. Kaysville, Utah, USA, ncss.com/software/pass.

5. Whitehead J. Sample size calculations for ordered categorical data. *Stat Med* 1993;12:2257-71.

6. Woertman W, de Hoop E, Moerbeek M, Zuidema SU, Gerritsen DL, Teerenstra S. Stepped wedge designs could reduce the required sample size in cluster randomized trials. *J Clin Epidemiol* 2013;66:752-8

7. Haybittle JL. Repeated assessment of results in clinical trials of cancer treatment. *Br J Radiol* 1971;44:793-97.

8. Abdi H. Holm's Sequential Bonferroni Procedure. In Neil Salkind (Ed.), Encyclopedia of Research Design. Thousand Oaks, CA: Sage. 2010.

9. Hemming K, Taljaard M, McKenzie JE, Hooper R, Copas A, Thompson JA, et al. Reporting of stepped wedge cluster randomised trials: extension of the CONSORT 2010 statement with explanation and elaboration. *BMJ* 2018;363:k1614.

10. Hussey MA, Hughes JP. Design and analysis of stepped wedge cluster randomized trials. *Contemp Clin Trials* 2007;28:182–91.

11. Hemming, K., Taljaard, M. & Forbes, A. Analysis of cluster randomised stepped wedge trials with repeated cross-sectional samples. *Trials* 2017;18:101

12. Cro S, Morris TP, Kenward MG, Carpenter JR. Sensitivity analysis for clinical trials with missing continuous outcome data using controlled multiple imputation: a practical guide. *Stat Med* 2020;39:2815-42.

13. van Buuren, S. Multiple imputation of discrete and continuous data by fully conditional specification. *Stat Meth Med Res* 2007;16:219-42.

14. Austin PC, Lee DS, Fine JP. Introduction to the analysis of survival data in the presence of competing risks. *Circulation* 2016;133:601-9

15. Ripatti S, Palmgren J. Estimation of multivariate frailty models using penalized partial likelihood. *Biometrics* 2002;56:1016-22

5 Proposed outputs

5.1 Tables

Table 3. Number of subjects enrolled and with primary outcome data per group and per period

Group	Period 1	Period 2	Period 3	Period 4	Total
**1 (xx sites)**	xxx / xxx (xx%)	xxx / xxx (xx%)	xxx / xxx (xx%)	xxx / xxx (xx%)	xxx / xxx (xx%)
**2 (xx sites)**	xxx / xxx (xx%)	xxx / xxx (xx%)	xxx / xxx (xx%)	xxx / xxx (xx%)	xxx / xxx (xx%)
**3 (xx sites)**	xxx / xxx (xx%)	xxx / xxx (xx%)	xxx / xxx (xx%)	xxx / xxx (xx%)	xxx / xxx (xx%)
**Total (xxx sites)**	xxx / xxx (xx%)	xxx / xxx (xx%)	xxx / xxx (xx%)	xxx / xxx (xx%)	xxx / xxx (xx%)

Notes: the denominator represents the number of subjects enrolled during the period. The numerator represents the number of subjects with a primary endpoint measure (mRS at 6 months). Cells shaded in grey represent usual care periods. Cells shaded in orange represent care bundle periods.

Table 4. Baseline characteristics

	Care Bundle (N =)	Usual Care (N =)	Total (N =)
**Age (years)**	xxx	xxx	xxx
Mean (SD)	xxx.x (xxx.x)	xxx.x (xxx.x)	xxx.x (xxx.x)
Median (Q1; Q3)	xxx (xxx; xxx)	xxx (xxx; xxx)	xxx (xxx; xxx)
min max	xxx to xxx	xxx to xxx	xxx to xxx
**Sex**	xxx	xxx	xxx
Male	xxx xx.x%	xxx xx.x%	xxx xx.x%
Female	xxx xx.x%	xxx xx.x%	xxx xx.x%
**Ethnicity**	xxx	xxx	xxx
Han Chinese	xxx xx.x%	xxx xx.x%	xxx xx.x%
Non-Han Chinese	xxx xx.x%	xxx xx.x%	xxx xx.x%
Caucasian/European	xxx xx.x%	xxx xx.x%	xxx xx.x%
Latin American	xxx xx.x%	xxx xx.x%	xxx xx.x%
Mixed	xxx xx.x%	xxx xx.x%	xxx xx.x%
Other Asian	xxx xx.x%	xxx xx.x%	xxx xx.x%
Indian Subcontinent	xxx xx.x%	xxx xx.x%	xxx xx.x%
Other	xxx xx.x%	xxx xx.x%	xxx xx.x%
**Place of residency**	xxx	xxx	xxx
Own home	xxx xx.x%	xxx xx.x%	xxx xx.x%
Family member's home	xxx xx.x%	xxx xx.x%	xxx xx.x%
Care facility (eg nursing home, hostel, shelter, community based care)	xxx xx.x%	xxx xx.x%	xxx xx.x%
Other	xxx xx.x%	xxx xx.x%	xxx xx.x%
**Main occupation pre-stroke**	xxx	xxx	xxx
Professional, executive	xxx xx.x%	xxx xx.x%	xxx xx.x%
Business	xxx xx.x%	xxx xx.x%	xxx xx.x%
Sales and service	xxx xx.x%	xxx xx.x%	xxx xx.x%
Driver	xxx xx.x%	xxx xx.x%	xxx xx.x%
Farmer, labourer	xxx xx.x%	xxx xx.x%	xxx xx.x%
Home duties	xxx xx.x%	xxx xx.x%	xxx xx.x%
Other	xxx xx.x%	xxx xx.x%	xxx xx.x%
**Highest education degree completed**	xxx	xxx	xxx
Primary school	xxx xx.x%	xxx xx.x%	xxx xx.x%

	Care Bundle (N =)	Usual Care (N =)	Total (N =)
Junior school	xxx xx.x%	xxx xx.x%	xxx xx.x%
High school	xxx xx.x%	xxx xx.x%	xxx xx.x%
Undergraduate (bachelor degree)	xxx xx.x%	xxx xx.x%	xxx xx.x%
Postgraduate (master degree or higher)	xxx xx.x%	xxx xx.x%	xxx xx.x%
Never had education	xxx xx.x%	xxx xx.x%	xxx xx.x%
**Height (cm)**	xxx	xxx	xxx
Mean (SD)	xxx.x (xxx.x)	xxx.x (xxx.x)	xxx.x (xxx.x)
Median (Q1; Q3)	xxx (xxx; xxx)	xxx (xxx; xxx)	xxx (xxx; xxx)
min max	xxx to xxx	xxx to xxx	xxx to xxx
**Weight (kg)**	xxx	xxx	xxx
Mean (SD)	xxx.x (xxx.x)	xxx.x (xxx.x)	xxx.x (xxx.x)
Median (Q1; Q3)	xxx (xxx; xxx)	xxx (xxx; xxx)	xxx (xxx; xxx)
min max	xxx to xxx	xxx to xxx	xxx to xxx
**Body Mass Index**	xxx	xxx	xxx
Mean (SD)	xxx.x (xxx.x)	xxx.x (xxx.x)	xxx.x (xxx.x)
Median (Q1; Q3)	xxx (xxx; xxx)	xxx (xxx; xxx)	xxx (xxx; xxx)
min max	xxx to xxx	xxx to xxx	xxx to xxx
**Systolic Blood Pressure (mmHg)**	xxx	xxx	xxx
Mean (SD)	xxx.x (xxx.x)	xxx.x (xxx.x)	xxx.x (xxx.x)
Median (Q1; Q3)	xxx (xxx; xxx)	xxx (xxx; xxx)	xxx (xxx; xxx)
min max	xxx to xxx	xxx to xxx	xxx to xxx
**Diastolic Blood Pressure (mmHg)**	xxx	xxx	xxx
Mean (SD)	xxx.x (xxx.x)	xxx.x (xxx.x)	xxx.x (xxx.x)
Median (Q1; Q3)	xxx (xxx; xxx)	xxx (xxx; xxx)	xxx (xxx; xxx)
min max	xxx to xxx	xxx to xxx	xxx to xxx
**Heart Rate (bpm)**	xxx	xxx	xxx
Mean (SD)	xxx.x (xxx.x)	xxx.x (xxx.x)	xxx.x (xxx.x)
Median (Q1; Q3)	xxx (xxx; xxx)	xxx (xxx; xxx)	xxx (xxx; xxx)
min max	xxx to xxx	xxx to xxx	xxx to xxx
**Blood Glucose (mmol/L)**	xxx	xxx	xxx
Mean (SD)	xxx.x (xxx.x)	xxx.x (xxx.x)	xxx.x (xxx.x)

	Care Bundle (N =)	Usual Care (N =)	Total (N =)
Median (Q1; Q3)	xxx (xxx; xxx)	xxx (xxx; xxx)	xxx (xxx; xxx)
min max	xxx to xxx	xxx to xxx	xxx to xxx
**Body Temperature (°C)**	xxx	xxx	xxx
Mean (SD)	xxx.x (xxx.x)	xxx.x (xxx.x)	xxx.x (xxx.x)
Median (Q1; Q3)	xxx (xxx; xxx)	xxx (xxx; xxx)	xxx (xxx; xxx)
min max	xxx to xxx	xxx to xxx	xxx to xxx
**Pre-stroke modified Rankin scale**	xxx	xxx	xxx
0 - no symptoms	xxx xx.x%	xxx xx.x%	xxx xx.x%
1 - no significant disability (with symptoms)	xxx xx.x%	xxx xx.x%	xxx xx.x%
2 - slight disability (but independent in daily activities)	xxx xx.x%	xxx xx.x%	xxx xx.x%
3 - moderate disability (requiring some help from another person for daily activities)	xxx xx.x%	xxx xx.x%	xxx xx.x%
4 - moderate severe disability (requiring regular help from another person)	xxx xx.x%	xxx xx.x%	xxx xx.x%
5 - severe disability (bed bound, totally dependent)	xxx xx.x%	xxx xx.x%	xxx xx.x%
**NIHSS Score**	xxx	xxx	xxx
Mean (SD)	xxx.x (xxx.x)	xxx.x (xxx.x)	xxx.x (xxx.x)
Median (Q1; Q3)	xxx (xxx; xxx)	xxx (xxx; xxx)	xxx (xxx; xxx)
min max	xxx to xxx	xxx to xxx	xxx to xxx
**GCS Score**	xxx	xxx	xxx
Mean (SD)	xxx.x (xxx.x)	xxx.x (xxx.x)	xxx.x (xxx.x)
Median (Q1; Q3)	xxx (xxx; xxx)	xxx (xxx; xxx)	xxx (xxx; xxx)
min max	xxx to xxx	xxx to xxx	xxx to xxx
Eyes Open	xxx	xxx	xxx
1=none	xxx xx.x%	xxx xx.x%	xxx xx.x%
2=To pain	xxx xx.x%	xxx xx.x%	xxx xx.x%
3=To speech	xxx xx.x%	xxx xx.x%	xxx xx.x%
4=Spontaneously	xxx xx.x%	xxx xx.x%	xxx xx.x%
Verbal Response	xxx	xxx	xxx
1=none	xxx xx.x%	xxx xx.x%	xxx xx.x%
2=Incomprehensible sounds	xxx xx.x%	xxx xx.x%	xxx xx.x%
3=Inappropriate words	xxx xx.x%	xxx xx.x%	xxx xx.x%
4=Confused	xxx xx.x%	xxx xx.x%	xxx xx.x%

	Care Bundle (N =)	Usual Care (N =)	Total (N =)
5=Orientated	xxx xx.x%	xxx xx.x%	xxx xx.x%
Motor Response	xxx	xxx	xxx
1=none	xxx xx.x%	xxx xx.x%	xxx xx.x%
2=Extension to pain	xxx xx.x%	xxx xx.x%	xxx xx.x%
3=Flexion to pain	xxx xx.x%	xxx xx.x%	xxx xx.x%
4=Withdraws	xxx xx.x%	xxx xx.x%	xxx xx.x%
5=Localises to pain	xxx xx.x%	xxx xx.x%	xxx xx.x%
6=Obey commands	xxx xx.x%	xxx xx.x%	xxx xx.x%
**Used anticoagulation**	xxx	xxx	xxx
Yes	xxx xx.x%	xxx xx.x%	xxx xx.x%
No	xxx xx.x%	xxx xx.x%	xxx xx.x%
**INR on arrival**	xxx	xxx	xxx
Mean (SD)	xxx.x (xxx.x)	xxx.x (xxx.x)	xxx.x (xxx.x)
Median (Q1; Q3)	xxx (xxx; xxx)	xxx (xxx; xxx)	xxx (xxx; xxx)
min max	xxx to xxx	xxx to xxx	xxx to xxx
**Haematoma present**	xxx	xxx	xxx
Yes	xxx xx.x%	xxx xx.x%	xxx xx.x%
No	xxx xx.x%	xxx xx.x%	xxx xx.x%
**Initial volume of haematoma (mL)**	xxx	xxx	xxx
Mean (SD)	xxx.x (xxx.x)	xxx.x (xxx.x)	xxx.x (xxx.x)
Median (Q1; Q3)	xxx (xxx; xxx)	xxx (xxx; xxx)	xxx (xxx; xxx)
min max	xxx to xxx	xxx to xxx	xxx to xxx
**Side of haematoma**	xxx	xxx	xxx
Left	xxx xx.x%	xxx xx.x%	xxx xx.x%
Right	xxx xx.x%	xxx xx.x%	xxx xx.x%
Midline	xxx xx.x%	xxx xx.x%	xxx xx.x%
**Site of haematoma**	xxx	xxx	xxx
Cortical	xxx xx.x%	xxx xx.x%	xxx xx.x%
Deep	xxx xx.x%	xxx xx.x%	xxx xx.x%
Cerebellum	xxx xx.x%	xxx xx.x%	xxx xx.x%
Brainstem	xxx xx.x%	xxx xx.x%	xxx xx.x%

	Care Bundle (N =)	Usual Care (N =)	Total (N =)
**Intraventricular blood present**	xxx	xxx	xxx
Yes	xxx xx.x%	xxx xx.x%	xxx xx.x%
No	xxx xx.x%	xxx xx.x%	xxx xx.x%
**Volume of intraventricular blood (mL)**	xxx	xxx	xxx
Mean (SD)	xxx.x (xxx.x)	xxx.x (xxx.x)	xxx.x (xxx.x)
Median (Q1; Q3)	xxx (xxx; xxx)	xxx (xxx; xxx)	xxx (xxx; xxx)
min max	xxx to xxx	xxx to xxx	xxx to xxx
**Admission service**	xxx	xxx	xxx
Neurosurgery	xxx xx.x%	xxx xx.x%	xxx xx.x%
Neurology	xxx xx.x%	xxx xx.x%	xxx xx.x%
Intensive care	xxx xx.x%	xxx xx.x%	xxx xx.x%
Emergency department	xxx xx.x%	xxx xx.x%	xxx xx.x%
Other	xxx xx.x%	xxx xx.x%	xxx xx.x%
**Clinician's opinion of patient surviving next 48 hours**	xxx	xxx	xxx
Yes	xxx xx.x%	xxx xx.x%	xxx xx.x%
No	xxx xx.x%	xxx xx.x%	xxx xx.x%
**Level of certainty of prognosis**	xxx	xxx	xxx
Definite no	xxx xx.x%	xxx xx.x%	xxx xx.x%
Possible no	xxx xx.x%	xxx xx.x%	xxx xx.x%
Uncertain	xxx xx.x%	xxx xx.x%	xxx xx.x%
Possible yes	xxx xx.x%	xxx xx.x%	xxx xx.x%
Definite yes	xxx xx.x%	xxx xx.x%	xxx xx.x%
**Years of practice of doctor giving prognosis**	xxx	xxx	xxx
Mean (SD)	xxx.x (xxx.x)	xxx.x (xxx.x)	xxx.x (xxx.x)
Median (Q1; Q3)	xxx (xxx; xxx)	xxx (xxx; xxx)	xxx (xxx; xxx)
min max	xxx to xxx	xxx to xxx	xxx to xxx
**Final diagnosis**			
Hypertension-related ICH	xxx xx.x%	xxx xx.x%	xxx xx.x%
Presumed amyloid angiopathy	xxx xx.x%	xxx xx.x%	xxx xx.x%
AVM	xxx xx.x%	xxx xx.x%	xxx xx.x%

	Care Bundle (N =)	Usual Care (N =)	Total (N =)
Aneurysm	xxx xx.x%	xxx xx.x%	xxx xx.x%
Other vascular abnormality	xxx xx.x%	xxx xx.x%	xxx xx.x%
Tumour	xxx xx.x%	xxx xx.x%	xxx xx.x%
Systemic/other disease	xxx xx.x%	xxx xx.x%	xxx xx.x%
Anticoagulation	xxx xx.x%	xxx xx.x%	xxx xx.x%
Other definite pathology	xxx xx.x%	xxx xx.x%	xxx xx.x%
Uncertain aetiology	xxx xx.x%	xxx xx.x%	xxx xx.x%

Table 5. Medical history

Variable	Care Bundle (N =)	Usual Care (N =)	Total (N =)
Intracerebral haemorrhage	xxx/xxx xx.x%	xxx/xxx xx.x%	xxx/xxx xx.x%
Ischaemic stroke	xxx/xxx xx.x%	xxx/xxx xx.x%	xxx/xxx xx.x%
Stroke of unknown type	xxx/xxx xx.x%	xxx/xxx xx.x%	xxx/xxx xx.x%
Acute coronary syndrome (STEMI/NSTEMI)	xxx/xxx xx.x%	xxx/xxx xx.x%	xxx/xxx xx.x%
Atrial fibrillation	xxx/xxx xx.x%	xxx/xxx xx.x%	xxx/xxx xx.x%
Heart failure	xxx/xxx xx.x%	xxx/xxx xx.x%	xxx/xxx xx.x%
Other heart disease	xxx/xxx xx.x%	xxx/xxx xx.x%	xxx/xxx xx.x%
Hypertension	xxx/xxx xx.x%	xxx/xxx xx.x%	xxx/xxx xx.x%
Hypertension on treatment	xxx/xxx xx.x%	xxx/xxx xx.x%	xxx/xxx xx.x%
Diabetes mellitus	xxx/xxx xx.x%	xxx/xxx xx.x%	xxx/xxx xx.x%
Hypercholesterolaemia	xxx/xxx xx.x%	xxx/xxx xx.x%	xxx/xxx xx.x%
Known coagulation or other haematological disorder	xxx/xxx xx.x%	xxx/xxx xx.x%	xxx/xxx xx.x%
Epilepsy	xxx/xxx xx.x%	xxx/xxx xx.x%	xxx/xxx xx.x%
Liver disease	xxx/xxx xx.x%	xxx/xxx xx.x%	xxx/xxx xx.x%
Current smoker	xxx/xxx xx.x%	xxx/xxx xx.x%	xxx/xxx xx.x%
Current alcohol use	xxx/xxx xx.x%	xxx/xxx xx.x%	xxx/xxx xx.x%
Other major health condition(s) that limits daily activity	xxx/xxx xx.x%	xxx/xxx xx.x%	xxx/xxx xx.x%

Table 6. Medications at time of admission

Variable	Care Bundle (N =)	Usual Care (N =)	Total (N =)
**Anticoagulant agent used**	xxx/xxx xx.x%	xxx/xxx xx.x%	xxx/xxx xx.x%
**Type of anticoagulant agent**	xxxx	xxxx	xxxx
Vitamin K antagonist (warfarin, neo-sintrom)	xxx xx.x%	xxx xx.x%	xxx xx.x%
New oral anticoagulant	xxx xx.x%	xxx xx.x%	xxx xx.x%
Heparin (unfractionated or low molecular weight)	xxx xx.x%	xxx xx.x%	xxx xx.x%
Other anticoagulant	xxx xx.x%	xxx xx.x%	xxx xx.x%
**Type of new oral anticoagulant**	xxxx	xxxx	xxxx
Dabigatran	xxx xx.x%	xxx xx.x%	xxx xx.x%
Rivaroxaban	xxx xx.x%	xxx xx.x%	xxx xx.x%
Apixaban	xxx xx.x%	xxx xx.x%	xxx xx.x%
**Type of Heparin**	xxxx	xxxx	xxxx
Subcutaneous low-dose for VTE prophylaxis	xxx xx.x%	xxx xx.x%	xxx xx.x%
Subcutaneous full-dose for treatment	xxx xx.x%	xxx xx.x%	xxx xx.x%
Intravenous therapy	xxx xx.x%	xxx xx.x%	xxx xx.x%
**Antiplatelet agent used**	xxx/xxx xx.x%	xxx/xxx xx.x%	xxx/xxx xx.x%
**Detailed antiplatelet agent**	xxxx	xxxx	xxxx
Aspirin	xxx xx.x%	xxx xx.x%	xxx xx.x%
Clopidogrel	xxx xx.x%	xxx xx.x%	xxx xx.x%
Cilostazol	xxx xx.x%	xxx xx.x%	xxx xx.x%
Dipyridamole	xxx xx.x%	xxx xx.x%	xxx xx.x%
Other antiplatelet agent	xxx xx.x%	xxx xx.x%	xxx xx.x%
**Antihypertension drugs used**	xxx/xxx xx.x%	xxx/xxx xx.x%	xxx/xxx xx.x%
**Type of antihypertension drugs used**	xxxx	xxxx	xxxx
ACE or ARB	xxx xx.x%	xxx xx.x%	xxx xx.x%
Diuretic	xxx xx.x%	xxx xx.x%	xxx xx.x%
Calcium channel blocker	xxx xx.x%	xxx xx.x%	xxx xx.x%
Beta-blocker	xxx xx.x%	xxx xx.x%	xxx xx.x%
Other antihypertensive agent	xxx xx.x%	xxx xx.x%	xxx xx.x%
**Blood glucose lowering agents used**	xxx/xxx xx.x%	xxx/xxx xx.x%	xxx/xxx xx.x%

Variable	Care Bundle (N =)	Usual Care (N =)	Total (N =)
**Type of glucose lowering agents**	xxxx	xxxx	xxxx
Oral agents	xxx xx.x%	xxx xx.x%	xxx xx.x%
Insulin	xxx xx.x%	xxx xx.x%	xxx xx.x%
**Nitrates used**	xxx/xxx xx.x%	xxx/xxx xx.x%	xxx/xxx xx.x%
**Statin or other lipid lowering agent**	xxx/xxx xx.x%	xxx/xxx xx.x%	xxx/xxx xx.x%
**Anti-epilepsy drugs used**	xxx/xxx xx.x%	xxx/xxx xx.x%	xxx/xxx xx.x%
**Type of anti-epilepsy drugs**	xxxx	xxxx	xxxx
Sodium valproate	xxx xx.x%	xxx xx.x%	xxx xx.x%
Levetiracetam	xxx xx.x%	xxx xx.x%	xxx xx.x%
Carbamazepine	xxx xx.x%	xxx xx.x%	xxx xx.x%
Phenytoin	xxx xx.x%	xxx xx.x%	xxx xx.x%
Other anti-epilepsy agents	xxx xx.x%	xxx xx.x%	xxx xx.x%

Table 7. Laboratory and vital measures recorded during the first 24 hours

Variable	Care Bundle (N =)	Usual Care (N =)	Total (N =)
**Highest SBP (mmHg)**	xxx	xxx	xxx
Mean (SD)	xxx.x (xxx.x)	xxx.x (xxx.x)	xxx.x (xxx.x)
Median (Q1; Q3)	xxx (xxx; xxx)	xxx (xxx; xxx)	xxx (xxx; xxx)
min max	xxx to xxx	xxx to xxx	xxx to xxx
**Highest DBP (mmHg)**	xxx	xxx	xxx
Mean (SD)	xxx.x (xxx.x)	xxx.x (xxx.x)	xxx.x (xxx.x)
Median (Q1; Q3)	xxx (xxx; xxx)	xxx (xxx; xxx)	xxx (xxx; xxx)
min max	xxx to xxx	xxx to xxx	xxx to xxx
**Lowest SBP (mmHg)**	xxx	xxx	xxx
Mean (SD)	xxx.x (xxx.x)	xxx.x (xxx.x)	xxx.x (xxx.x)
Median (Q1; Q3)	xxx (xxx; xxx)	xxx (xxx; xxx)	xxx (xxx; xxx)
min max	xxx to xxx	xxx to xxx	xxx to xxx
**Lowest DBP (mmHg)**	xxx	xxx	xxx
Mean (SD)	xxx.x (xxx.x)	xxx.x (xxx.x)	xxx.x (xxx.x)
Median (Q1; Q3)	xxx (xxx; xxx)	xxx (xxx; xxx)	xxx (xxx; xxx)
min max	xxx to xxx	xxx to xxx	xxx to xxx
**Highest blood glucose (mmol/L)**	xxx	xxx	xxx
Mean (SD)	xxx.x (xxx.x)	xxx.x (xxx.x)	xxx.x (xxx.x)
Median (Q1; Q3)	xxx (xxx; xxx)	xxx (xxx; xxx)	xxx (xxx; xxx)
min max	xxx to xxx	xxx to xxx	xxx to xxx
**Lowest blood glucose (mmol/L)**	xxx	xxx	xxx
Mean (SD)	xxx.x (xxx.x)	xxx.x (xxx.x)	xxx.x (xxx.x)
Median (Q1; Q3)	xxx (xxx; xxx)	xxx (xxx; xxx)	xxx (xxx; xxx)
min max	xxx to xxx	xxx to xxx	xxx to xxx
**Highest body temperature (mmHg)**	xxx	xxx	xxx
Mean (SD)	xxx.x (xxx.x)	xxx.x (xxx.x)	xxx.x (xxx.x)
Median (Q1; Q3)	xxx (xxx; xxx)	xxx (xxx; xxx)	xxx (xxx; xxx)
min max	xxx to xxx	xxx to xxx	xxx to xxx
**Lowest body temperature (mmHg)**	xxx	xxx	xxx
Mean (SD)	xxx.x (xxx.x)	xxx.x (xxx.x)	xxx.x (xxx.x)

Variable	Care Bundle (N =)	Usual Care (N =)	Total (N =)
Median (Q1; Q3)	xxx (xxx; xxx)	xxx (xxx; xxx)	xxx (xxx; xxx)
min max	xxx to xxx	xxx to xxx	xxx to xxx
**INR at Day 1**	xxx	xxx	xxx
Mean (SD)	xxx.x (xxx.x)	xxx.x (xxx.x)	xxx.x (xxx.x)
Median (Q1; Q3)	xxx (xxx; xxx)	xxx (xxx; xxx)	xxx (xxx; xxx)
min max	xxx to xxx	xxx to xxx	xxx to xxx

Table 8. Antihypertensive and hypothermia treatments administered during the first 24 hours

Variable	Care Bundle (N =)	Usual Care (N =)	Total (N =)
**IV hypertensive treatment**	xxx/xxx xx.x%	xxx/xxx xx.x%	xxx/xxx xx.x%
**Type of IV antihypertensive treatment**	xxx	xxx	xxx
<treatment 1>	xxx xx.x%	xxx xx.x%	xxx xx.x%
<treatment 2>	xxx xx.x%	xxx xx.x%	xxx xx.x%
Etc. in decreasing order of frequency	xxx xx.x%	xxx xx.x%	xxx xx.x%
**Hypothermia treatment**	xxx/xxx xx.x%	xxx/xxx xx.x%	xxx/xxx xx.x%
**Type of hypothermia treatment**	xxx	xxx	xxx
<treatment 1>	xxx xx.x%	xxx xx.x%	xxx xx.x%
<treatment 2>	xxx xx.x%	xxx xx.x%	xxx xx.x%
Etc. in decreasing order of frequency	xxx xx.x%	xxx xx.x%	xxx xx.x%

Table 9. Management care administered until Day 7

Variable	Care Bundle (N =)	Usual Care (N =)	Total (N =)
IV hypertensive treatment	xxx/xxx xx.x%	xxx/xxx xx.x%	xxx/xxx xx.x%
Oral hypertensive treatment	xxx/xxx xx.x%	xxx/xxx xx.x%	xxx/xxx xx.x%
Insulin	xxx/xxx xx.x%	xxx/xxx xx.x%	xxx/xxx xx.x%
Hypothermia treatment	xxx/xxx xx.x%	xxx/xxx xx.x%	xxx/xxx xx.x%
FFP	xxx/xxx xx.x%	xxx/xxx xx.x%	xxx/xxx xx.x%
Vitamin K	xxx/xxx xx.x%	xxx/xxx xx.x%	xxx/xxx xx.x%
PCC	xxx/xxx xx.x%	xxx/xxx xx.x%	xxx/xxx xx.x%
Mannitol	xxx/xxx xx.x%	xxx/xxx xx.x%	xxx/xxx xx.x%
Hyperosmolar saline	xxx/xxx xx.x%	xxx/xxx xx.x%	xxx/xxx xx.x%
Antibiotic treatment	xxx/xxx xx.x%	xxx/xxx xx.x%	xxx/xxx xx.x%
Anti-epilepsy drugs	xxx/xxx xx.x%	xxx/xxx xx.x%	xxx/xxx xx.x%
Dexamethasone	xxx/xxx xx.x%	xxx/xxx xx.x%	xxx/xxx xx.x%
Statins	xxx/xxx xx.x%	xxx/xxx xx.x%	xxx/xxx xx.x%
Any decompressive surgery	xxx/xxx xx.x%	xxx/xxx xx.x%	xxx/xxx xx.x%
Craniotomy	xxx/xxx xx.x%	xxx/xxx xx.x%	xxx/xxx xx.x%
Craniectomy	xxx/xxx xx.x%	xxx/xxx xx.x%	xxx/xxx xx.x%
Endoscopy	xxx/xxx xx.x%	xxx/xxx xx.x%	xxx/xxx xx.x%
Intraventricular drainage	xxx/xxx xx.x%	xxx/xxx xx.x%	xxx/xxx xx.x%
Catheterisation with/without thrombolysis	xxx/xxx xx.x%	xxx/xxx xx.x%	xxx/xxx xx.x%
Other surgery	xxx/xxx xx.x%	xxx/xxx xx.x%	xxx/xxx xx.x%
Endotracheal intubation	xxx/xxx xx.x%	xxx/xxx xx.x%	xxx/xxx xx.x%
Mechanical ventilation	xxx/xxx xx.x%	xxx/xxx xx.x%	xxx/xxx xx.x%
Airways suction of secretions	xxx/xxx xx.x%	xxx/xxx xx.x%	xxx/xxx xx.x%
Urinary catheter insertion	xxx/xxx xx.x%	xxx/xxx xx.x%	xxx/xxx xx.x%
Intensive care unit admission	xxx/xxx xx.x%	xxx/xxx xx.x%	xxx/xxx xx.x%
Acute stroke unit admission	xxx/xxx xx.x%	xxx/xxx xx.x%	xxx/xxx xx.x%
Pneumatic calf compression	xxx/xxx xx.x%	xxx/xxx xx.x%	xxx/xxx xx.x%
Physiotherapy	xxx/xxx xx.x%	xxx/xxx xx.x%	xxx/xxx xx.x%
Occupational therapy	xxx/xxx xx.x%	xxx/xxx xx.x%	xxx/xxx xx.x%
Psychological therapy	xxx/xxx xx.x%	xxx/xxx xx.x%	xxx/xxx xx.x%
Intravenous traditional Chinese medicine	xxx/xxx xx.x%	xxx/xxx xx.x%	xxx/xxx xx.x%
Intravenous neuroprotective agent	xxx/xxx xx.x%	xxx/xxx xx.x%	xxx/xxx xx.x%
Clinical decision to withdraw active care	xxx/xxx xx.x%	xxx/xxx xx.x%	xxx/xxx xx.x%
Assisted feeding	xxx/xxx xx.x%	xxx/xxx xx.x%	xxx/xxx xx.x%
Ward/Department where patient stayed longest			
Neurosurgery	xxx/xxx xx.x%	xxx/xxx xx.x%	xxx/xxx xx.x%
Neurology	xxx/xxx xx.x%	xxx/xxx xx.x%	xxx/xxx xx.x%

Variable	Care Bundle (N =)	Usual Care (N =)	Total (N =)
Intensive care	xxx/xxx xx.x%	xxx/xxx xx.x%	xxx/xxx xx.x%
Emergency department	xxx/xxx xx.x%	xxx/xxx xx.x%	xxx/xxx xx.x%
Other	xxx/xxx xx.x%	xxx/xxx xx.x%	xxx/xxx xx.x%
Prognosis, will patient survive next 6 months			
Definitely no	xxx/xxx xx.x%	xxx/xxx xx.x%	xxx/xxx xx.x%
Possible no	xxx/xxx xx.x%	xxx/xxx xx.x%	xxx/xxx xx.x%
Uncertain	xxx/xxx xx.x%	xxx/xxx xx.x%	xxx/xxx xx.x%
Possible yes	xxx/xxx xx.x%	xxx/xxx xx.x%	xxx/xxx xx.x%
Definite yes	xxx/xxx xx.x%	xxx/xxx xx.x%	xxx/xxx xx.x%

Table 10. Details of hypertensive treatments between Days 2 and 7

Variable	Care Bundle (N =)	Usual Care (N =)	Total (N =)
**IV antihypertensive treatment**	xxx/xxx xx.x%	xxx/xxx xx.x%	xxx/xxx xx.x%
**Type of IV antihypertensive treatment**	xxx	xxx	xxx
<treatment 1>	xxx xx.x%	xxx xx.x%	xxx xx.x%
<treatment 2>	xxx xx.x%	xxx xx.x%	xxx xx.x%
Etc. in decreasing order of frequency	xxx xx.x%	xxx xx.x%	xxx xx.x%
**Oral hypertensive treatment**	xxx/xxx xx.x%	xxx/xxx xx.x%	xxx/xxx xx.x%
**Type of oral antihypertensive treatment**	xxx	xxx	xxx
<treatment 1>	xxx xx.x%	xxx xx.x%	xxx xx.x%
<treatment 2>	xxx xx.x%	xxx xx.x%	xxx xx.x%
Etc. in decreasing order of frequency	xxx xx.x%	xxx xx.x%	xxx xx.x%

Table 11. Treatment targets

Variable	Care Bundle (N =)	Usual Care (N =)	Total (N =)
**Baseline SBP ≥140 mmHg**	xxx/xxx xx.x%	xxx/xxx xx.x%	xxx/xxx xx.x%
**SBP target <140mmHg) achieved**	xxx/xxx xx.x%	xxx/xxx xx.x%	xxx/xxx xx.x%
**Time to achieving SBP target (hours)**	xxx	xxx	xxx
Mean (SD)	xxx.x (xxx.x)	xxx.x (xxx.x)	xxx.x (xxx.x)
Median (Q1; Q3)	xxx (xxx; xxx)	xxx (xxx; xxx)	xxx (xxx; xxx)
min max	xxx to xxx	xxx to xxx	xxx to xxx
**Baseline Glucose <6.1 or >7.8 mmol/L for non-diabetic (<7.8 or >10.0 mmol/L for diabetic patients)**	xxx/xxx xx.x%	xxx/xxx xx.x%	xxx/xxx xx.x%
**Blood glucose target achieved**	xxx/xxx xx.x%	xxx/xxx xx.x%	xxx/xxx xx.x%
**Time to achieving blood glucose target (hours)**	xxx	xxx	xxx
Mean (SD)	xxx.x (xxx.x)	xxx.x (xxx.x)	xxx.x (xxx.x)
Median (Q1; Q3)	xxx (xxx; xxx)	xxx (xxx; xxx)	xxx (xxx; xxx)
min max	xxx to xxx	xxx to xxx	xxx to xxx
**Baseline Temperature (>37.5 ^{o}C)**	xxx/xxx xx.x%	xxx/xxx xx.x%	xxx/xxx xx.x%
**Temperature target of achieved**	xxx/xxx xx.x%	xxx/xxx xx.x%	xxx/xxx xx.x%
**Time to achieving temperature target (hours)**	xxx	xxx	xxx
Mean (SD)	xxx.x (xxx.x)	xxx.x (xxx.x)	xxx.x (xxx.x)
Median (Q1; Q3)	xxx (xxx; xxx)	xxx (xxx; xxx)	xxx (xxx; xxx)
min max	xxx to xxx	xxx to xxx	xxx to xxx
**Baseline INR ≥ 1.5**	xxx/xxx xx.x%	xxx/xxx xx.x%	xxx/xxx xx.x%
**INR target achieved**	xxx/xxx xx.x%	xxx/xxx xx.x%	xxx/xxx xx.x%
**Time to achieving INR target (hours)**	xxx	xxx	xxx
Mean (SD)	xxx.x (xxx.x)	xxx.x (xxx.x)	xxx.x (xxx.x)
Median (Q1; Q3)	xxx (xxx; xxx)	xxx (xxx; xxx)	xxx (xxx; xxx)
min max	xxx to xxx	xxx to xxx	xxx to xxx

Table 12. Clinical outcomes

Outcome / analysis method	Unadjusted model [1]				Adjusted model [2]			
	N	OR/MD/HR (95% CI)	P	ICC	N	OR/MD/HR (95% CI)	P	ICC
**mRS at Month 6 (ordinal)**								
Common time trend [3]	xxxx	xx.xx (xx.xx to xx.xx)	**0.xxx**	0.xx	xxxx	xx.xx (xx.xx to xx.xx)	0.xxx	0.xx
Multiple imputations (MAR) [5]	*xxxx*	*xx.xx (xx.xx to xx.xx)*	*0.xxx*		*xxxx*	*xx.xx (xx.xx to xx.xx)*	*0.xxx*	
Multiple imputations (+1) [6]	*xxxx*	*xx.xx (xx.xx to xx.xx)*	*0.xxx*		*xxxx*	*xx.xx (xx.xx to xx.xx)*	*0.xxx*	
Multiple imputations (death) [7]	*xxxx*	*xx.xx (xx.xx to xx.xx)*	*0.xxx*		*xxxx*	*xx.xx (xx.xx to xx.xx)*	*0.xxx*	
Varying time trends [4]	xxxx	xx.xx (xx.xx to xx.xx)	0.xxx	0.xx	xxxx	xx.xx (xx.xx to xx.xx)	0.xxx	0.xx
**NIHSS at Day 7 (ordinal)**								
Common time trend	xxxx	xx.xx (xx.xx to xx.xx)	**0.xxx**	0.xx	xxxx	xx.xx (xx.xx to xx.xx)	0.xxx	0.xx
Varying time trends	xxxx	xx.xx (xx.xx to xx.xx)	0.xxx	0.xx	xxxx	xx.xx (xx.xx to xx.xx)	0.xxx	0.xx
**NIHSS at Day 7 (continuous)**								
Common time trend	xxxx	xx.xx (xx.xx to xx.xx)	0.xxx	0.xx	xxxx	xx.xx (xx.xx to xx.xx)	0.xxx	0.xx
Varying time trends	xxxx	xx.xx (xx.xx to xx.xx)	0.xxx	0.xx	xxxx	xx.xx (xx.xx to xx.xx)	0.xxx	0.xx
**Poor outcome (mRS 3-6) at Month 6 (binary)**								
Common time trend	xxxx	xx.xx (xx.xx to xx.xx)	**0.xxx**	0.xx	xxxx	xx.xx (xx.xx to xx.xx)	0.xxx	0.xx
Varying time trends	xxxx	xx.xx (xx.xx to xx.xx)	0.xxx	0.xx	xxxx	xx.xx (xx.xx to xx.xx)	0.xxx	0.xx
**Death at Month 6 (binary)**								
Common time trend	xxxx	xx.xx (xx.xx to xx.xx)	**0.xxx**	0.xx	xxxx	xx.xx (xx.xx to xx.xx)	0.xxx	0.xx
Varying time trends	xxxx	xx.xx (xx.xx to xx.xx)	0.xxx	0.xx	xxxx	xx.xx (xx.xx to xx.xx)	0.xxx	0.xx
**Dependency at Month 6 (binary)**								
Common time trend	xxxx	xx.xx (xx.xx to xx.xx)	**0.xxx**	0.xx	xxxx	xx.xx (xx.xx to xx.xx)	0.xxx	0.xx
Varying time trends	xxxx	xx.xx (xx.xx to xx.xx)	0.xxx	0.xx	xxxx	xx.xx (xx.xx to xx.xx)	0.xxx	0.xx
**EQ-5D scores at Month 6**								
Problems with mobility (ordinal)	xxxx	xx.xx (xx.xx to xx.xx)	0.xxx	0.xx				

Problems with self-care (ordinal)	xxxx	xx.xx (xx.xx to xx.xx)	0.xxx	0.xx				
Problems with usual activities (ordinal)	xxxx	xx.xx (xx.xx to xx.xx)	0.xxx	0.xx				
Problems with pain/discomfort (ordinal)	xxxx	xx.xx (xx.xx to xx.xx)	0.xxx	0.xx				
Problems with anxiety/depression (ordinal)	xxxx	xx.xx (xx.xx to xx.xx)	0.xxx	0.xx				
Overall health utility (continuous)	xxxx	xx.xx (xx.xx to xx.xx)	**0.xxx**	0.xx				
**Time to hospital discharge (survival)**								
Cox model	xxxx	xx.xx (xx.xx to xx.xx)	**0.xxx**	0.xx				
**Place of residence at Month 6 (ordinal)**								
Common time trend	xxxx	xx.xx (xx.xx to xx.xx)	**0.xxx**	0.xx				

1) For ordinal, continuous and binary outcomes, the unadjusted model consists in a generalised linear mixed model with a random effect for cluster (hospital site), a fixed effect indicating the group assignment of each cluster at each step and a fixed categorical effect of time (each step). Appropriate distributions and link functions are used depending on the nature of the outcome. For survival outcomes, we use a Cox model with a random cluster effect, a fixed categorical period effect as well as a binary variable indicating the group allocation during each period in each cluster.
2) Adjusted models include the following baseline covariates: country, mRS before stroke, age (continuous), sex, and baseline NIHSS score (continuous)

Programming notes:

- *add/edit footnotes as appropriate*
- *for the analysis of dependency alone, restrict denominator to subjects who are alive at 6 months (mRS 0-5)*
- *the p-value in bold red indicates the main analysis of the primary outcome*
- *p-values in bold green indicate the main analysis of the 7 secondary outcomes, these will be subject to multiplicity adjustments using the Holm-Sidak correction*
- *should the examination of baseline characteristics by COVID period reveal important changes in patient characteristics over time, additional baseline variables will be added as covariates (see Section 3.8.3)*

Table 13. SAEs

Event	Care Bundle (N =)	Usual Care (N =)	P-value
**Any SAE**	**nEVT nPT(xx.x%)**	**nEVT nPT(xx.x%)**	**0.xx**
Resulted in death	nEVT nPT(xx.x%)	nEVT nPT(xx.x%)	
Life threatening	nEVT nPT(xx.x%)	nEVT nPT(xx.x%)	
Requires prolonged hospitalisation	nEVT nPT(xx.x%)	nEVT nPT(xx.x%)	
Results in persistent or severe disability/incapacity	nEVT nPT(xx.x%)	nEVT nPT(xx.x%)	
Results in congenital anomaly/birth defect	nEVT nPT(xx.x%)	nEVT nPT(xx.x%)	
Medically significant	nEVT nPT(xx.x%)	nEVT nPT(xx.x%)	
**MedDRA body system 1**			
MedDRA preferred term a	nEVT nPT(xx.x%)	nEVT nPT(xx.x%)	
MedDRA preferred term b	nEVT nPT(xx.x%)	nEVT nPT(xx.x%)	
MedDRA preferred term c	nEVT nPT(xx.x%)	nEVT nPT(xx.x%)	
Etc. by descending frequency of body system and descending frequency of preferred term			

Programming note:
AEs will be summarised as the number (nPT) and proportion of patients experiencing at least one event. In addition, the total number of events (nEVT) will be reported.

Table 14. Causes of death

Cause	Care Bundle (N =)	Usual Care (N =)
**Proximate cause of death**	N=XXX	N=XXX
Most common cause #1	nnn xx%	nnn xx%
Most common cause #2	nnn xx%	nnn xx%
Etc.		
Most common cause #10	nnn xx%	nnn xx%
All other causes	nnn xx%	nnn xx%
**Underlying causes of death**	N=XXX	N=XXX
Most common cause #1	nnn xx%	nnn xx%
Most common cause #2	nnn xx%	nnn xx%
Etc.		
Most common cause #10	nnn xx%	nnn xx%
All other causes	nnn xx%	nnn xx%
**Deaths related to COVID-19**	N=XXX	N=XXX
Most common cause/association #1	nnn xx%	nnn xx%
Most common cause/association #2	nnn xx%	nnn xx%
Etc.		
Most common cause/association #10	nnn xx%	nnn xx%
All other causes/associations	nnn xx%	nnn xx%

Programming notes:

- *Order causes of death by descending frequency.*
- *Do not list categories with 0 deaths.*
- *Use number who died*
- *Depending on the distribution of deaths, we may choose to only report the x (e.g. 10) most common in the publication; however, the original table should include all causes with at least one patient. The breakdown of COVID-19 deaths by cause/association will depend on the final numbers and might include less than 10 categories.*

Table 15. Protocol violations and deviations

Cause	Care Bundle (N =)	Usual Care (N =)
**Randomisation violations**	N=XXX	N=XXX
Age <18 years	nnn xx%	nnn xx%
Acute stroke syndrome not primary ICH	nnn xx%	nnn xx%
Presentation >6 hours from last seen well	nnn xx%	nnn xx%
**Non-compliance with intervention**	N=XXX	N=XXX
Reason 1	nnn xx%	nnn xx%
Reason 2	nnn xx%	nnn xx%
Etc.	nnn xx%	nnn xx%
**Missing primary outcome**	N=XXX	N=XXX
Refused in-person or telephone assessment	nnn xx%	nnn xx%
Lost to follow-up	nnn xx%	nnn xx%
Etc.	nnn xx%	nnn xx%

Programming notes:

- *Reasons for non-compliance and missing outcome will be defined while blinded.*

Table 16. Form of assessment of 6-month outcomes

Cause	Care Bundle (N =)	Usual Care (N =)
**Phone to patient**	nnn xx%	nnn xx%
**Face-to-face with patient**	nnn xx%	nnn xx%
**Phone to caregiver**	nnn xx%	nnn xx%
**Phone to patient's doctor or medical practitioner**	nnn xx%	nnn xx%
**Non-compliance with intervention**	nnn xx%	nnn xx%
**Other source of information**	nnn xx%	nnn xx%

5.2 Figures

Figure 1. CONSORT diagram

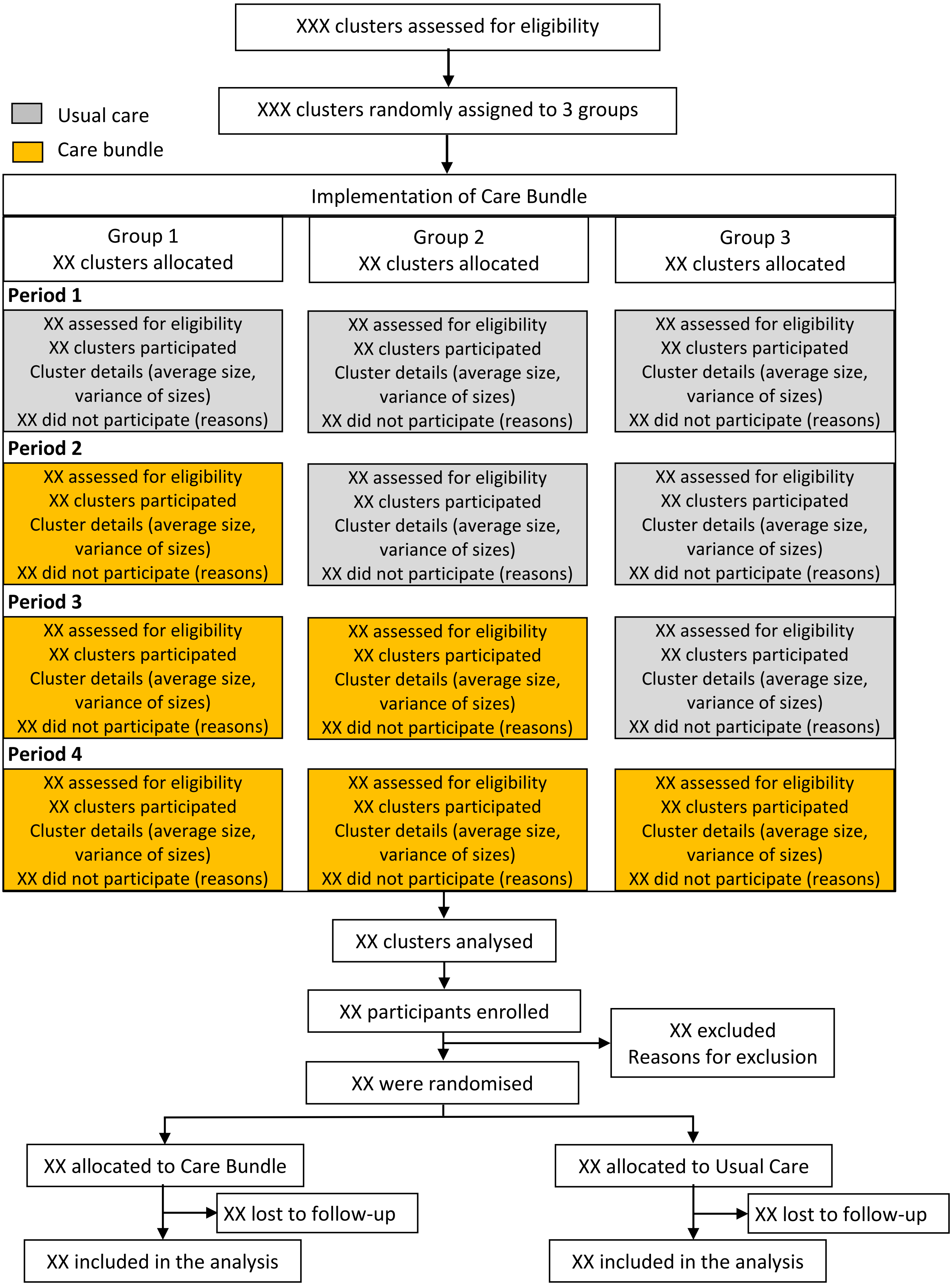

Figure 2. Start and stop dates of each period with number recruited per site per country

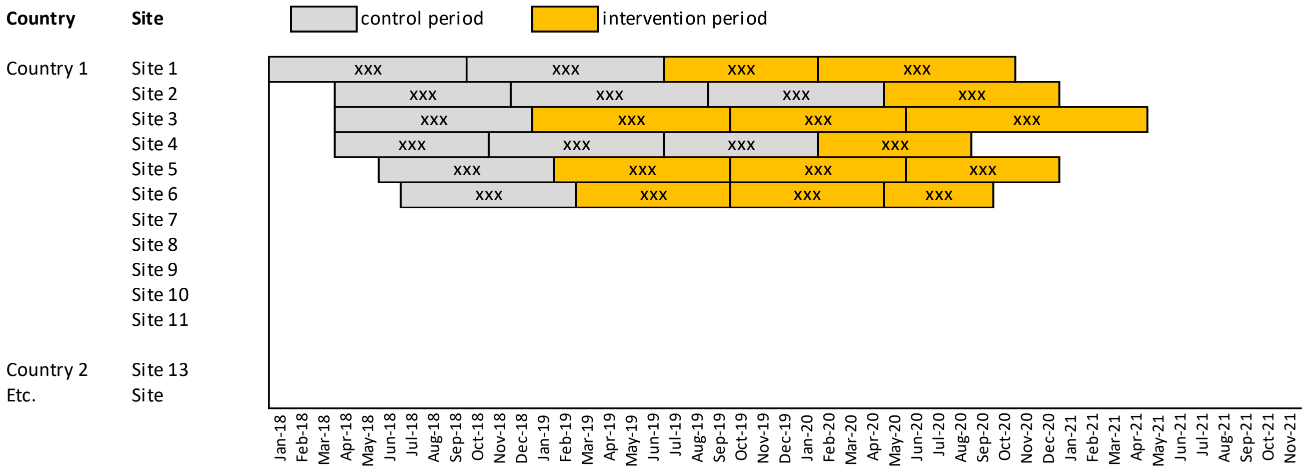

Programming note: order by country and then by randomised group and then by start date

Figure 3. Systolic blood pressure over time

Programming note: Longitudinal mean plot with 95% confidence intervals. Display denominators (N) under the x-axis at key timepoints.

Figure 4. Diastolic blood pressure over time

Programming note: Longitudinal mean plot with 95% confidence intervals. Display denominators (N) under the x-axis at key timepoints.

Figure 5. Blood glucose over time

Programming note: Longitudinal mean plot with 95% confidence intervals. Display denominators (N) under the x-axis at key timepoints.

Figure 6. Body temperature over time

Programming note: Longitudinal mean plot with 95% confidence intervals. Display denominators (N) under the x-axis at key timepoints.

Figure 7. Grotta bar charts of mRS

Programming note: Stacked bar chart with 2 bars (intervention vs control) per visit. Each bar to be of the same high (100%). Show the proportion in each category using labels on the bars.

Figure 8: Boxplot of hematoma volume by follow-up assessment

Figure 9: Boxplot of NIHSS by follow-up assessment

Figure 10. Forest plot for subgroup analysis of mRS at 6 months

Figure 11. Cumulative incidence function of time to hospital discharge

Programming note: add number at risk every 10 days, median, quartiles, hazard ratio, 95% CI and P value from the Cox model.

Figure 12. Kaplan Meier curve of mortality over 6 months

Programming note: add number at risk every 10 days, median, quartiles, hazard ratio, 95% CI and P value from the Cox model.

5.3 Listings

Listing 1. Protocol deviations

Site ID	Patient ID	Date	Type of deviation	Description	Corrective action taken
6	6001	27-Sep-10			
	6008	20-Sep-10			
	6052	26-Jun-11			
	6054	21-Jun-11			

6 Proposed content and timing of primary and subsequent publications

N	2023
1	Main results paper: treatment effects of care bundle on primary and secondary efficacy and safety outcomes, and according to pre-specified subgroups
2	Further subgroup analysis: relation of treatment effects by time, ethnicity, age, and neurological severity
3	Further subgroup analysis: differential treatment effects by surgical management
4	Further subgroup analysis: differential treatment effects by baseline neurological severity
5	Overall and individual treatment effects on haematoma growth and perihaematomal oedema
6	Effect of compliance including per-protocol analyses
	**2024 and subsequent years**
7	Treatment effects according to utility-weighted mRS scores
8	Association of achieved blood pressure and glucose levels and outcomes
9	Feasibility and cost-effectiveness of care bundle for ICH
10	Influence of specific surgical interventions on outcome from ICH – propensity score analysis
11	Accuracy of clinician predictions of outcome from ICH
12	Clinical and imaging predictors of poor outcome
13	Health economic analysis: estimating cost-effectiveness of the intervention
14	Patterns and predictors of major outcomes (death, recurrent ICH, coronary events, falls and re-admission to hospital) after ICH
15	Determinants of HRQoL and influence of age, sex, ethnicity, and level of disability
16	Regional determinants of presentation and treatment times in ICH
17	Regional variation in the management of intracerebral haemorrhage
18	Frequency, predictors, and prognostic significance of seizures after ICH
19	Patterns and determinants of antithrombotic and statin use after ICH
20	Relation of clinical profile to recovery patterns after ICH
21	Patterns of recovery according to mRS scores over 6 months follow-up
22	Clinical-radiological correlations of baseline imaging and clinical and pathological classifications
23	Inclusion of data in systematic reviews/meta-analyses including the Blood pressure in Acute Stroke Collaboration (BASC) and of brain imaging determinants of outcome
